# Encoding stability and dynamics in hydrogen-bonded octameric capsules through amino acid α-position design

**DOI:** 10.1039/d6qo01259k

**Published:** 2026-11-06

**Authors:** Filip Perlitius, Grzegorz Markiewicz, Mateusz Gołdyn, Gokay Avci, Kim E. Jelfs, Artur R. Stefankiewicz

**Affiliations:** a Center for Advanced Technologies, Adam Mickiewicz University Uniwersytetu Poznańskiego 10 61-614 Poznań Poland ars@amu.edu.pl; b Faculty of Chemistry, Adam Mickiewicz University Uniwersytetu Poznańskiego 8 61-614 Poznań Poland; c Department of Chemistry, Molecular Sciences Research Hub, Imperial College London 82 Wood Lane London W12 0BZ UK; d Department of Chemistry, University College London Christopher Ingold Building 20 Gordon St London WC1H 0AJ UK

## Abstract

How local molecular determinants encode stability and dynamics in supramolecular assemblies remains a central challenge in adapting biological design principles for synthetic systems. Here, we systematically investigate eleven amino acid-functionalized benzene-1,3,5-tricarboxamides to elucidate how chirality, α-substitution, and side-chain architecture control the formation, stability and function of noncovalent octameric capsules. Achiral derivatives lacking an α-hydrogen exhibit poor solubility in noncompetitive solvents and preferentially form extended polymeric aggregates, highlighting the role of the α-H motif in mediating solvation and preorganization of hydrogen-bonding networks. In contrast, chiral analogues retaining the α-H group readily self-assemble into discrete, *D*_2_-symmetric octameric capsules. Substitution of the α-hydrogen with a methyl group disrupts solubility and capsular stability; however, introduction of long aliphatic side chains partially restores capsule formation, albeit with increased dynamics. Moreover, α-methylation profoundly affects capsule function, leading to a complete loss of fullerene encapsulation ability compared to the corresponding α-H-containing capsules. Overall, these results reveal how subtle variations at the α-position translate into emergent differences in stability, dynamics, function and architecture of hydrogen-bonded supramolecular capsules.

## Abstract 2

Subtle changes at the α-position of amino acid-derived BTAs profoundly affect their self-assembly. Chirality, α-substitution, and side-chain architecture govern the stability, dynamics, and fullerene-binding ability of hydrogen-bonded capsules.

## Graphical abstract

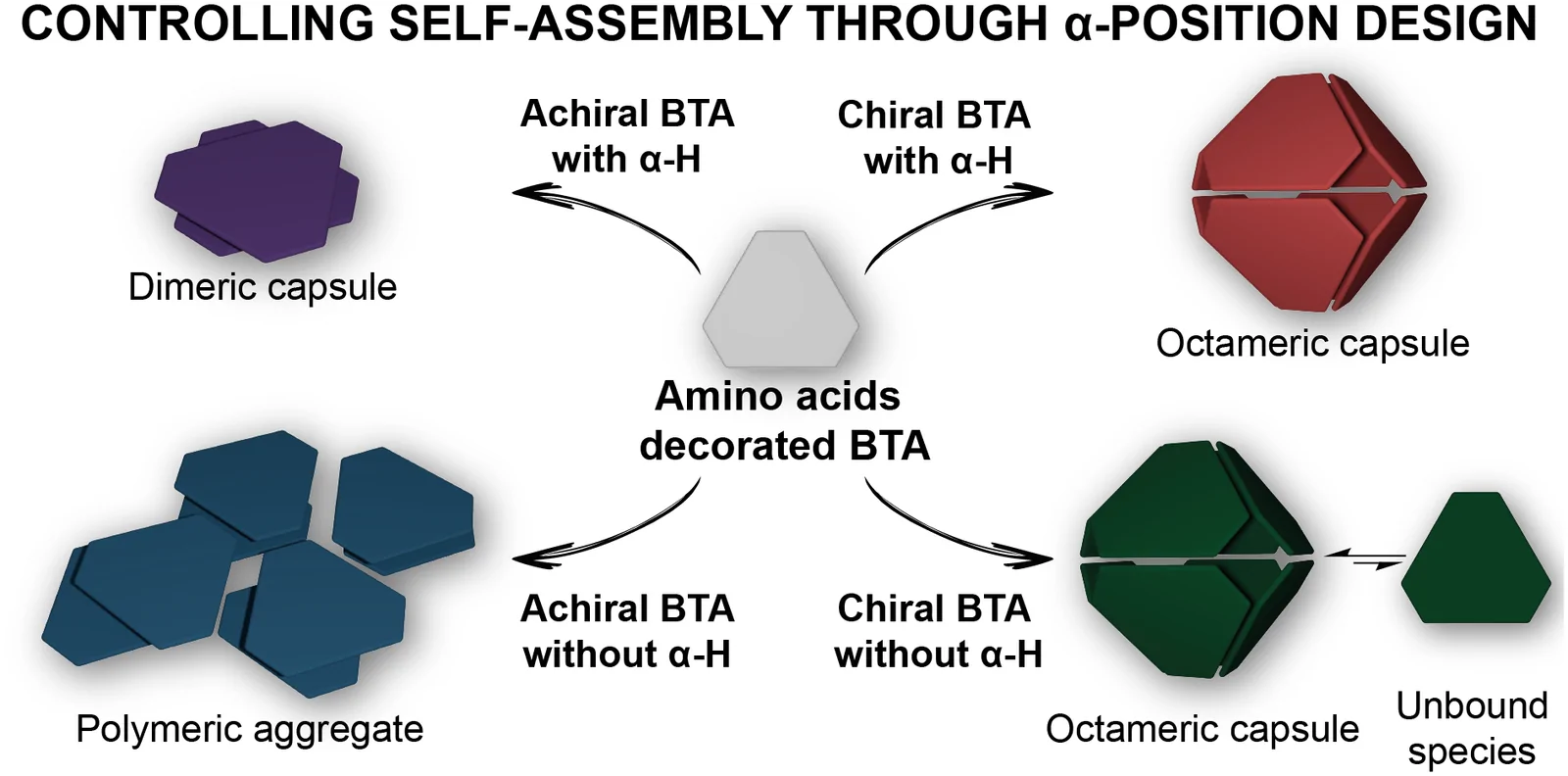

Nature constructs highly complex and multidimensional molecular architectures from a limited set of simple chemical building blocks, translating subtle local structural information into precisely organized and functionally adaptive systems.^1^ Among these building blocks, amino acids play a central role, enabling the formation of polymeric structures that span multiple length scales, from short oligopeptides to folded, three-dimensional proteins.^2,3^ Beyond covalent connectivity, amino acids also engage in highly directional noncovalent interactions, allowing them to assemble into supramolecular biomolecules^4^ whose stability and dynamics are finely balanced to support biological function.^5,6^ A striking feature of biological self-assembly is its reliance on a narrowly defined molecular alphabet: the overwhelming majority of protein-based supramolecular architectures are constructed from twenty homochiral α-CH amino acids, a structural motif that is only rarely modified in nature.^7^ α,α-Disubstituted amino acids represent a notable exception.^8–11^ Although non-proteinogenic, they are key components of biologically active compounds and are distinguished by enhanced resistance to racemization and restricted conformational flexibility – features that often translate into increased stability and rigidified secondary structural motifs.^12–14^

A defining yet often overlooked feature of amino acids in biological systems is the presence of a single hydrogen atom at the α-carbon.^5,8^ This substituent plays a disproportionate role in encoding conformational adaptability, enabling polypeptide backbones to access compact secondary structures while minimizing steric congestion.^6,8,15^ The functional importance of this design is underscored by notable exceptions: glycine, bearing two α-hydrogens, imparts exceptional local flexibility, whereas proline, in which the α-hydrogen is effectively replaced by a cyclic constraint, introduces rigidity and disrupts regular secondary structure.^15,16^ More broadly, the near-universal reliance of biological systems on α-CH amino acids highlights how subtle variations at the α-position translate into profound consequences for structural organization and dynamics.^15,16^ Despite this central role in biology, the influence of α-hydrogen substitution on supramolecular self-assembly remains poorly understood. In particular, it is unclear how replacing the α-H with sterically demanding groups affects the delicate balance between solubility, stability, and adaptability in hydrogen-bonded synthetic assemblies. The modularity and structural definition of amino acids make them particularly well suited for dissecting these relationships in a fully synthetic systems.

Inspired by these principles, amino acids have become key synthons in supramolecular chemistry,^17–19^ particularly in the design of hydrogen-bonded systems.^20–24^ In this context, amino acid-functionalized benzene-1,3,5-tricarboxamides (BTAs) have emerged as versatile building blocks for constructing diverse supramolecular architectures.^25–27^ Such systems have been explored across a broad range of assemblies, including disulfide-linked capsules,^28–30^ coordination cages,^24,31–33^ metal–organic frameworks (MOFs),^34^ hydrogen-bonded dimers,^35^ gels,^36^ columnar polymers,^35,37,38^ and discrete hydrogen-bonded capsules.^39–41^

Despite this progress, a fundamental gap remains. While numerous hydrogen-bonded capsules and amino acid-derived supramolecular systems have been reported, the field still lacks transferable molecular principles that explain how local amino-acid features such as α-substitution, chirality, and polarity govern three interdependent parameters that ultimately determine whether a capsule is accessible and functional in solution: solubility, structural stability, and dynamic behaviour. As a consequence, the rational design of adaptive supramolecular capsules remains largely empirical, with limited predictive power regarding whether a given molecular modification will promote robust capsule formation, induce uncontrolled aggregation, or render assemblies inaccessible due to poor solubility.^42^

In our previous work, we demonstrated that BTAs decorated with three homochiral *S*-trityl-l-cysteine moieties self-assemble into octameric nanocapsule stabilized by 48 NH⋯O=C and OH⋯O=C hydrogen bonds.^41^ While this system established amino acid-derived BTAs as robust capsular synthons, it also raised fundamental questions regarding the molecular features that encode capsule accessibility, robustness, and adaptability. In particular, the roles of chirality, substitution at the α-position, and side-chain architecture in coupling local molecular structure to global capsular behaviour have not been systematically dissected. Here, we address this challenge through a systematic study of three classes of amino acid-derived BTAs designed to (i) eliminate point chirality within the side chains, (ii) vary aliphatic and aromatic side-chain structures, and (iii) replace the α-CH motif with sterically demanding methyl substituents. By correlating molecular structure with self-assembly behaviour in both the solid and solution states, we reveal how subtle variations in steric congestion, solubility, and local polarity translate into pronounced differences in the stability, dynamics, and accessibility of hydrogen-bonded supramolecular capsules. These findings establish a set of transferable design principles that connect local amino-acid structure to emergent supramolecular behaviour, advancing the rational design of adaptive hydrogen-bonded assemblies beyond empirical trial-and-error approaches.

## Results and discussion

### Molecular design

The design of components 1–11 is reminiscent of our previously reported *S*-Tr-cysteine-derived synthon, which, in non-competitive media, self-assembles to yield a hydrogen-bonded octameric capsule.^41^ Specifically, the benzene-1,3,5-tricarboxamide (BTA) core was decorated with three amino acid side chains, providing secondary amido and carboxylic acid centres for hydrogen-bonded driven self-assembly. To interrogate the structural factors governing octamer formation, components 1–11 were intentionally modified with respect to the three distinct molecular features:

#### Chirality

The use of proteogenic amino acids inherently introduces chirality into the BTA side chains. However, a detailed analysis of the crystal structure of the octameric capsule suggests that chirality is not a primary determinant of capsule formation. The chiral side chains are consistently oriented outward, away from the hydrogen-bonded capsular core, suggesting that their stereochemistry does not play a direct role in guiding or stabilizing the assembly. However, the only viable strategy for accessing achiral products, without disrupting the NH/COOH hydrogen-bonding centres, relies on the glycine motif. Glycine, the simplest amino acid, lacks point chirality at the α-position due to the presence of two identical hydrogen substituents. Based on this principle, component 1 was designed with three glycine arms attached to the BTA core (Fig. 1a, top left), yielding an achiral BTA derivative bearing α-H units. To address concerns that the high polarity of compound 1 might impair its solubility in non-polar media (*vide infra*), the glycine motif was systematically modified to generate a series of analogues incorporating hydrophobic groups at the α-position. These included dimethyl (2), cyclopropyl (3), cyclobutyl (4), cyclohexyl (5), and bis-*n*-butyl (6) substituents. Each was designed to maintain two identical substituents at the α-position, thereby eliminating point chirality while simultaneously introducing progressively bulkier aliphatic groups to reduce overall polarity. Notably, these modifications simultaneously remove the α-H unit and introduce increasing steric congestion at the α-position. This design enabled us to decouple the roles of chirality, steric bulk, and polarity in governing self-assembly behaviour (Fig. 1a, bottom).

**Fig. 1:**
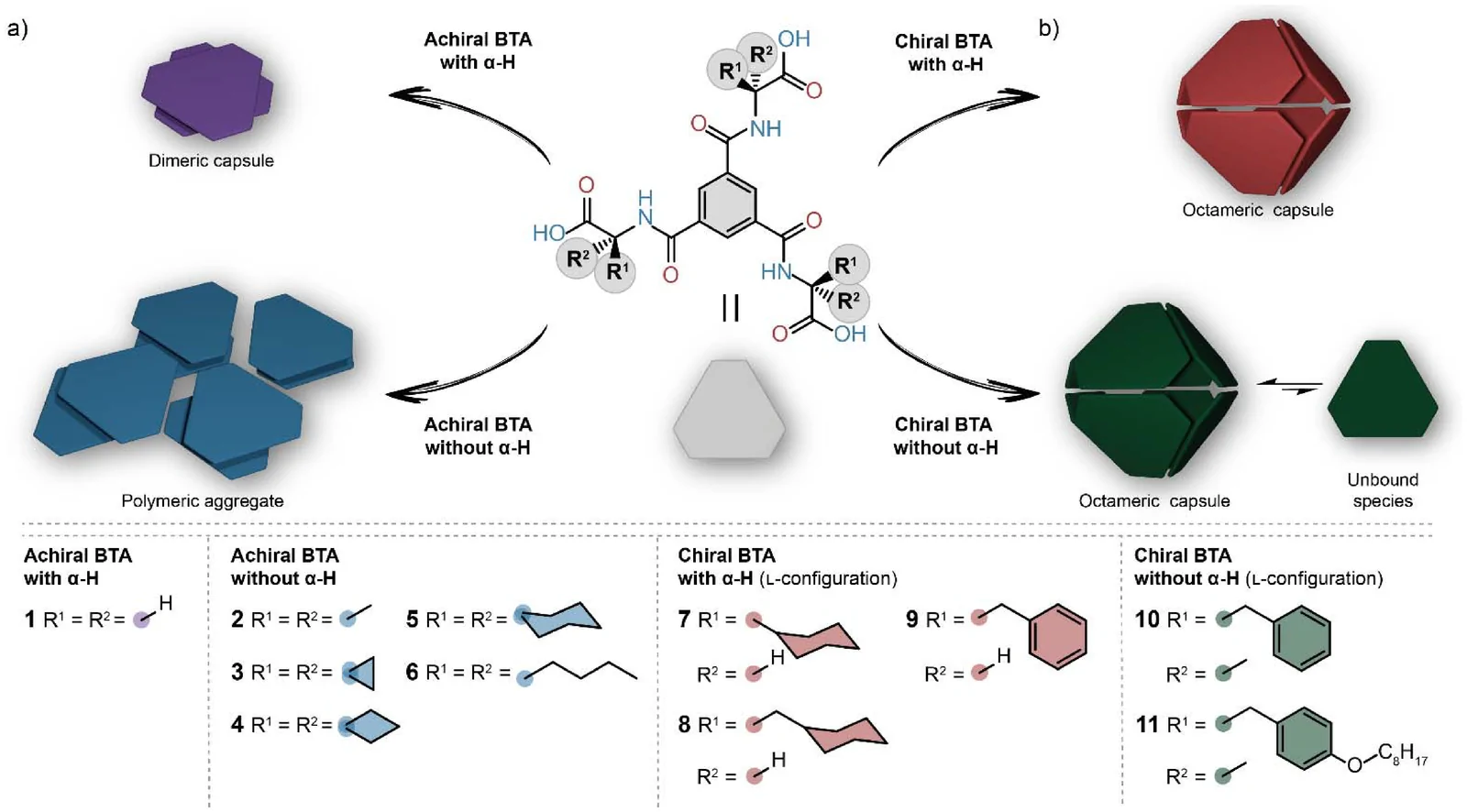
Molecular design of components 1–11 along with the schematic representation of their self-assembly outcomes: (a) Achiral pathway; (b) Chiral pathway.

#### Side chain

The second modification strategy involved altering the amino acid side chains. As noted above, these side chains are not directly involved in the self-assembly of an octamer, suggesting that the originally reported –CH_2_*S*-Tr units can be systematically replaced by other neutral groups without significantly affecting the assembly outcome. This variation is represented by components 7–9, which bear cyclohexyl (7), cyclohexyl –CH_2_– (8), and phenylalanine (9) side chains, respectively. Derivative 7 constitutes a structural variation of an achiral derivative 5, in which the cyclohexyl ring has been repositioned further from the α-C, thereby restoring the presence of an α-H unit. Building on this design, derivative 8 introduces an additional methylene (–CH_2_–) spacer, shifting the steric bulk even further away from the BTA core. These modifications were intended to probe whether the assembly behaviour is dictated by the cyclohexyl group itself or by the steric effects arising from its proximity to the core. In contrast, derivative 9, which is chemically closest to our previously reported *S*-Tr-cysteine-based synthon, restores the aromatic character of the side chains, potentially enabling π–π interactions while simultaneously reducing steric bulk. Importantly, mono-substitution at the α-C reintroduces point chirality, resulting in 7, 8, and 9 each possessing three (lll) homochiral arms around the BTA core.

#### α-H

The third and final modification of the original *S*-Tr-cysteine-derived BTA motif^41^ specifically targeted the α-position, aiming to replace the α-H atom while preserving the stereogenic centres (lll) in the resulting compounds. In this case, α-H was substituted with methyl (–Me) groups to determine their influence on self-assembly behaviour. Although the crystal structure of *S*-Tr-cysteine-derived BTA^41^ reveals that α-H is not directly involved in self-assembly, being located within the internal cavity of the octamer, we hypothesized that introducing steric bulk at this position could perturb capsule formation and alter its physicochemical properties. This series is represented by components 10 and 11, both featuring α-Me groups with phenylalanine (10) and *C*_8_-decorated tyrosine (11) side chains, respectively. Together, these three design axes provided a structurally diverse yet conceptually unified library for probing how local molecular features translate into distinct self-assembly pathways.

### Synthesis

Despite the similarities in molecular design, the synthetic procedures leading to the formation of components 1–11 had to be substantially adapted to overcome the reduced nucleophilic activity and steric demands of amino acids bearing sterically demanding α-substitution. Specifically, three independent strategies were employed to generate the anticipated amino acid functionalized components (Scheme 1).

**Scheme 1:**
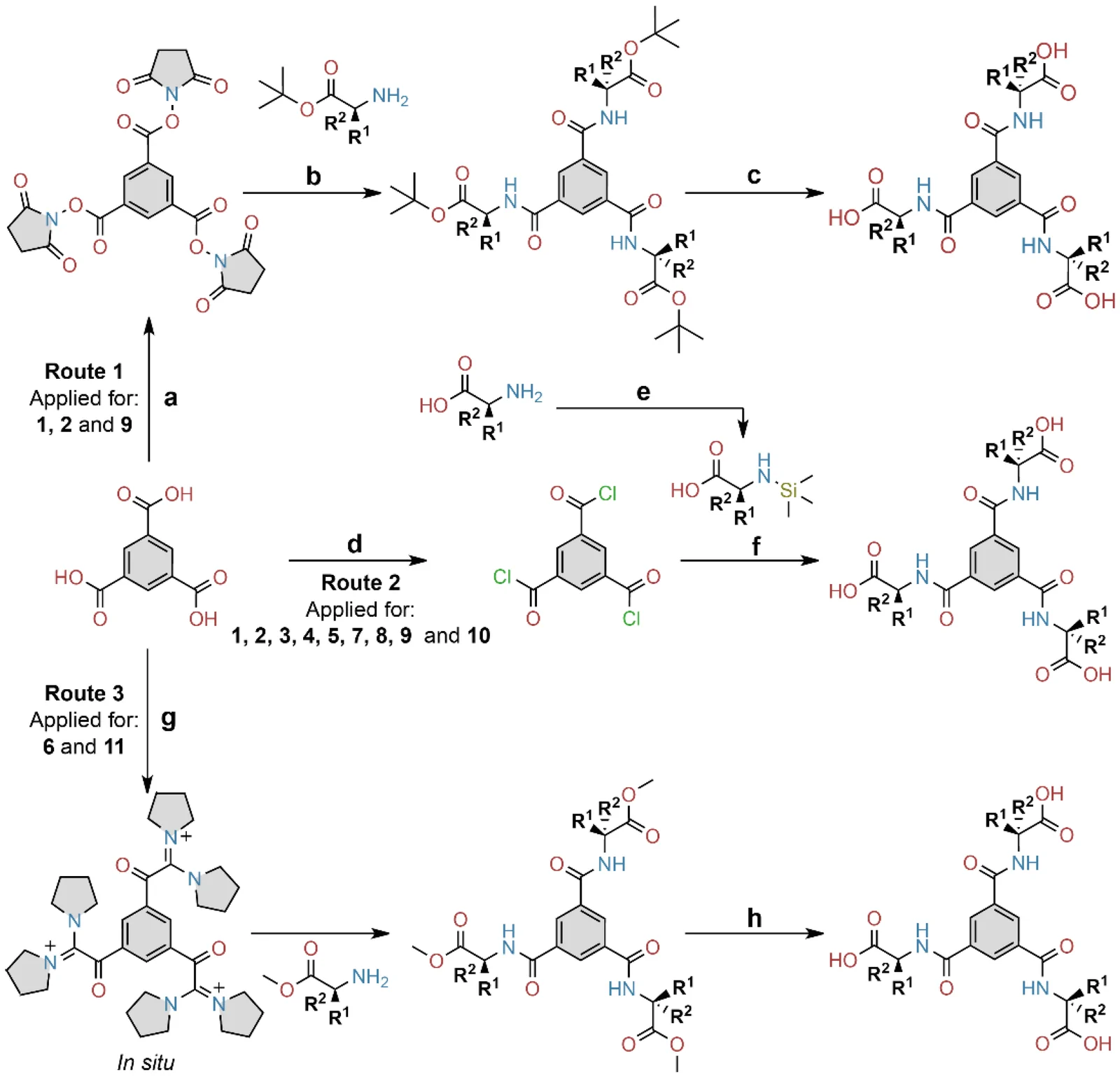
Synthesis of 1–11. (a) NHS, EDC, DMF. (b) DCM, DMF, Et_3_N. (c) TFA, TES, DCM. (d) SOCl_2_, DMF(cat). (e) TMS-Cl, DCM. (f) DIPEA. (g) BTFFH, DIPEA, DCM. (h) LiOH, THF/H_2_O 4 : 1.

#### Route 1 (applied for 1, 2 and 9)

Benzene-1,3,5-tricarboxylic acid was first converted to its *N*-hydroxysuccinimide ester (NHS) and then reacted with an excess (1.66 equiv. per ester group) of a *tert*-butyl-protected amino acid of choice. The introduction of amino acids in the form of *tert*-butyl esters significantly facilitated the isolation and purification of products, allowing the crude materials to be chromatographed on standard silica gel. Subsequent reductive deprotection of the C-termini *tert*-butyl esters under acidic conditions (TFA, Et_3_SiH) afforded the triacid products 1, 2 and 9 in overall yields exceeding 60%. However, despite multiple attempts, this route proved ineffective for the preparation of compounds 3, 4, 5, 6, and 11, resulting only in mixtures of partially substituted BTA derivatives. As a result, alternative reaction conditions were explored to overcome these limitations.

#### Route 2

In this approach, benzene-1,3,5-tricarboxylic acid was first treated with an excess of SOCl_2_ to afford the corresponding triacyl trichloride – a more active and less sterically hindered intermediate for BTA synthesis. The triacyl trichloride was then added to an alkalized (*N*,*N*-diisopropylethylamine, DIEA) mixture containing an excess of amino acid (1.33 equiv. per acyl chloride unit), which had been pre-activated using trimethylsilyl chloride (TMSCl). This dual-activation protocol enabled complete tris-substitution of the BTA core, successfully yielding not only previously accessible derivatives 1, 2, and 9 (in improved yields) but also sterically demanding variants 3, 4, 5, 7, 8 and 10. Notably, this method eliminated the need for C-terminus protection/deprotection steps, as the desired products could be obtained in high purity following simple aqueous and diethyl ether washes. Owing to its broad applicability and operational simplicity, Route 2 emerged as a particularly versatile strategy for synthesizing this class of compounds.

#### Route 3

Despite the efficiency of Routes 1 and 2, neither method was successful in synthesizing derivatives 6 and 11. These were instead obtained *via in situ* activation of benzene-1,3,5-tricarboxylic acid with fluoro-*N*,*N*,*N*′,*N*′-bis(tetramethylene) formamidinium hexafluorophosphate (BTFFH)^43^ under basic (DIEA) conditions. The resulting triacyl trifluoride intermediate was reacted with an excess of C-terminally protected (–OMe) amino acids to afford the corresponding methyl esters 6 –OMe and 11 –OMe. After purification, the C-termini were deprotected through mild saponification (LiOH), resulting in pure 6 and 11 components, respectively.

Together, these three synthetic routes enabled the preparation of all eleven target compounds with satisfactory yields (44–80%), thereby providing a robust library for subsequent structural and self-assembly studies.

### Characterization in solution

To confirm the structures and chemical purity of components 1–11, their monomeric forms were characterized by ^1^H and ^13^C NMR spectroscopy. DMSO-*d*_6_ was selected as the solvent for these measurements, as it effectively dissolved all compounds and, owing to its high polarity and strong solvation capacity, efficiently disrupted any intermolecular interactions between the solvated monomers. As shown in Fig. 2, selected components for each group 1, 5, 9, and 11 display the expected *C*_3_ symmetry around the BTA core, with characteristic resonances of the DMSO-solvated NH and COOH groups appearing at approximately 9.0 ppm and 12.5 ppm, respectively. For derivatives bearing α-H atoms, the corresponding signals exhibited distinct splitting patterns – either doublets for components 1 and 7, or doublet of doublets for 8 and 9 – arising from coupling with diastereotopic β-CH_2_ protons. The corresponding spectra for all investigated derivatives (1–11) are provided in Fig. S77 in the SI.

**Fig. 2:**
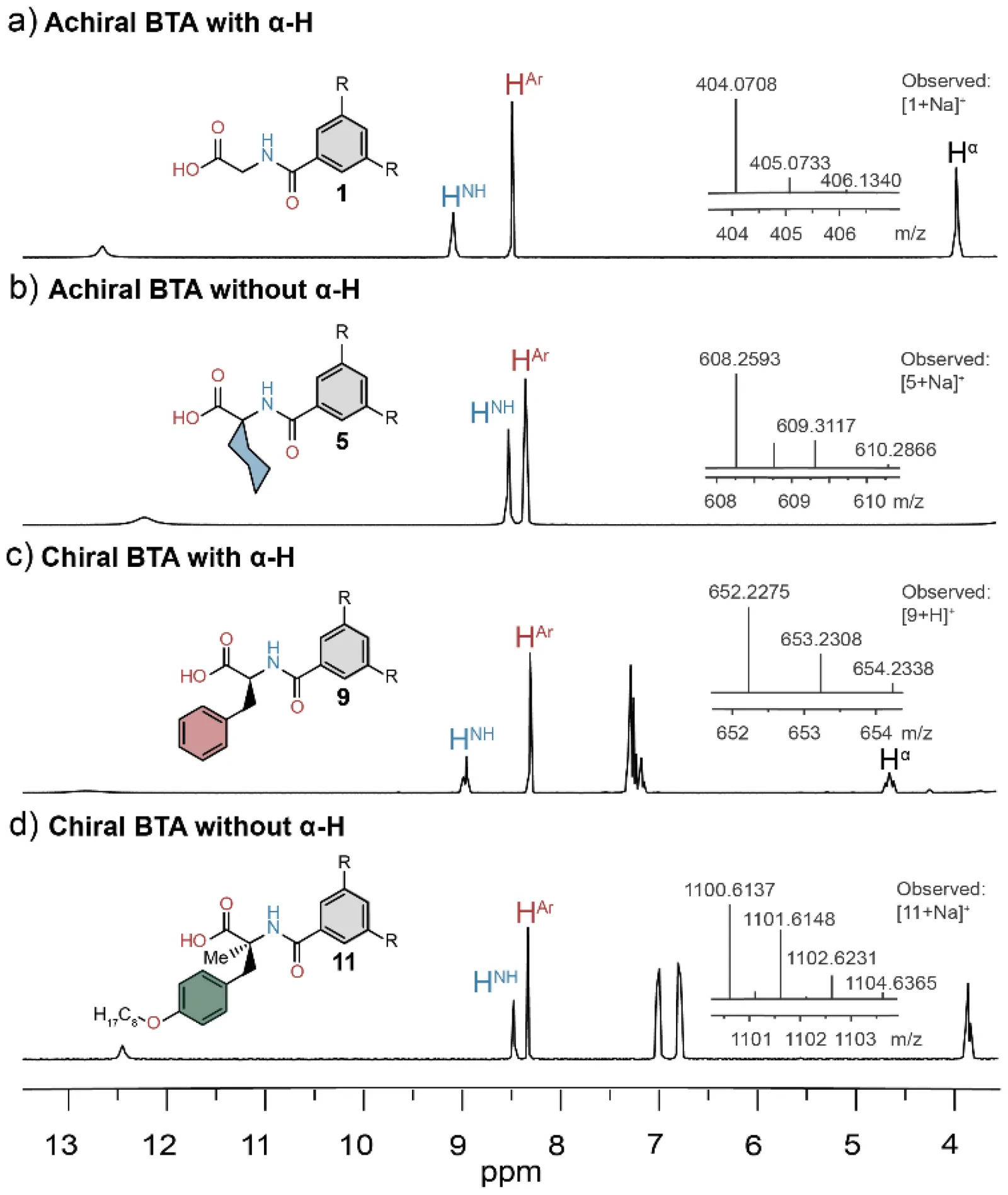
^1^H NMR (600 MHz, DMSO-*d*_6_) spectra of (a) 1, (b) 5, (c) 9 and (d) 11. Insets: HR-ESI-MS+ spectra of (a) 1, (b) 5, (c) 9 and (d) 11 as obtained from MeOH solutions.

Further evidence for the successful formation of components 1–11 was obtained from HR-ESI-MS analysis. Upon dissolution in methanol, all compounds produced the expected molecular ion peaks corresponding to singly charged [M + Na]^+^ or [M + H]^+^ adducts (see insets in Fig. 2 and Fig. S77 in the SI).

### Characterization in the solid-state

Single crystals of 1·3H_2_O (Fig. 3a), previously reported by Y. Yan *et al.*,^44^ were obtained by slow cooling of its hot aqueous solution. Molecule 1 exhibits *C*_3_ symmetry, with a threefold axis passing through the centre of the aromatic ring, and all three amide arms are oriented on the same side of the benzene plane. The torsion angle C2(Ar)–C1(Ar)–C3–O3 is 21.0(2)° (Fig. S129 in SI) consistent with a *ttg*^+^ conformation of the side chains. The absence of substitution at the α-position enables uniform alignment of all arms and promotes the formation of centrosymmetric, spiral-type [6–6/0] dimers with *S*_6_ symmetry, stabilized by N–H(amide)⋯O(acid) hydrogen bonds. Additional C–H⋯O interactions involving aromatic protons, together with π–π stacking between aromatic cores (centroid–centroid distance 3.618(2) Å), further stabilize the dimeric units. Hydrogen bonding involving water molecules links the dimers into supramolecular chains. Importantly, the outward orientation of the α-hydrogen atoms facilitates additional weak intermolecular contacts (C–H⋯O and C–H⋯π), reinforcing the packing without disrupting the overall symmetry. As a result of the lack of steric hindrance in the form of an alkyl substituent in the α-position of the glycine fragment also affects the self-assembly of molecules in the crystal lattice. Interestingly, despite the absence of side chains imposing a defined orientation and increased lability, all arms adopt the same directional alignment, leading to a fully symmetrical configuration. The α-H atoms are directed outward from the forming dimer, which promotes the formation of non-covalent interactions involving these hydrogen atoms with molecules of neighbouring dimers.

**Fig. 3:**
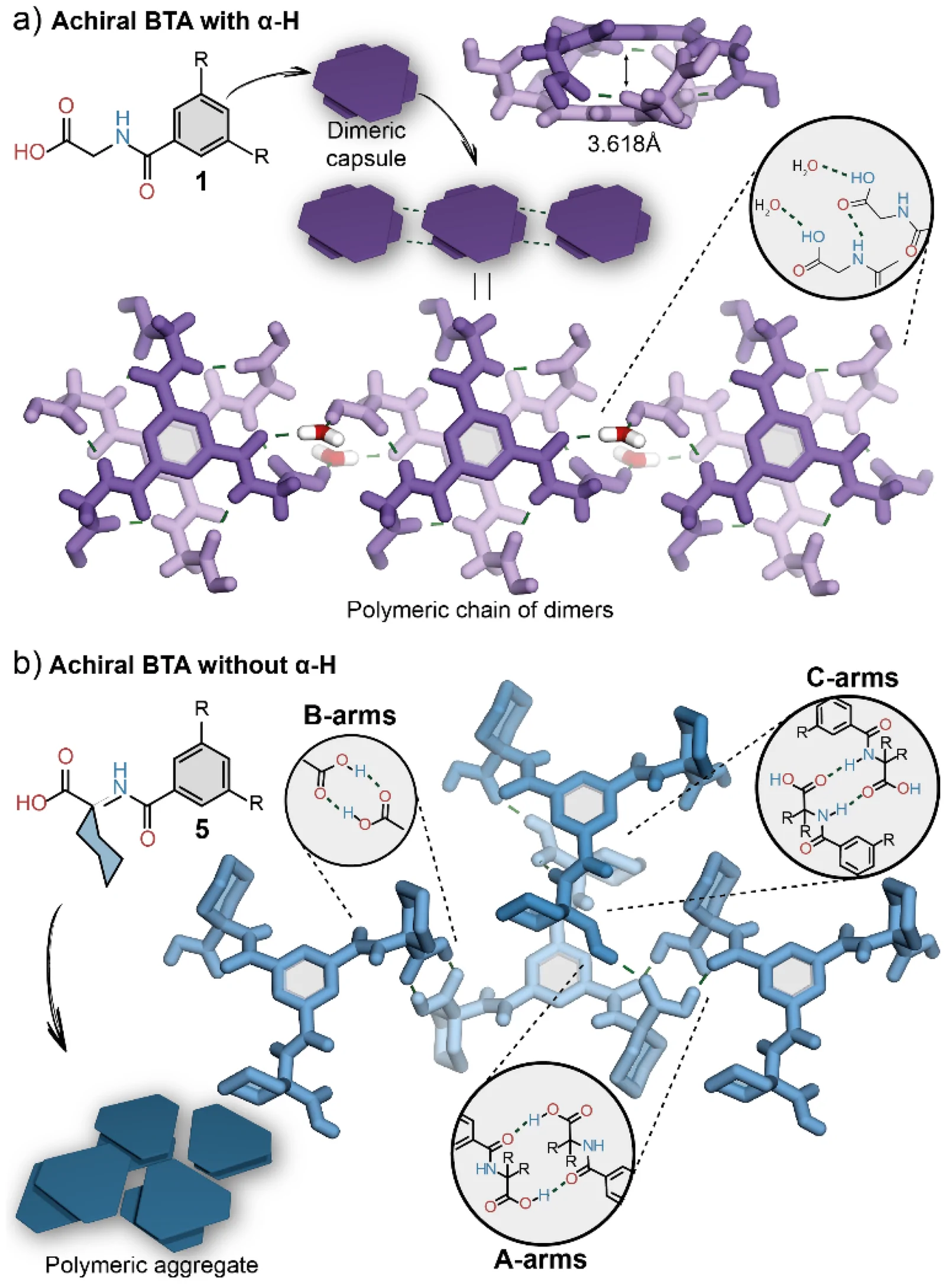
(a) Dimeric assembly of 1 stabilized by six intermolecular hydrogen bonds. Side view showing the centroid⋯centroid separation between the aromatic rings. Fragment of crystal structure of 1, illustrating the supramolecular polymer formed by centrosymmetric, spiral-type [6–6/0] dimers linked to water molecules *via* O–H⋯O hydrogen bonds. C–H⋯O interactions were omitted for clarity. (b) Representation of the non-covalent interactions between equivalent arms of 5 in the crystal polymeric structure, stabilized by intermolecular hydrogen bonding involving carboxylic acid and amide functional groups. TCE and TFA molecules, as well as C–H aromatic and aliphatic interactions, were omitted for clarity.

Compound 5, a modified analogue of 1, in which the introduction of a bulky cyclohexyl group at the α-position increases steric hindrance, eliminates the α-hydrogen, and at the same time preserves the molecule's achirality. It crystallizes as a 5 × 2TFA × TCE solvate and displays a markedly different structural organization. Introduction of the cyclohexyl group removes the α-H and introduces significant steric hindrance, leading to loss of internal symmetry and differentiation of the amide arms, with one arm oriented opposite to the other two. The molecular conformation is further modulated by TFA molecules, which form N–H(amide)⋯O=C(TFA) and O–H(TFA)⋯O=C(amide) hydrogen bonds with the amide groups. This altered hydrogen-bonding environment and steric demand redirect the orientation of functional groups, favouring the formation of extended supramolecular ribbons rather than discrete dimers. Within these ribbons, the A-labelled arm forms a non-covalent connection with the A-arm of a neighbouring molecule *via* O–H(acid)⋯O=C(amide) hydrogen bonds. Simultaneously, the C–arm engages in N–H(amide)⋯O=C(acid) hydrogen bonding with the corresponding arm of another molecule (Table S127 in SI). This assembly is further stabilized by O–H(acid)⋯O=C(acid) hydrogen bonds between the carboxyl groups of the A and C-arms (Fig. 3b). Interactions of the carboxyl groups from the B-arms, forming carboxylic acid dimers, organize the ribbons into a two-dimensional supramolecular polymer. A full crystallographic description is provided in the SI, in the section Crystallographic data. Taken together, the solid-state structures of 1 and 5 highlight how substitution at the α-position profoundly alters hydrogen-bonding directionality and packing preferences, providing a structural basis for the contrasting self-assembly behaviours observed in solution. Furthermore, these observations are fully consistent with the solid-state FT-IR data (Fig. S93 in SI), which clearly indicate a less uniform hydrogen-bonding network and an increased degree of structural heterogeneity in the α-Me derivatives compared to their α-H-containing analogues. The broader and poorly defined carbonyl stretching bands observed for these compounds further support the reduced structural order and increased conformational variability,^45^ as observed in solution. A detailed discussion of the solid-state FT-IR analysis was provided in the SI (see section FT-IR analysis in the solid state).

### Self-assembly in solution

To assess whether chirality or substitution at the α-position of amino acid-derived BTA derivatives influences supramolecular capsule formation, we focused on studying their self-assembly behaviour in solution. Specifically, we aimed to determine whether (i) the removal of point chirality in the side chains (components 1–6), and/or (ii) the replacement of the α-hydrogen atom with a sterically more demanding group (components 10 and 11; *vide infra*), would significantly affect the formation of hydrogen-bonded octameric capsules in non-competitive solvents.

**(a) Achiral BTAs**1–6: while the poor solubility of polar compound 1 in non-polar solvents was anticipated (*vide supra*), the incorporation of bulky, hydrophobic groups in compounds such as 5 and 6 was intended to improve compatibility with chlorinated media and thereby enable direct observation of their aggregation in solution. Contrary to expectations, none of the achiral components 1–6 showed adequate solubility in standard non-competitive solvents such as CHCl_3_, TCE or alkanes. Even compound 5, bearing three lipophilic cyclohexyl groups, remained insoluble under conditions typically used to promote supramolecular aggregation of BTA derivatives. We attribute this unexpected behaviour to two complementary effects, which may operate independently or synergistically: (i) an unfavourable distribution of polarity, which hampers effective solvation in chlorinated solvents, and (ii) the absence of a stereogenic centre, which leads to altered orientation of the peripheral amide arms, disrupting the preorganization required for capsule formation and instead favouring the growth of robust, polymeric aggregates. This hypothesis is supported by both computed electrostatic potential maps and solid-state characterization data: compounds 1–6 exhibit a difference in the electrostatic surface potential (ESP) between the inward and outward faces, therefore frustrating the solubility in chlorinated solvents (Fig. S132 in SI). Both X-ray diffraction and FT-IR analyses of compounds 1 and 5 revealed the presence of extended hydrogen-bonding networks, centrosymmetric dimers, and polymeric chains-architectures clearly distinct from the well-defined, *D*_2_-symmetric assemblies observed for their chiral analogues forming octameric capsules.^41^ Taken together, the uniform insolubility observed across this structurally diverse set, ranging from the minimally substituted 2 to the substantially bulkier and more lipophilic derivatives 5 and 6, indicates that this behaviour is a systematic characteristic of the series rather than a property of any individual derivative.

**(b) Chiral BTAs**7–11: due to these solubility limitations, the self-assembly behaviour in solution could be reliably studied only for selected chiral and α-substituted derivatives. As a benchmark for solution-state self-assembly, we selected component 9, which bears a phenylalanine residue linked to the BTA core. This synthon closely resembles the reference architecture used in our earlier studies, where homochiral amino acids such as cysteine promoted well-defined octameric capsule formation.^41^ As expected, compound 9 displayed excellent solubility in TCE-*d*_2_ – a solvent non-competitive toward hydrogen bonding – and upon dissolution, underwent supramolecular aggregation into a highly ordered capsule structure. The ^1^H NMR spectrum (Fig. 5a) revealed the characteristic loss of *C*_3_ symmetry and emergence of three distinct sets of resonances, consistent with *D*_2_-symmetric octamer formation. DOSY NMR analysis (Fig. S69 in SI) confirmed the formation of a single, monodisperse aggregate with a solvodynamic diameter of 26.7 Å (*D* = 1.12 × 10^−10^ m^2^ s^−1^) matching that of previously characterized octamers. The formation of hydrogen bonds characteristic for the octameric assembly was also clearly observed in FT-IR analysis (Fig. S94 in SI). IR spectrum recorded in TCE revealed bands corresponding to: *ν*(C=O)^acid^ at 1726 cm^−1^ and *ν*(C=O)^amide^ at 1644 cm^−1^; – both bathochromically shifted in comparison to the spectrum recorded in a competitive solvent (THF), where the C=O donors are unable to form H-bonds. Having established compound 9 as a reliable, chiral reference system, we next sought to investigate whether subtle structural variations at the α-position would influence aggregation behaviour. To this end, we investigated compounds 7 and 8, which were designed to examine the effect of steric bulk positioned at different distances from the α-carbon (*vide supra*). Remarkably, both compounds displayed excellent solubility in TCE and other non-polar solvents, and both underwent clean, efficient self-assembly into octameric capsules, as evidenced by solution-state NMR and FT-IR analyses (see Fig. S63, S64, S109 and S110 in SI). These results suggest that, at least within the limits of our current design, the steric bulk at the α-position – provided an H atom is present – whether directly adjacent or slightly displaced from the BTA core, does not significantly interfere with the capsule formation process in solution. Instead, both 7 and 8 reinforce the notion that solubility and/or presence of an α-H are the principal factors governing self-assembly. Once this barrier is overcome, the system exhibits remarkable structural tolerance toward moderate steric modifications at the α-position.

Encouraged by the successful assembly of compounds 7–9, we next turned our attention to the role of α-substitution more directly. Both 7 and 8 retained a hydrogen atom at the α-position, and their ability to form well-defined octameric capsules suggested that steric bulk around the α-position is not a limiting factor – provided the α-position remained monosubstituted. To test whether this α-H played a critical structural role in capsule formation, we synthesized compound 10, a direct analogue of phenylalanine-derived component 9, in which the α-H is replaced by a methyl group. This seemingly subtle change had a profound effect on the physical properties of the molecule. While compound 9 exhibited excellent solubility in non-competitive solvents and reliably formed octameric assemblies, its α-methylated analogue 10 proved to be practically insoluble in all media suitable for probing supramolecular aggregation. Despite sharing nearly identical side chain composition and overall polarity with 9, compound 10 consistently failed to dissolve in chlorinated solvents such as CHCl_3_ or TCE. This dramatic loss of solubility was particularly striking and suggested that α-methyl substitution, beyond its steric implications, fundamentally alters the solvation dynamics and/or solid-state packing behaviour of the molecule.

In response to this challenge, we introduced a structural modification aimed at restoring solubility while retaining the α-methyl substitution motif. Specifically, we appended a long aliphatic C_8_ chain to the aromatic ring of the tyrosine scaffold, generating compound 11. The rationale behind this design was twofold: (i) the hydrophobic C_8_ tail was expected to improve solubility in non-polar media through enhanced dispersibility, and (ii) the tyrosine platform offered a synthetically accessible site for targeted functionalization for capsule formation. Indeed, compound 11 displayed markedly improved solubility in TCE and could be successfully studied in solution. However, its behaviour diverged significantly from that of its α-H-containing counterparts. Instead of the well-resolved, symmetry-broken ^1^H NMR patterns observed for compounds 7–9, the spectrum of 11 in TCE-*d*_2_ revealed four sets of highly broadened signals (Fig. 5b). The amide H^NH^ protons appeared as singlets, lacking the *J*^3^-coupling with H^α^ observed in *e.g.* compound 9 – an expected consequence of α-methyl substitution. Four singlets corresponding to H^NH^ and four H^Ar^ signals were observed, although two of the latter overlapped into a broadened resonance at approximately 8.5 ppm. Overall the spectrum suggests that 11 in solution is involved in a dynamic equilibrium between H-bonded capsule-type and unbound species, however the components of this mixture do not separate in DOSY NMR spectra, yielding instead a single diffusion coefficient of 9.85 × 10^−11^ m^2^ s^−1^, corresponding to a solvodynamic diameter of approximately 30.4 Å in good agreement with octameric species. The total diameter of the computational octameric model for 11 is 28.3 Å, in line with the experimental solvodynamic diameter. It should be noted the octameric models do not consider explicit solvents, therefore the gas phase models are isolated from solvation effect. In line with this, FT-IR spectrum of 11 in TCE revealed clear splitting of the *ν*(C=O)^acid^*ν*(C=O)^amide^ as well as *ν*(N–H)^amide^ bands assigned to the presence of H-bonded and free species, when compared to the spectrum in THF (Fig. S94b in SI). To better understand the equilibrium observed between the two forms in solution, variable-temperature (VT) NMR spectra of 11 were performed. Results revealed that the equilibrium remained unchanged within the range of −30 to 70 °C. Only a sharpening of the signals was observed upon heating, which might be attributed to the changes in the overall dynamics of the assembly (Fig. S78 and S79 in SI). To further examine this equilibrium, compound 11 was titrated with triethylamine (Et_3_N) (Fig. S80 and S81 in SI). Upon addition of 1.0–3.0 equivalents of base per BTA molecule, the signals corresponding to the octameric capsule progressively decreased in intensity and fully disappeared at approximately 3.0 equivalents, giving rise to a single, significantly broadened set of resonances.

Increasing the amount of base to 6.0–9.0 equiv. resulted in a distinct intermediate state, in which the acid–base equilibrium caused even more pronounced broadening of both amide and aromatic proton signals. Upon addition of a large excess of amine (5.0–10.0 equiv.) the equilibrium has shifted fully towards the salt form, leading to a noticeable sharpening of the resonances. The final spectrum of 11 in its salt form displayed aromatic signals at *δ* ≈ 8.21 ppm, identical to those observed for the species coexisting in equilibrium with the octameric assembly. These results unambiguously confirm that the hydrogen-bonded capsule exists in dynamic equilibrium with unbound species in solution.

### Density functional theory calculations

Comparing the electrostatic potential (ESP) maps of octameric computational models of the simplest achiral BTA (1), chiral BTA with α-H (9), and chiral BTA without an α-H (11) reveals marked differences in the electrostatic environment around the window portals (Fig. 4). The octameric assembly of 9 exhibits noticeable regions of positive electrostatic potential surrounding window portals (isocontours of up to +80 kJ mol^−1^), generating a favourable electrostatic environment anticipated to contribute to overall capsule stabilization. A similar, albeit less pronounced, electropositive region is also present in the octameric model of 11 that lacks α-hydrogen atoms compared to 9. This observation is fully consistent with the experimental results, which indicate that 11 is capable of forming an octameric capsule, albeit one that remains in equilibrium with monomeric species in solution. By contrast, the hypothetical octamer derived from achiral building block 1 lacks the steric bulk provided by the chiral substituents present in 9 and 11, resulting in a larger window diameter (4.5 Å *versus* 4.1 Å for 9). Consequently, the electropositive potential around the window portals is diminished, and the stabilizing electrostatic environment observed for the chiral analogues is largely absent. These results suggest the possibility that the generation of a sufficiently electropositive environment around the capsule windows is a key factor governing the stability of these supramolecular assemblies. We should note that all the Density Functional Theory calculation derived ESP maps were calculated in the gas phase, and the electrostatic potentials would be dampened based on the presence of solvents and dynamical motion of the systems, however, we expect for these qualitative differences to remain.

**Fig. 4:**
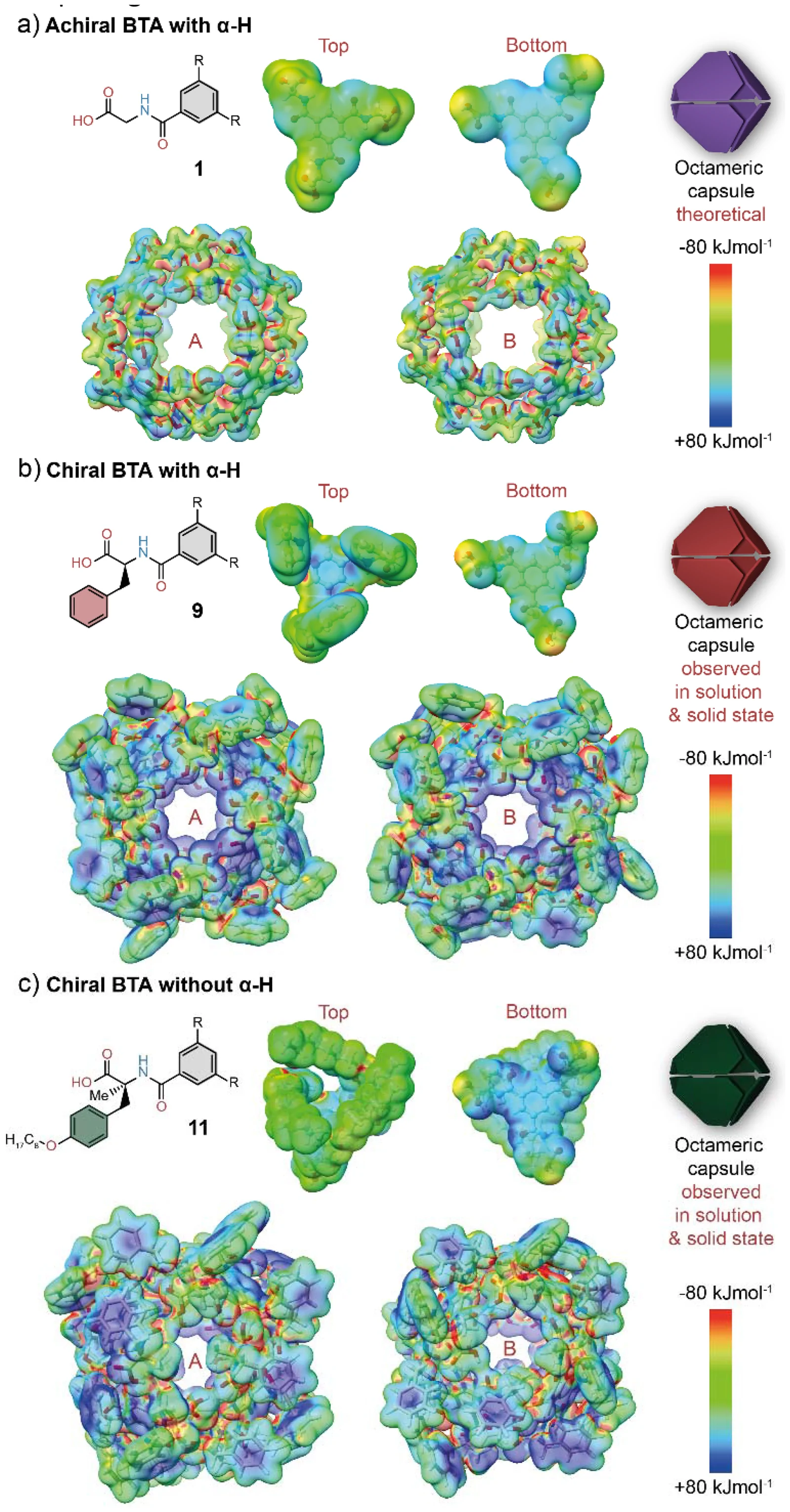
Electrostatic potential maps for (a) 1, (b) 9 and (c) 11, highlighting “Top” and “Bottom” parts of BTA cores and showing pore A, and pore B for octameric computational models. The electron energies have been cutoff beyond −0.03 and 0.03 a.u. (−80 to +80 kJ mol^−1^), with the electrostatic potential evaluated from a surface 1.4 Å outside the molecule surface for clarity. “Top view” corresponds to the R peripheral direction, whereas “Bottom” views correspond to carboxylate arms dentation direction.

### Fullerenes C_70_ & C_60_ encapsulation

Finally, the influence of α-methyl substitution on the host–guest properties of the obtained capsules was investigated. To evaluate their ability to act as molecular hosts, encapsulation studies were performed using C_60_ and C_70_ fullerenes as the model guest molecules^39,41,46–48^ (Fig. 5c, d and S76). Binding constants were calculated directly from ^13^C NMR spectra under equimolar host–guest conditions, where the peaks corresponding to the free guest [G] and [HG] are clearly resolved, thus allowing the equilibrium constants to be determined directly from the *K* = [HG]/([H] × [G]) equation.^41^

**Fig. 5:**
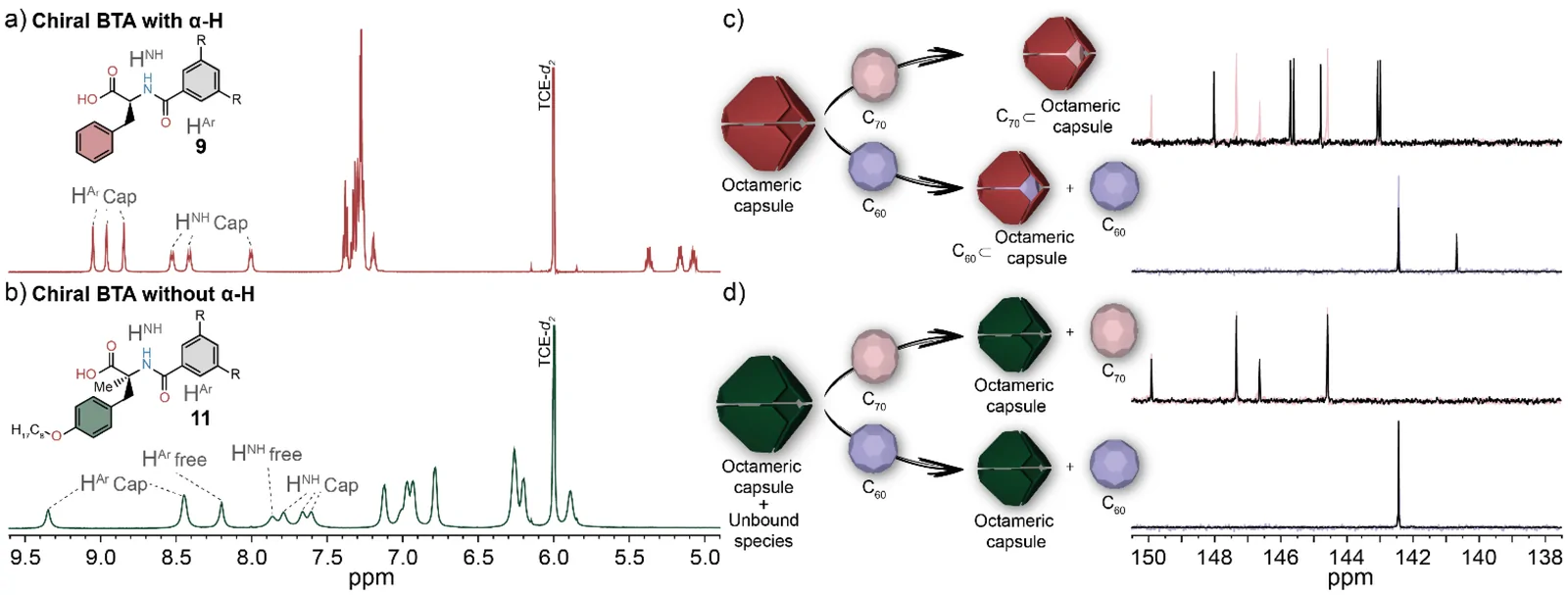
(a) ^1^H NMR spectrum of 9 in TCE-*d*_2_. (b) ^1^H NMR spectrum of 11 in TCE-*d*_2_. (c) Superimposed ^13^C NMR spectra in TCE-*d*_2_ top: mixture of 9 with C_70_ fullerene (black) and free C_70_ fullerene (pink), bottom: mixture of 9 with C_60_ fullerene (black) and free C_60_ fullerene (light purple). (d) Superimposed ^13^C NMR spectra in TCE-*d*_2_ top: mixture of 11 with C_70_ fullerene (black) and free C_70_ fullerene (pink). Complete overlap of the spectra are observed. Bottom: mixture of 11 with C_60_ fullerene (black) and free C_60_ fullerene (light purple). Complete overlap of the spectra are observed.

Interestingly, in this experiment 9 (α-H) exhibited an apparently quantitative (*K* > 10 × 10^4^ M^−1^) encapsulation of C_70_ with concomitant high affinity towards C_60_ (*K* ≈ 6 × 10^2^ M^−1^). The observed values are significantly higher than those for the previously studied derivatives,^39,41^ yet the capsule retains the preference for the larger fullerene (Fig. S72 and S73 in SI).

In contrast, the corresponding α-methylated derivative 11 showed no evidence for the complex formation neither with C_70_ nor C_60_ (Fig. S74 and S75 in SI). This behaviour clearly distinguishes 11 from capsules bearing α-hydrogen atoms and demonstrates that substitution at the α-positions influences not only the dynamics of capsule formation but also fundamentally alters their host–guest properties. Apparently, substitution of the α-positions imposes minor restrictions also on the internal void, thus limiting the available cavity for the guest uptake. A similar effect was also observed for the previously reported amido-derivatives, where shrinking of the cavity size fully blocked the uptake of the bigger C_70_.^39^

Taken together, these observations underscore the critical role of the α-position in governing supramolecular self-assembly and in determining key physical properties, particularly solubility. Compound 10, though structurally similar to the well-behaved 9, is rendered practically processable due to poor solubility. While sterically demanding side chains can be accommodated – as demonstrated by compounds 7 and 8, full substitution at the α-carbons, and thus removal of the α-H, as in compounds 10 and 11, introduces significant structural and solubility challenges. Compound 11 with α-methyl, although soluble, forms capsules that are dynamic and structurally fragile, likely due to the need to accommodate bulky substituents within the confined space of the capsule's internal cavity. These findings point to the α-hydrogen as a key structural feature, not only because it minimizes steric congestion, but also because it appears to play an indirect role in preserving the conformational plasticity needed for robust capsule formation.

## Conclusions

This study provides a systematic dissection of how subtle molecular features at the α-position, the nature of the amino acid side chain, and chirality collectively govern the self-assembly behaviour, physicochemical and host–guest properties of amino acid-derived benzene-1,3,5-tricarboxamides (BTAs). Through comparative analysis of eleven rationally designed components, we identify the presence of an α-hydrogen atom as a key structural element enabling the conformational adaptability required for the formation of discrete, hydrogen-bonded octameric capsules. Achiral derivatives and α-methylated analogues lacking this α-hydrogen (compounds 1–6 and 10) exhibit markedly reduced solubility in nonpolar media, precluding the formation of well-defined capsular assemblies. In contrast, chiral derivatives retaining the α-H motif (7–9) consistently self-assemble into robust, *D*_2_-symmetric octameric capsules, even in the presence of substantial steric bulk proximal to the α-position. While solubility can be partially restored in α-methylated systems through peripheral side-chain engineering, as exemplified by compound 11, the resulting assemblies remain more dynamic and structurally fragile, underscoring the delicate balance between steric congestion, polarity, and conformational flexibility in hydrogen-bonded capsules. Moreover, these structural effects translate directly into their host–guest properties, profoundly influencing fullerene encapsulation. Taken together, these findings establish transferable design rules that link local molecular structure at the α-carbon to higher-order supramolecular assembly, stability, and dynamics.

More broadly, a practical advantage of building these assemblies from amino acids is that the α-position remains available as a design element for tuning stability and function, mirroring a strategy long exploited by biology. Here, this position influences assembly through monomer solubility and steric accessibility of the capsule's internal cavity, rather than through the backbone-conformational control that governs polypeptide folding. Such insights provide a foundation for the rational design of next-generation supramolecular architectures with programmable assembly pathways, tuneable dynamics, and adaptive functionality. These findings further highlight the critical role of the α-H atom in governing both the structural integrity of the capsules and their ability to efficiently bind guest molecules.

## Conflicts of interest

There are no conflicts to declare.

## Supplementary Material

QO-OLF-D6QO01259K-s001

## Data Availability

All relevant data is contained within the article: the original contributions presented in the study are included in the article/supplementary information (SI), further inquiries can be directed to the corresponding author. Supplementary information is available. See DOI: https://doi.org/10.1039/d6qo01259k. CCDC 2421892 and 2422058 contain the supplementary crystallographic data for this paper.^49*a*,*b*^
