# Supplementary material for: Encoding stability and dynamics in hydrogen-bonded octameric capsules through amino acid α-position design
Source: Org Chem Front. 2026 Nov 6. Online ahead of print. doi: 10.1039/d6qo01259k (PMC13742164; doi:10.1039/d6qo01259k)
Supplement: Supplementary file 1 [file QO-OLF-D6QO01259K-s001.pdf]

Supplementary Information (SI) for Organic Chemistry Frontiers.
This journal is © the Partner Organisations

SUPPORTING INFORMATION

Encoding stability and dynamics in hydrogen-bonded octameric capsules through amino acid α-position design

Filip Perlitius, [a, b] Grzegorz Markiewicz, [a, b] Mateusz Gołdyn, [a, b] Gokay Avci, [c, d] Kim E. Jelfs, [c] and Artur R. Stefankiewicz [*a, b]

[a.] Center for Advanced Technologies Adam Mickiewicz University, Uniwersytetu Poznańskiego 10, 61-614 Poznań, Poland.

[b.] Faculty of Chemistry Adam Mickiewicz University, Uniwersytetu Poznańskiego 8, 61-614 Poznań, Poland.

[c.] Department of Chemistry, Molecular Sciences Research Hub, Imperial College London, 82 Wood Lane, London W12 0BZ, U.K.

[d.] Department of Chemistry, University College London, Christopher Ingold Building, 20 Gordon St, London WC1H 0AJ, U.K.

* E-mail: ars@amu.edu.pl

Content

1 General 3

2 Synthesis 5

2.1 Synthesis of tris-succinimidyl-1,3,5-benzenetricarboxylate (BTC-Osu) 5

2.2 Synthesis of benzene-1,3,5-tricarbonyl trichloride (BTC-Cl) 5

2.3 Synthesis of 1 6

2.4 Synthesis of 1 v2 7

2.5 Synthesis of 2 8

2.6 Synthesis of 2 v2 9

2.7 Synthesis of 3 10

2.8 Synthesis of 4 10

2.9 Synthesis of 5 11

2.10 Synthesis of 6 12

2.11 Synthesis of 7 13

2.12 Synthesis of 8 14

2.13 Synthesis of 9 15

2.14 Synthesis of 9 v2 16

2.15 Synthesis of 10 17

2.16 Synthesis of 11 17

3 NMR data 22

4 MS data 52

5 FT-IR data 58

5.1 FT-IR analysis in solid state 58

6 Crystallographic data 75

6.1 Single crystal XRD analysis of 1 and 5 75

7 Computational details 80

8 References 83

1 General

Materials and methods

Chemicals were purchased from commercial suppliers (mainly Sigma Aldrich, Merck and Fluorochem) and used as received. NMR solvents were purchased from Deutero GmbH (Germany) and used as received.

NMR Spectroscopy

NMR spectra were recorded on a Bruker Fourier 300 MHz, Bruker Avance III HD 400 MHz and Bruker Avance III HD 600 MHz spectrometers and referenced on solvent residual peaks.

DOSY Spectra were recorded using Bruker LED pulse sequence and then replotted with TopSpin software using DOSY transformation. The solvodynamic radii were calculated using standard Stokes-Einstein equation assuming that the nanoparticles are spherical. Viscosities of nondeuterated solvents at 298 K were used for this calculation, as follows:

- TCE □298 K = 1.46 mPa × s

ESI-MS

ESI-MS spectra were recorded on Bruker HD Impact and Bruker qTOF compact spectrometers in positive ion mode.

FT-IR Spectroscopy

The FT-IR spectra were recorded on Thermo Fisher Nicolet iS50 FT-IR spectrometer in the KBr pellet or airtight CaF_2-crystal cuvette, and then processed in Omnic spectra software. Spectra of the pure solvents were used for subtraction.

X-ray Crystallography

Reflection intensities for the **1·3H$_2$O** and **5·2TFA·TCE** monocrystals were collected on an Oxford Diffraction SuperNova diffractometer using graphite-monochromated Cu Kα radiation source (λ = 1.54178 Å). The Cryojet cooling device kept the crystals at a low temperature under a nitrogen stream during the experiment. The CrysAlis PRO software was used for data reduction and multi-scan absorption correction.[1] The crystal structures were solved by intrinsic phasing with ShelXT and refined by full-matrix least-squares against F^2 with ShelXL through the graphical interface Olex2.[2-4] Details on crystal data and structures refinement were listed in Tab. S117. Non-hydrogen atoms were refined anisotropically. The hydrogen atoms in **1·3H$_2$O** were derived from the difference Fourier maps and refined freely. The trifluoromethyl groups in both TFA molecules in **5·2TFA·TCE** are disordered over two positions with fixed occupancies of 0.50. Solvent molecule (TCE) is disordered over two positions with fixed occupancies of 0.85 and 0.15. The hydrogen atoms bound to carbon atoms in the solvent molecule were placed at idealized positions and refined using a riding model. The remaining hydrogen atoms were derived from the difference Fourier maps and refined freely. Some geometrical restraints (SADI, RIGU) and constraints (EADP) were applied.
The data have been deposited in the Cambridge Crystallographic Data Collection (CCDC), deposition numbers CCDC **2421892** and **2422058**. These data can be obtained free of charge *via*

www.ccdc.cam.ac.uk/data_request/cif, or by emailing data_request@ccdc.cam.ac.uk, or by contacting The Cambridge Crystallographic Data Centre, 12, Union Road, Cambridge CB2.

2 Synthesis

2.1 Synthesis of tris-succinimidyl-1,3,5-benzenetricarboxylate (BTC-Osu)

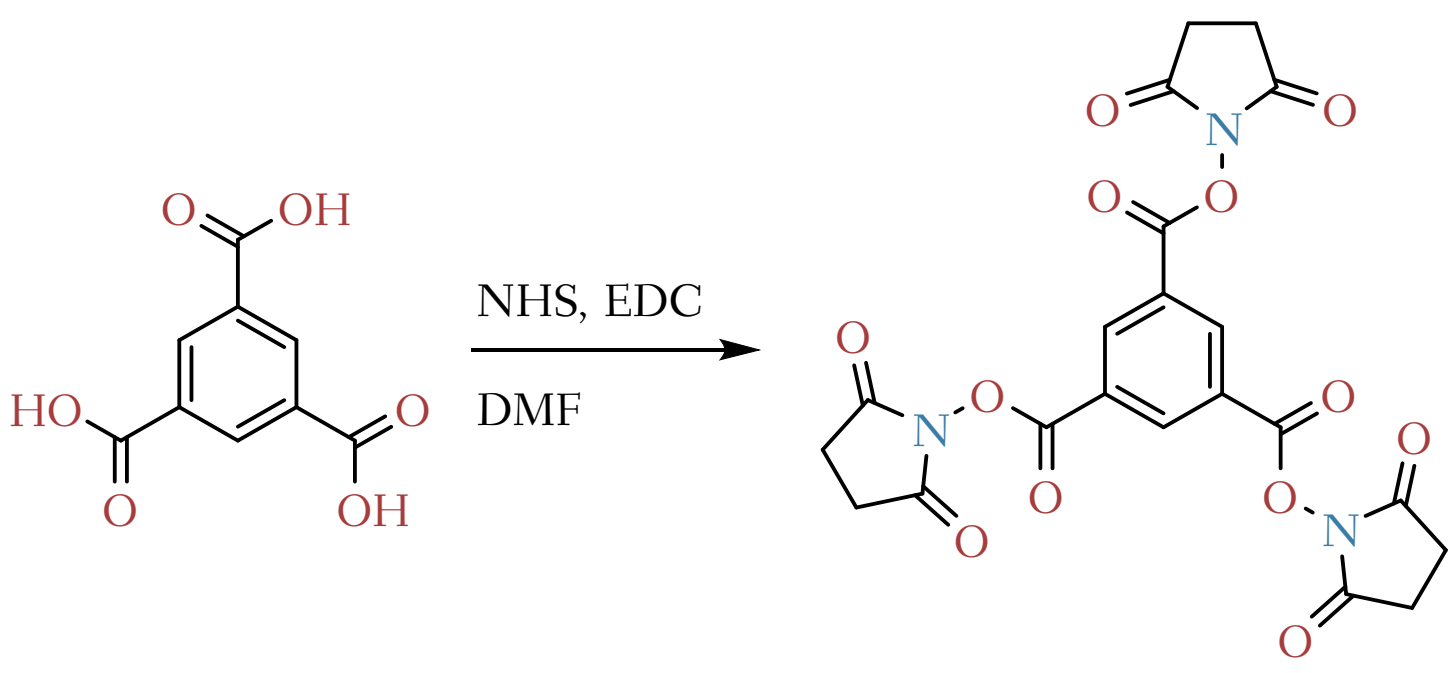

Figure S1. The synthesis of ***BTC-OSu.***

To a solution of benzene-1,3,5-tricarboxylic acid (BTC) (0.78 g, 3.70 mmol) in anhydrous DMF (20 mL), N-hydroxysuccinimide (2.55 g, 22.2 mmol) and 1-ethyl-3-(3-dimethylaminopropyl)-carbodiimide hydrochloride (4.26 g, 22.2 mmol) were added. The mixture was stirred at room temperature for 24 h. After solvent removal, the residue was dissolved in acetone (25 mL) and poured into 1 M HCl (200 mL). The white precipitate was filtered off, washed with H_2O (50 mL) and hot isopropanol (50 mL) and dried in vacuo. The pure product was obtained as a white solid in 70% yield.
Synthesis of N-succinimidyl ester of 1,3,5-benzenetricarboxylic acid was taken from literature.[5]

^{1}H NMR (300 MHz, DMSO-*d6*) δ 8,93 (s, 1H), 2,92 (s, 4H).

2.2 Synthesis of benzene-1,3,5-tricarbonyl trichloride (BTC-Cl)

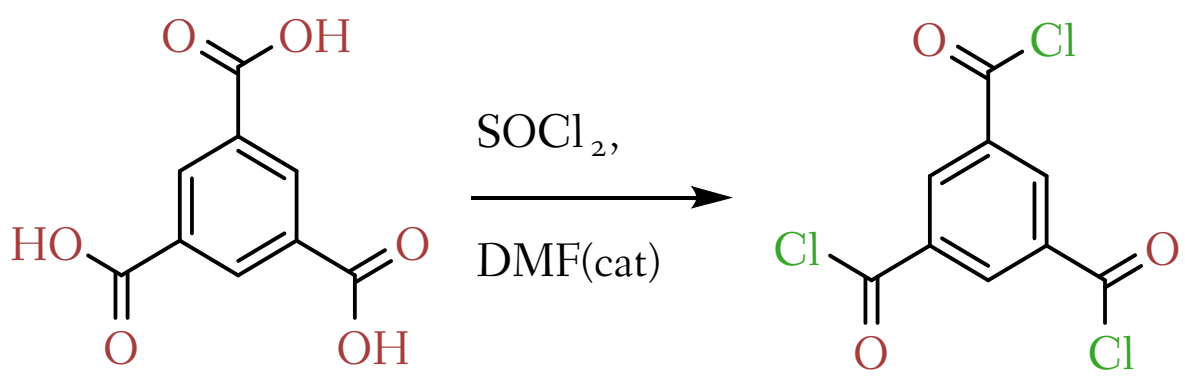

Figure S2. The synthesis of ***BTC-Cl.***

Benzene-1,3,5-tricarboxylic acid (BTC) (6.0 g, 28.7 mmol) was added to a flask along with SOCl₂ (12 mL) and a drop of DMF. The mixture was refluxed for 4 h, resulting in a clear solution. The residual SOCl₂ was removed under reduced pressure. The crude product was used directly without purification. Yield: 98%.

Synthesis of benzene-1,3,5-tricarbonyl trichloride was taken from literature.[6]

2.3 Synthesis of 1

Preparation of 1-*t*Bu

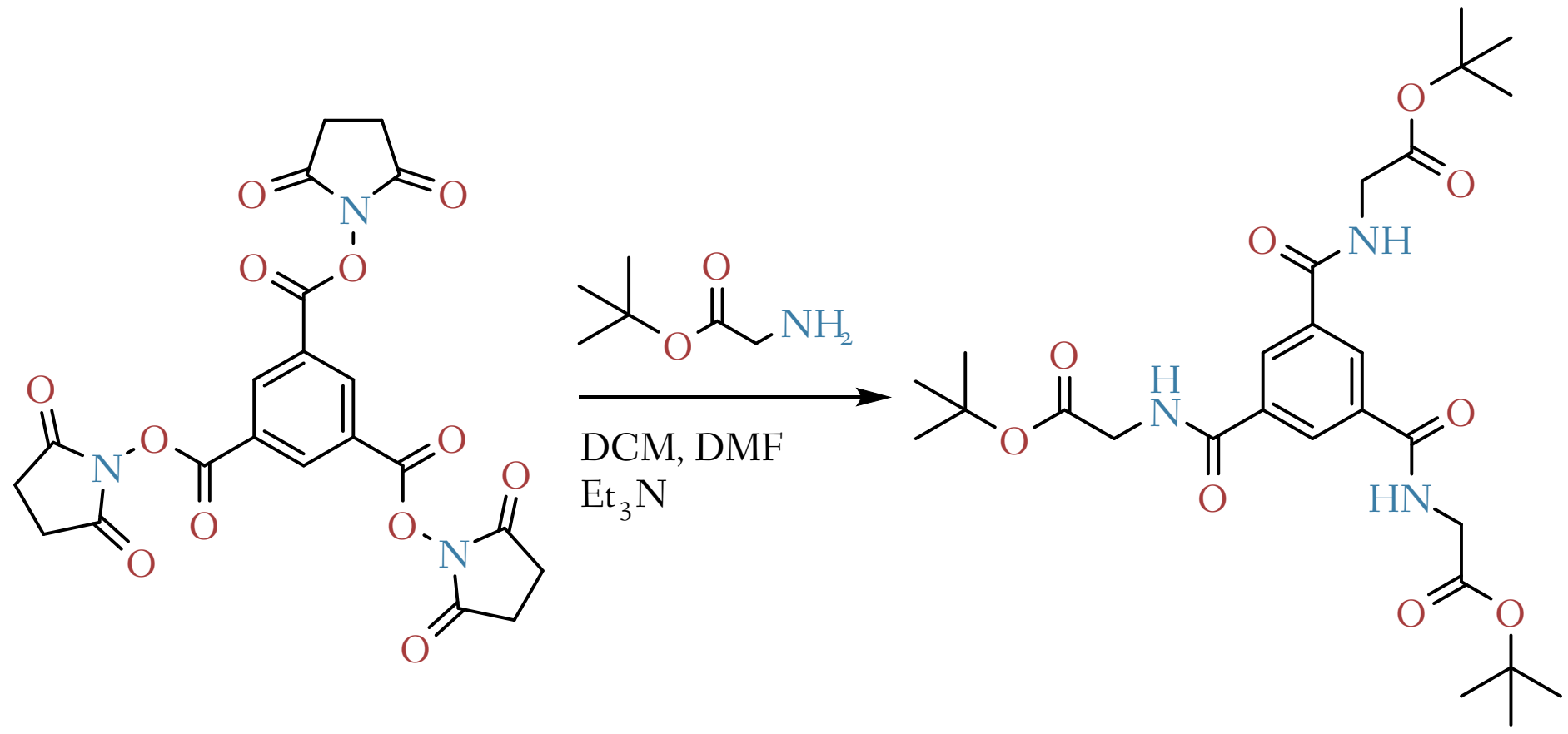

Figure S3. *The synthesis of 1-tBu.*

To a solution of the N-succinimidyl ester of 1,3,5-benzenetricarboxylic acid (0.501 g, 1 mmol) in anhydrous 3:1 DCM/DMF (16 mL) was added tert-butyl glycinate (0.787 g, 6 mmol) and triethylamine (0.837 mL, 6 mmol). The mixture was stirred for 24 h at room temperature under an argon atmosphere. After this time, DCM (25 mL) was added to the reaction mixture, which was then washed three times with a saturated aqueous solution of sodium bicarbonate ($NaHCO_3$) (3 × 20 mL), three times with a 5% aqueous solution $KHSO_4$ (3 × 20 mL), and once with brine (20 mL). The organic phase was dried over anhydrous sodium sulphate and evaporated under reduced pressure using a rotary evaporator, yielding an oily product. After purification by column chromatography using ethyl acetate/n-hexane (1:1) as the solvent, the pure product was obtained as a white powder. Yield: 46%.

^{1}H NMR (600 MHz, DMSO-d_6) δ 9.12 (t, 1H), 8.51 (s, 1H), 3.94 (d, *J* = 5.7 Hz, 2H), 1.43 (s, 9H).

^{13}C NMR (151 MHz, DMSO-d_6) δ 168.78, 165.67, 134.48, 128.87, 80.74, 41.99, 27.74.

Preparation of 1

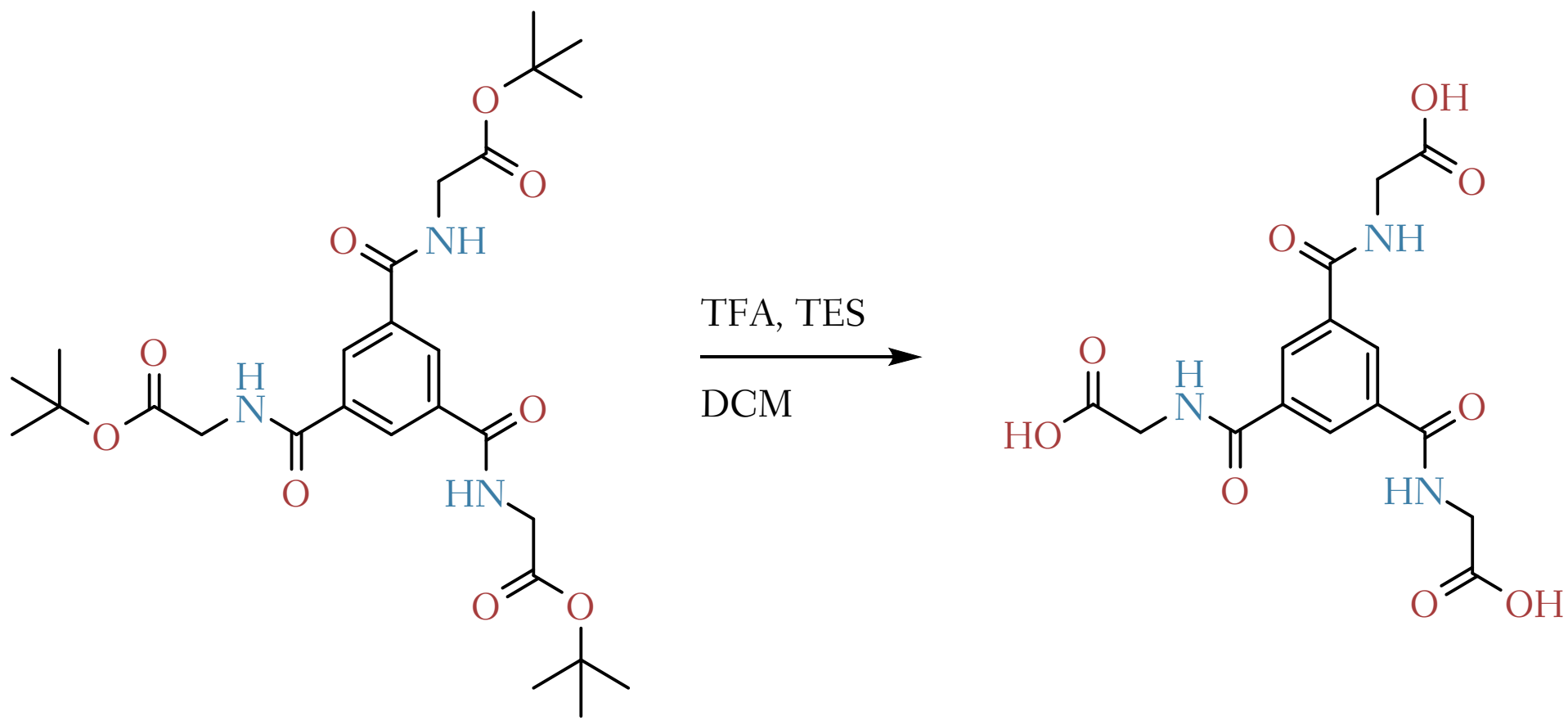

Figure S4. *The synthesis of 1.*

1-*t*Bu (0.100 g, 0.18 mmol) was dissolved in a mixture of trifluoroacetic acid and triethylsilane (9:1, 4.2 mL) with the addition of dichloromethane (2 mL). The mixture was stirred at room temperature for 24 h. After this time, the volatile substances were evaporated, followed by the addition of DCM, which was then evaporated

again to remove residual TFA. The product was purified by washing with ethyl acetate. After drying under reduced pressure, the pure product was obtained as a white powder. Yield: 95%.

^{1}H NMR (300 MHz, DMSO-d_6) δ 12.66 (s, 1H), 9.09 (t, *J* = 5.9 Hz, 1H), 8.49 (s, 1H), 3.97 (d, *J* = 5.7 Hz, 2H).

^{13}C NMR (75 MHz, DMSO-d_6) δ 171.12, 165.69, 134.52, 128.91, 41.35.

HRMS (ESI) Calcd for $C_{15}H_{15}N_3O_9Na$ $[M+Na]^+$: 404.0706 m/z, Found: $[M+Na]^+$ = 404.0708 m/z.

FT-IR (KBr) $ν_{max}$ [cm^{-1}] = 3469, 3392, 3360, 3082, 2986, 2948, 2640, 2575, 1722, 1659, 1595, 1550, 1418, 1362, 1312, 1262, 1251, 1197.

2.4 Synthesis of 1 v2

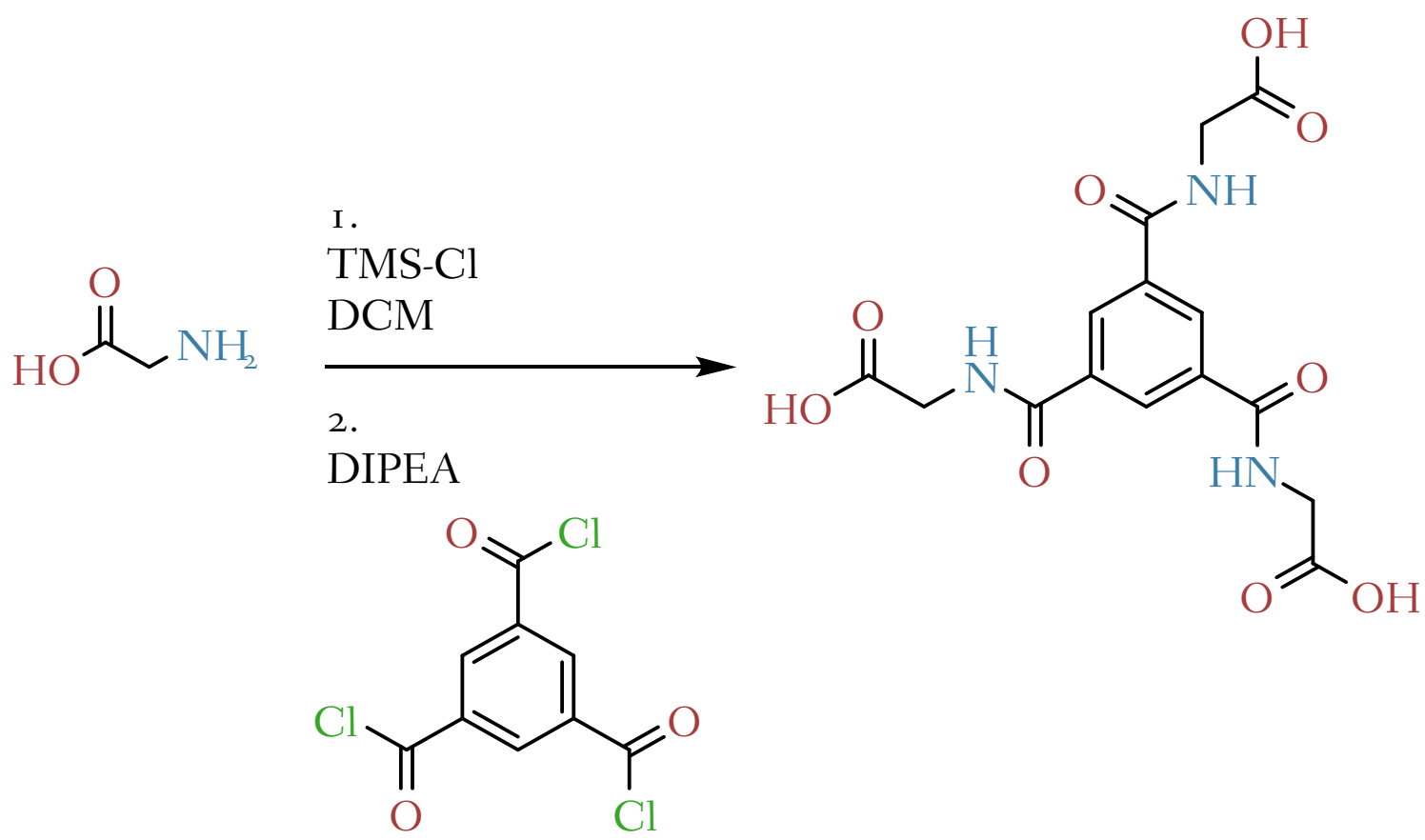

Figure S5. *The synthesis of 1.*

TMSCl (0.87 g, 8.0 mmol) was added to a suspension of glycine (0.3 g, 4.0 mmol) in dry CH_2Cl_2 (10 mL) and refluxed under an argon atmosphere for 8 h. The mixture was cooled to 0 °C, and DIEA (1.05 g, 8.0 mmol) and BTC-Cl (0.26 g, 1.0 mmol) were added. The reaction was allowed to warm to 25 °C and stirred for 20 h. The resulting mixture was concentrated in vacuo to provide a yellow solid which was then suspended in 1 M HCl (50 mL). The resulting solid was collected by filtration, washed with water and Et_2O and dried in vacuo. The pure product was obtained as a white solid in 68% yield.

^{1}H NMR (300 MHz, DMSO-d_6) δ 12.66 (s, 1H), 9.09 (t, *J* = 5.9 Hz, 1H), 8.49 (s, 1H), 3.97 (d, *J* = 5.7 Hz, 2H).

^{13}C NMR (75 MHz, DMSO-d_6) δ 171.12, 165.69, 134.52, 128.91, 41.35.

HRMS (ESI) Calcd for $C_{15}H_{15}N_3O_9Na$ $[M+Na]^+$: 404.0706 m/z, Found: 404.0708 m/z.

FT-IR (KBr) $ν_{max}$ [cm^{-1}] = 3469, 3392, 3360, 3082, 2986, 2948, 2640, 2575, 1722, 1659, 1595, 1550, 1418, 1362, 1312, 1262, 1251, 1197.

2.5 Synthesis of 2

Preparation of 2-*t*Bu

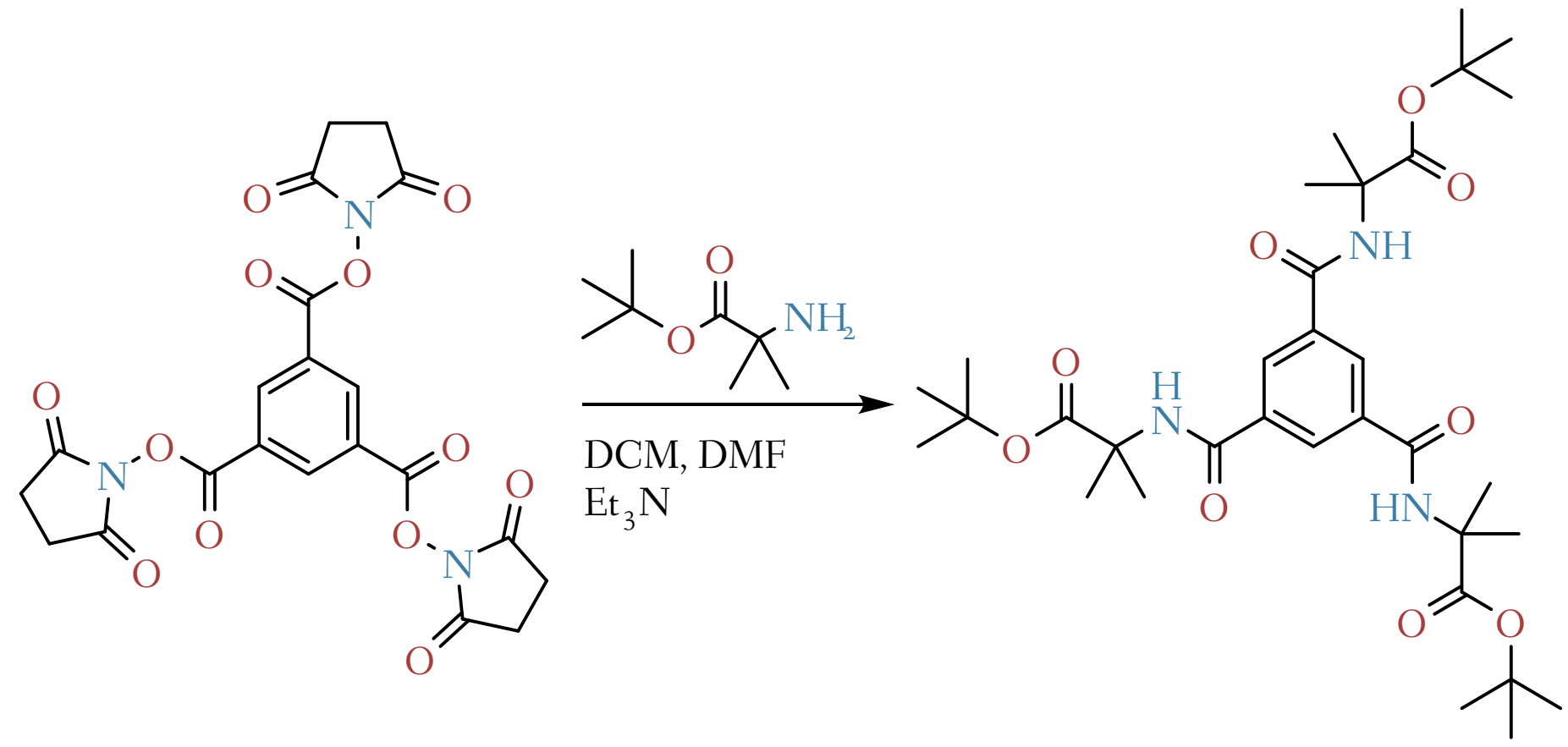

Figure S6.** The synthesis of **2-tBu.

To a solution of the *N*-succinimidyl ester of 1,3,5-benzenetricarboxylic acid (0.501 g, 1 mmol) in anhydrous 3:1 DCM/DMF (16 mL) was added tert-butyl ester of 2-aminoisobutyric acid (0.954 g, 6 mmol) and triethylamine (0.837 mL, 6 mmol). The mixture was stirred for 24 h at room temperature under an argon atmosphere. After this time, DCM (25 mL) was added to the reaction mixture, which was then washed three times with a saturated aqueous solution of sodium bicarbonate ($NaHCO_3$) (3 × 20 mL), three times with a 5% aqueous solution $KHSO_4$ (3 × 20 mL), and once with brine (20 mL). The organic phase was dried over anhydrous sodium sulphate and evaporated under reduced pressure using a rotary evaporator, yielding an oily product. After purification by washing with ethyl acetate, the pure product was obtained as a white powder. Yield: 48%.

^{1}H NMR (300 MHz, DMSO-d_6) δ 8.76 (s, 1H), 8.34 (s, 1H), 1.45 (s, 6H), 1.37 (s, 9H).

^{13}C NMR (75 MHz, DMSO-d_6) δ 172.76, 165.26, 134.73, 128.91, 79.53, 56.34, 27.56, 24.88.

Preparation of 2

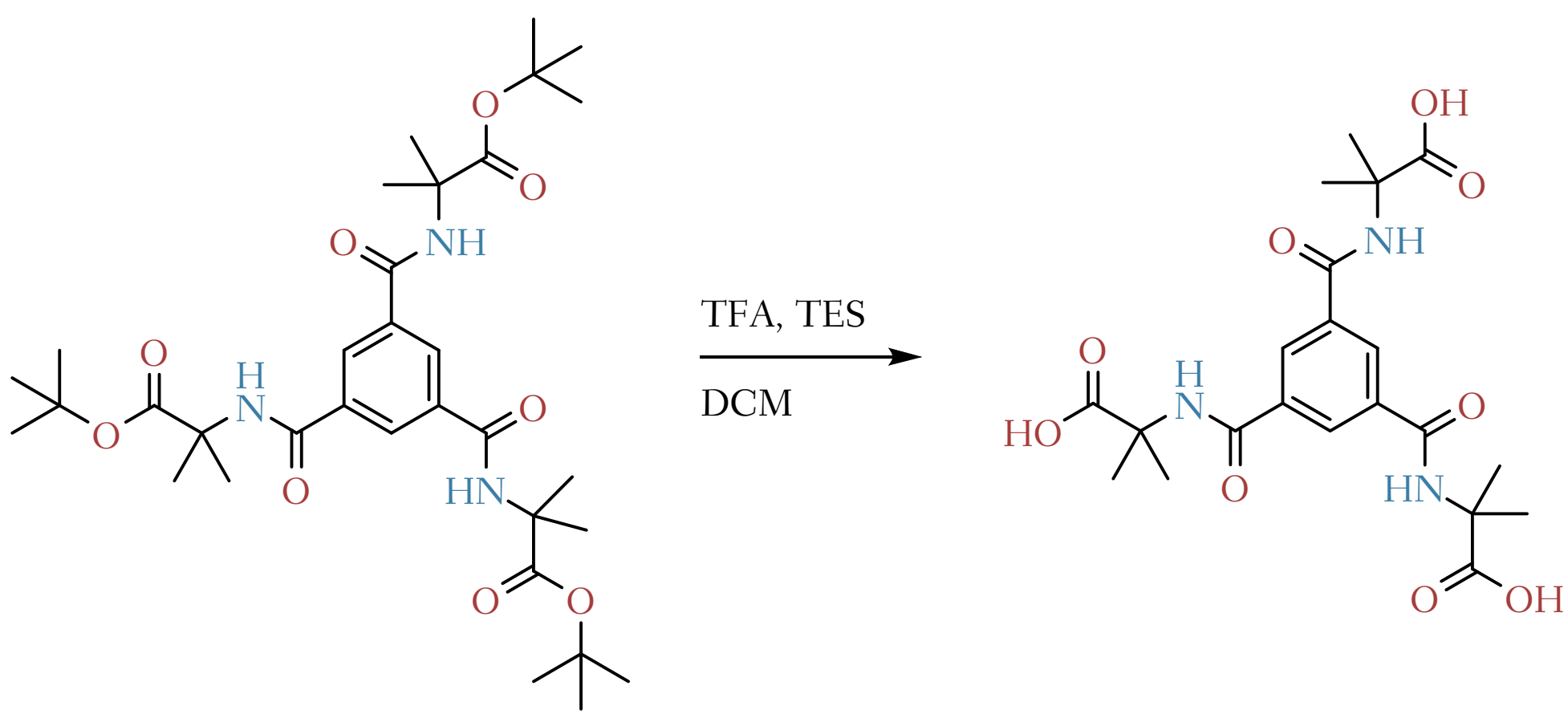

***Figure S7.** The synthesis of **1**.*

2-*t*Bu (0.158 g, 0.25 mmol) was dissolved in a mixture of trifluoroacetic acid and triethylsilane (9:1, 5.7 mL) with the addition of dichloromethane (2 mL). The mixture was stirred at room temperature for 24 h. After this time, the volatile substances were evaporated, followed by the addition of DCM, which was then evaporated again to remove residual TFA. The product was purified by washing with ethyl acetate. After drying under reduced pressure, the pure product was obtained as a white powder. Yield: 94%.

^{1}H NMR (300 MHz, DMSO-d_6) δ 12.09 (s, 1H), 8.78 (s, 1H), 8.37 (s, 1H), 1.48 (s, 6H).

^{13}C NMR (75 MHz, DMSO-d_6) δ 175.45, 165.25, 134.60, 129.22, 55.63, 24.98.

HRMS (ESI) Calcd for $C_{21}H_{27}N_3O_9Na$ $[M+Na]^+$: 488.1645 m/z, Found: $[M+Na]^+$ = 488.1645 m/z.

FT-IR (KBr) $ν_{max}$ [cm^{-1}] = 3342, 3227, 3060, 2994, 1741, 1717, 1640, 1557, 1538, 1473, 1309, 1218, 1174, 1153.

2.6 Synthesis of 2 v2

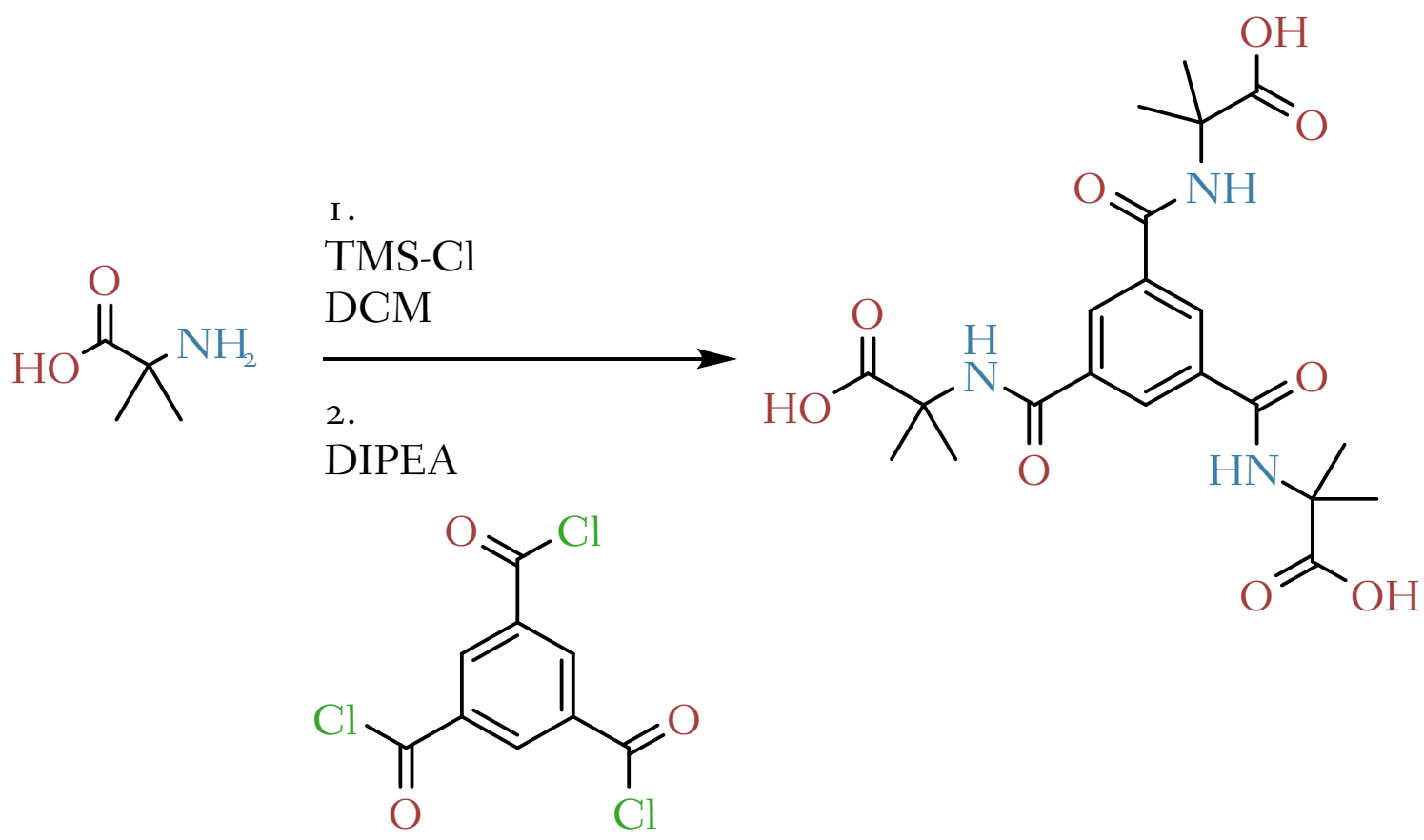

Figure S8. *The synthesis of* ***2.***

TMSCl (0.87 g, 8.0 mmol) was added to a suspension of 2-aminoisobutyric acid (0.42 g, 4.0 mmol) in dry CH_2Cl_2 (10 mL) and refluxed under an argon atmosphere for 8 h. The mixture was cooled to 0 °C, and DIEA (1.05 g, 8.0 mmol) and BTC-Cl (0.26 g, 1.0 mmol) were added. The reaction was allowed to warm to 25 °C and stirred for 20 h. The resulting mixture was concentrated in vacuo to provide a yellow solid which was then suspended in 1 M HCl (50 mL). The resulting solid was collected by filtration, washed with water and Et_2O and dried in vacuo. The pure product was obtained as a white solid in 60% yield.

^{1}H NMR (300 MHz, DMSO-d_6) δ 12.09 (s, 1H), 8.78 (s, 1H), 8.37 (s, 1H), 1.48 (s, 6H).

^{13}C NMR (75 MHz, DMSO-d_6) δ 175.45, 165.25, 134.60, 129.22, 55.63, 24.98.

HRMS (ESI) Calcd for $C_{21}H_{27}N_3O_9Na$ $[M+Na]^+$: 488.1645 m/z, Found: $[M+Na]^+$ = 488.1645 m/z.

FT-IR (KBr) $ν_{max}$ [cm^{-1}] = 3342, 3227, 3060, 2994, 1741, 1717, 1640, 1557, 1538, 1473, 1309, 1218, 1174, 1153.

2.7 Synthesis of 3

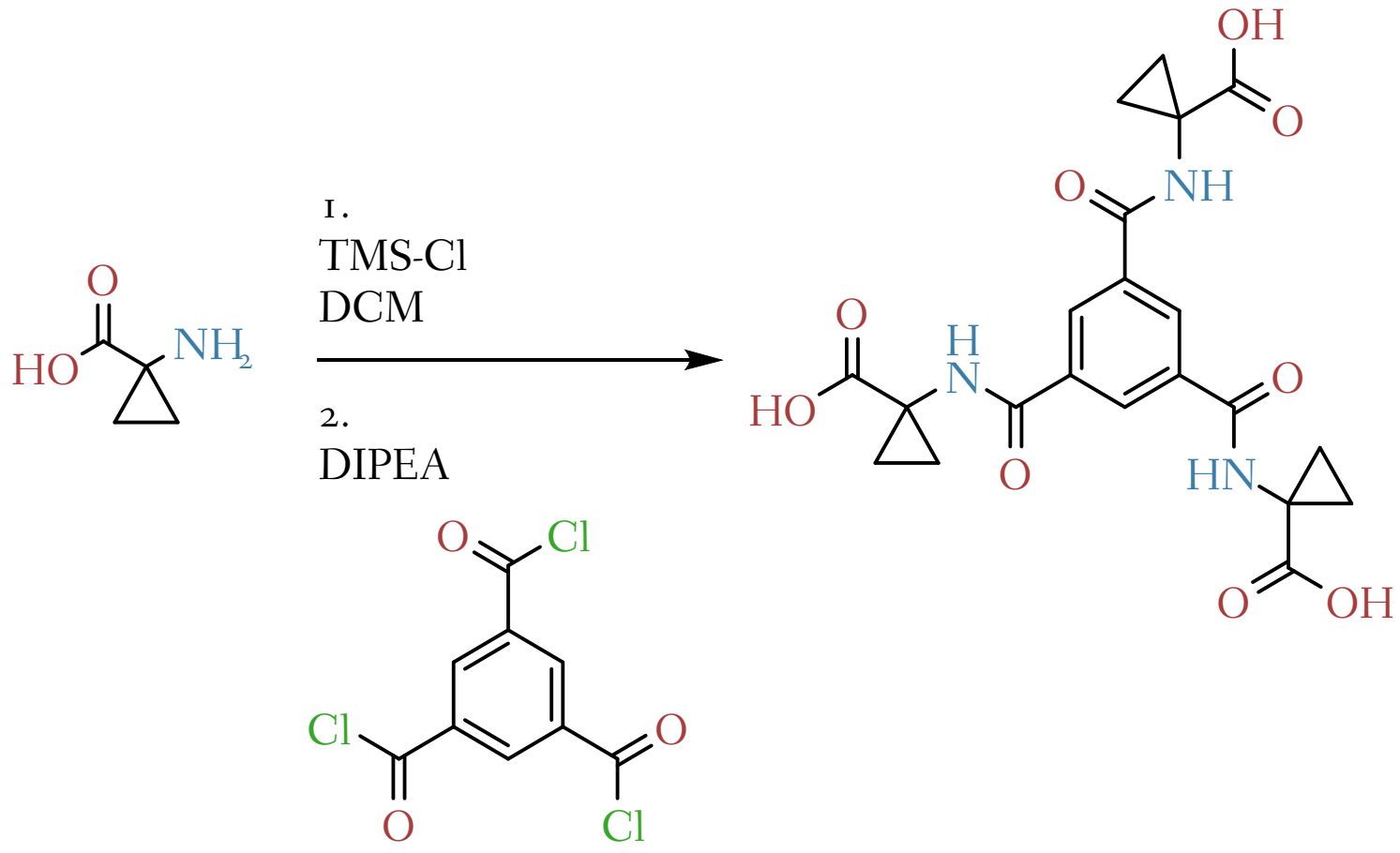

*Figure S9. The synthesis of **3**.*

TMSCl (0.87 g, 8.0 mmol) was added to a suspension of 1-aminocyclopropane-1-carboxylic acid (0. 404 g, 4.0 mmol) in dry CH_2Cl_2 (10 mL) and refluxed under an argon atmosphere for 8 h. The mixture was cooled to 0 °C, and DIEA (1.05 g, 8.0 mmol) and BTC-Cl (0.26 g, 1.0 mmol) were added. The reaction was allowed to warm to 25 °C and stirred for 20 h. The resulting mixture was concentrated in vacuo to provide a yellow solid which was then suspended in 1 M HCl (50 mL). The resulting solid was collected by filtration, washed with water and Et_2O and dried in vacuo. The pure product was obtained as a white solid in 67% yield.

^{1}H NMR (300 MHz, DMSO-d_6) δ 12.44 (s, 1H), 9.18 (s, 1H), 8.40 (s, 1H), 1.43 (q, J = 4.5 Hz, 2H), 1.13 (q, J = 4.5 Hz, 2H).

^{13}C NMR (75 MHz, DMSO-d_6) δ 173.73, 166.45, 134.67, 128.96, 33.21, 16.46.

HRMS (ESI) Calcd for $C_{21}H_{21}N_3O_9Na$ $[M+Na]^+$: 482.1175 m/z, Found: $[M+Na]^+$ = 482.1181 m/z.

FT-IR (KBr) $ν_{max}$ [cm^{-1}] = 3330, 3082, 3029, 2973, 2931, 1716, 1630, 1594, 1529, 1297, 1266, 1193, 916, 901, 775, 745, 592.

2.8 Synthesis of 4

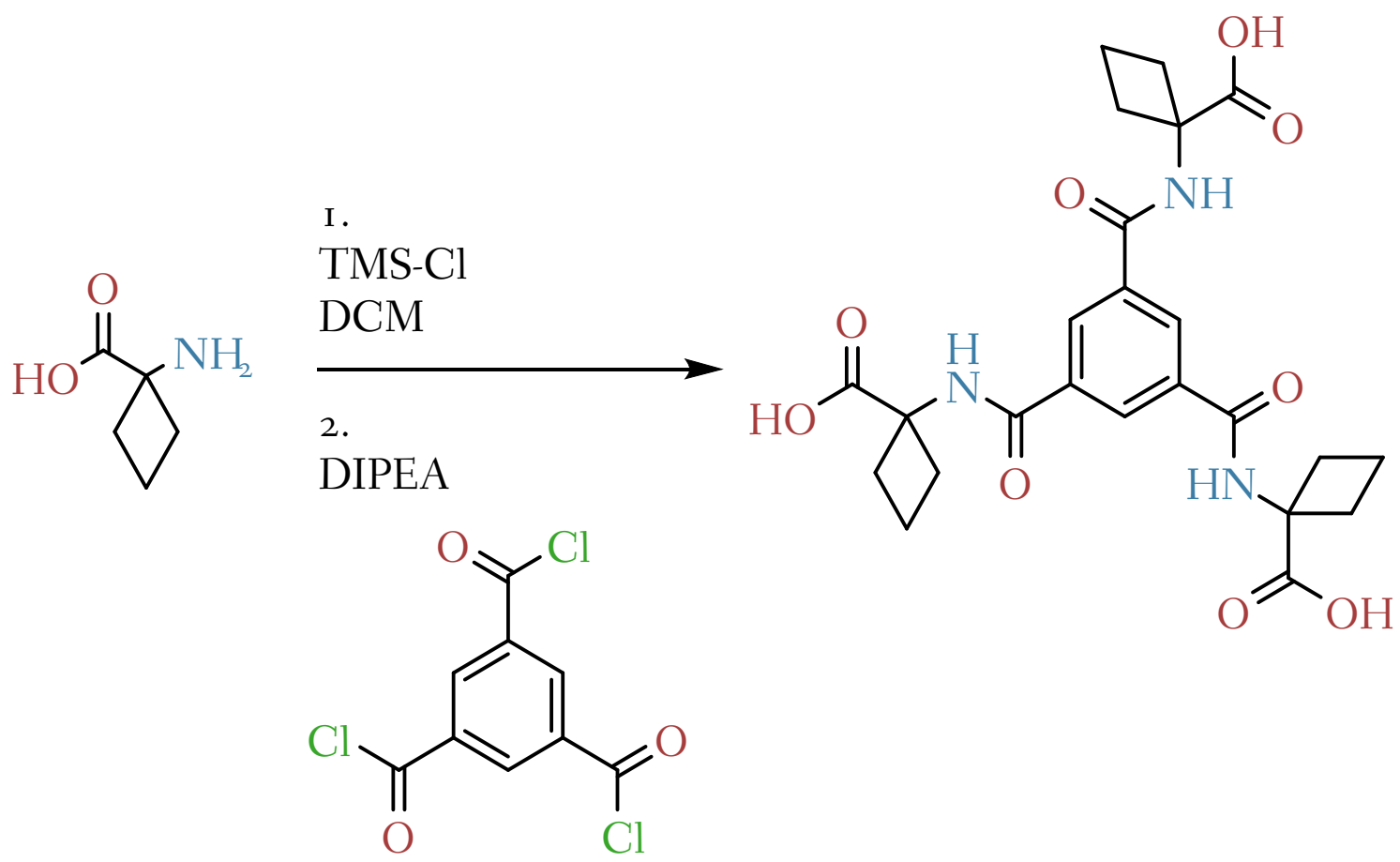

*Figure S10. The synthesis of **4**.*

TMSCl (0.87 g, 8.0 mmol) was added to a suspension of 1-aminocyclobutanecarboxylic acid (0. 461 g, 4.0 mmol) in dry CH_2Cl_2 (10 mL) and refluxed under an argon atmosphere for 8 h. The mixture was cooled to 0 °C, and DIEA (1.05 g, 8.0 mmol) and BTC-Cl (0.26 g, 1.0 mmol) were added. The reaction was allowed to warm to 25 °C and stirred for 20 h. The resulting mixture was concentrated in vacuo to provide a yellow solid which was then suspended in 1 M HCl (50 mL). The resulting solid was collected by filtration, washed with water and Et_2O and dried in vacuo. The pure product was obtained as a white solid in 63% yield.

^{1}H NMR ^{1}H NMR (300 MHz, DMSO-*d*$_6$) δ 12.27 (s, 1H), 9.26 (s, 1H), 8.47 (s, 1H), 2.59 (ddd, *J* = 12.2, 9.2, 6.7 Hz, 2H), 2.33 (dt, *J* = 12.1, 8.5 Hz, 2H), 1.96 (p, *J* = 7.4 Hz, 2H).

^{13}C NMR (75 MHz, DMSO-*d*$_6$) δ 174.59, 165.18, 134.44, 129.26, 58.09, 30.88, 15.35.

HRMS (ESI) Calcd for $C_{24}H_{27}N_3O_9Na$ $[M+Na]^+$: 524.1645 m/z, Found: $[M+Na]^+$ = 524.1644 m/z.

FT-IR (KBr) $ν_{max}$ [cm^{-1}] = 3383, 3230, 3048, 3002, 2981, 2958, 1738, 1715, 1642, 1531, 1521, 1336, 1291, 1234, 1215.

2.9 Synthesis of 5

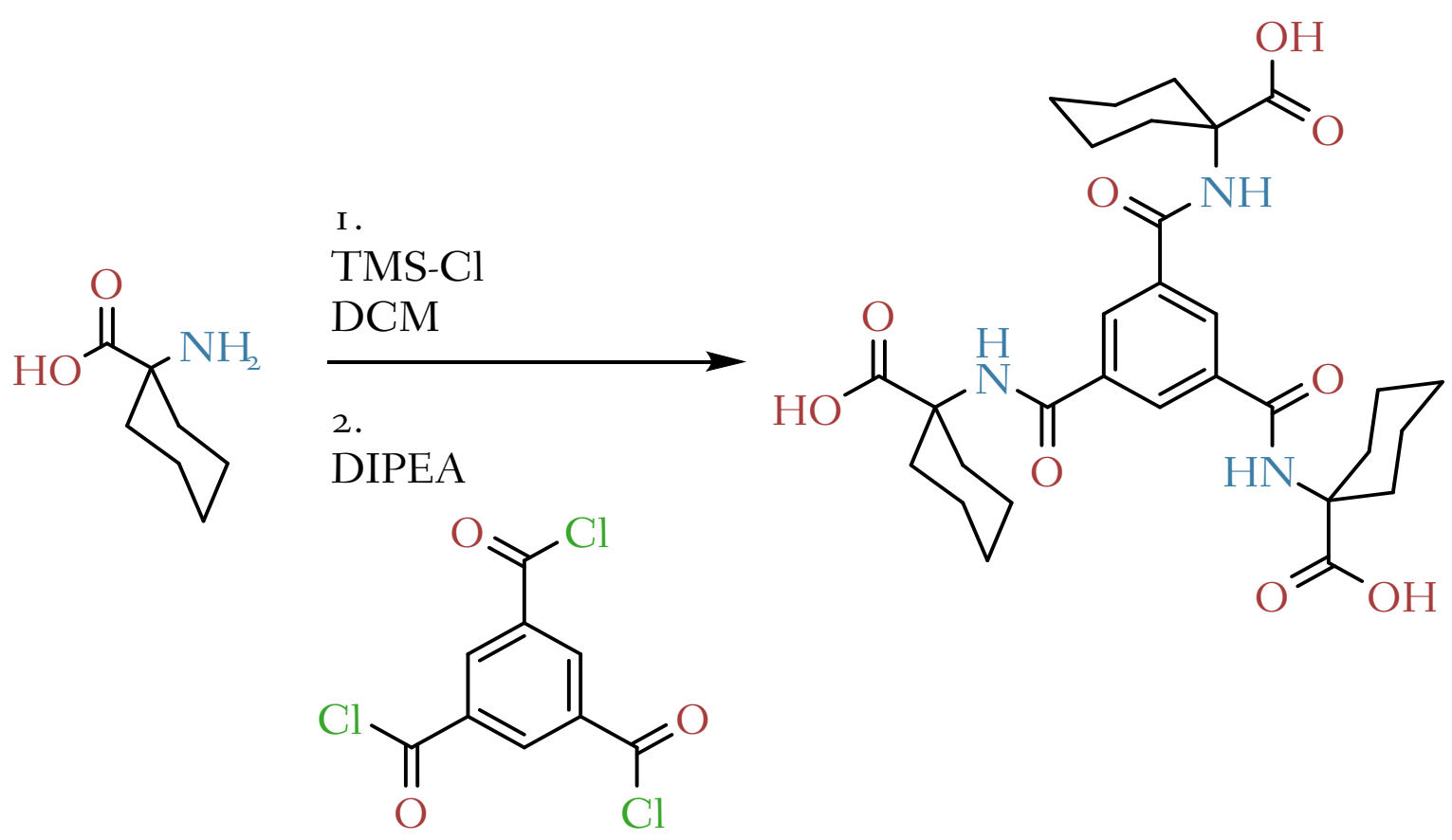

Figure S11. *The synthesis of **5**.*

TMSCl (0.87 g, 8.0 mmol) was added to a suspension of 1-aminocyclohexanecarboxylic acid (0.573 g, 4.0 mmol) in dry CH_2Cl_2 (10 mL) and refluxed under an argon atmosphere for 8 h. The mixture was cooled to 0 °C, and DIEA (1.05 g, 8.0 mmol) and BTC-Cl (0.26 g, 1.0 mmol) were added. The reaction was allowed to warm to 25 °C and stirred for 20 h. The resulting mixture was concentrated in vacuo to provide a yellow solid which was then suspended in 1 M HCl (50 mL). The resulting solid was collected by filtration, washed with water and Et_2O and dried in vacuo. The pure product was obtained as a white solid in 59% yield.

^{1}H NMR ^{1}H NMR (300 MHz, DMSO-*d*$_6$) δ 12.23 (s, 1H), 8.53 (s, 1H), 8.36 (s, 1H), 2.16 (d, *J* = 13.1 Hz, 2H), 1.78 (dt, *J* = 13.5, 6.8 Hz, 2H), 1.58 (s, 4H), 1.31 (q, *J* = 9.4, 8.7 Hz, 2H).

^{13}C NMR (75 MHz, DMSO-*d*$_6$) δ 175.55, 165.80, 134.73, 129.59, 58.62, 31.78, 25.15, 21.38.

HRMS (ESI) Calcd for $C_{30}H_{39}N_3O_9Na$ $[M+Na]^+$: 608.2584 m/z, Found: $[M+Na]^+$ = 608.2593 m/z.

FT-IR (KBr) ν$_{max}$ [cm^{-1}] = 3317, 3255, 3062, 2941, 2858, 1713, 1644, 1534, 1454, 1325, 1292, 1273, 1245, 1226, 1198, 1163, 665.

2.10 Synthesis of 6

Preparation of 6-OMe

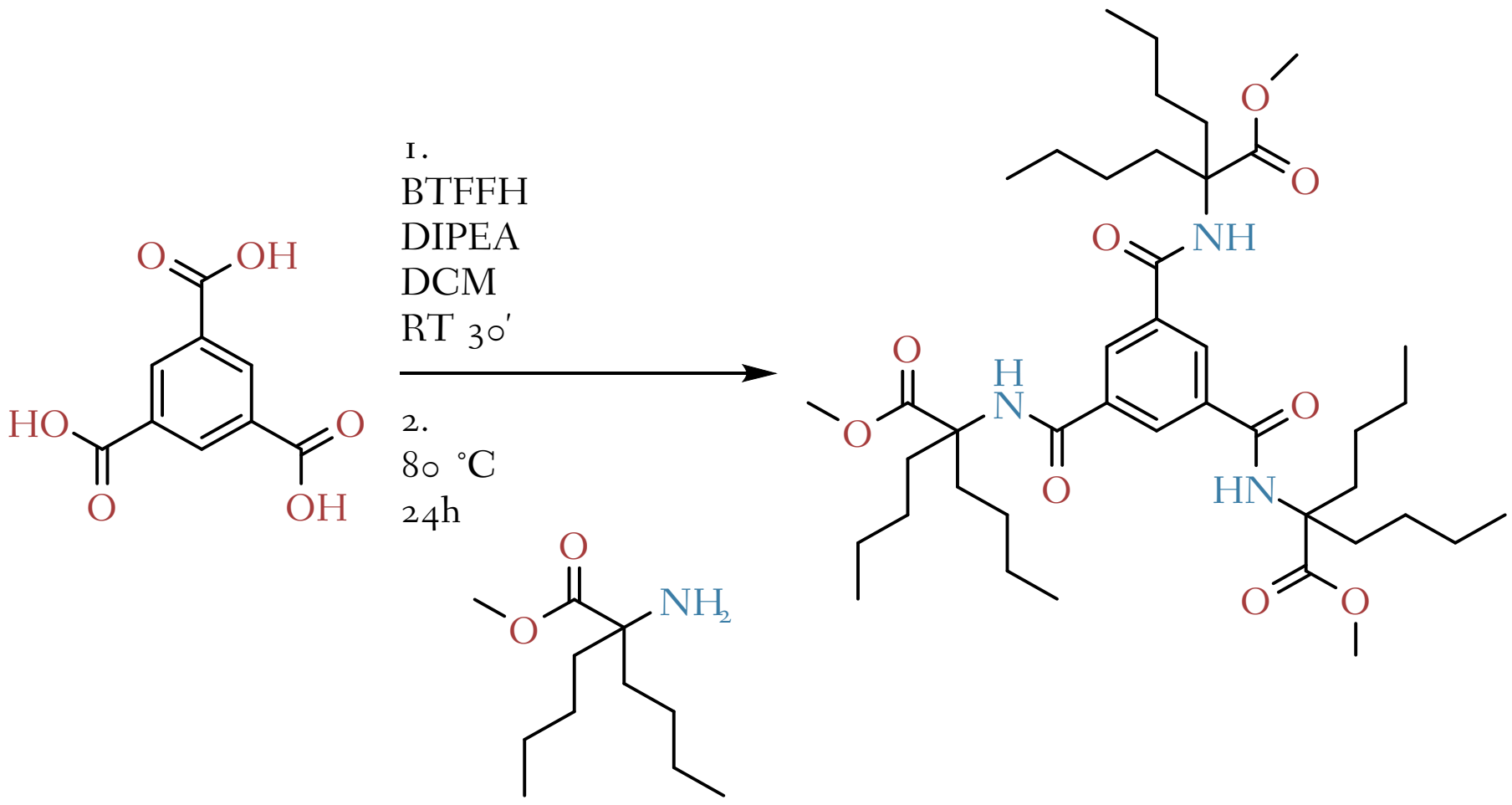

*Figure S12. The synthesis of **6-OMe**.*

To Schlenk flask benzene-1,3,5-tricarboxylic acid (0.42 g 0,2 mmol) and BTFFH (0.316 g 1 mmol) in dry CH_2Cl_2 (2 mL) was added under argon. DIPEA (0.320 mL 1.8 mmol) was subsequently added and the reaction was stirred under an argon atmosphere for 30 min. The 2-Amino-2-butylhexanoic acid methyl ester (0.201 g 1 mmol) was added and the flask was sealed and heated in an oil bath at 80 °C 24h under an argon atmosphere. The reaction mixture was cooled to room temperature and evaporated under reduced pressure. The residue was dissolved in EtOAc (10 mL) and washed with 1 M HCl (3 × 10 mL) and brine (10 mL), dried over Na_2SO_4 and concentrated. The crude product was purified by column chromatography 3/1 hexane/ethyl acetate. The pure product was obtained as a white solid in 88% yield.

^{1}H NMR (300 MHz, DMSO-*d*$_6$) δ 8.67 (s, 1H), 8.41 (s, 1H), 3.61 (s, 3H), 2.15 – 1.90 (m, 2H), 1.81 (dt, *J* = 13.6, 8.3 Hz, 2H), 1.44 – 1.05 (m, 8H), 0.86 (t, *J* = 7.1 Hz, 6H).

^{13}C NMR (75 MHz, DMSO-*d*$_6$) δ 173.44, 164.74, 134.22, 61.96, 51.77, 32.62, 25.17, 22.40, 13.91.

Preparation of 6

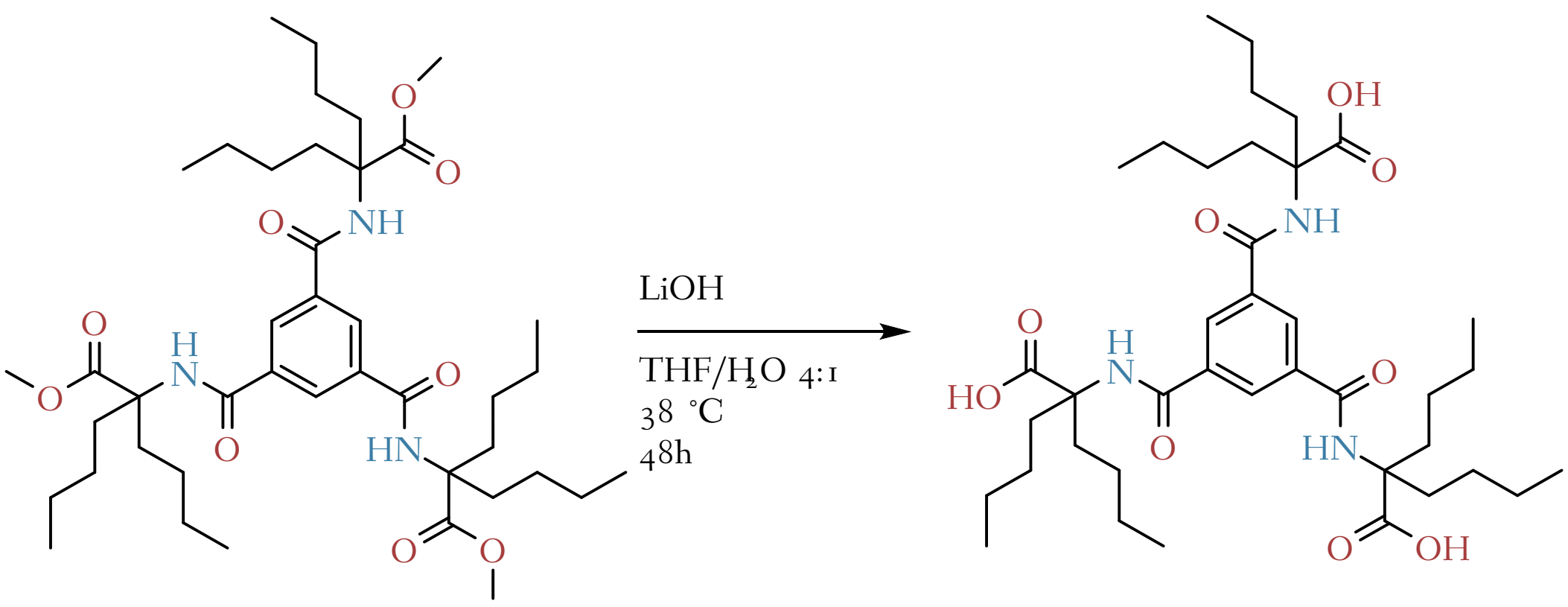

Figure S13. The synthesis of ***6.***

6-OMe (0.268 g 0.35) mol was dissolved in a 4:1 THF/H₂O mixture (15 mL), and (0.058 g 2.1 mmol) of LiOH was added. The reaction mixture was heated at 45 °C for 120 h. After this time, the mixture was evaporated, dissolved in water (10 mL), and acidified to pH 1 using 1 M HCl. The resulting precipitate was filtered, washed with water, and dried under reduced pressure. The pure product was obtained as a white solid in 92% yield.

^{1}H NMR (300 MHz, DMSO-d_6) δ 12.59 (s, 1H), 8.35 (s, 1H), 8.32 (s, 1H), 1.95 (t, *J* = 8.2 Hz, 4H), 1.23 (dddd, *J* = 24.1, 17.3, 10.9, 7.5 Hz, 8H), 0.85 (t, *J* = 7.0 Hz, 6H)

^{13}C NMR (75 MHz, DMSO-d_6) δ 174.65, 164.50, 134.79, 128.85, 62.37, 32.99, 25.50, 22.42, 13.96.

HRMS (ESI) Calcd for $C_{39}H_{63}N_3O_9Na$ $[M+Na]^+$: 740.4457 m/z, Found: $[M+Na]^+$ = 740.4451 m/z.

FT-IR (KBr) $ν_{max}$ [cm^{-1}] = 3378, 2960, 2933, 2873, 2863, 1720, 1651, 1648, 1518, 1467, 1457, 1284, 1253, 1215, 1159, 605.

2.11 Synthesis of 7

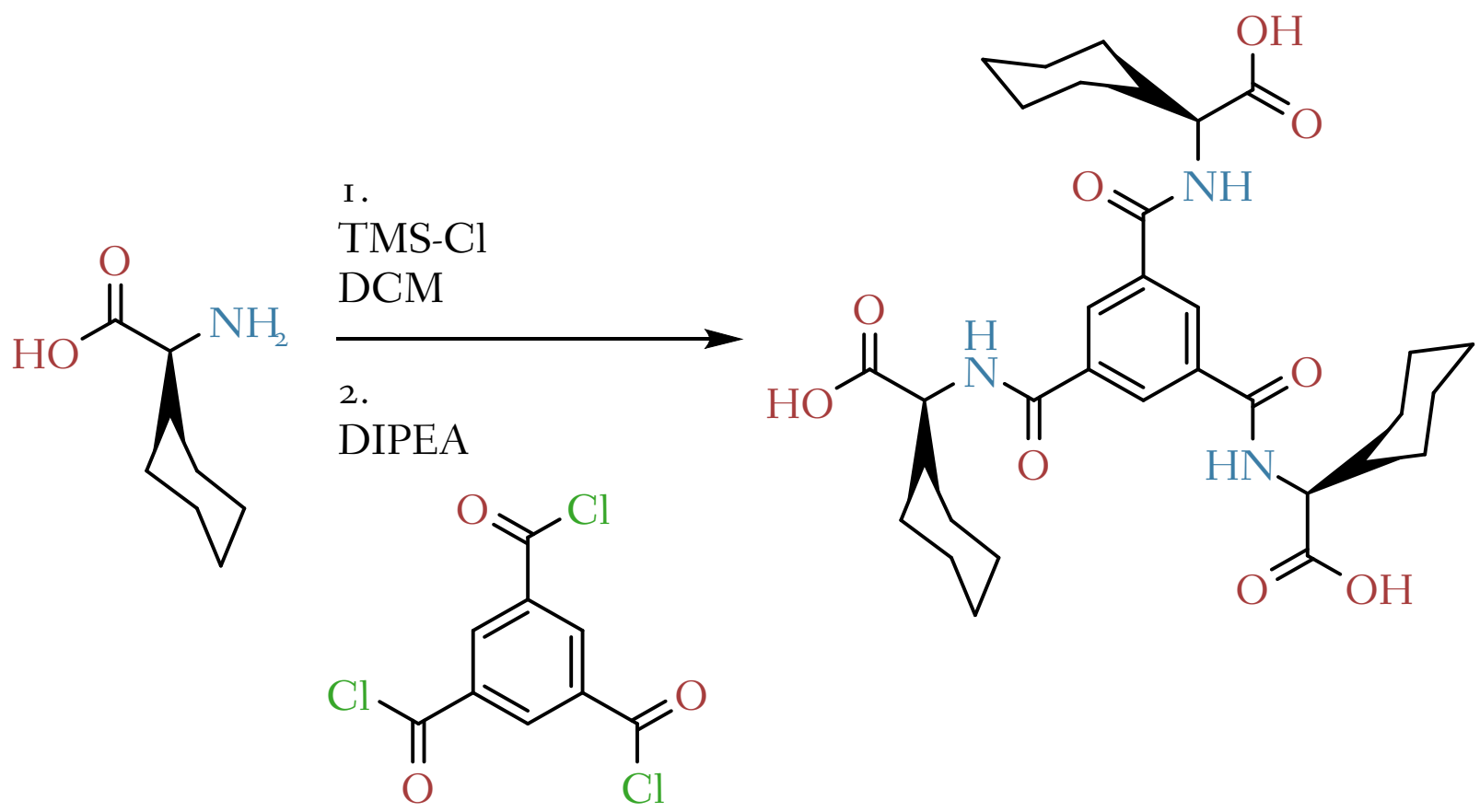

Figure S14. The synthesis of 7.

TMSCl (0.87 g, 8.0 mmol) was added to a suspension of 2-cyclohexyl- L-glycine (0.63 g, 4.0 mmol) in dry CH_2Cl_2 (10 mL) and refluxed under an argon atmosphere for 8 h. The mixture was cooled to 0 °C, and DIEA (1.05 g, 8.0 mmol) and BTC-Cl (0.26 g, 1.0 mmol) were added. The reaction was allowed to warm to 25 °C and stirred for 20 h. The resulting mixture was concentrated in vacuo to provide a yellow solid which was then suspended in 1 M HCl (50 mL). The resulting solid was collected by filtration, washed with water and Et_2O and dried in vacuo. The pure product was obtained as a white solid in 66% yield.

^{1}H NMR (300 MHz, DMSO-d_6) δ 12.65 (s, 1H), 8.79 (d, *J* = 8.1 Hz, 1H), 8.37 (s, 1H), 4.32 (t, *J* = 7.5 Hz, 1H), 1.87 (s, 1H), 1.67 (d, *J* = 26.6 Hz, 5H), 1.19 (dq, *J* = 23.1, 12.9, 9.4 Hz, 6H).

^{13}C NMR (75 MHz, DMSO-d_6) δ 172.96, 166.35, 134.46, 129.60, 57.95, 38.84, 29.38, 28.73, 25.68, 25.61.

HRMS (ESI) Calcd for $C_{33}H_{45}N_3O_9Na$ $[M+Na]^+$: 650.3048 m/z, Found: $[M+Na]^+$ = 650.3046 m/z.

FT-IR (KBr) ν$_{max}$ [cm^{-1}] = 3346, 3079, 2930, 2854, 1724, 1650, 1595, 1529, 1450, 1412, 1333, 1297, 1263, 1229, 1201, 674.

2.12 Synthesis of 8

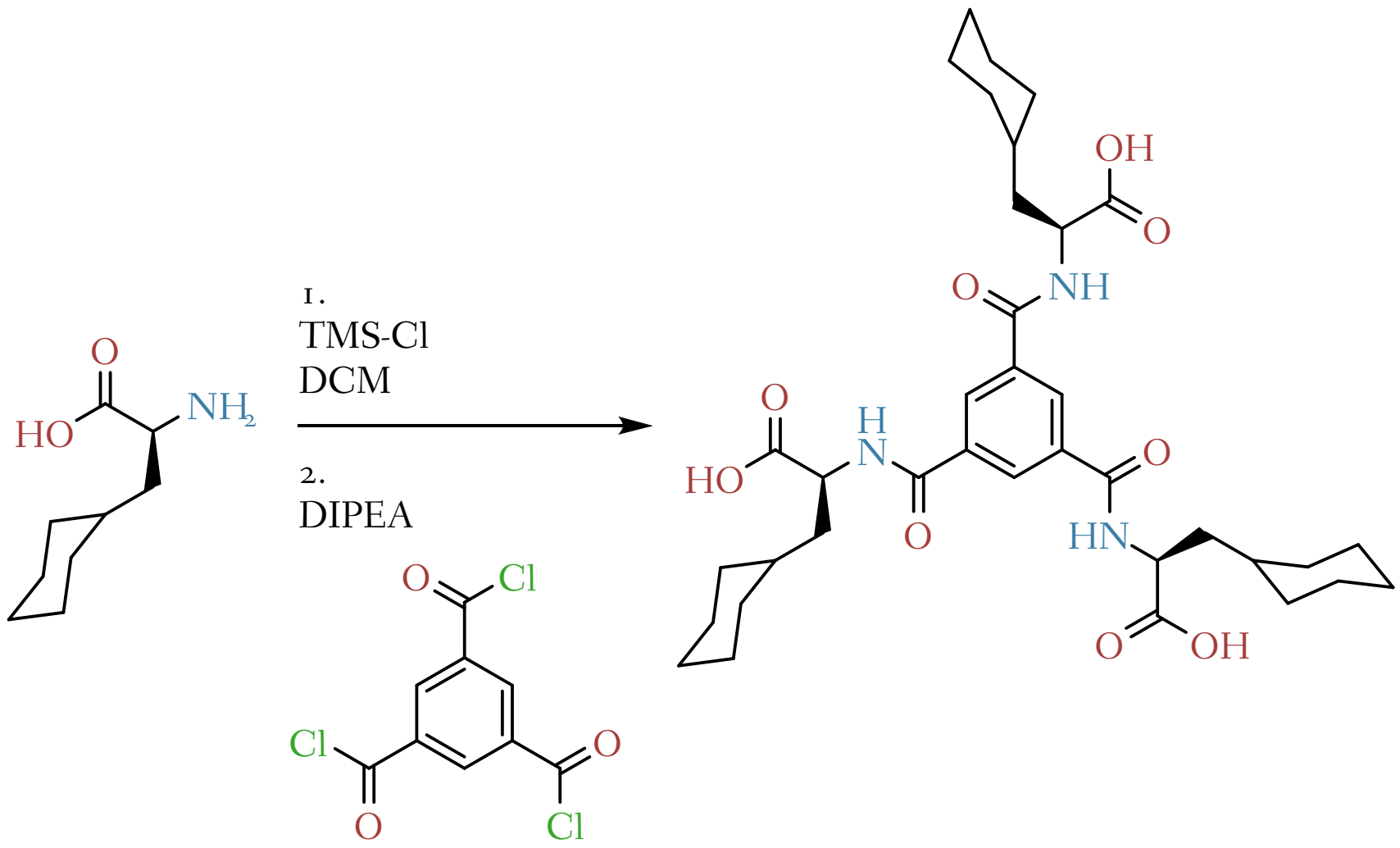

Figure S15. The synthesis of 8.

TMSCl (0.87 g, 8.0 mmol) was added to a suspension of L-3-cyclohexylalanin (0.685 g, 4.0 mmol) in dry CH_2Cl_2 (10 mL) and refluxed under an argon atmosphere for 8 h. The mixture was cooled to 0 °C, and DIEA (1.05 g, 8.0 mmol) and BTC-Cl (0.26 g, 1.0 mmol) were added. The reaction was allowed to warm to 25 °C and stirred for 20 h. The resulting mixture was concentrated in vacuo to provide a yellow solid which was then suspended in 1 M HCl (50 mL). The resulting solid was collected by filtration, washed with water and Et_2O and dried in vacuo. The pure product was obtained as a white solid in 61% yield.

^{1}H NMR (300 MHz, DMSO-d_6) δ 12.61 (s, 1H), 8.92 (d, *J* = 7.8 Hz, 1H), 8.46 (s, 1H), 4.50 (ddd, *J* = 10.6, 7.7, 4.4 Hz, 1H), 2.09 – 1.52 (m, 2H), 1.50 – 1.33 (m, 2H), 1.30 – 1.22 (m, 1H), 1.17 (d, *J* = 10.7 Hz, 1H), 1.07 – 0.78 (m, 2H).

^{13}C NMR (75 MHz, DMSO-d_6) δ 174.16, 165.91, 134.56, 129.25, 50.36, 37.88, 33.75, 33.26, 31.45, 26.07, 25.76, 25.59.

HRMS (ESI) Calcd for $C_{36}H_{51}N_3O_9Na$ $[M+Na]^+$: 692.3518 m/z, Found: $[M+Na]^+$ = 692.3539 m/z.

FT-IR (KBr) ν$_{max}$ [cm^{-1}] = 3346, 3077, 2925, 2852, 1725, 1648, 1596, 1535, 1449, 1411, 1332, 1316, 1297, 1279, 1266, 1246, 1201, 1149.

2.13 Synthesis of 9

Preparation of 9-*t*Bu

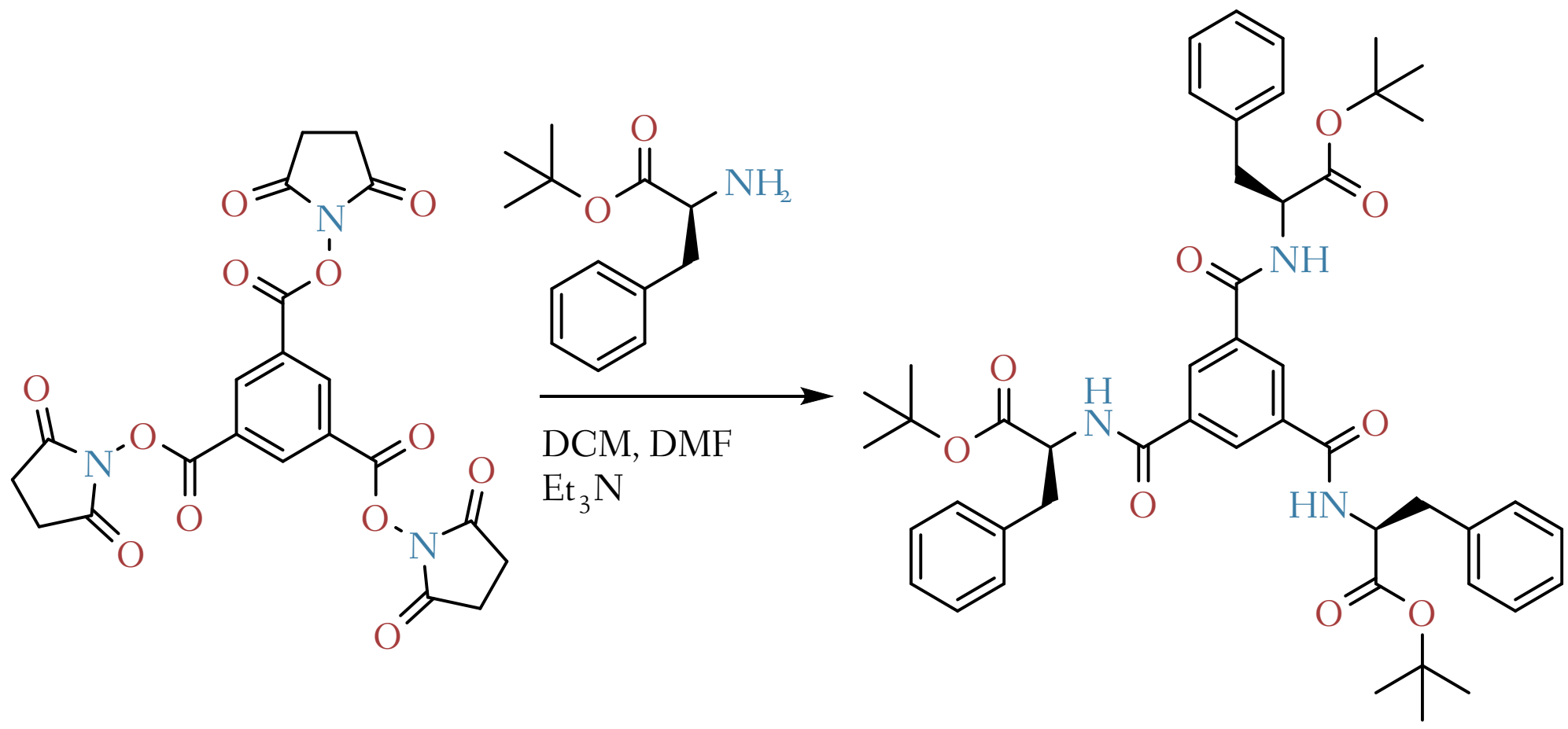

*Figure S16. The synthesis of **9-tBu***

To the solution of *N*-succinimidyl ester of 1,3,5-benzenetricarboxylic acid (501 mg, 1.0 mmol) in mixture of dry DCM/DMF (3:1, v/v, 16 mL), triethylamine (1.674 mL, 12.0 mmol) and L-phenylalanine tert-butyl ester (1.33 g, 6.0 mmol) were added under argon atmosphere. The mixture was stirred at RT for 24 h. After this time, DCM (25 mL) was added to the solution, and the mixture was washed with saturated water solution of $NaHCO_3$ (3 × 20 mL), then with 5% water solution of $KHSO_4$ (3 × 20 mL), and finally with brine (20 mL). The organic layer was dried over Na_2SO_4 and evaporated. After purification by column chromatography (1:2 EtOAc/hexanes), the pure product was obtained as a white solid in 50% yield.

^{1}H NMR (300 MHz, DMSO-*d*$_6$) δ 9.04 (d, *J* = 7.7 Hz, 1H), 8.38 (s, 1H), 7.34 – 7.26 (m, 3H), 7.20 (ddd, *J* = 8.5, 5.1, 2.7 Hz, 1H), 4.60 (q, *J* = 7.7 Hz, 1H), 3.17 – 3.06 (m, 2H), 1.35 (s, 9H).

^{13}C NMR (75 MHz, DMSO-*d*$_6$) δ 171.10, 166.08, 138.12, 134.85, 129.56, 128.67, 126.91, 81.22, 55.43, 36.93, 28.05.

Preparation of 9

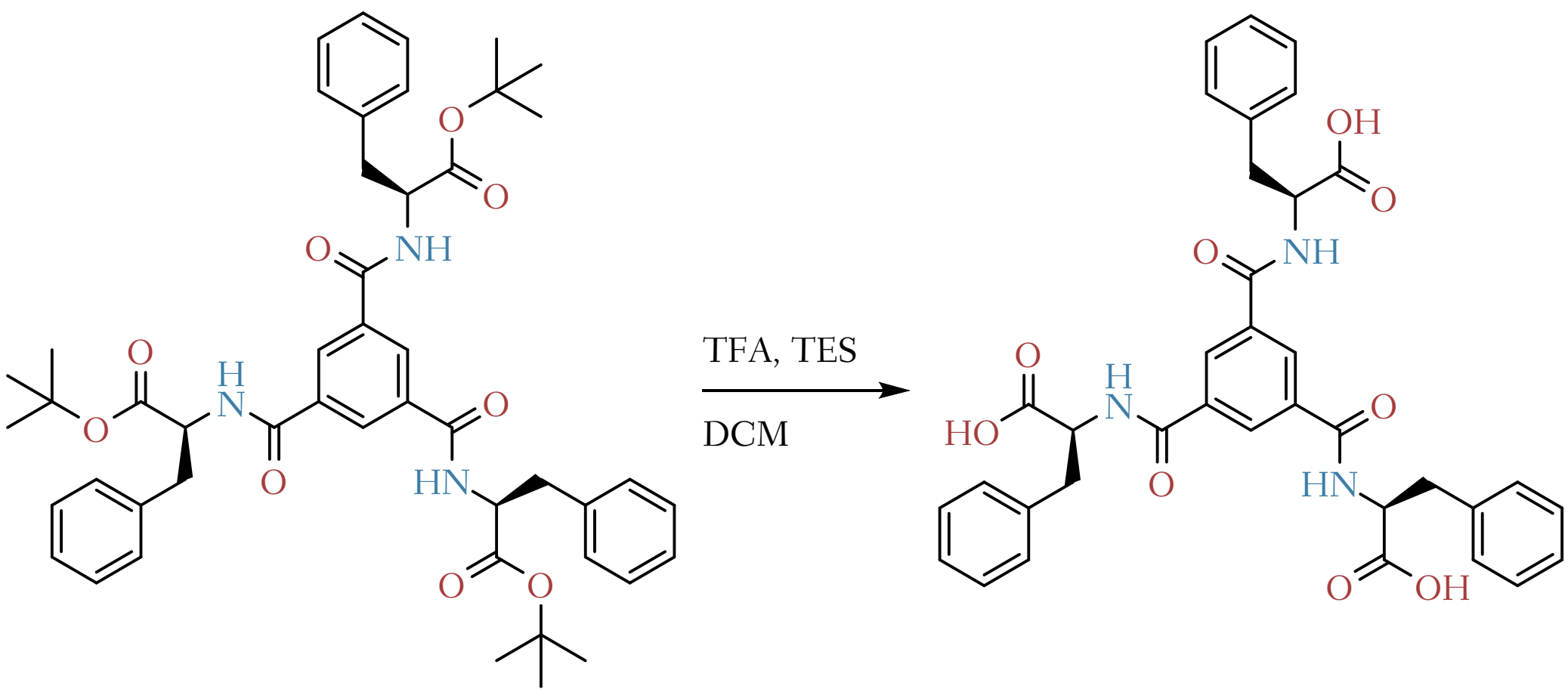

*Figure S17. The synthesis of **9***

To the solution of **9-*t*Bu** (148 mg, 0.18 mmol) in DCM (2 mL), trifluoroacetic acid (3.78 mL, 50 mmol) and triethylsilane (0.420 mL, 2.63 mmol) were added and reaction mixture was stirred at room temperature for 12 h. Afterwards, the solvents were evaporated under reduced pressure and the resulting paste was added ethyl acetate then evaporated again to remove residual TFA and dried in vacuo. The pure product was obtained as a white solid in 92% yield.

^{1}H NMR (600 MHz, DMSO-d_6) δ 12.83 (s, 1H), 8.99 (d, *J* = 8.1 Hz, 1H), 8.31 (s, 1H), 7.30 (d, *J* = 7.1 Hz, 2H), 7.26 (t, *J* = 7.5 Hz, 2H), 7.18 (t, *J* = 7.2 Hz, 1H), 4.66 (dd, *J* = 10.4, 8.1, 4.6 Hz, 1H), 3.20 (dd, *J* = 13.9, 4.6 Hz, 1H), 3.08 (dd, *J* = 13.9, 10.3 Hz, 1H).

^{13}C NMR (151 MHz, DMSO-d_6) δ 172.96, 165.60, 138.05, 134.42, 129.01, 128.24, 126.40, 54.34, 36.24.

HRMS (ESI) Calcd for $C_{36}H_{34}N_3O_9$ $[M+H]^+$: 652.2295 m/z, Found: $[M+H]^+$ = 652.2275 m/z.

FT-IR (KBr) $ν_{max}$ [cm^{-1}] = 3362, 3064, 3030, 2932, 1725, 1648, 1597, 1535, 1498, 1335, 1318, 1300, 1269, 1220, 740, 701, 680, 556.

2.14 Synthesis of 9 v2

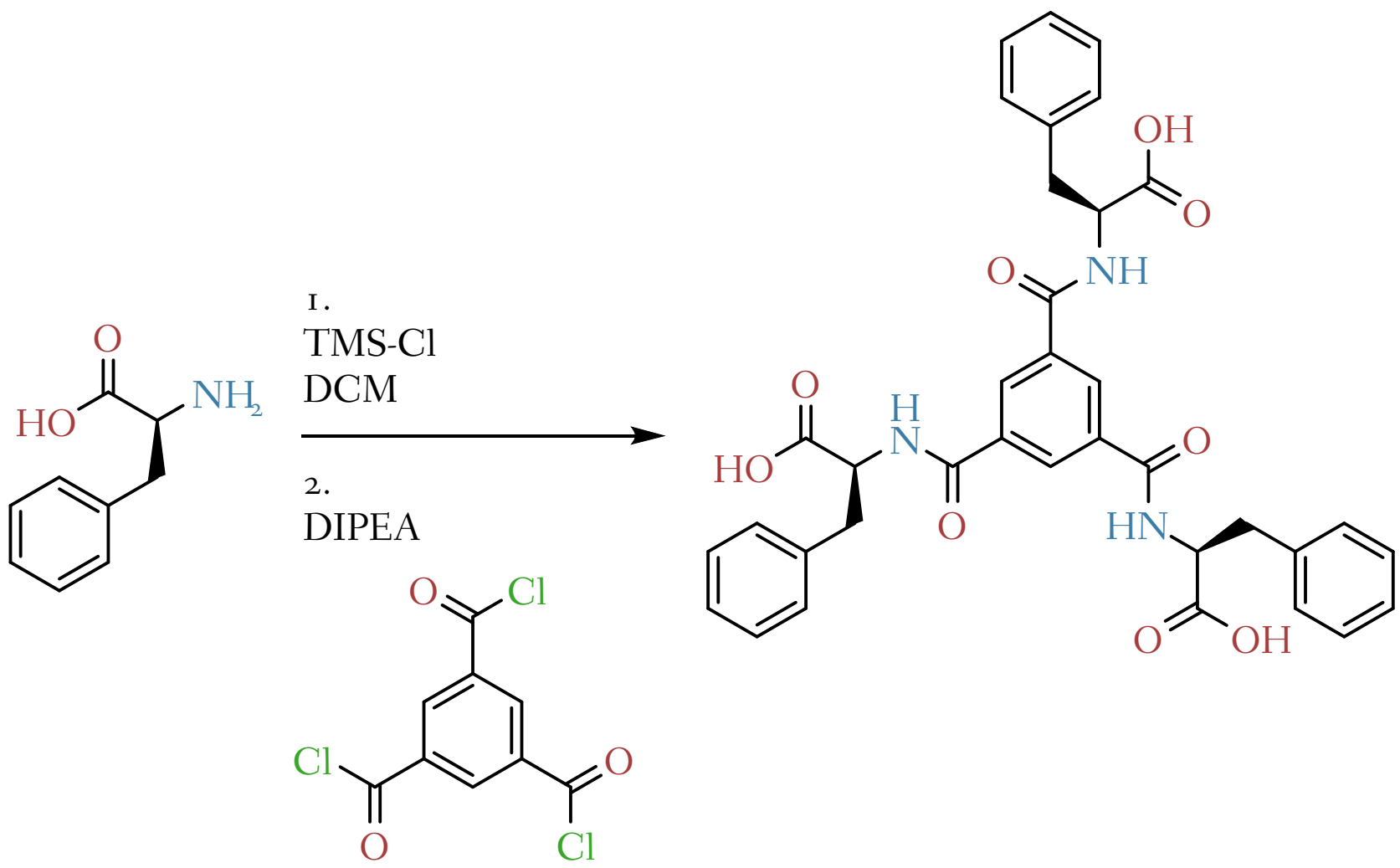

Figure S18. The synthesis of 9

TMSCl (1.74 g, 16.0 mmol) was added to a suspension of L-phenylalanine (1.320 g, 8.0 mmol) in dry CH_2Cl_2 (20 mL) and refluxed under an argon atmosphere for 8 h. The mixture was cooled to 0 °C, and DIEA (2.10 g, 16.0 mmol) and BTC-Cl (0.52 g, 2.0 mmol) were added. The reaction was allowed to warm to 25 °C and stirred for 20 h. The resulting mixture was concentrated in vacuo to provide a yellow solid which was then suspended in 1 M HCl (100 mL). The resulting solid was collected by filtration, washed with water and Et_2O and dried in vacuo. The pure product was obtained as a white solid in 68% yield.

^{1}H NMR (600 MHz, DMSO-d_6) δ 12.83 (s, 1H), 8.99 (d, *J* = 8.1 Hz, 1H), 8.31 (s, 1H), 7.30 (d, *J* = 7.1 Hz, 2H), 7.26 (t, *J* = 7.5 Hz, 2H), 7.18 (t, *J* = 7.2 Hz, 1H), 4.66 (dd, *J* = 10.4, 8.1, 4.6 Hz, 1H), 3.20 (dd, *J* = 13.9, 4.6 Hz, 1H), 3.08 (dd, *J* = 13.9, 10.3 Hz, 1H).

^{13}C NMR (151 MHz, DMSO-d_6) δ 172.96, 165.60, 138.05, 134.42, 129.01, 128.24, 126.40, 54.34, 36.24.

HRMS (ESI) Calcd for $C_{36}H_{34}N_3O_9$ $[M+H]^+$: 652.2295 m/z, Found: $[M+H]^+$ = 652.2275 m/z..

FT-IR (KBr) $ν_{max}$ [cm^{-1}] = 3362, 3064, 3030, 2932, 1725, 1648, 1597, 1535, 1498, 1335, 1318, 1300, 1269, 1220, 740, 701, 680, 556.

2.15 Synthesis of 10

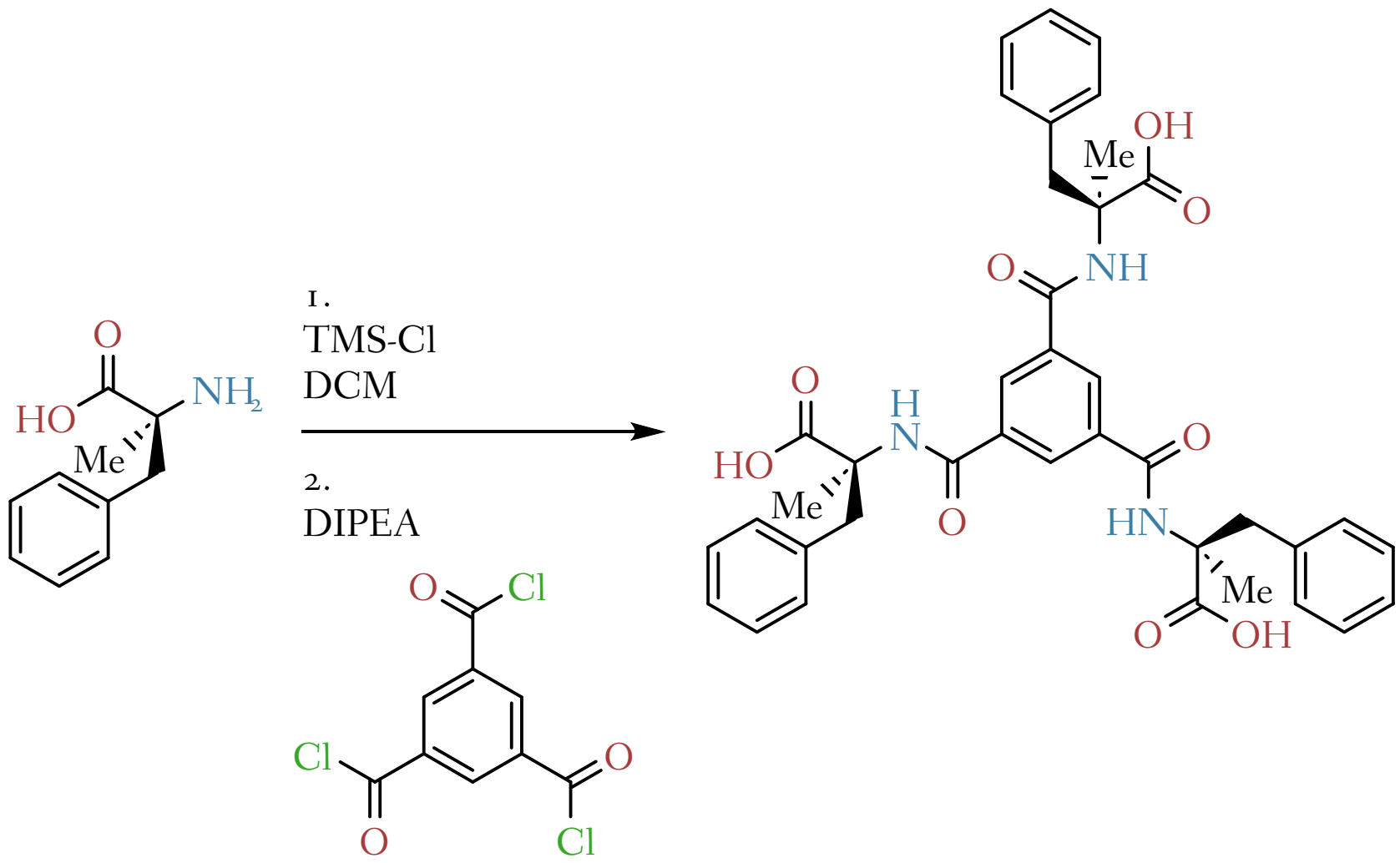

Figure S19. The synthesis of 10.

TMSCl (0.87 g, 8.0 mmol) was added to a suspension of α-methyl- L-phenylalanine (0.716 g, 4.0 mmol) in dry CH_2Cl_2 (10 mL) and refluxed under an argon atmosphere for 8 h. The mixture was cooled to 0 °C, and DIEA (1.05 g, 8.0 mmol) and BTC-Cl (0.26 g, 1.0 mmol) were added. The reaction was allowed to warm to 25 °C and stirred for 20 h. The resulting mixture was concentrated in vacuo to provide a yellow solid which was then suspended in 1 M HCl (50 mL). The resulting solid was collected by filtration, washed with water and Et_2O and dried in vacuo. The pure product was obtained as a white solid in 65% yield.

^{1}H NMR (300 MHz, DMSO-d_6) δ 12.51 (s, 1H), 8.51 (s, 1H), 8.32 (s, 1H), 7.33 – 7.16 (m, 3H), 7.15 – 7.09 (m, 2H), 3.49 (d, *J* = 13.3 Hz, 1H), 3.10 (d, *J* = 13.1 Hz, 1H), 1.36 (s, 3H).

^{13}C NMR (75 MHz, DMSO-d_6) δ 174.87, 165.42, 136.81, 134.75, 130.53, 129.25, 127.91, 126.52, 59.08, 22.35.

HRMS (ESI) Calcd for $C_{39}H_{39}N_3O_9Na$ $[M+Na]^+$: 716.2579 m/z, Found: $[M+Na]^+$ = 716.2539 m/z.

FT-IR (KBr) $ν_{max}$ [cm^{-1}] = 3388, 3063, 3031, 2990, 2942, 1724, 1656, 1519, 1454, 1377, 1320, 1289, 1213, 1125, 741, 703.

2.16 Synthesis of 11

Preparation of α-methyl- L-tyrosine methyl ester hydrochloride (α-Me_L-Tyr- *OMe_HCl*).

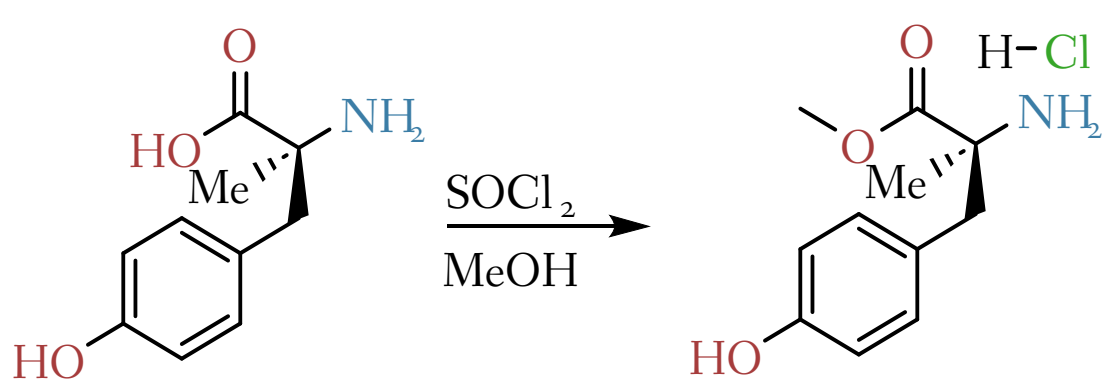

Figure S20. The synthesis of □-Me_L-Tyr-OMe_HCl.

To 20 mL of cooled to 0°C methyl alcohol, (2.0 mL 35.0 mmol) of thionyl chloride (SOCl₂) was slowly added dropwise. After the complete addition of SOCl₂, the mixture was left to reach room temperature, after which (0.976 g 5.0 mmol) of α-methyl- L-tyrosine (α-Me_ L-Tyr) was added, and the mixture was kept under reflux for 24 h. After this time, the reaction mixture became clear. The mixture was concentrated in reduced pressure, precipitated in diethyl ether (50 mL), filtered, and dried in vacuo. The pure product was obtained as a white solid in 96% yield.

^{1}H NMR (300 MHz, DMSO-*d*$_6$) δ 9.49 (s, 1H), 8.57 (s, 3H), 6.95 (d, *J* = 8.5 Hz, 2H), 6.72 (d, *J* = 8.5 Hz, 2H), 3.72 (s, 3H), 3.02 (s, 2H), 1.48 (s, 3H).

Preparation of α-Me_L-Tyr-OMe_Boc

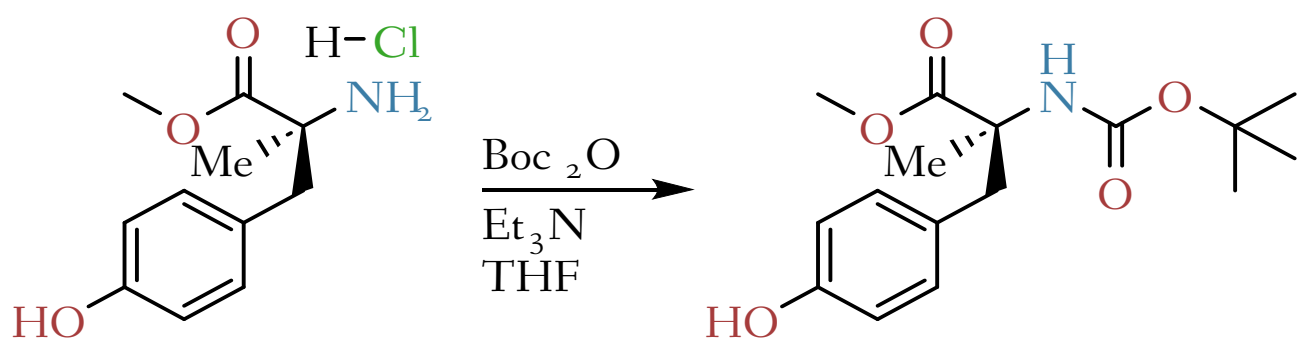

***Figure S21**. The synthesis of **□-Me_L-Tyr-OMe_Boc**.*

α-Me_L-Tyr-OMe_HCl (1.670 g, 5.2 mmol), Boc$_2$O (1.135 g, 5.2 mmol), and triethylamine (0.837 mL, 6 mmol) were stirred in 50 mL of dry THF at RT overnight. THF was removed in vacuo. The mixture was dissolved in EtOAc, washed three times with water, and dried over sodium sulphate. The pure product was obtained as a white solid in 68% yield.

^{1}H NMR (300 MHz, DMSO-*d*$_6$) δ 9.25 (s, 1H), 6.85 (d, *J* = 8.5 Hz, 2H), 6.64 (d, *J* = 8.5 Hz, 2H), 3.58 (s, 3H), 3.06 (d, *J* = 13.6 Hz, 1H), 2.78 (d, *J* = 13.4 Hz, 1H), 1.46 (s, 2H), 1.38 (s, 9H).

Preparation of α-Me_L-Tyr-OMe_Boc_C8

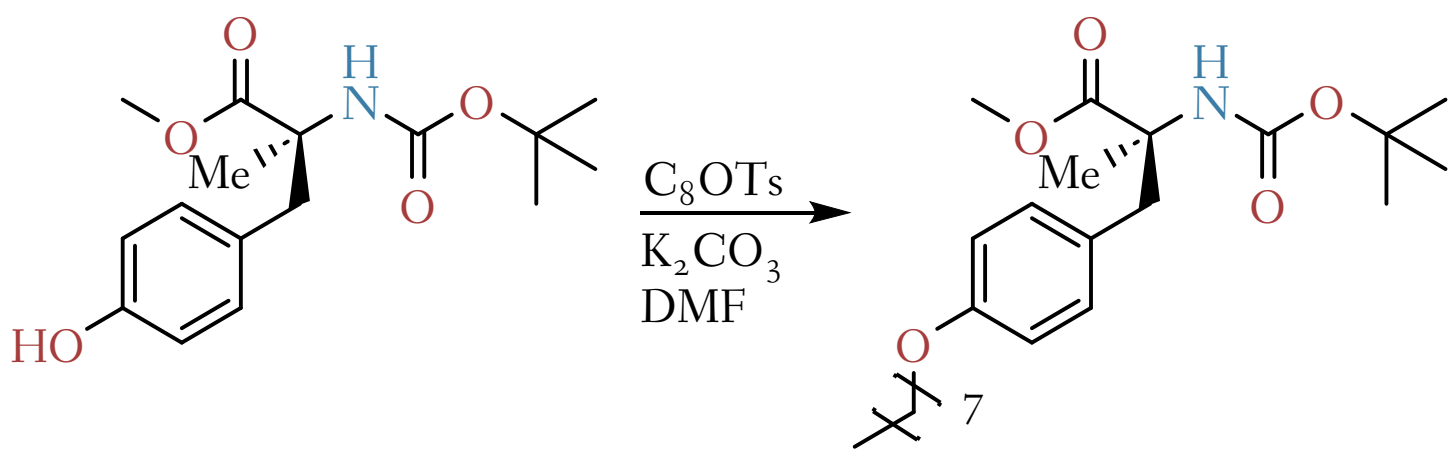

***Figure S22**. The synthesis of **□-Me_ L-Tyr- OMe_Boc_C8**.*

To the solution of **α-Me_L-Tyr-OMe_Boc** (1.237 g, 4.0 mmol) in dry DMF (50 mL), *n*-octyl p-toluenesulfonate (1.706 g, 6.0 mmol) and K_2CO_3 (0.967 g, 7.0 mmol) were added. The mixture was stirred at 75°C for 48 h. After this time, EtOAc (50 mL) was added to the solution, and the mixture was washed with water (2 × 20 mL), then with 1 M solution of NaOH (2 × 20 mL), and finally with water (20 mL). The organic layer was dried over Na_2SO_4 and evaporated. After purification by column chromatography (1:9 EtOAc/hexanes), the pure product was obtained as an oil in 67% yield.

^{1}H NMR (300 MHz, DMSO-d_6) δ 8.41 (s, 1H), 7.05 (d, J = 8.6 Hz, 2H), 6.89 (d, J = 8.6 Hz, 2H), 3.93 (t, J = 6.4 Hz, 2H), 3.75 (s, 3H), 3.20 – 2.86 (m, 2H), 1.69 (p, J = 6.5 Hz, 2H), 1.46 (s, 3H), 1.44 – 1.18 (m, 10H), 0.94 – 0.74 (m, 3H).

Preparation of α-Me_L-Tyr_ OMe _C8

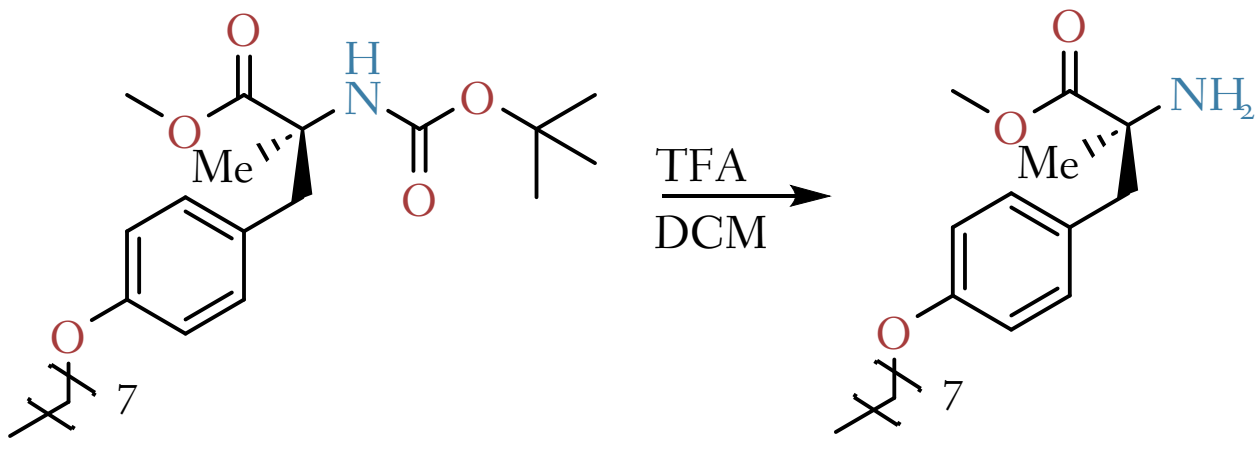

Figure S23.** The synthesis of **□-Me_L-Tyr- OMe _C8.

To the solution of **α-Me_L-Tyr- OMe_Boc_C8** (1.265 g, 3.0 mmol) in DCM (10 mL), trifluoroacetic acid (10 mL) were added and reaction mixture was stirred at room temperature for 12 h. Afterwards, the solvents were evaporated under reduced pressure and to the residue was added DCM (20 mL), then evaporated again to remove residual TFA and dried in vacuo. The pure product was obtained as a white solid in 94% yield.

^{1}**H NMR** (300 MHz, DMSO-d_6) δ 8.41 (s, 1H), 7.05 (d, *J* = 8.6 Hz, 2H), 6.89 (d, *J* = 8.6 Hz, 2H), 3.93 (t, *J* = 6.4 Hz, 2H), 3.75 (s, 3H), 3.20 – 2.86 (m, 2H), 1.69 (p, *J* = 6.5 Hz, 2H), 1.46 (s, 3H), 1.44 – 1.18 (m, 10H), 0.94 – 0.74 (m, 3H).

Preparation of 11-OMe

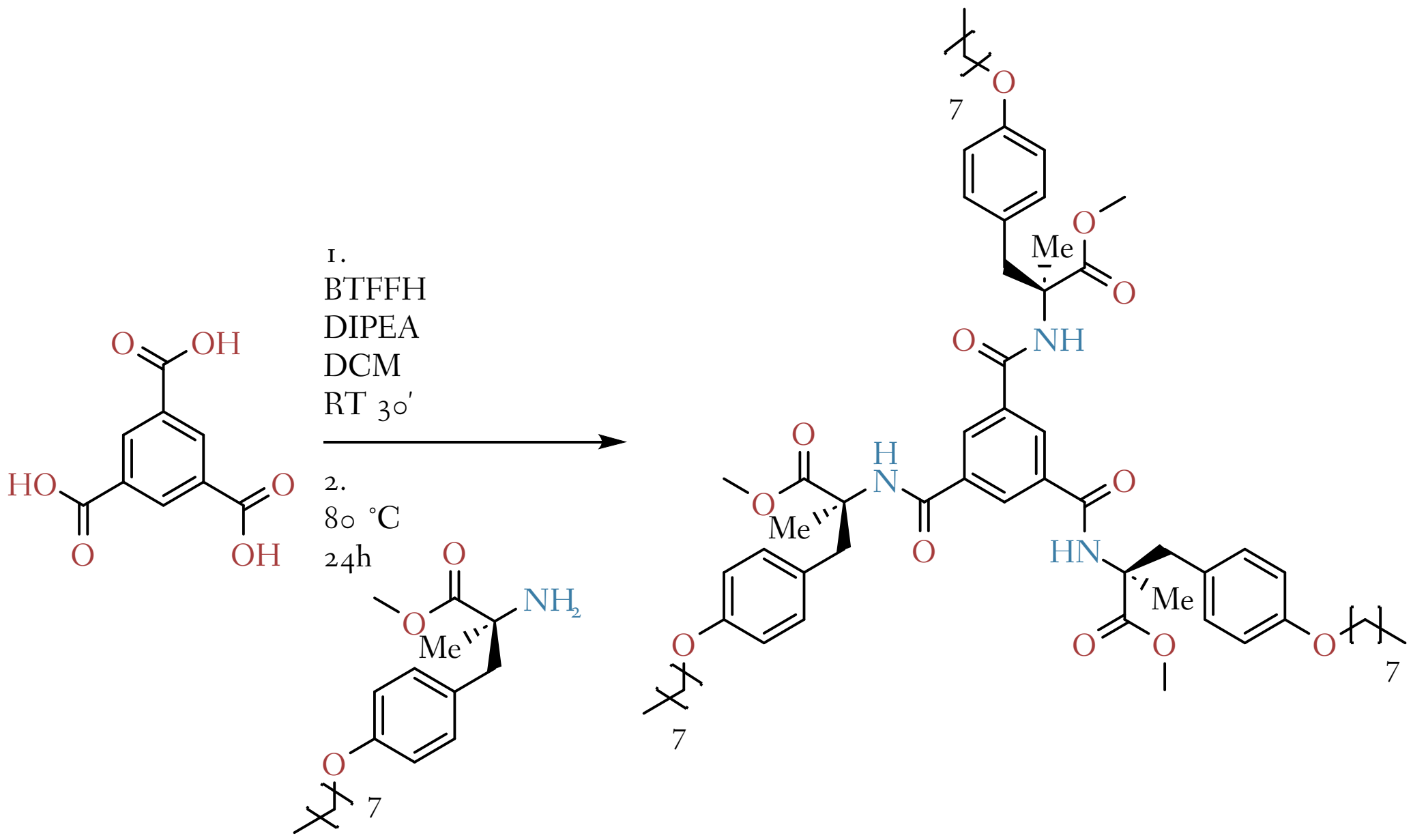

Figure S24.** The synthesis of **11-OMe.

To Schlenk flask was added benzene-1,3,5-tricarboxylic acid (0.168 g 0,8 mmol) and BTFFH (1.264 g, 4 mmol) in dry CH_2Cl_2 (6 mL) under argon. DIPEA (1.280 mL, 7.2 mmol) was subsequently added and the reaction was stirred under argon for 30 min. The α-Me_L-Tyr- OMe _C8 (1.280 g, 4.4 mmol) was added and the flask was sealed and heated in an oil bath at 80 °C 24 h under argon atmosphere. The reaction mixture was cooled to room temperature and evaporated under reduced pressure. The residue was dissolved in EtOAc (20 mL) and washed with 1 M HCl and brine, dried over Na_2SO_4 and concentrated. The crude product was purified by column chromatography 2/1 hexane/ethyl acetate. The pure product was obtained as a white solid in 51% yield.

^{1}**H NMR** (300 MHz, DMSO-d_6) δ 8.75 (s, 1H), 8.40 (s, 1H), 6.99 (d, *J* = 8.5 Hz, 2H), 6.81 (d, *J* = 8.6 Hz, 2H), 3.88 (t, *J* = 6.5 Hz, 2H), 3.60 (s, 3H), 3.38 (d, *J* = 13.4 Hz, 1H), 3.01 (d, *J* = 13.4 Hz, 1H), 1.64 (q, *J* = 6.9 Hz, 2H), 1.34 (s, 3H), 1.33 – 1.19 (m, 4H), 1.00 – 0.73 (m, 3H).

^{13}**C NMR** (75 MHz, DMSO-d_6) δ 173.69, 165.34, 157.59, 134.38, 131.43, 129.59, 127.78, 113.86, 67.29, 59.30, 51.86, 31.25, 28.72 (d, J = 7.0 Hz), 25.53, 22.10, 13.94.

Preparation of 11

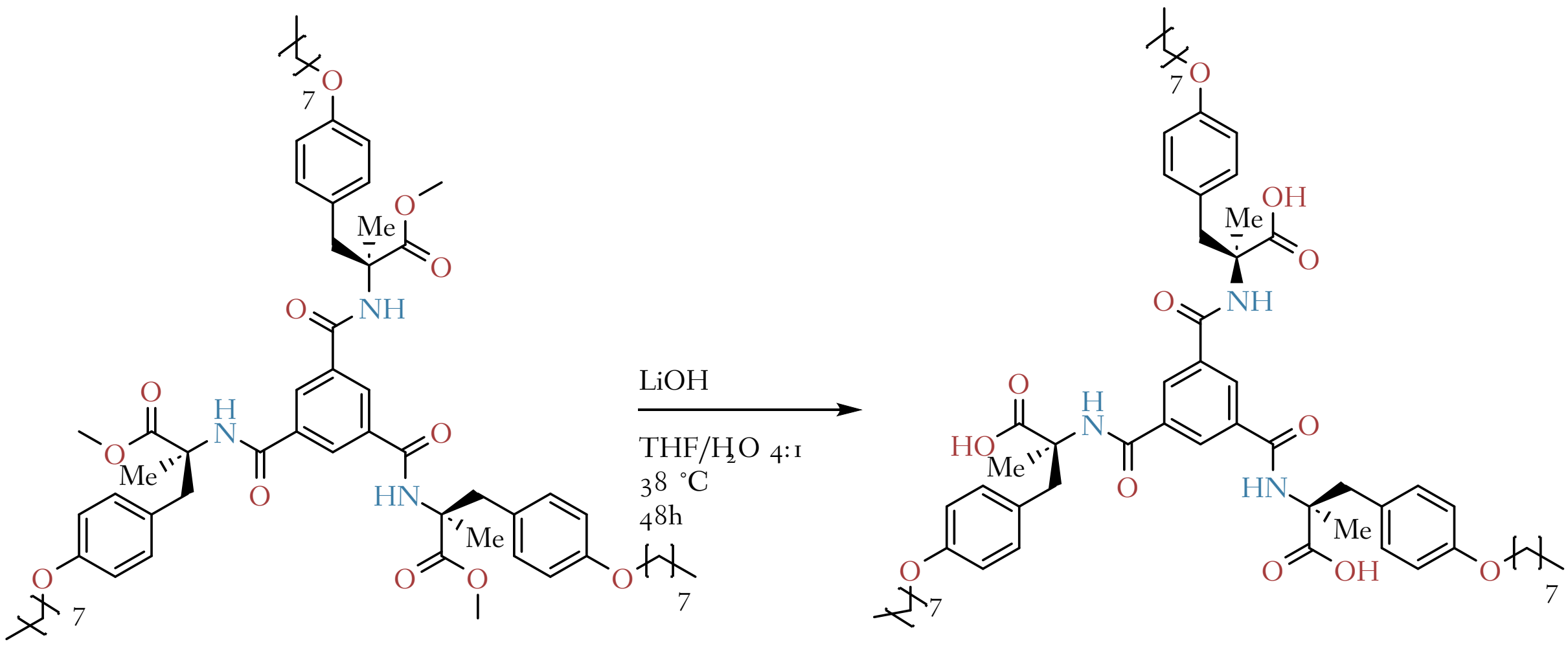

Figure S25. *The synthesis of* ***11.***

11-OMe (0.330 g, 0.3 mmol) was dissolved in a 4:1 THF/H₂O mixture (15 mL), and (0.045 g 1.8 mmol) of LiOH was added. The reaction mixture was heated at 38 °C for 48 h. After this time, the mixture was evaporated, dissolved in water (10 mL), and acidified to pH 1 using 1 M HCl. The resulting precipitate was filtered, washed with water, and dried under reduced pressure. The pure product was obtained as a white solid in 85% yield.

^{1}**H NMR** (300 MHz, DMSO-d_6) δ 12.45 (s, 1H), 8.48 (s, 1H), 7.01 (d, *J* = 8.6 Hz, 2H), 6.79 (d, *J* = 8.7 Hz, 2H), 3.86 (t, *J* = 6.5 Hz, 2H), 3.41 (d, *J* = 13.7 Hz, 1H), 3.01 (d, *J* = 13.5 Hz, 1H), 1.63 (q, *J* = 6.8 Hz, 2H), 1.34 (s, 3H), 1.24 (d, *J* = 4.7 Hz, 9H), 0.99 – 0.70 (m, 2H).

^{13}**C NMR** (75 MHz, DMSO-d_6) δ 174.88, 165.37, 157.48, 134.80, 131.40, 129.23, 128.38, 113.80, 67.29, 59.19, 31.26, 28.74 (d, *J* = 7.2 Hz), 25.55, 22.11, 13.96

HRMS (ESI) Calcd for $C_{63}H_{87}N_3O_{12}Na$ [M+Na]$^+$: 1100.6182 m/z, Found: [M+Na]$^+$ = 1100.6137 m/z.

FT-IR (KBr) ν$_{max}$ [cm^{-1}] = 3378, 3373, 3064, 3035, 2953, 2927, 2856, 1721, 1655, 1613, 1513, 1456, 1377, 1300, 1248, 1178, 1119, 1030, 836, 798.

3 NMR data

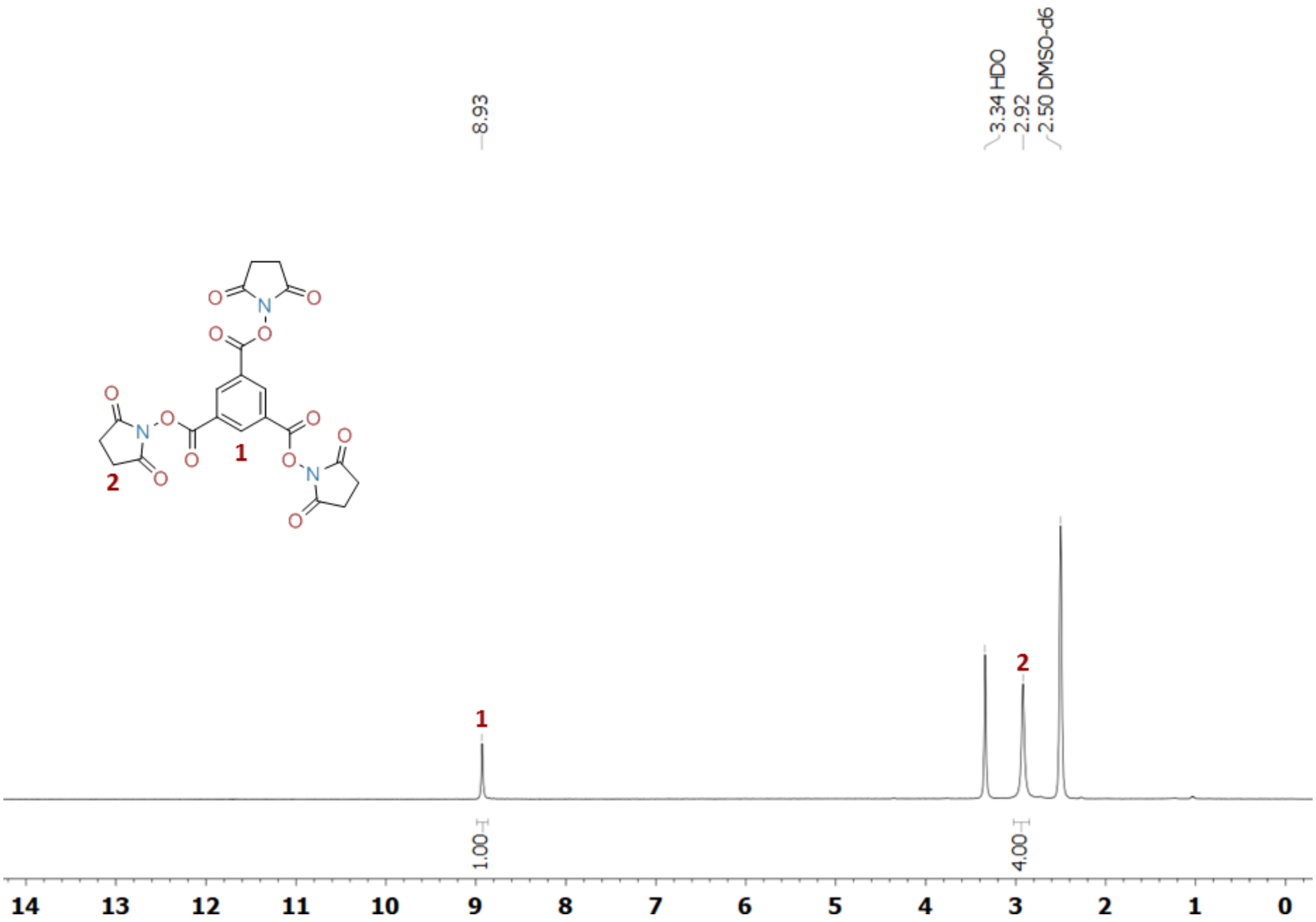

Figure S26. *^{1}H NMR (300 MHz DMSO-d_6) spectrum of* ***BTC-OSu***

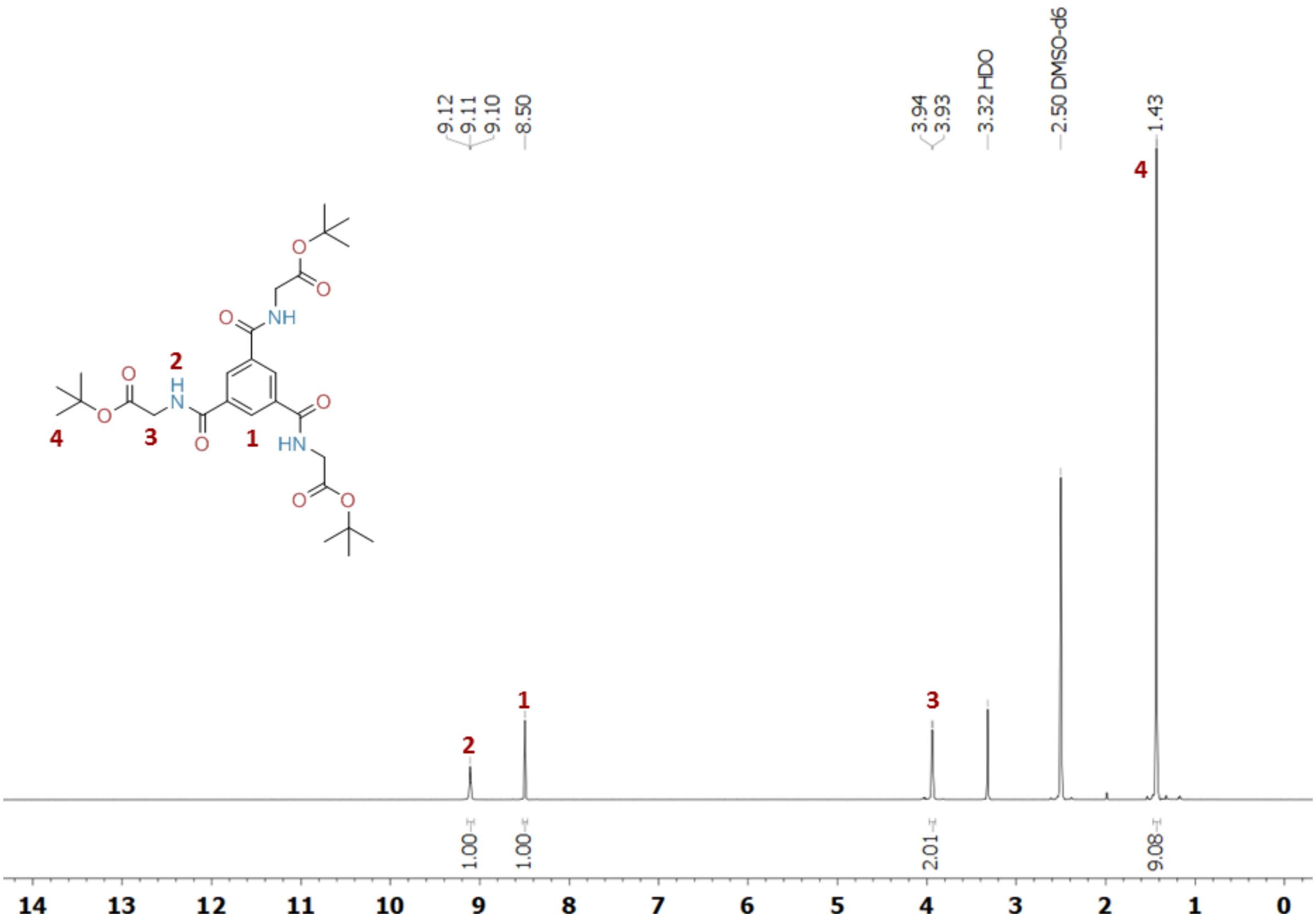

Figure S27. ^{1}H NMR (300 MHz DMSO-d_6) spectrum of **1-tBu**.

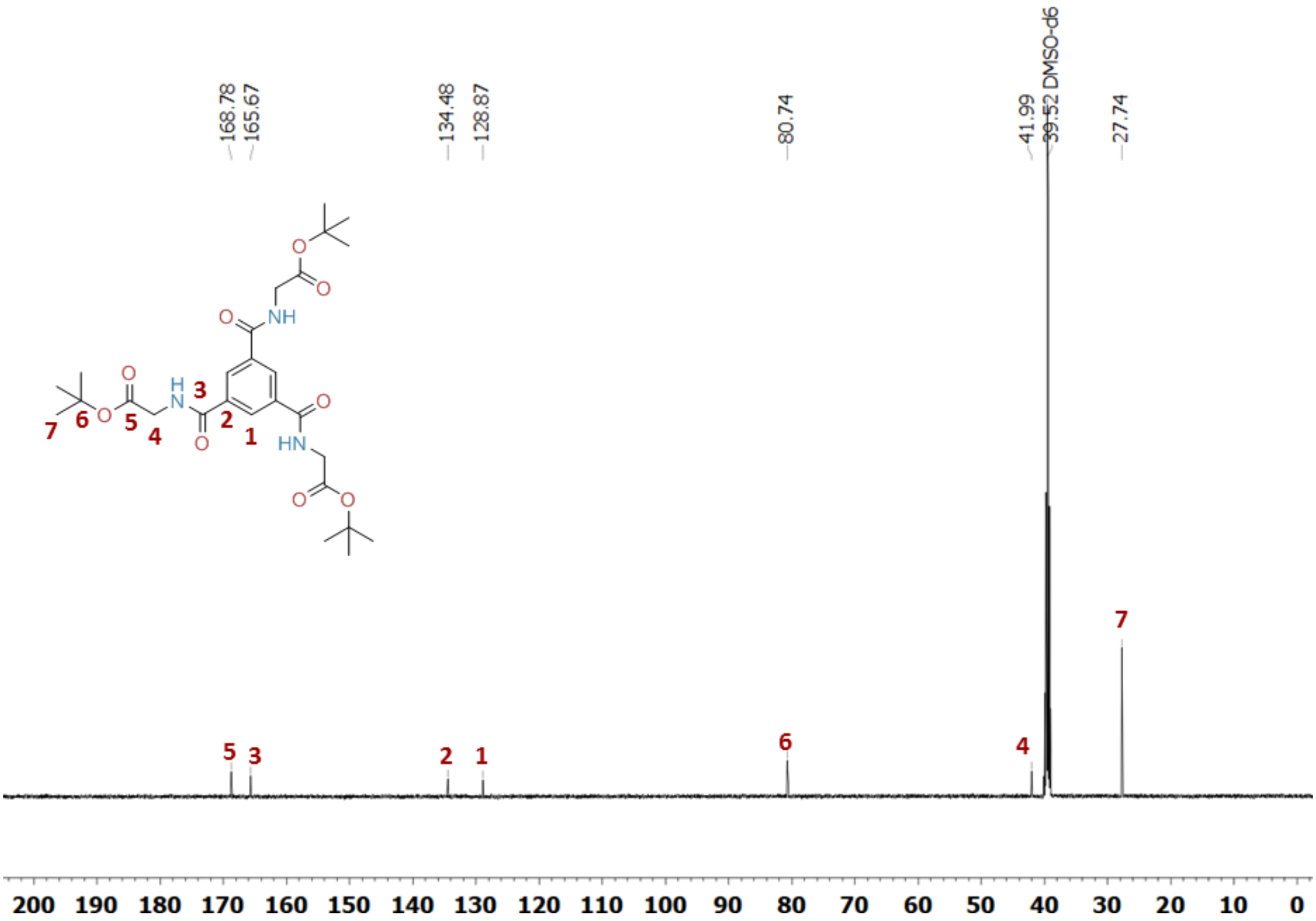

Figure S28. ^{13}C NMR (75 MHz DMSO-d_6) spectrum of **1-tBu**.

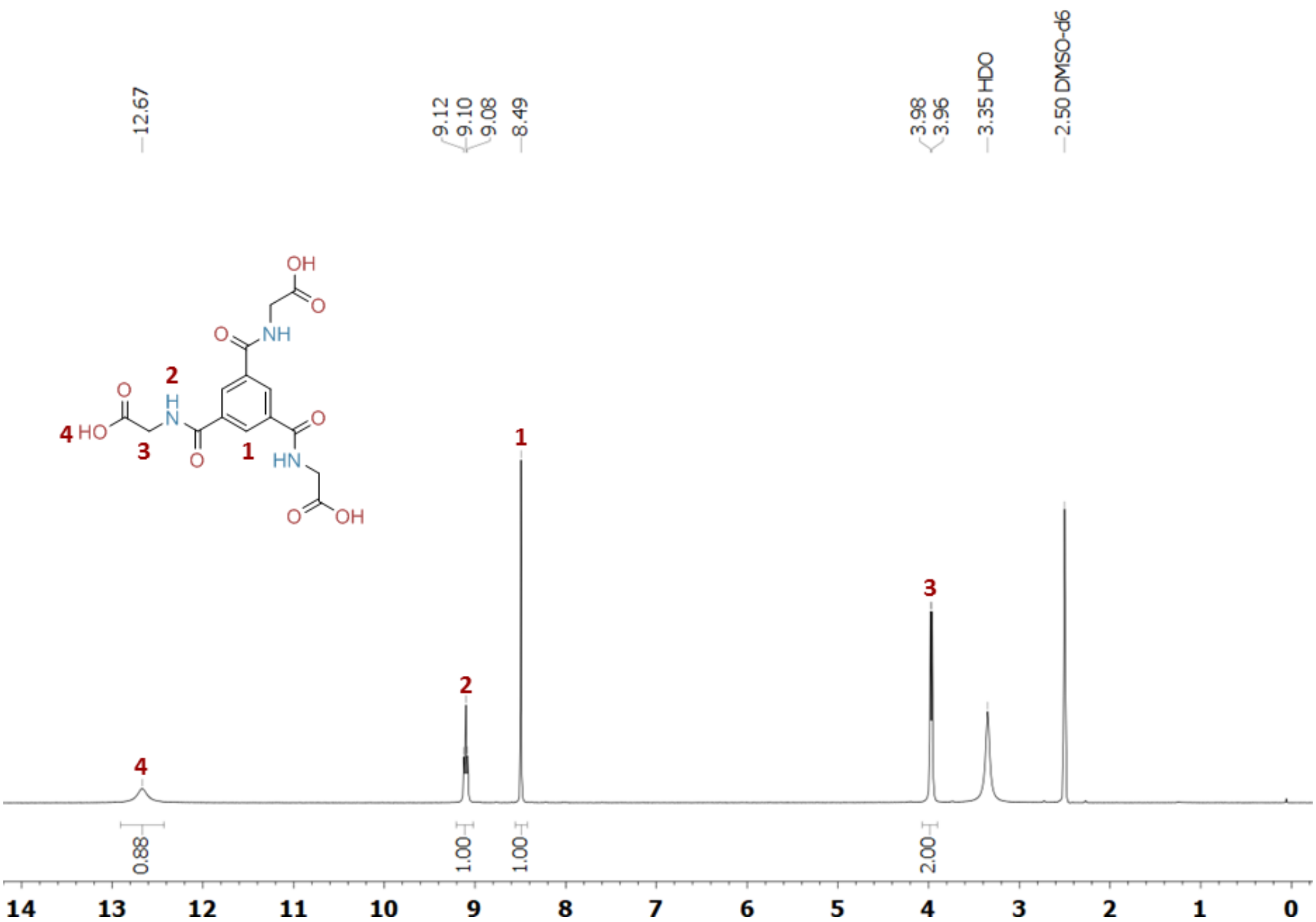

Figure S29. ^{1}H NMR (300 MHz DMSO-d_6) spectrum of **1**.

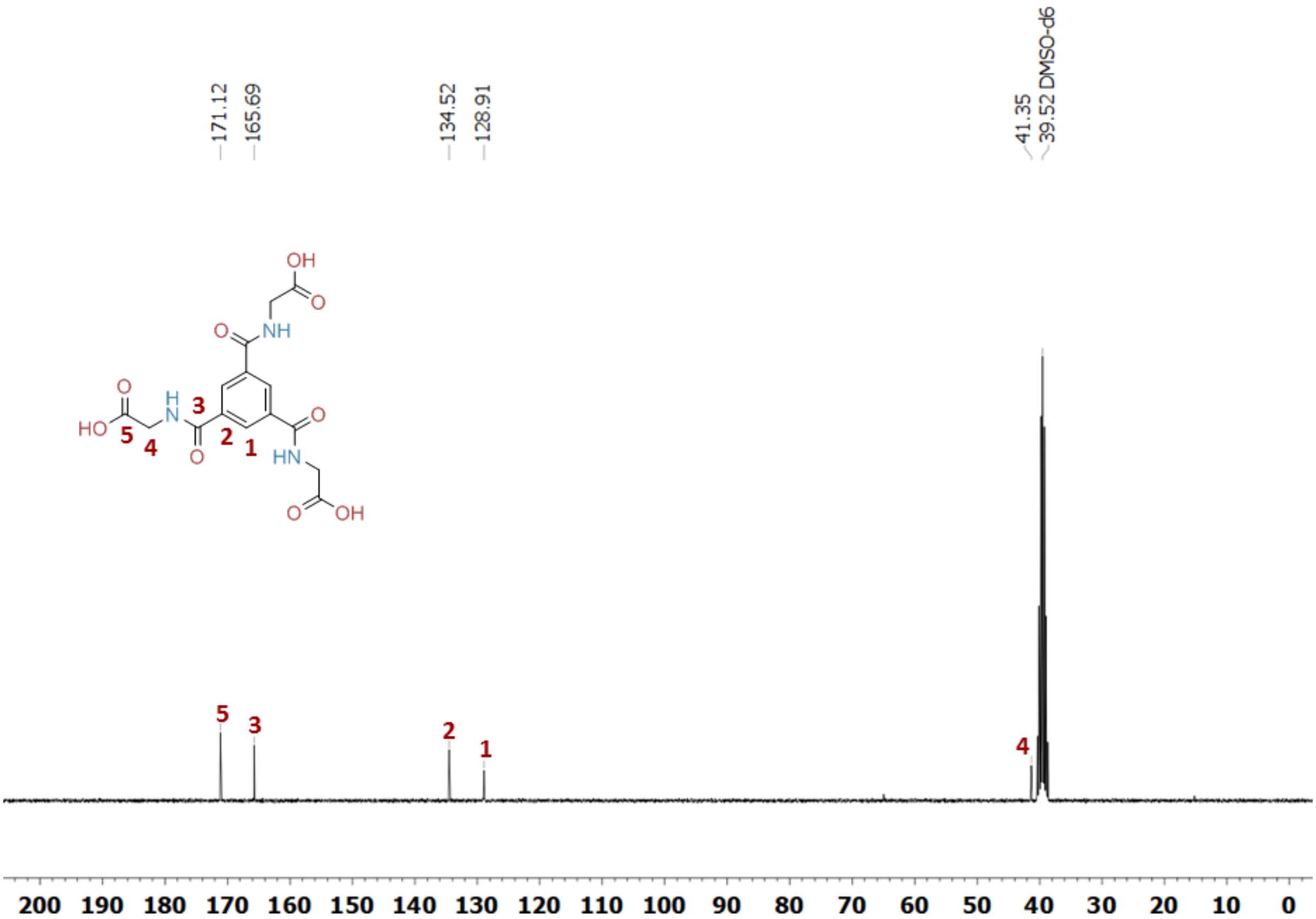

Figure S30. ^{13}C NMR (75 MHz DMSO-d_6) spectrum of **1**.

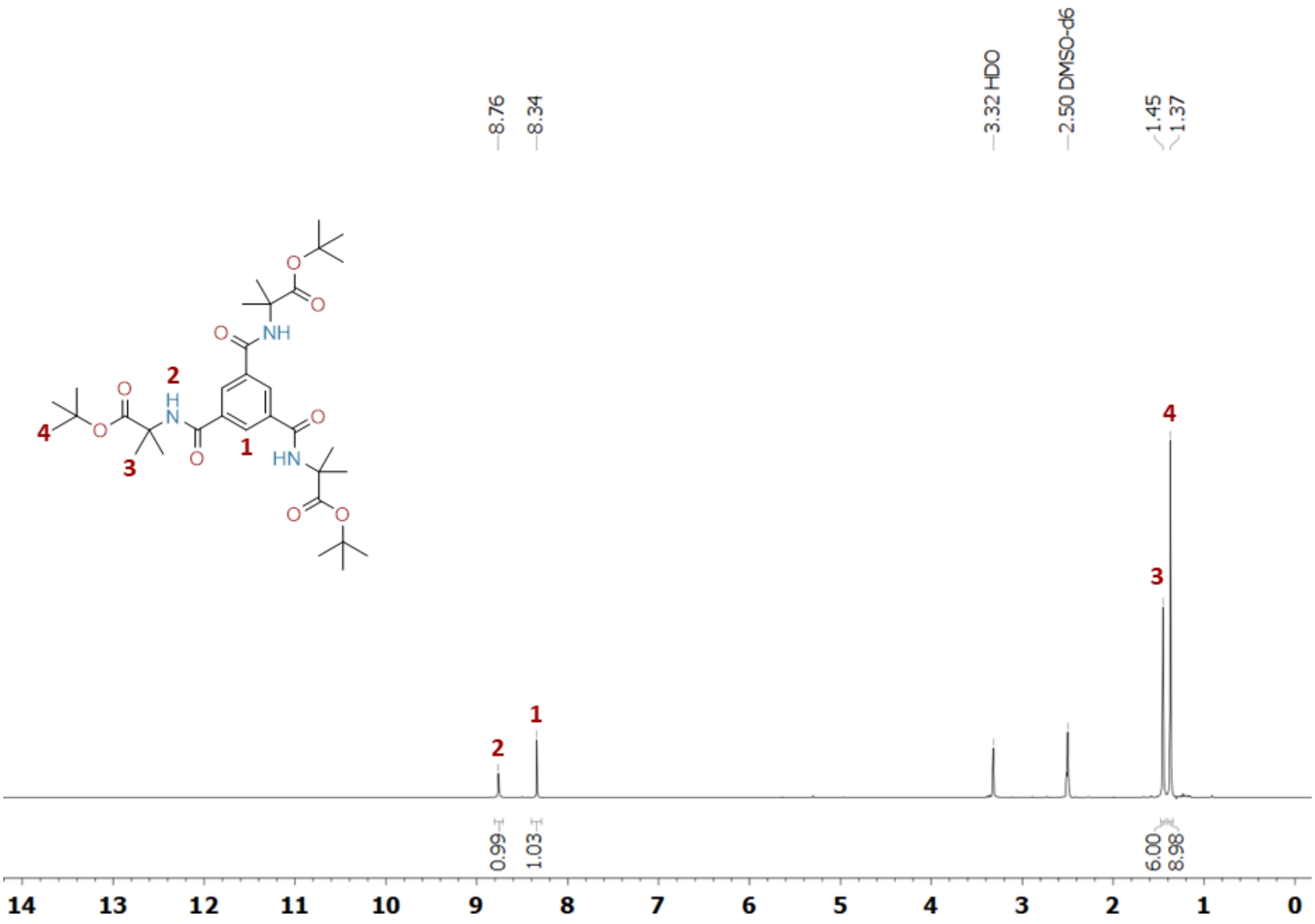

Figure S31. ^{1}H *NMR (300 MHz DMSO-d_6) spectrum of* ***2-tBu***.

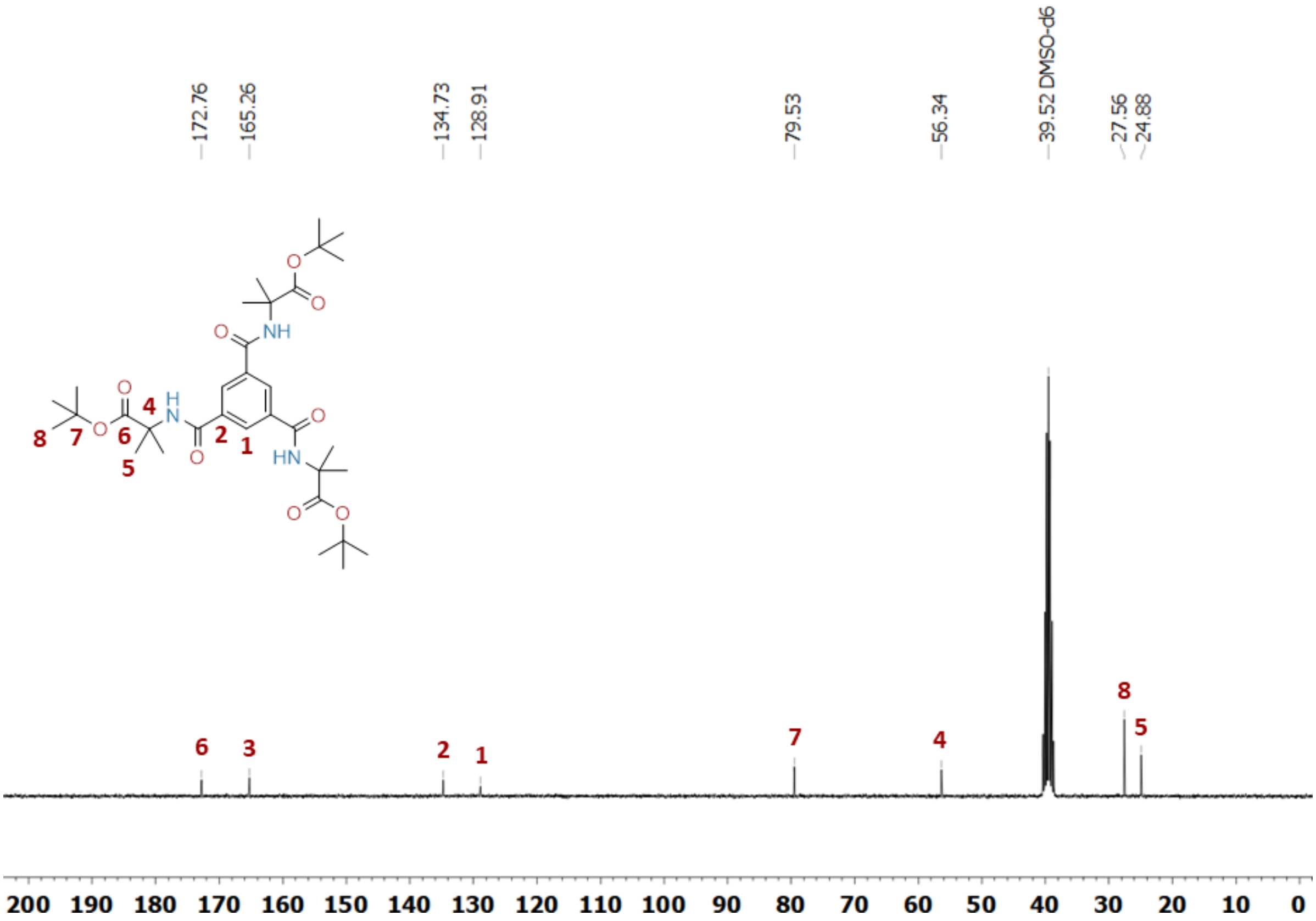

Figure S32. ^{13}C *NMR (75 MHz DMSO-d_6) spectrum of* ***2-tBu***.

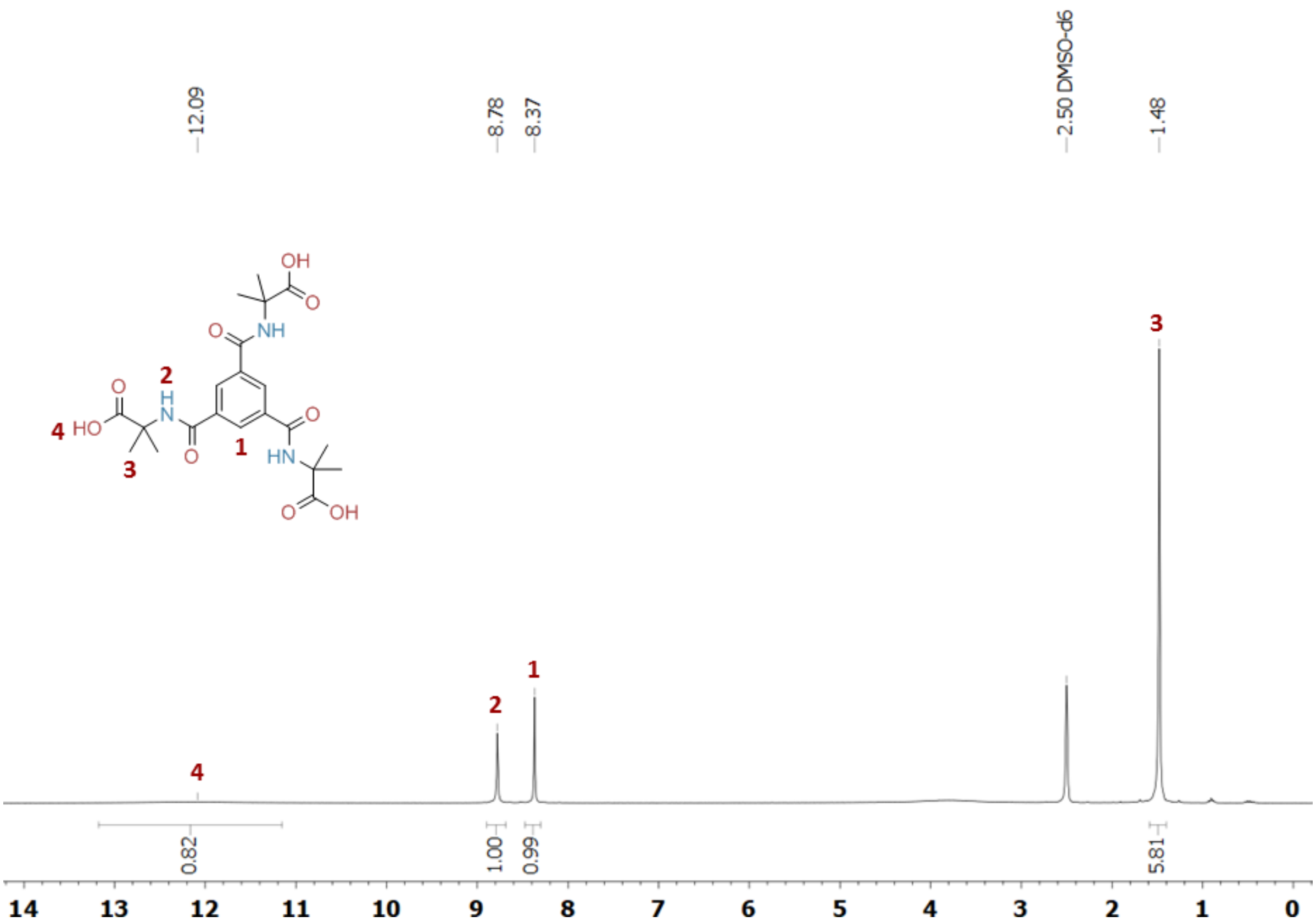

Figure S33. *^{1}H NMR (300 MHz DMSO-d$_6$) spectrum of* ***2.***

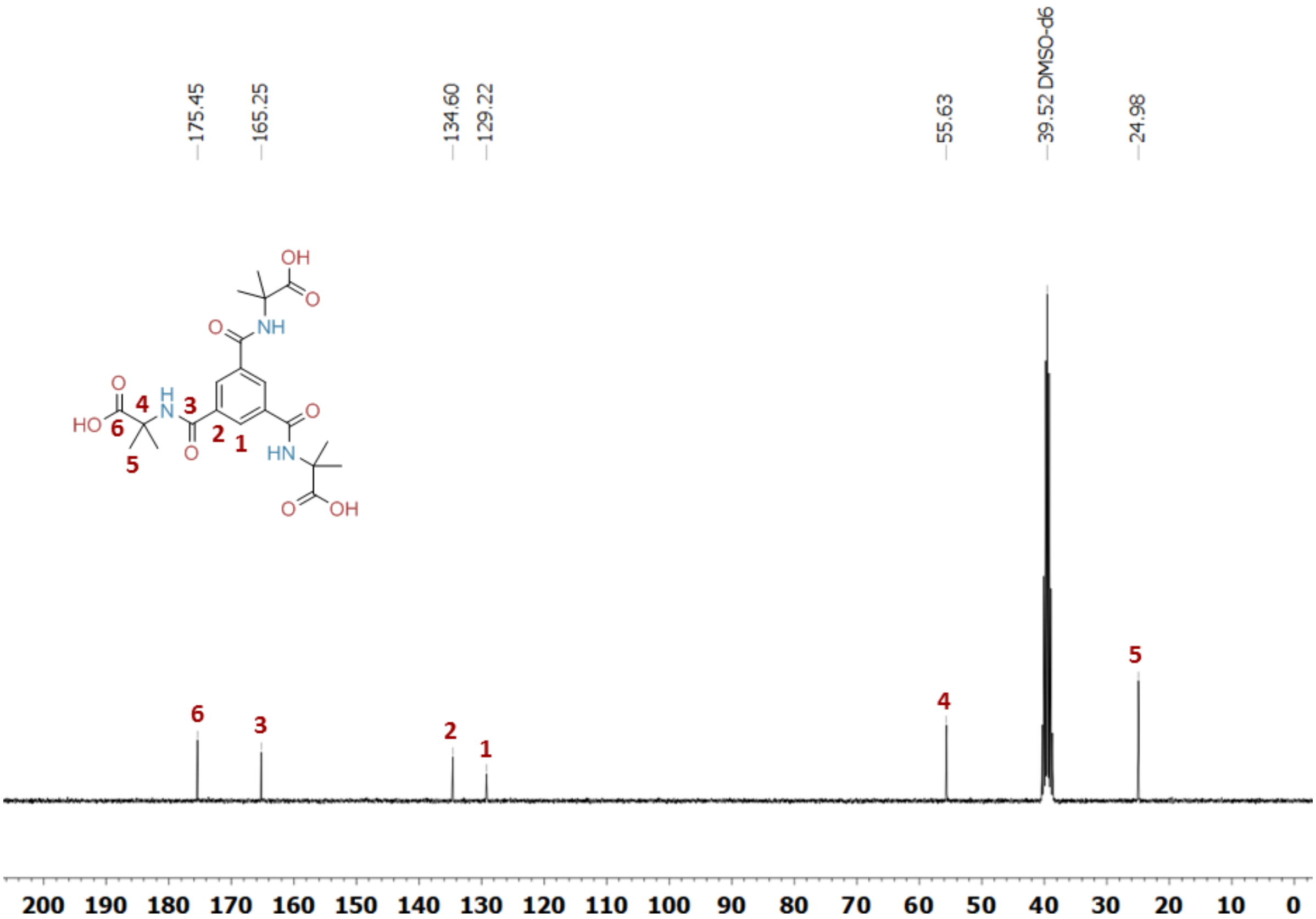

Figure S34. *^{13}C NMR (75 MHz DMSO-d$_6$) spectrum of* ***2.***

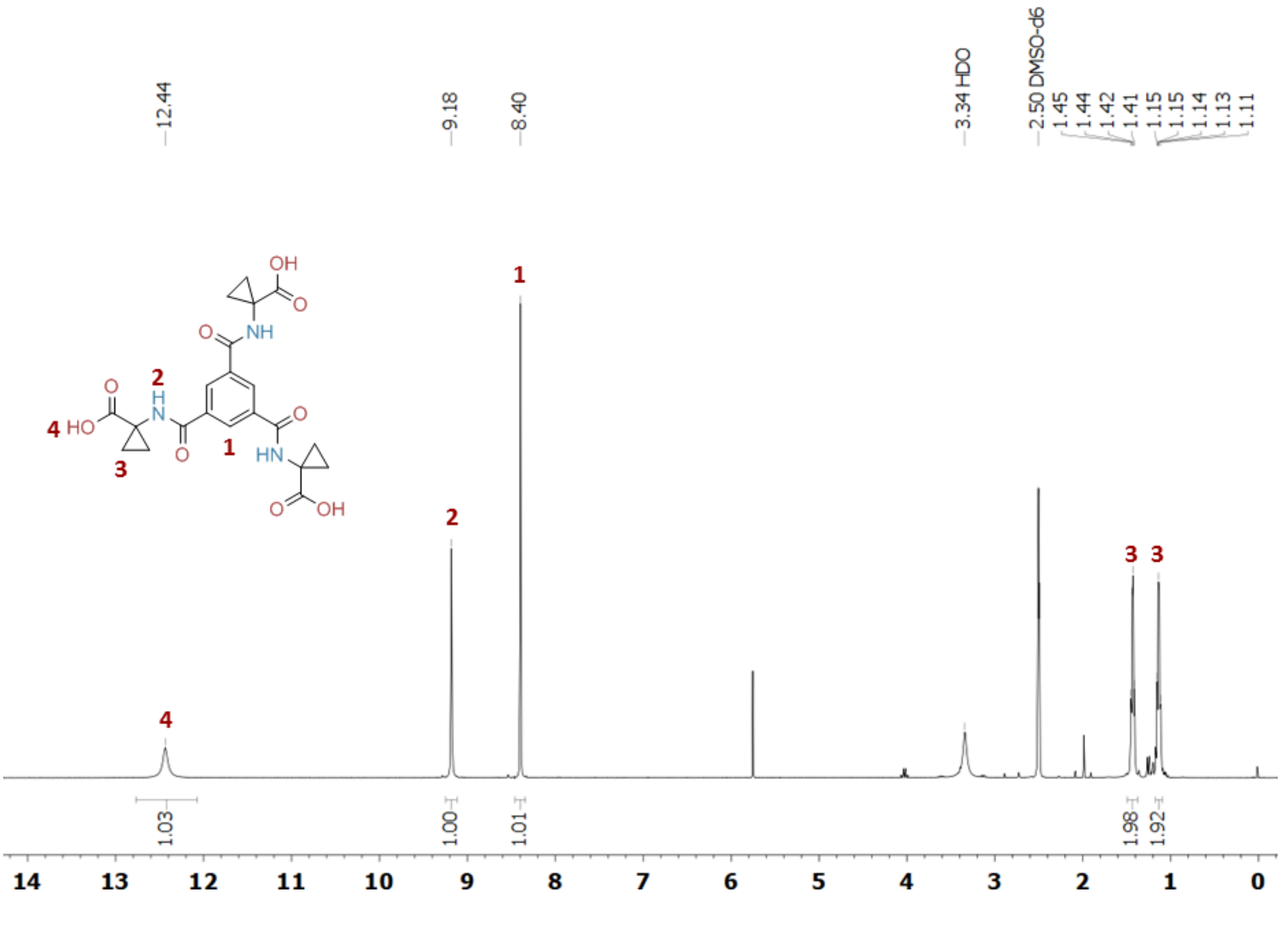

Figure S35. *^{1}H NMR (300 MHz DMSO-d_6) spectrum of **3**.*

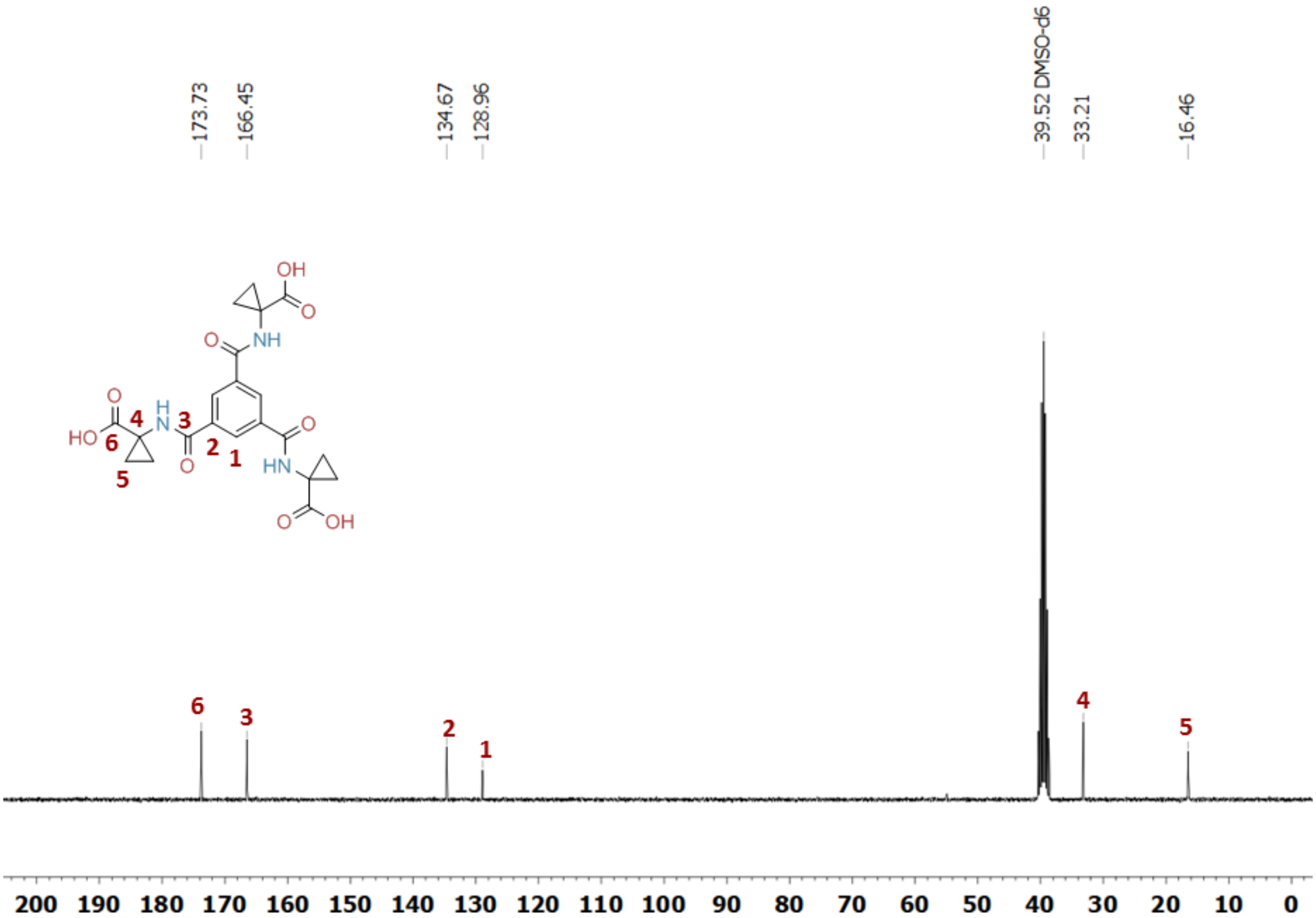

Figure S36. *^{13}C NMR (75 MHz DMSO-d_6) spectrum of **3**.*

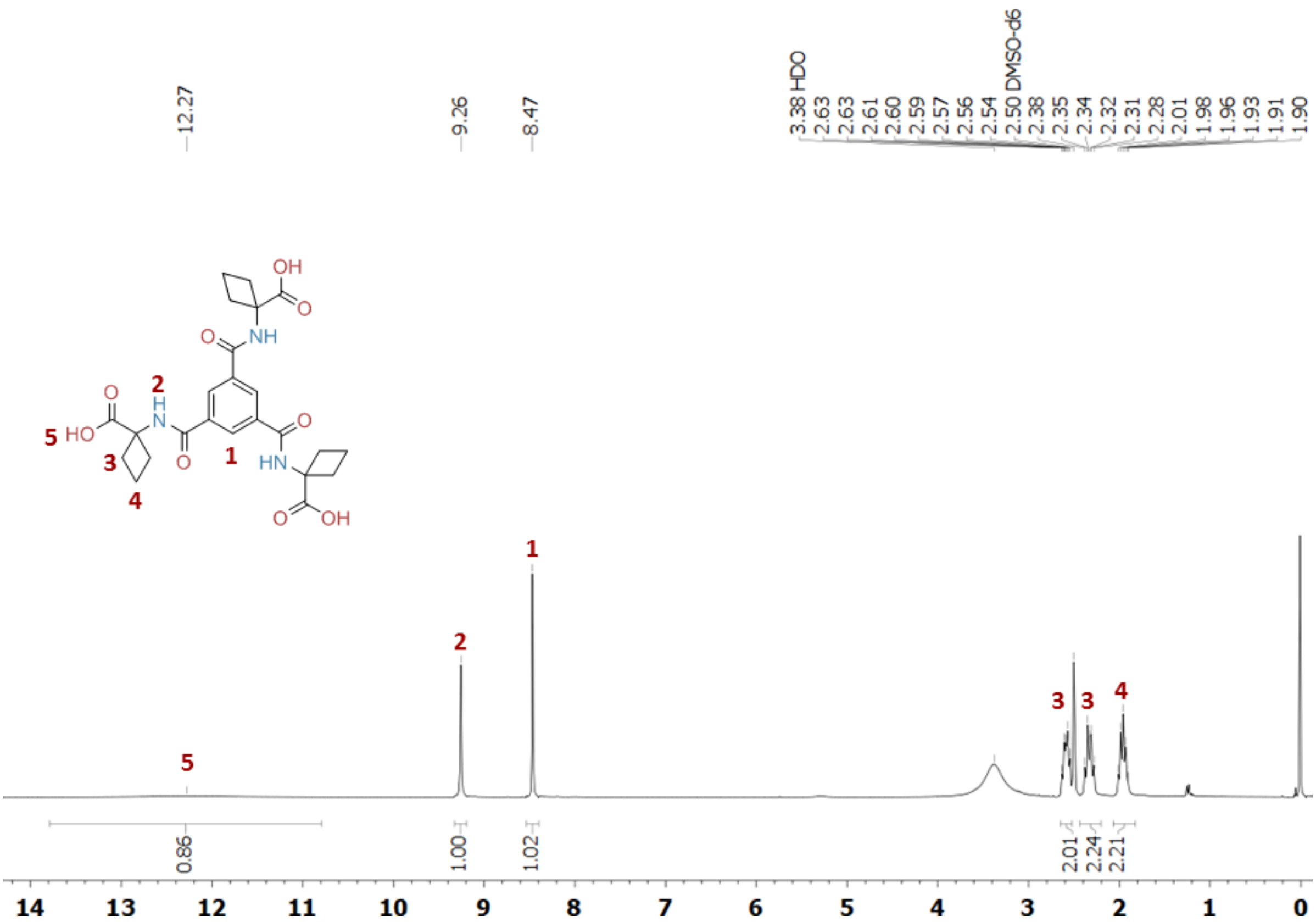

Figure S37. *^{1}H NMR (300 MHz DMSO-d_6) spectrum of* ***4***.

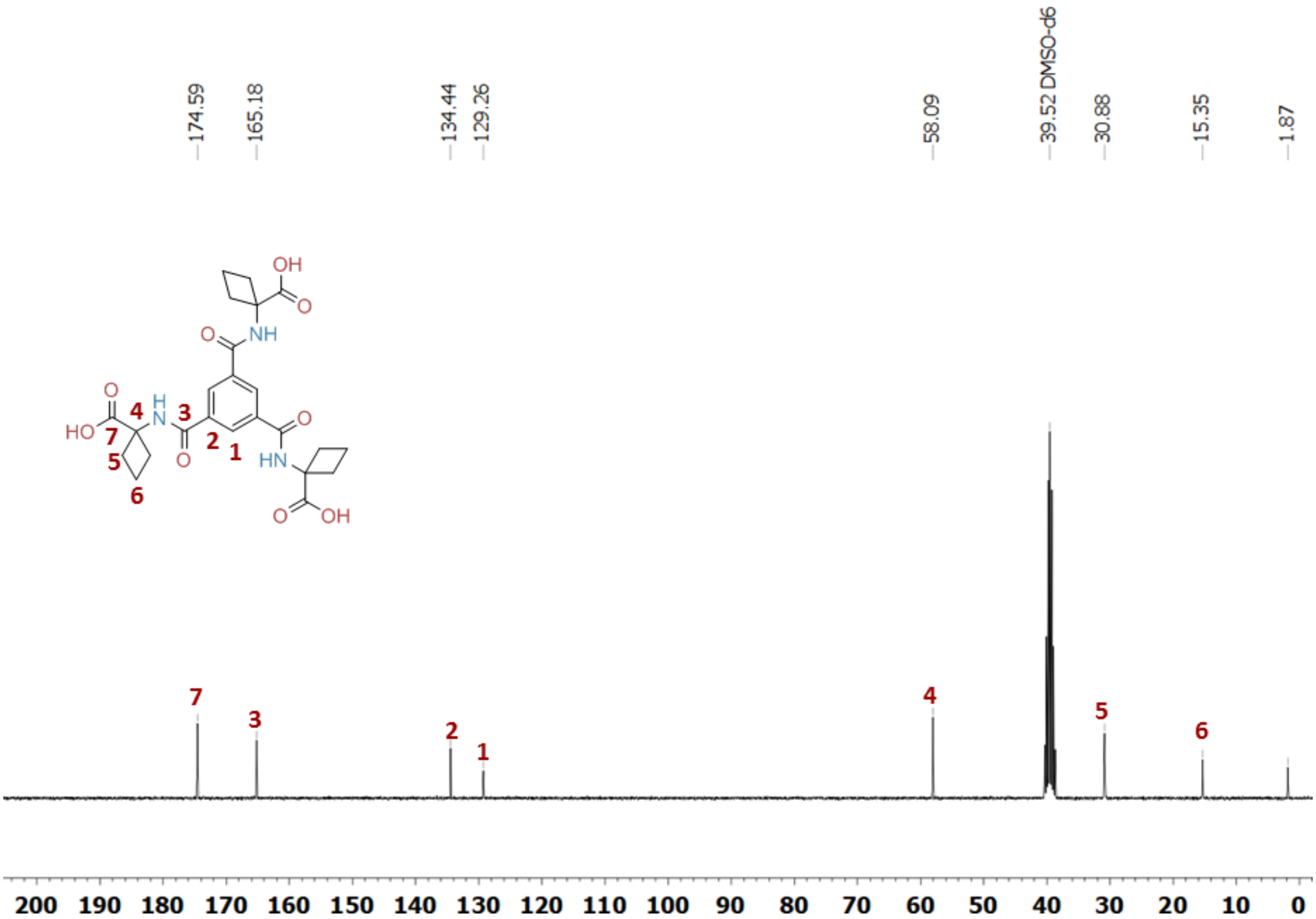

Figure S38. *^{13}C NMR (75 MHz DMSO-d_6) spectrum of* ***4***.

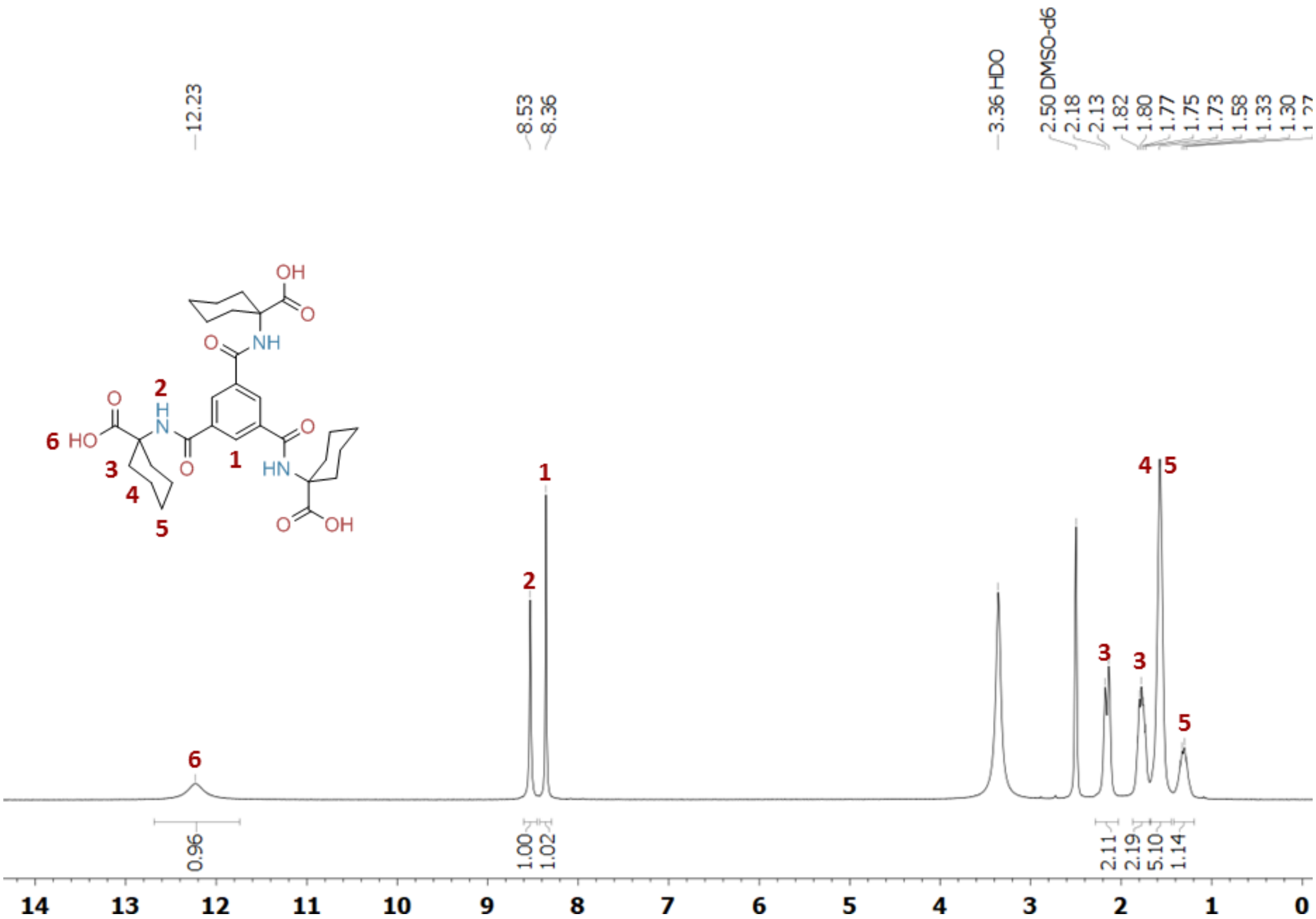

Figure S39. *^{1}H NMR (300 MHz DMSO-d_6) spectrum of* ***5***.

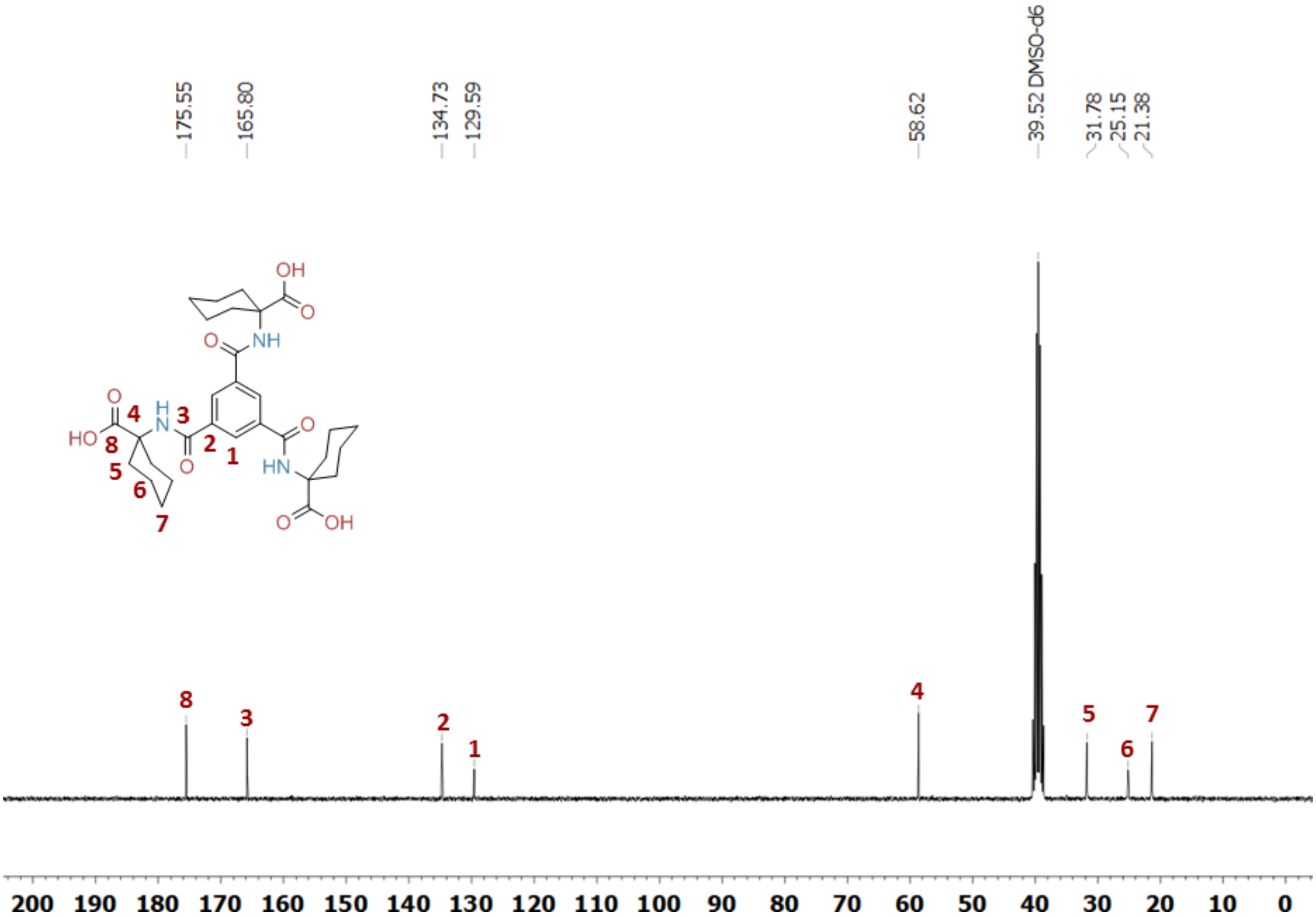

Figure S40. *^{13}C NMR (75 MHz DMSO-d_6) spectrum of* ***5***.

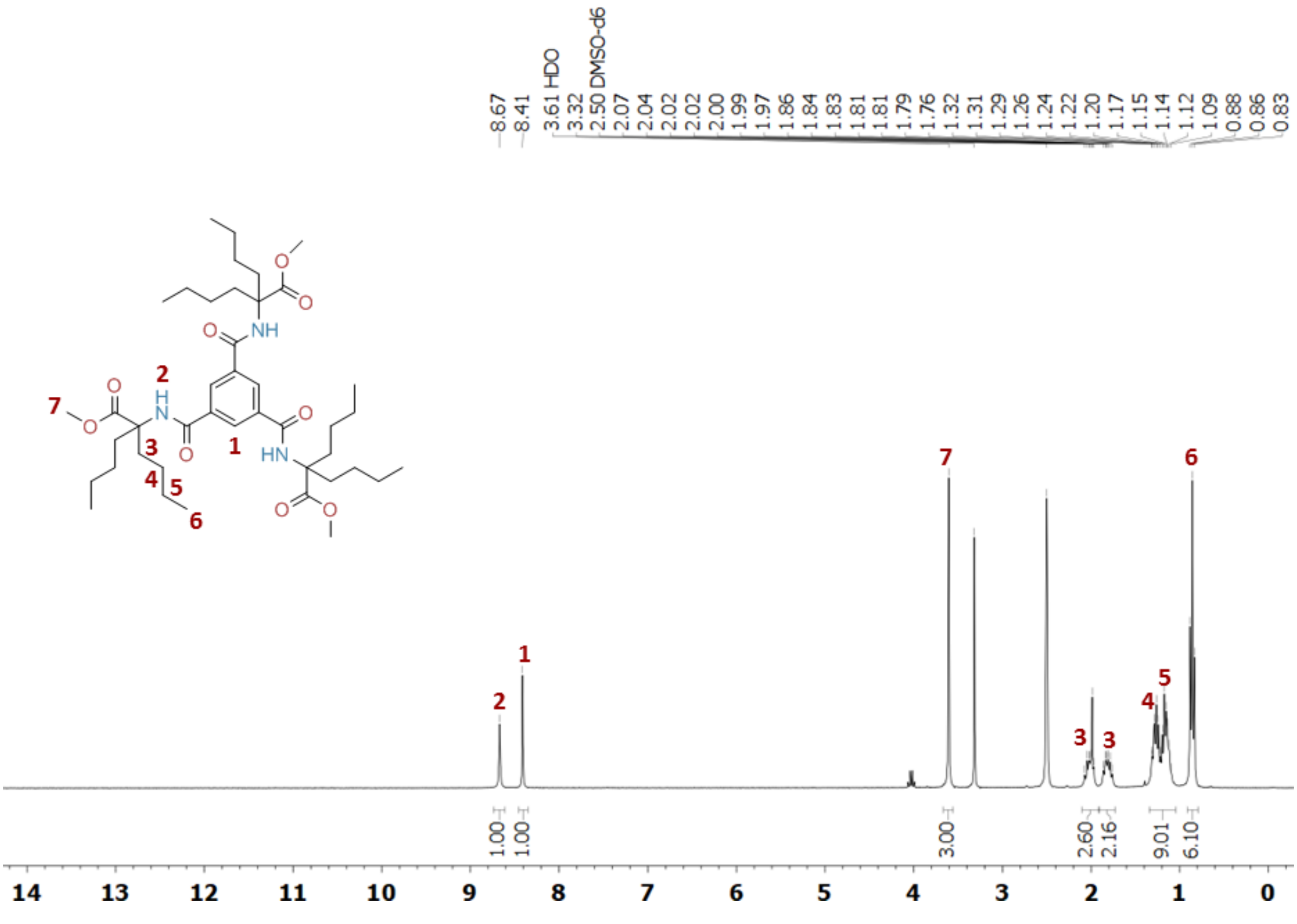

Figure S41. *¹H NMR (300 MHz DMSO-d₆) spectrum of* ***6-OMe.***

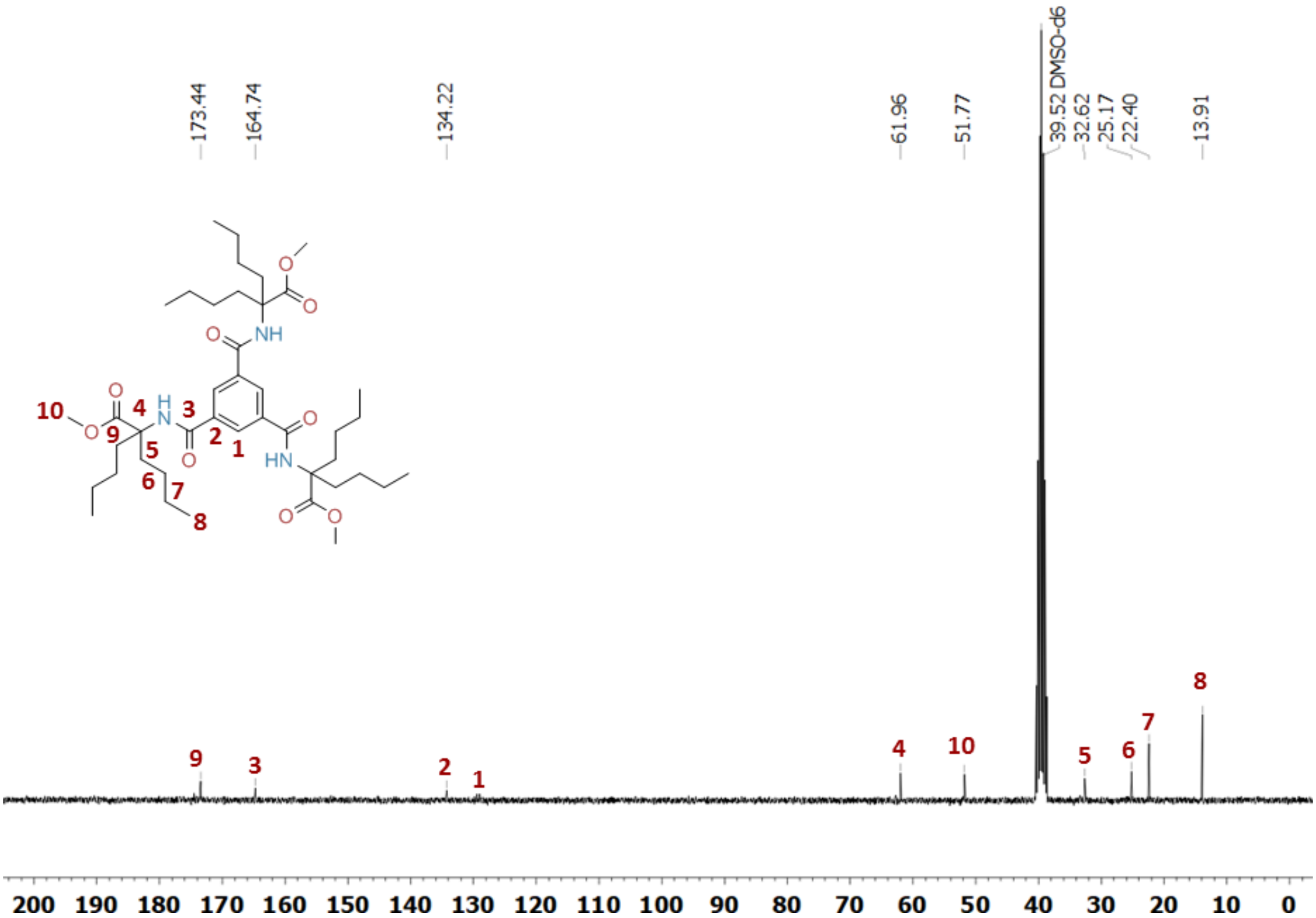

Figure S42. *¹³C NMR (75 MHz DMSO-d₆) spectrum of* ***6-OMe.***

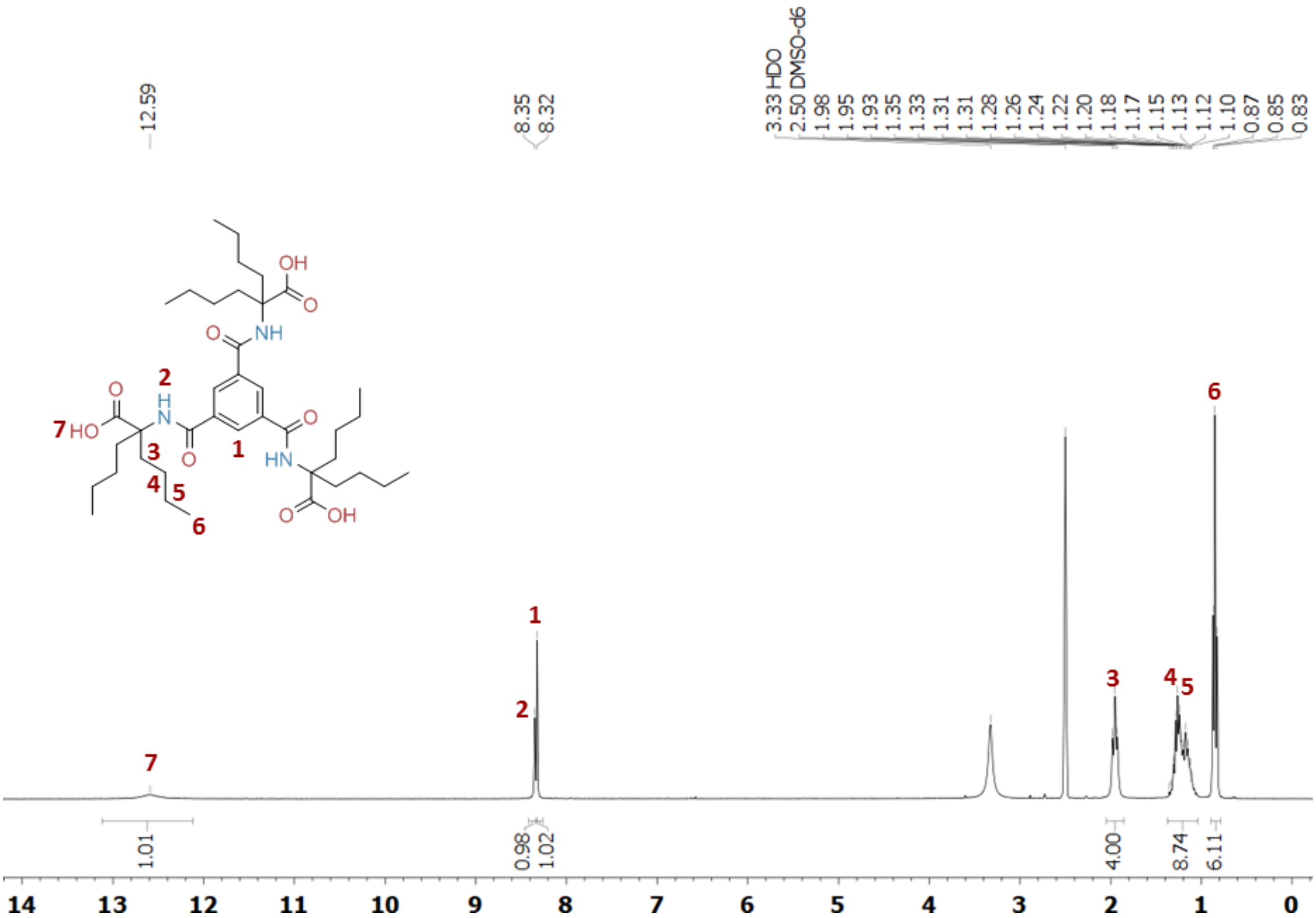

Figure S43. *[1] H NMR (300 MHz DMSO-d$_6$) spectrum of* ***6.***

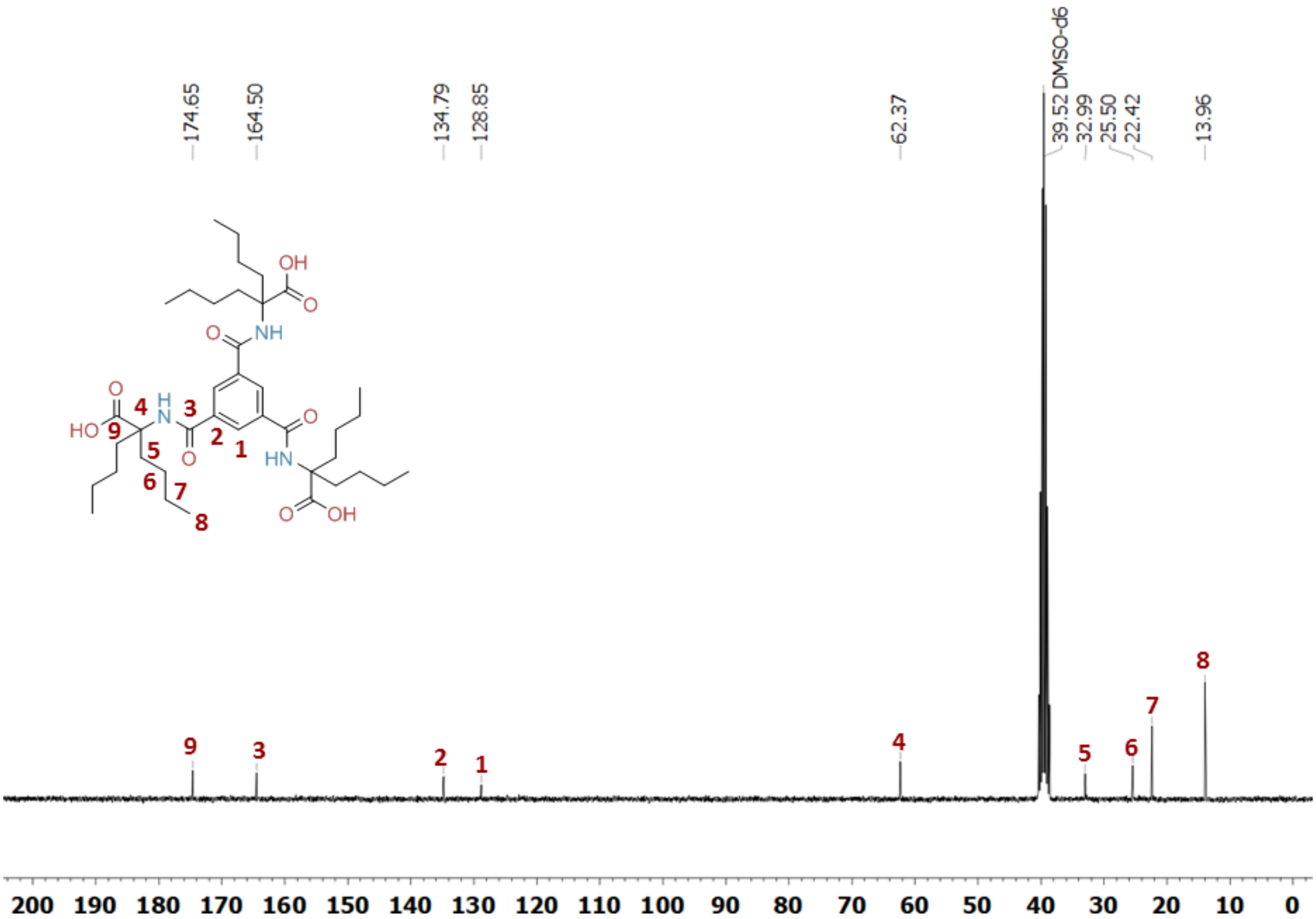

Figure S44. *[13] C NMR (75 MHz DMSO-d$_6$) spectrum of* ***6.***

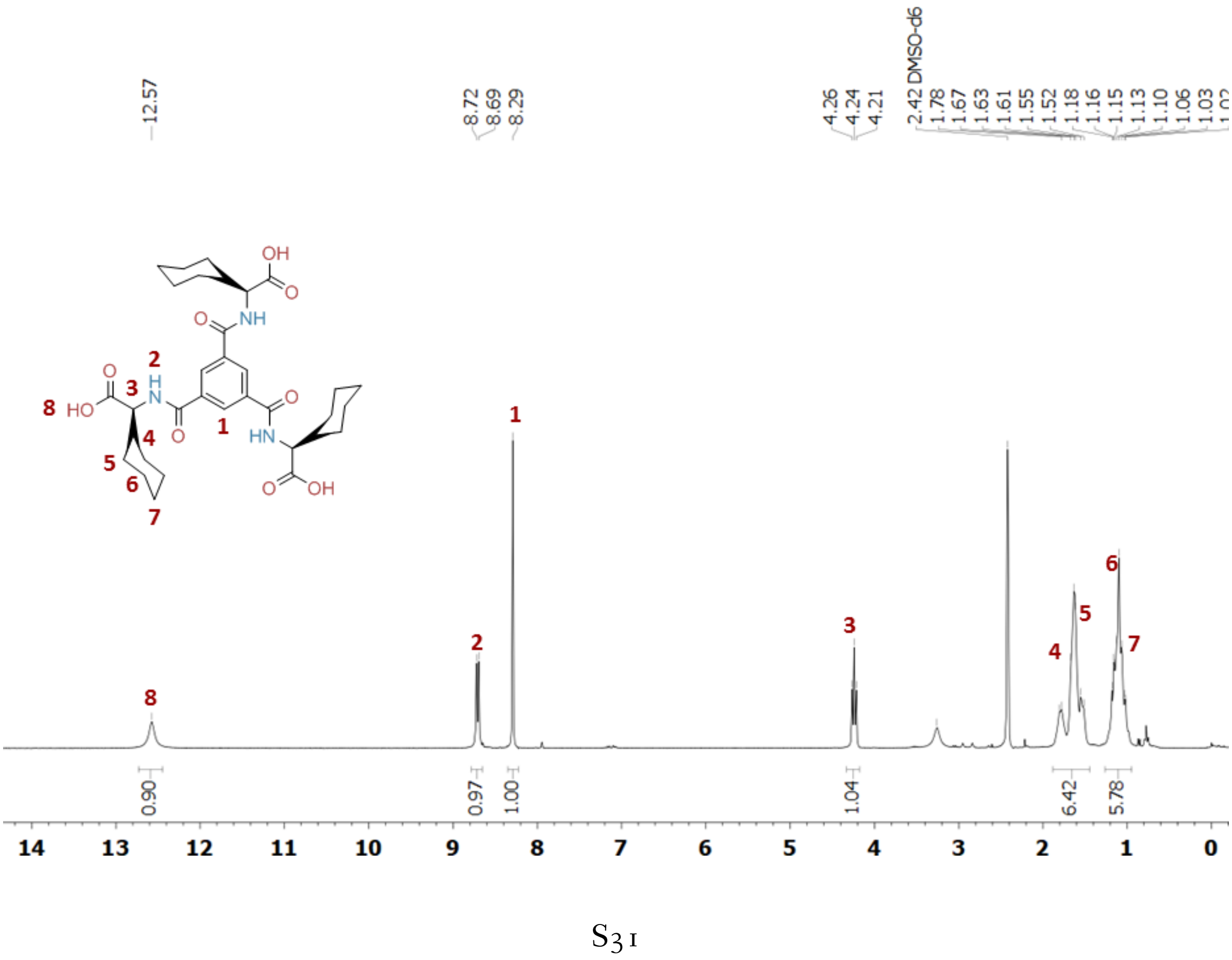

Figure S45. ^{1}H NMR (300 MHz DMSO-d_6) spectrum of **7**.

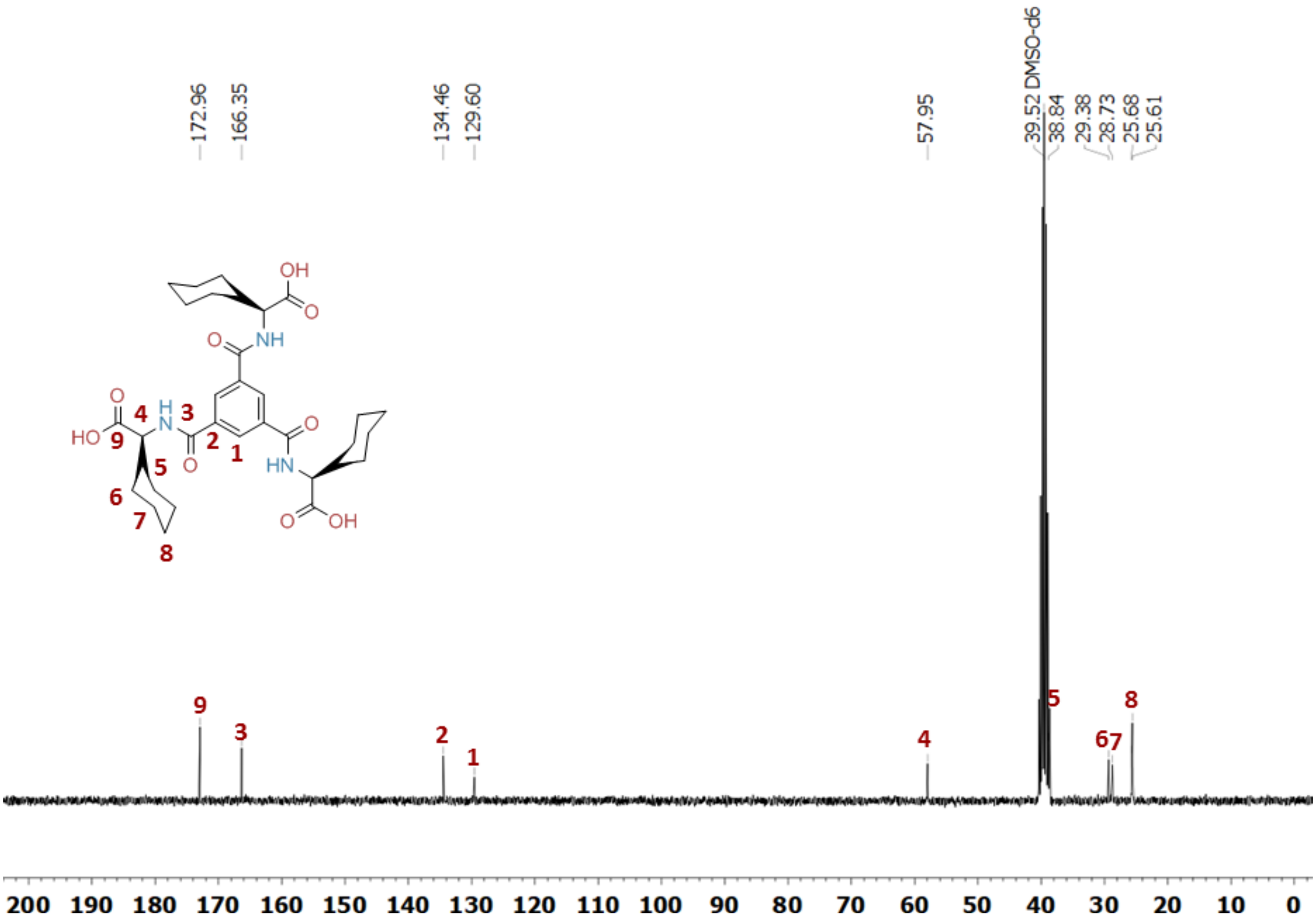

Figure S46. ^{13}C NMR (75 MHz DMSO-d_6) spectrum of **7**.

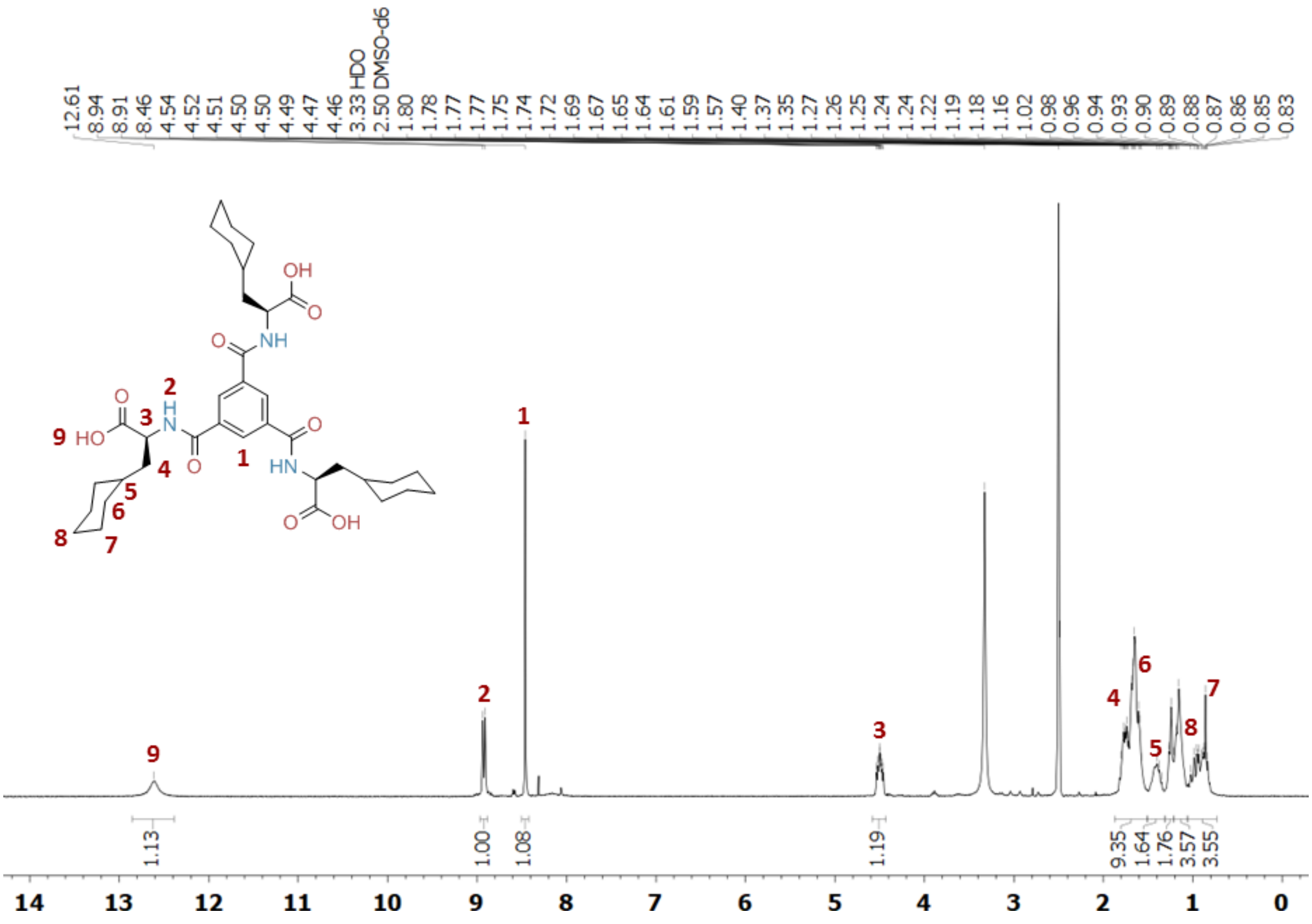

Figure S47. ^{1}H NMR (300 MHz DMSO-d_6) spectrum of **8**.

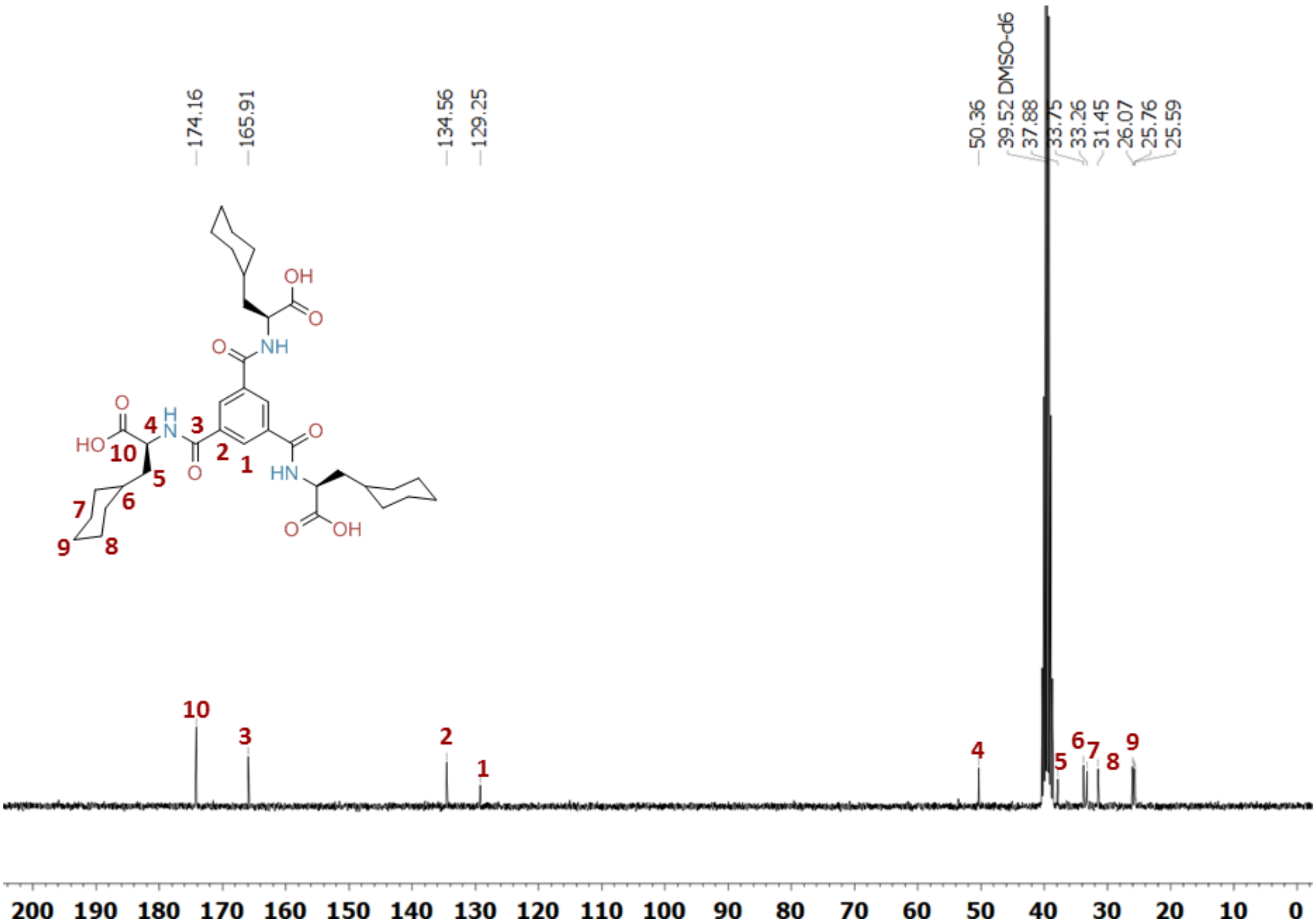

Figure S48. ^{13}C NMR (75 MHz DMSO-d_6) spectrum of **8**.

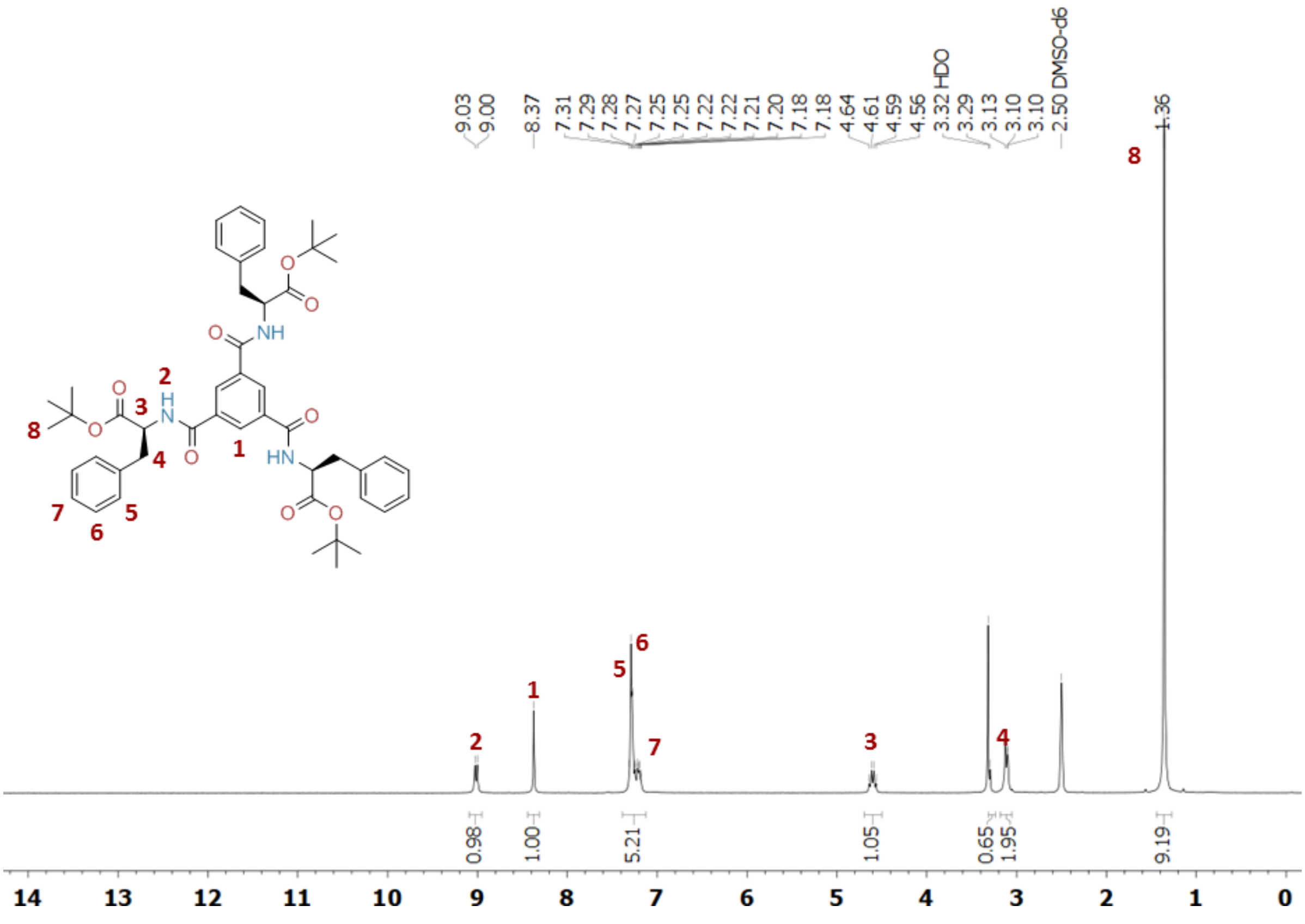

Figure S49. ^{1}H NMR (300 MHz DMSO-d_6) spectrum of **9-tBu.**

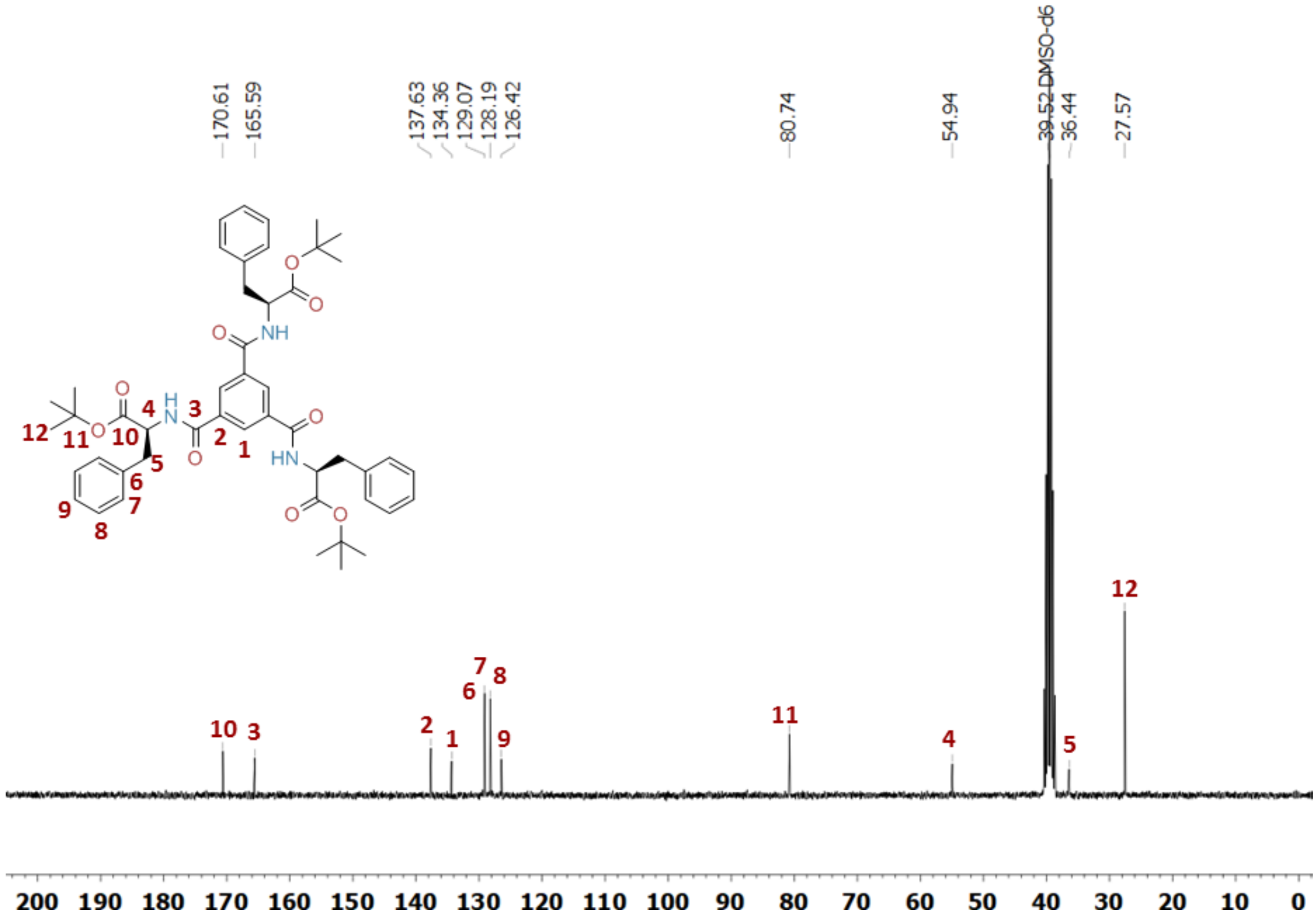

Figure S50. ^{13}C NMR (75 MHz DMSO-d_6) spectrum of **9-tBu.**

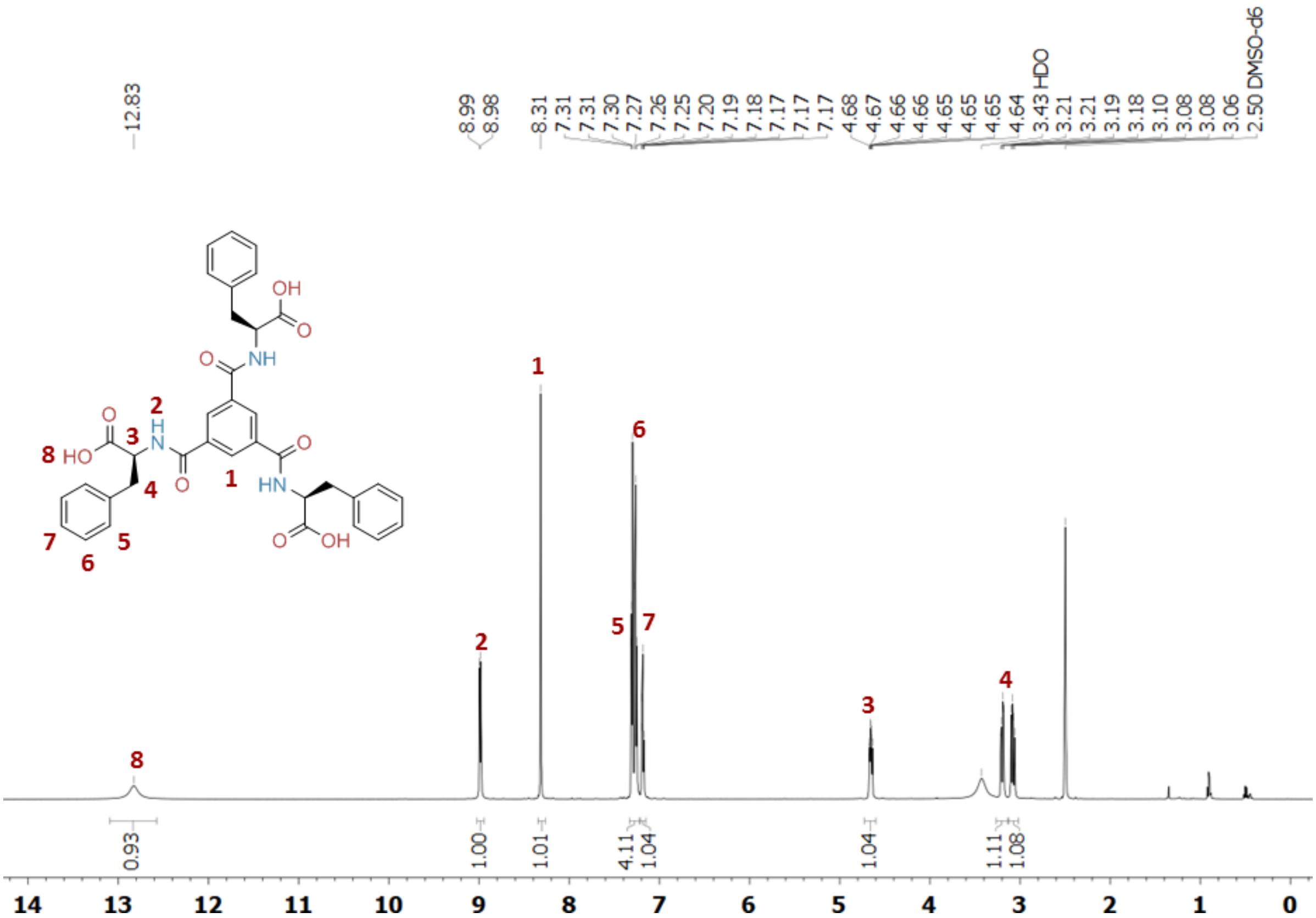

Figure S51. ^{1}H NMR (600 MHz DMSO-d_6) spectrum of **9.**

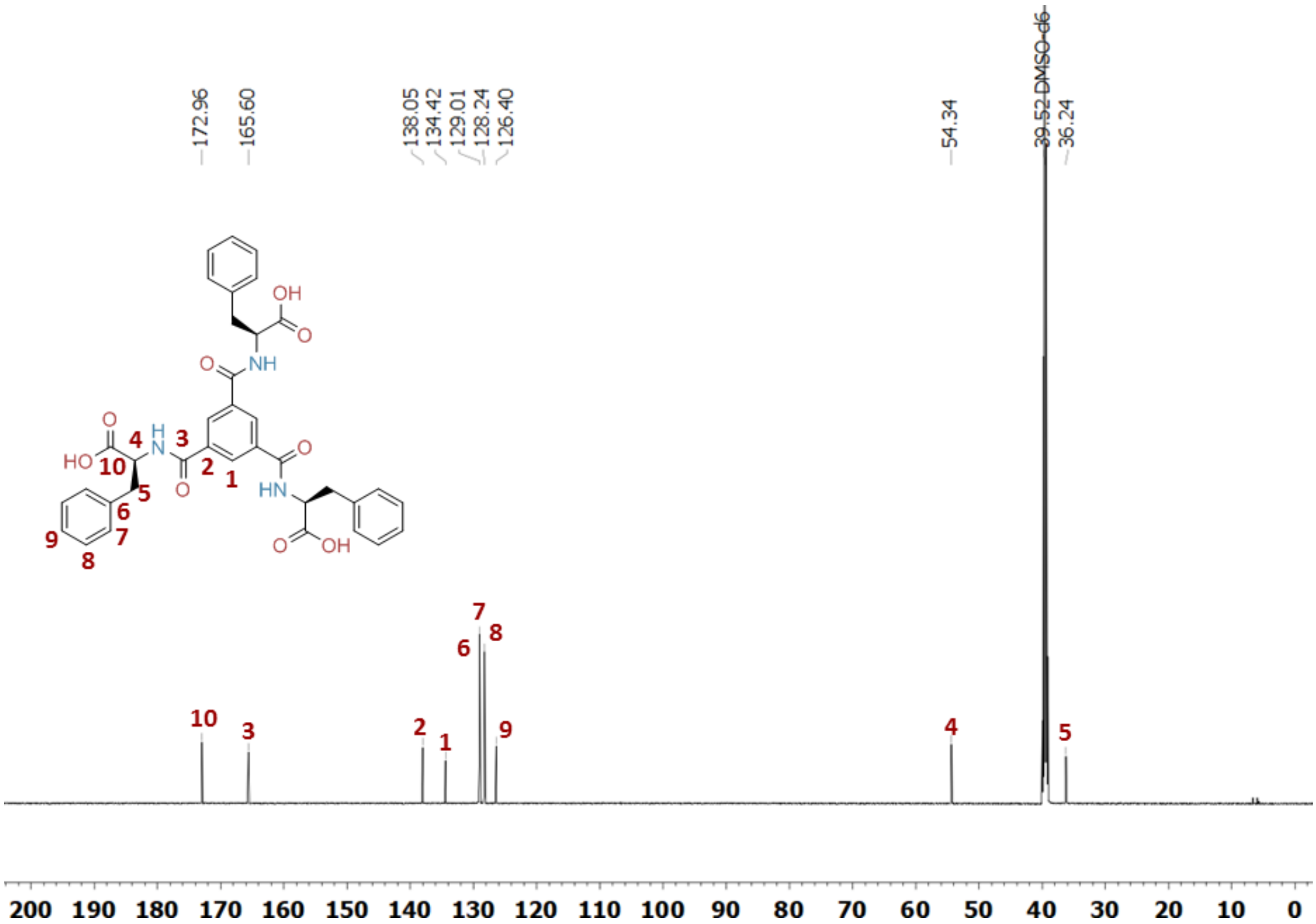

Figure S52. *^{13}C NMR (151 MHz DMSO-d_6) spectrum of **9**.*

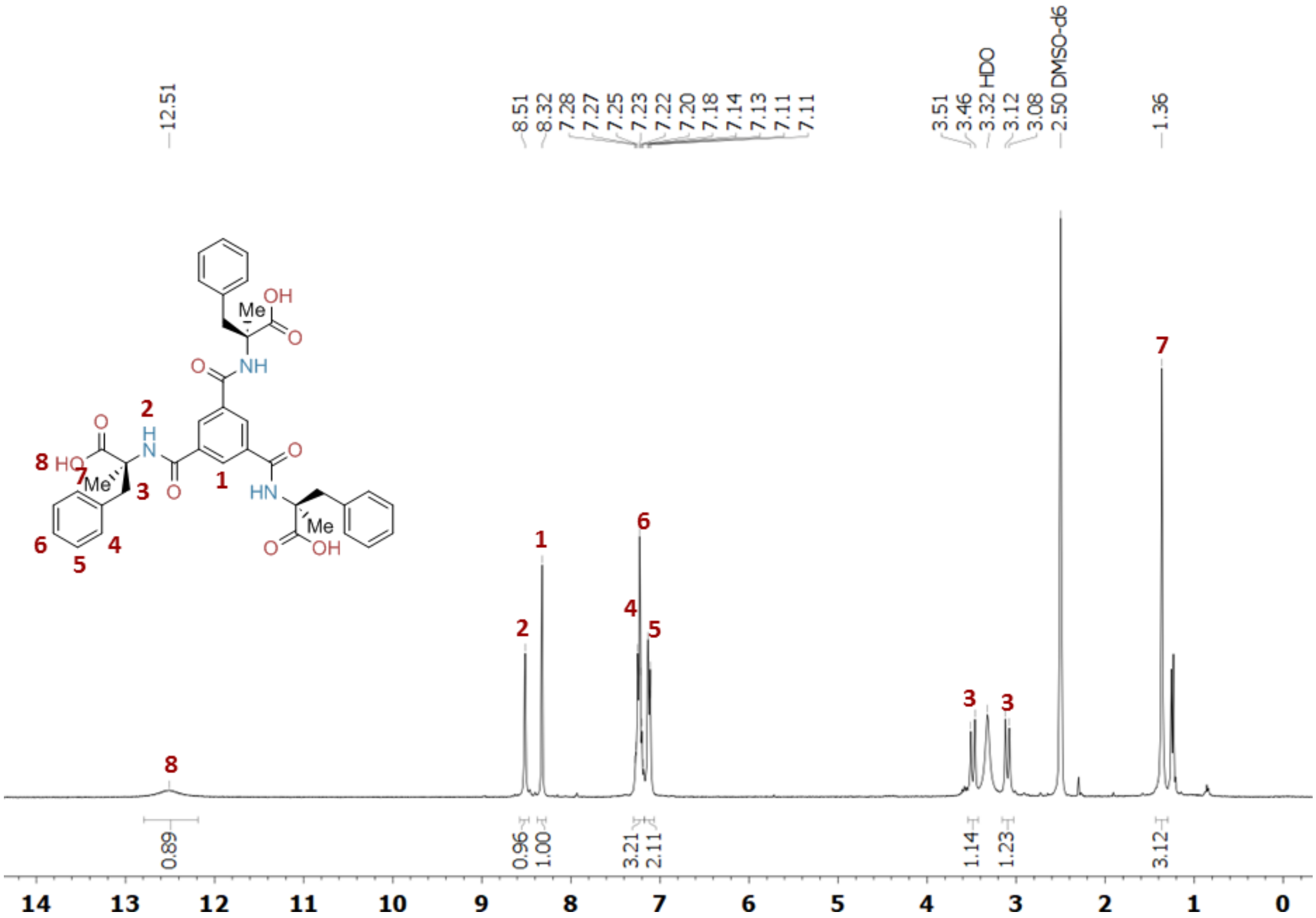

Figure S53. *^{1}H NMR (600 MHz DMSO-d_6) spectrum of **10**.*

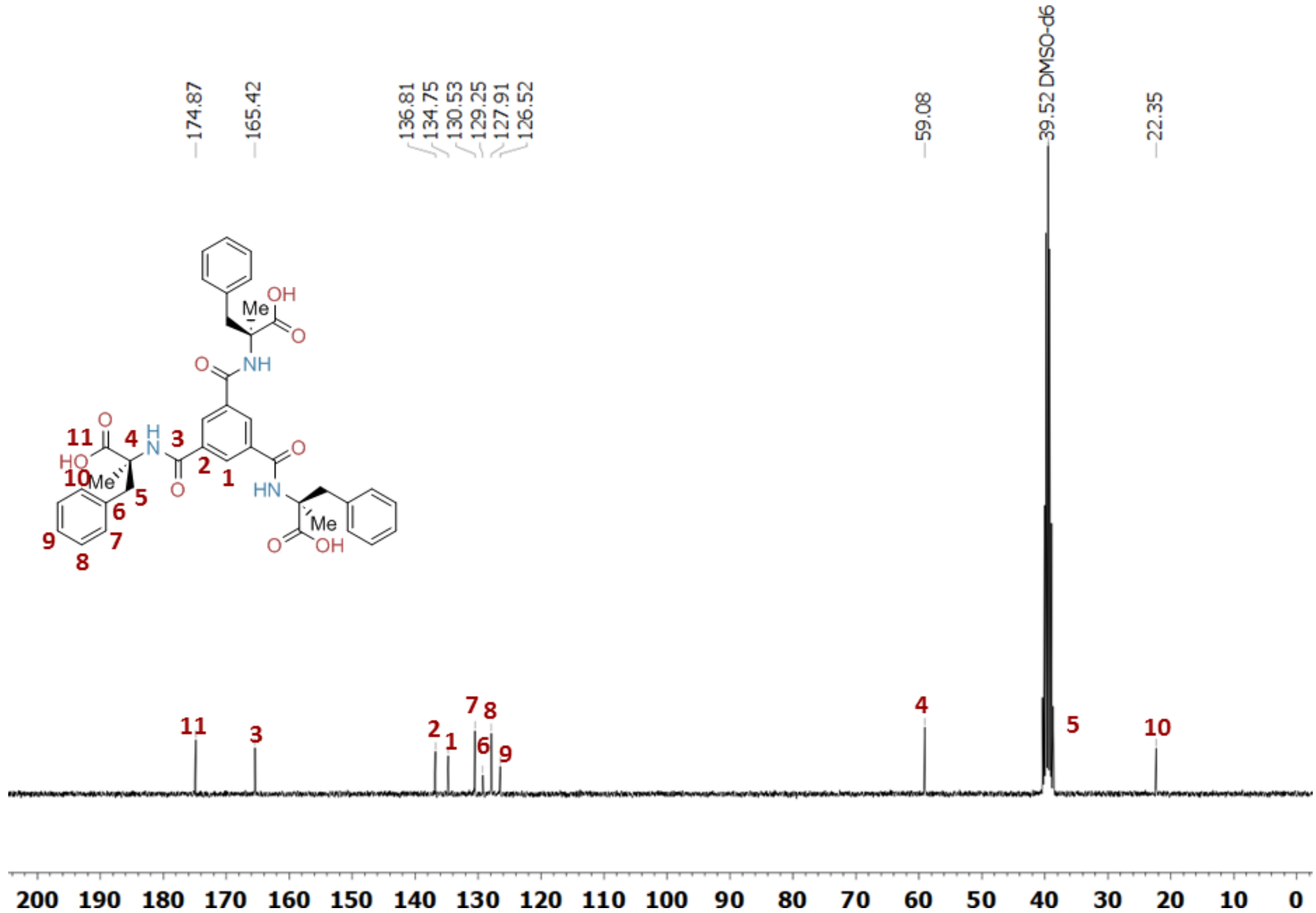

Figure S54. ^{13}C NMR (75 MHz DMSO-d_6) spectrum of **10**.

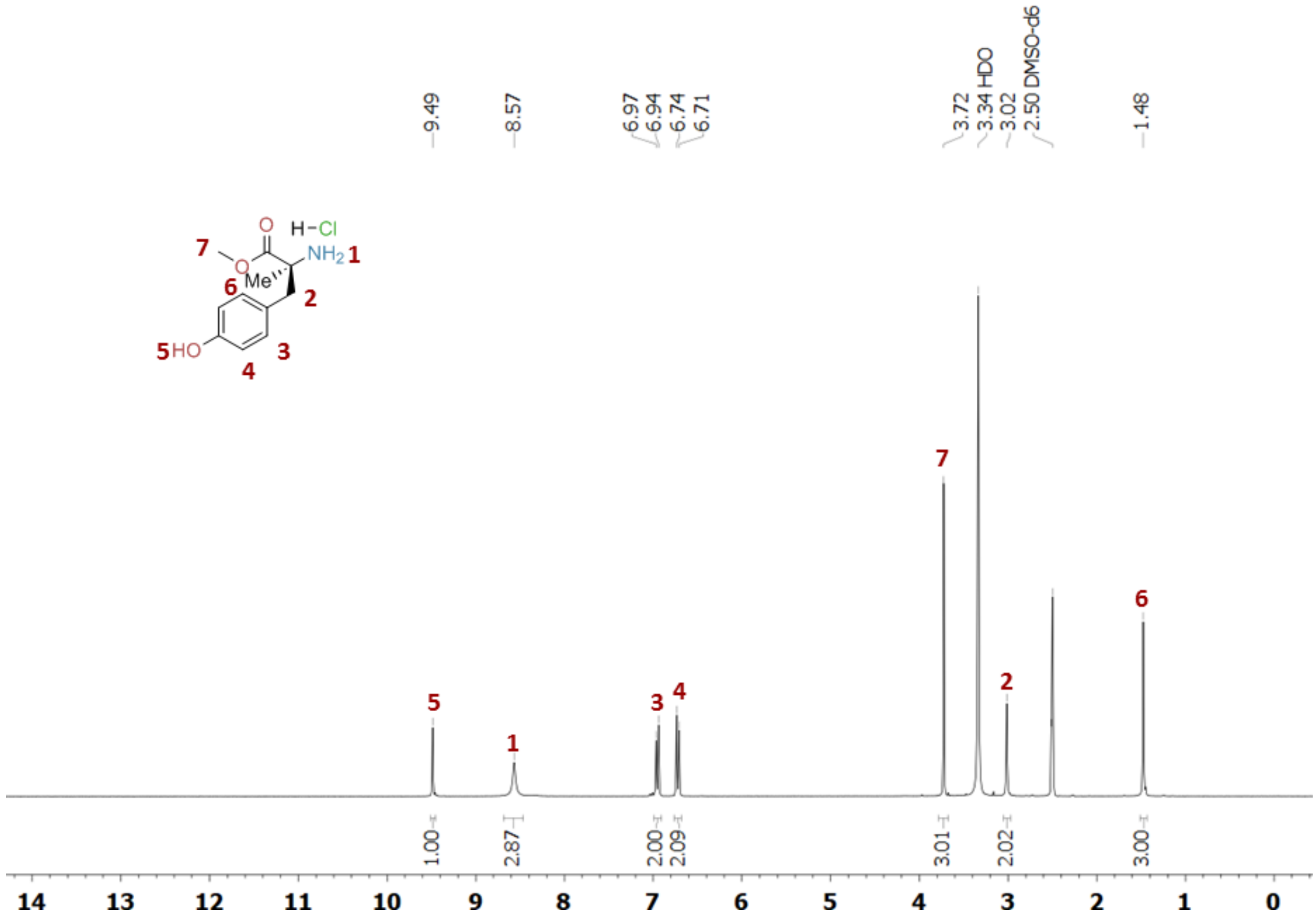

Figure S55. ^{1}H NMR (300 MHz DMSO-d_6) spectrum of **□-Me_L-Tyr_ OMe_HCl.**

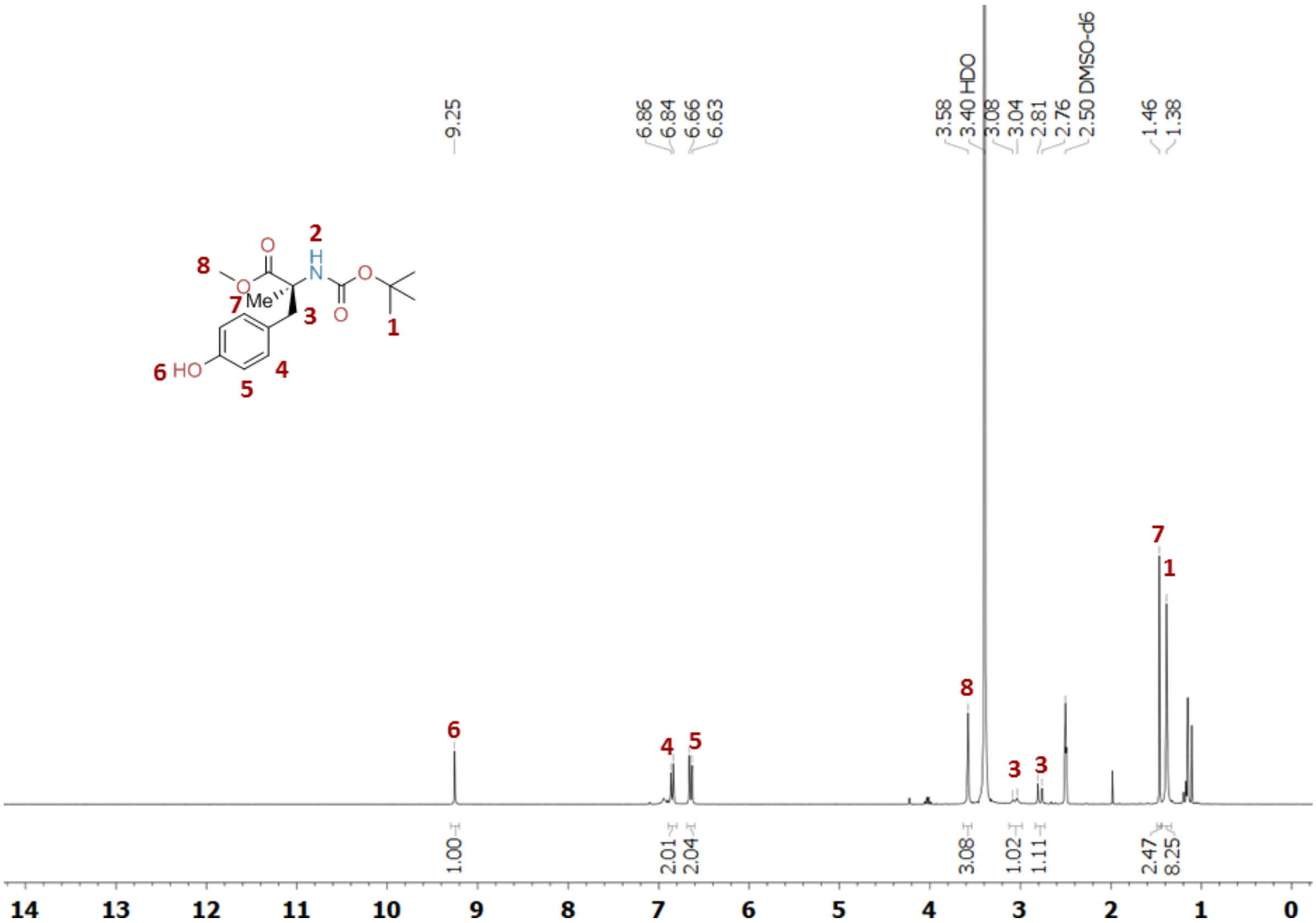

Figure S56. ¹H NMR (300 MHz DMSO-d_6) spectrum of **□-Me_L-Tyr_ OMe_Boc.**

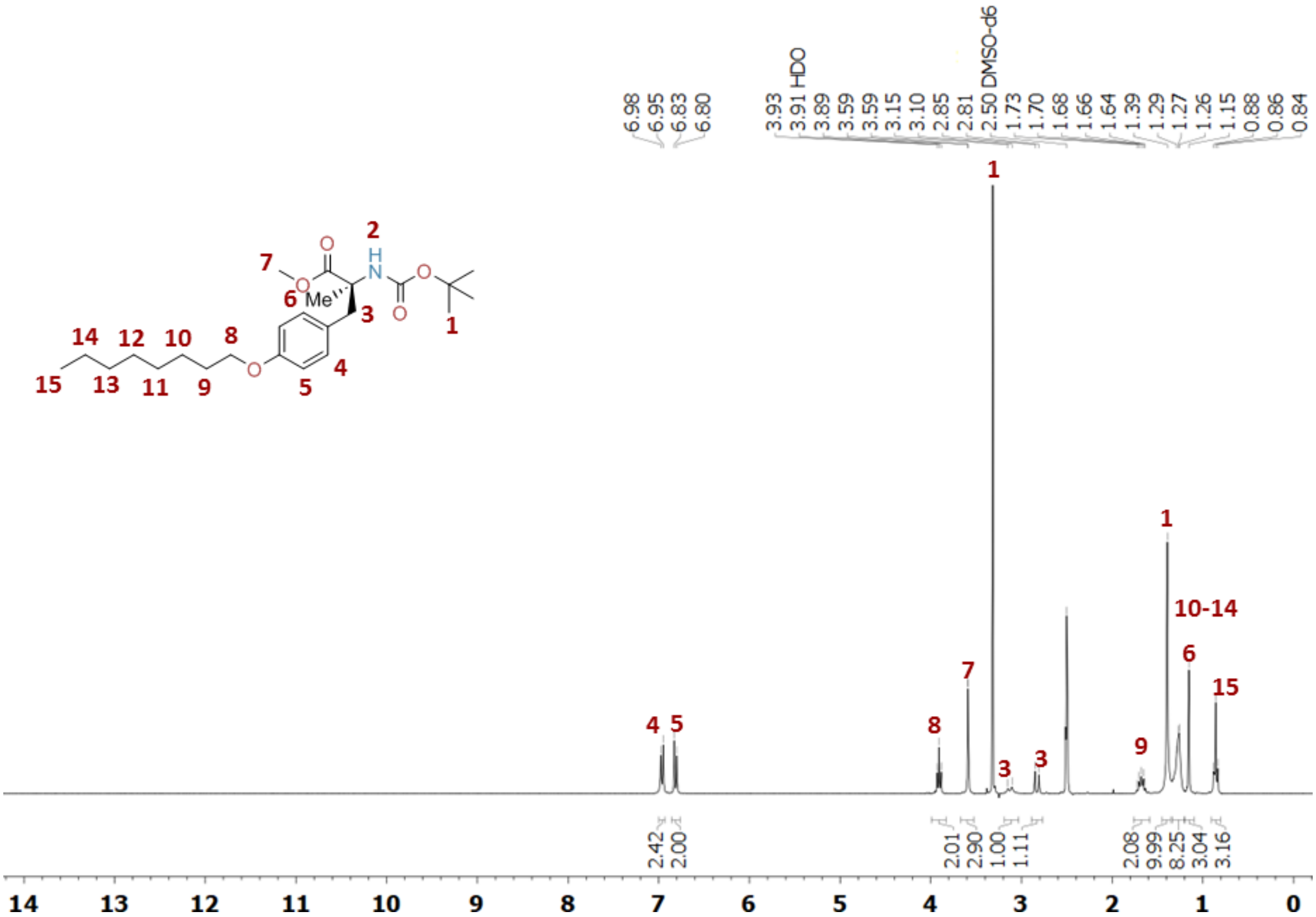

Figure S57. ¹H NMR (300 MHz DMSO-d_6) spectrum of **□-Me_L-Tyr_ OMe_Boc_C8.**

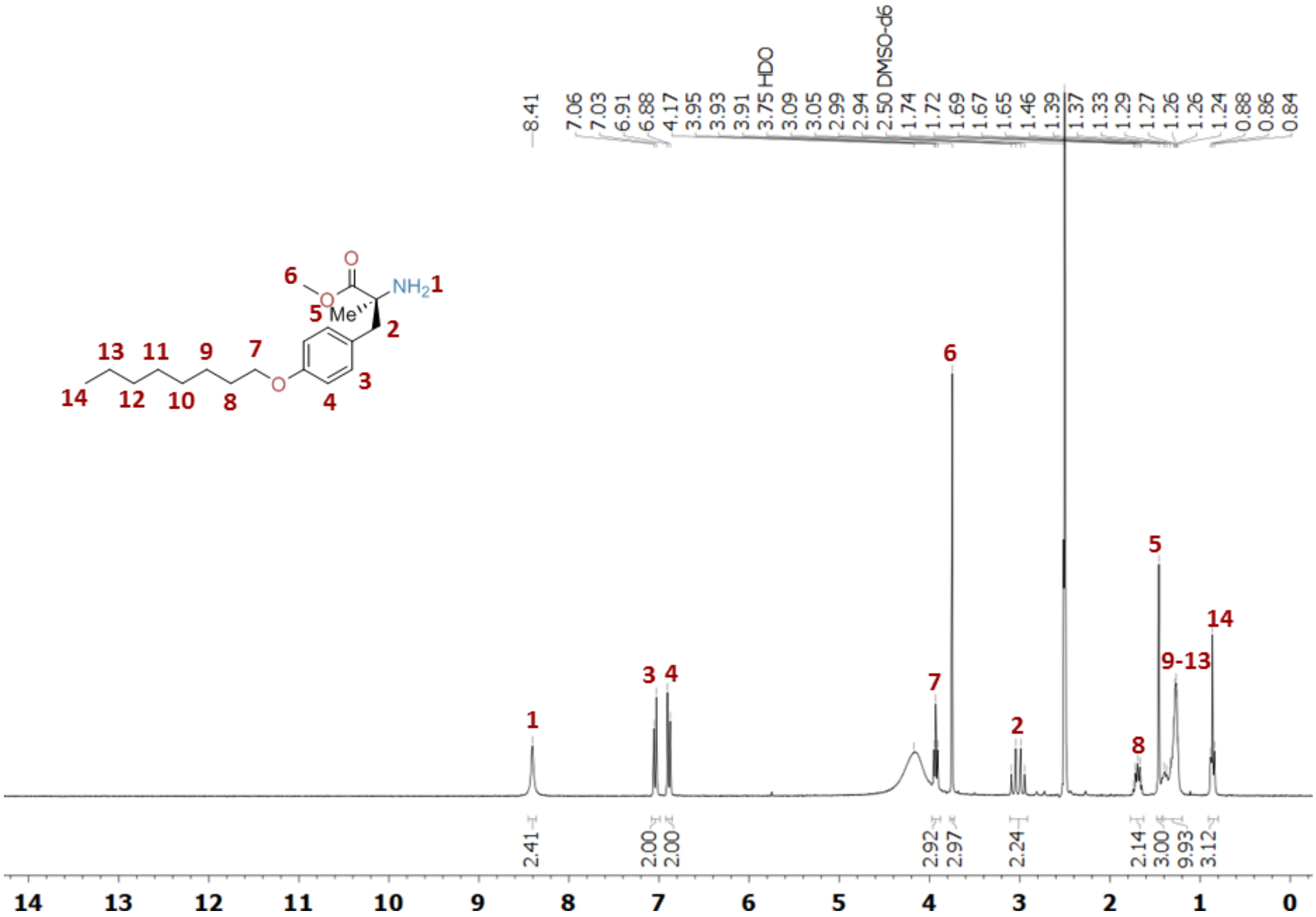

Figure S58. ^{1}H NMR (300 MHz DMSO-d_6) spectrum of ***□-Me_L-Tyr_ OMe_C8.***

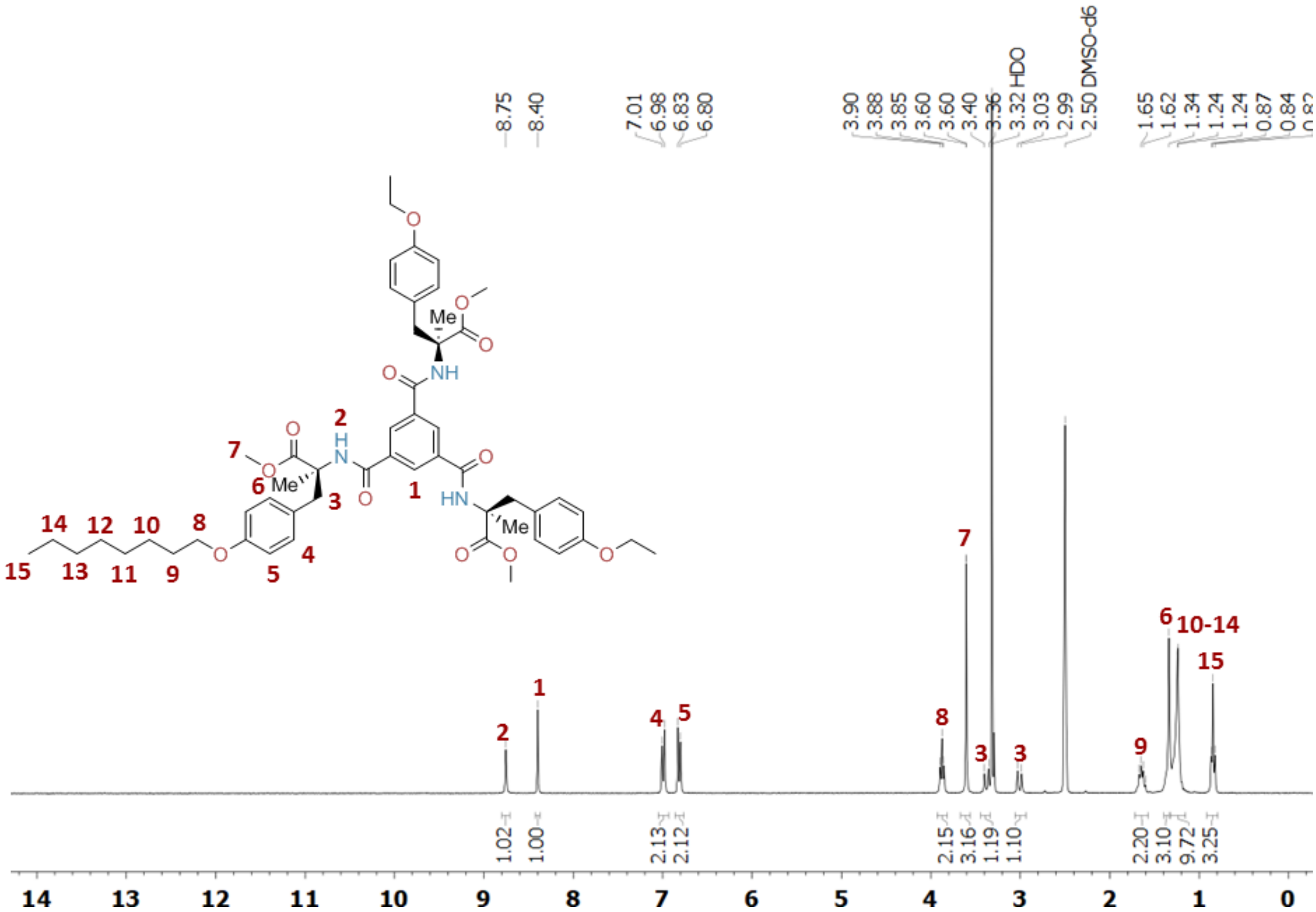

Figure S59. ^{1}H NMR (300 MHz DMSO-d_6) spectrum of ***11-OMe.***

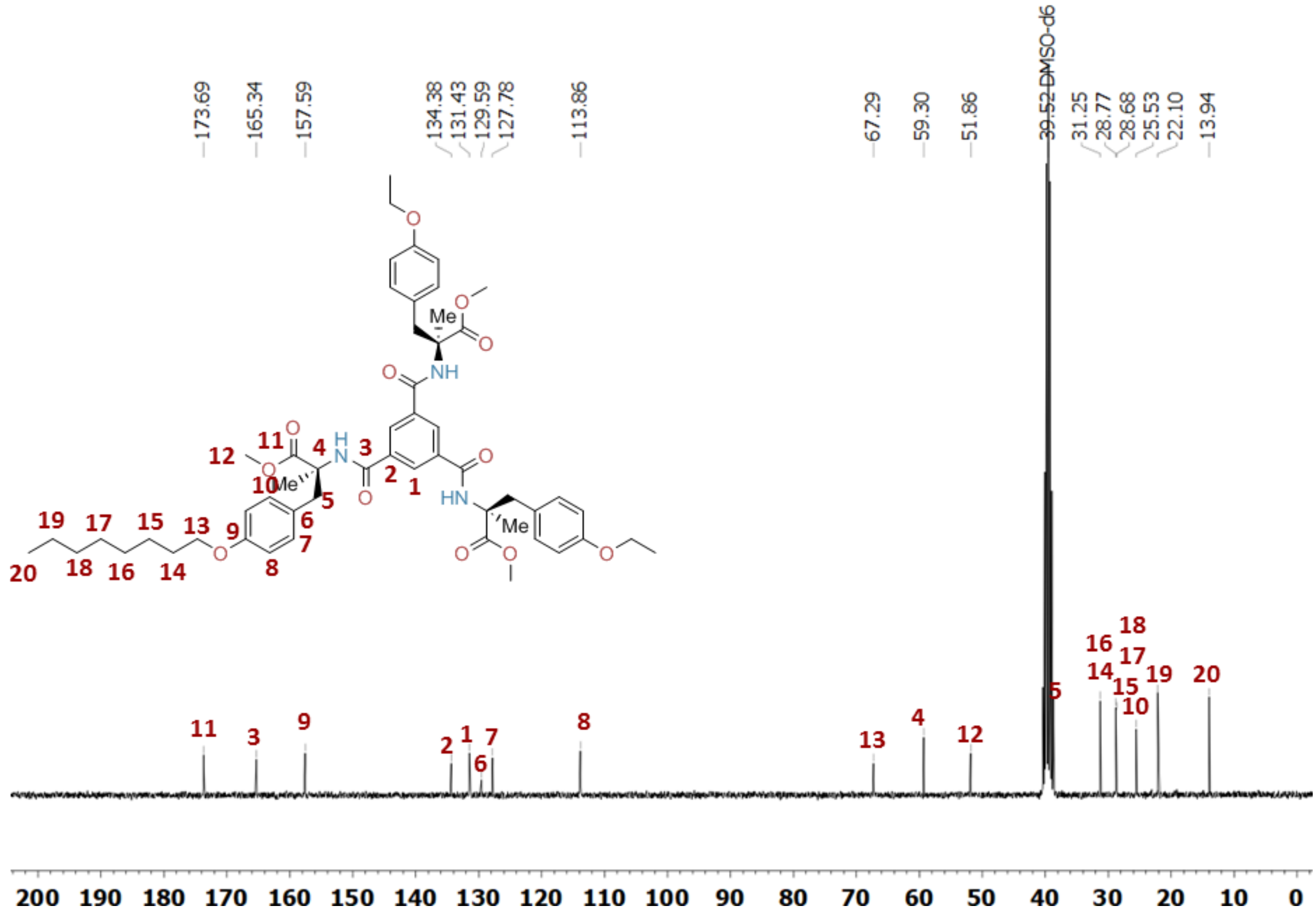

Figure S60. ^{13}C NMR (75 MHz DMSO-d_6) spectrum of **11-OMe.**

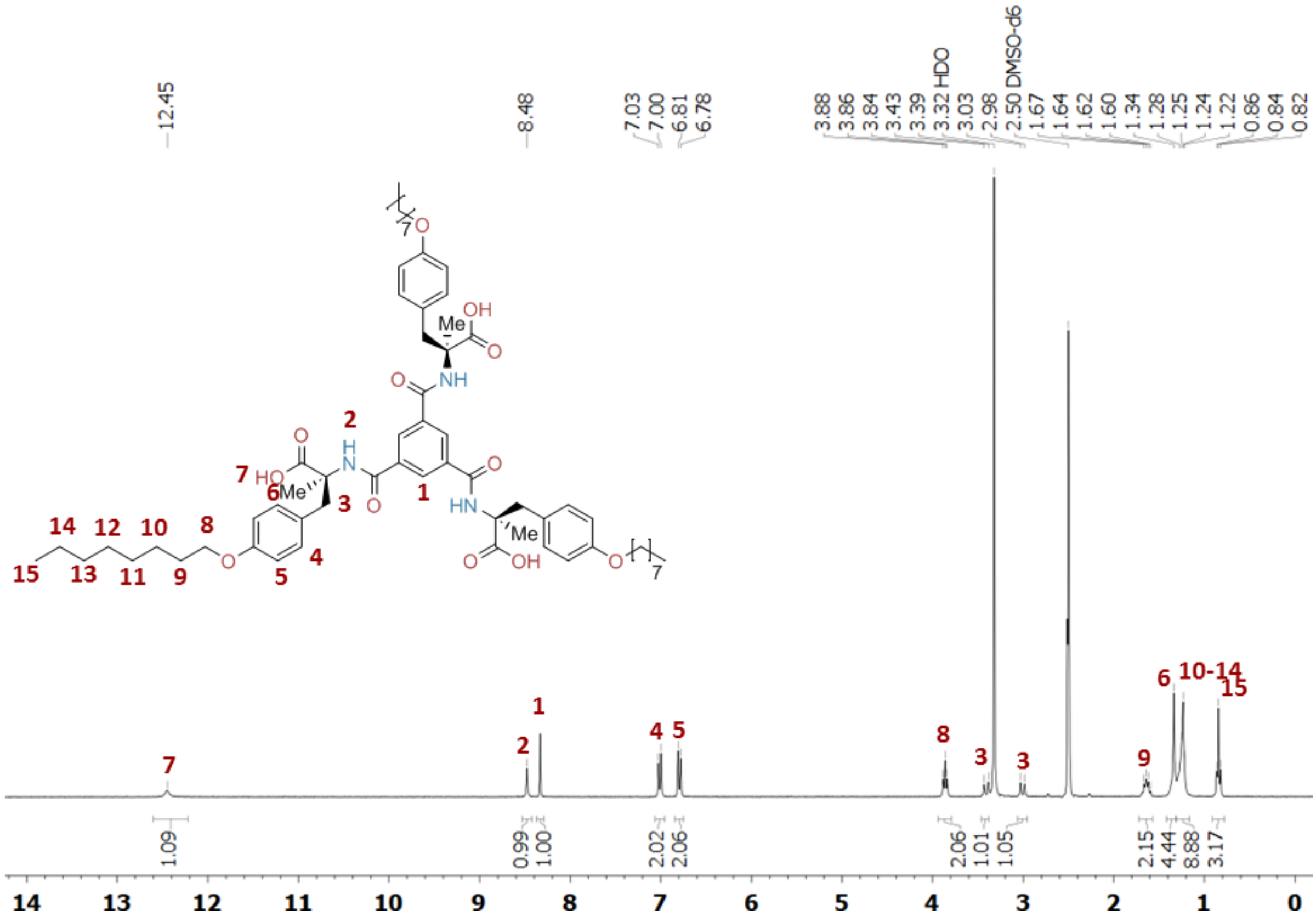

Figure S61. ^{1}H NMR (300 MHz DMSO-d_6) spectrum of **11.**

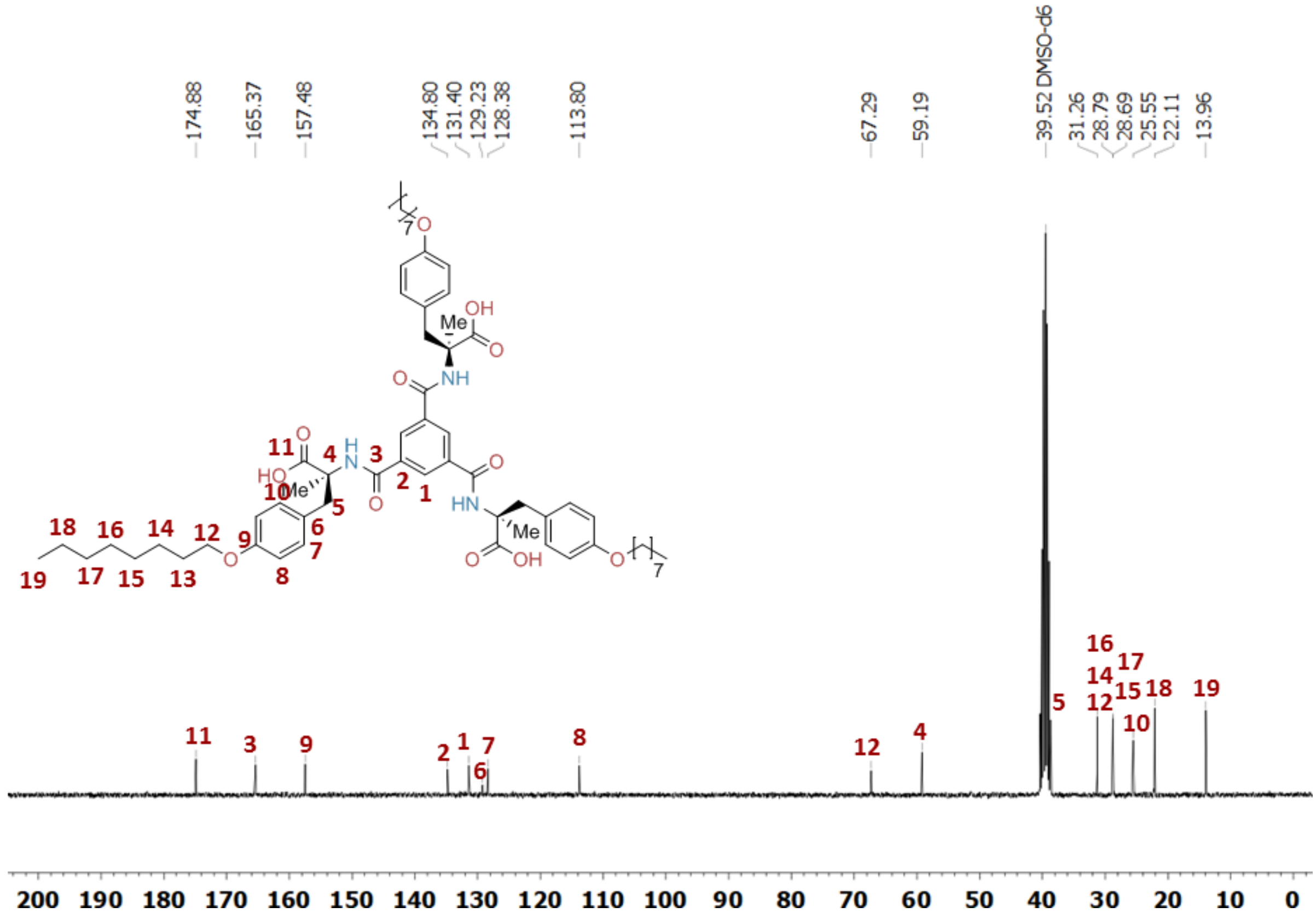

Figure S62. *^{13}C NMR (75 MHz DMSO-d_6) spectrum of* ***11.***

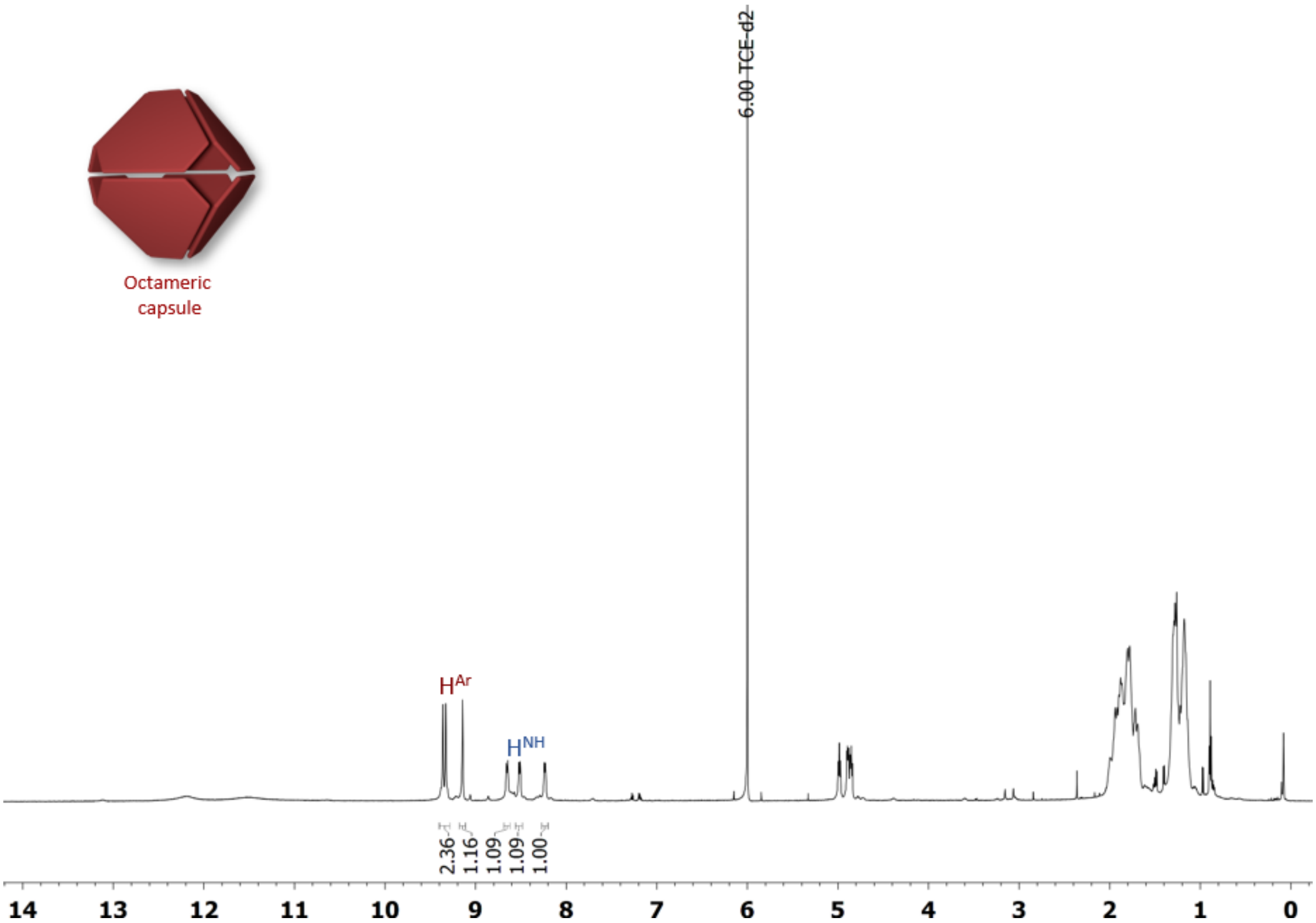

Figure S63. *^{1}H NMR (600 MHz TCE-d_2) spectrum of* ***7.***

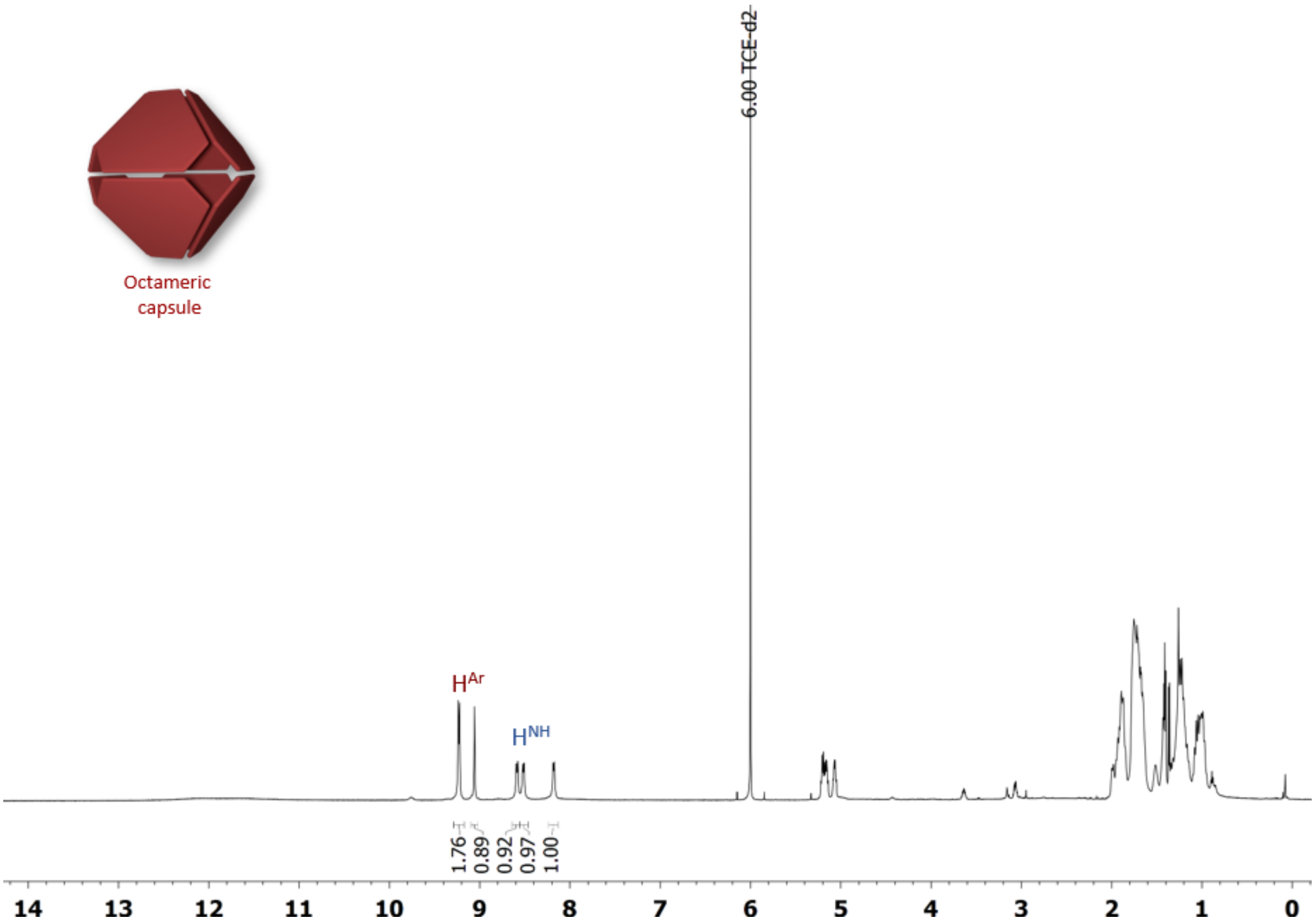

Figure S64. ^{1}H NMR (600 MHz TCE-d_2) spectrum of **8.**

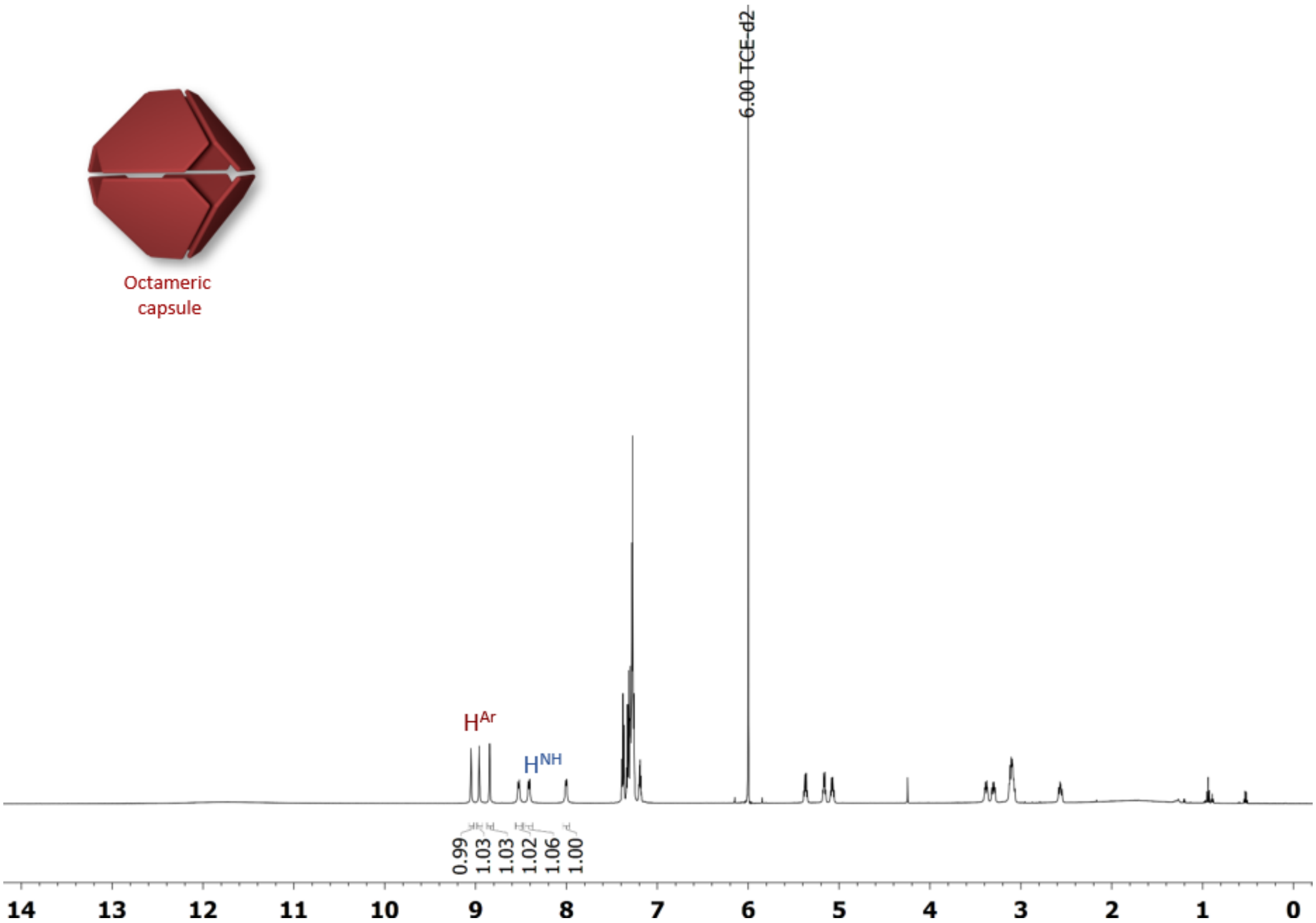

Figure S65. ^{1}H NMR (600 MHz TCE-d_2) spectrum of **9.**

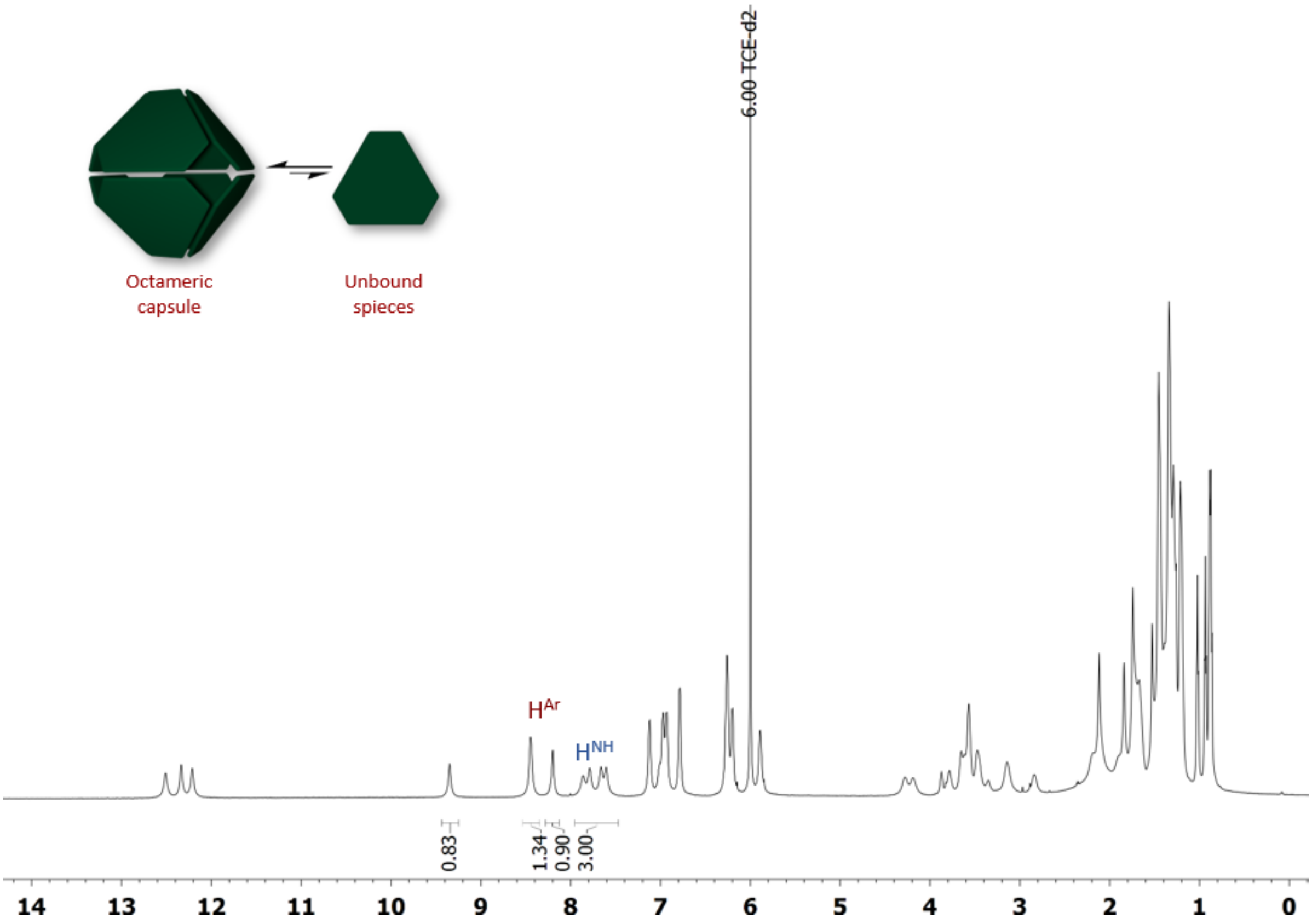

Figure S66. ^{1}H NMR (600 MHz TCE-d_2) spectrum of **11**.

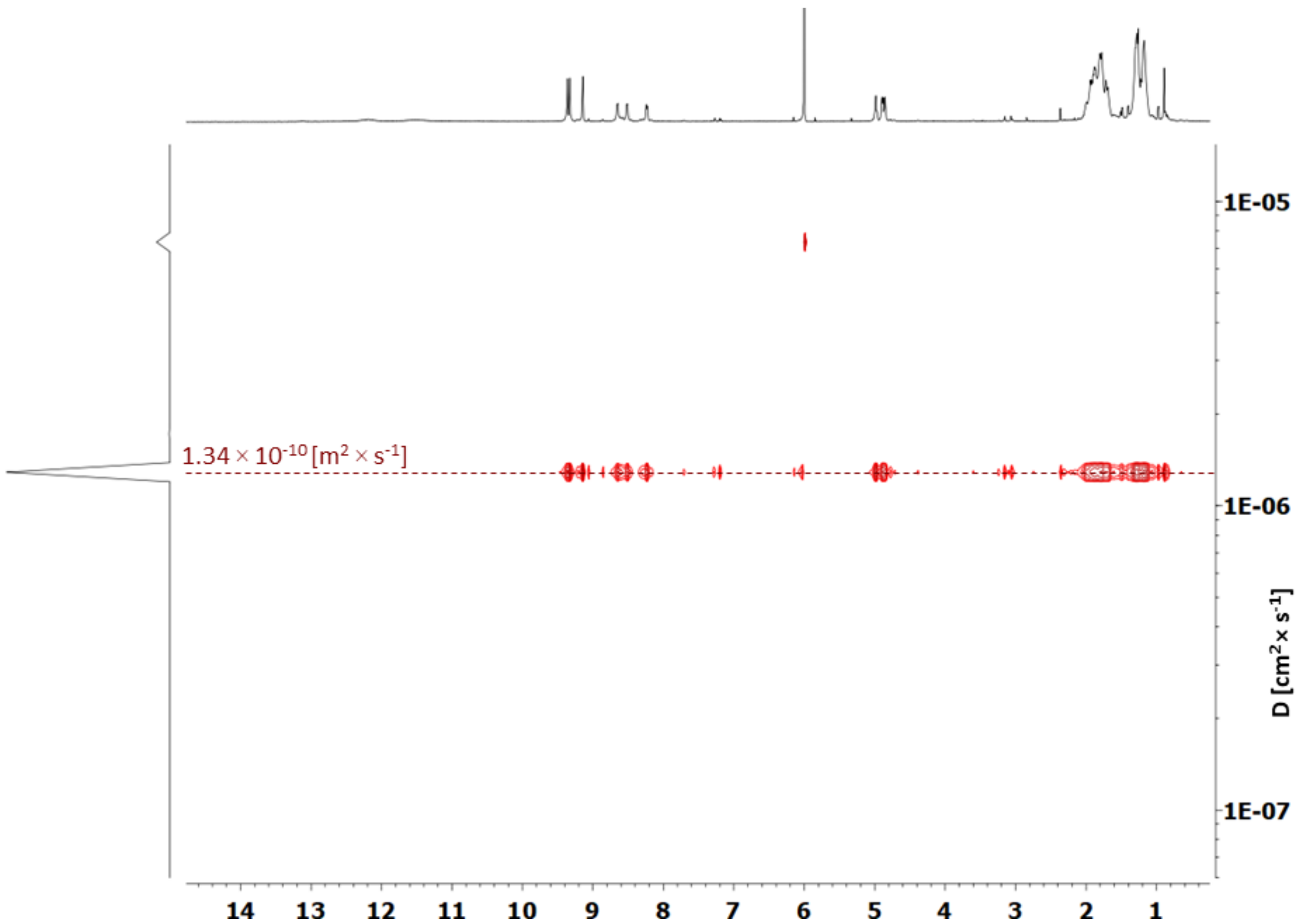

Figure S67. DOSY NMR (600 MHz TCE-d_2) spectrum of **7**.

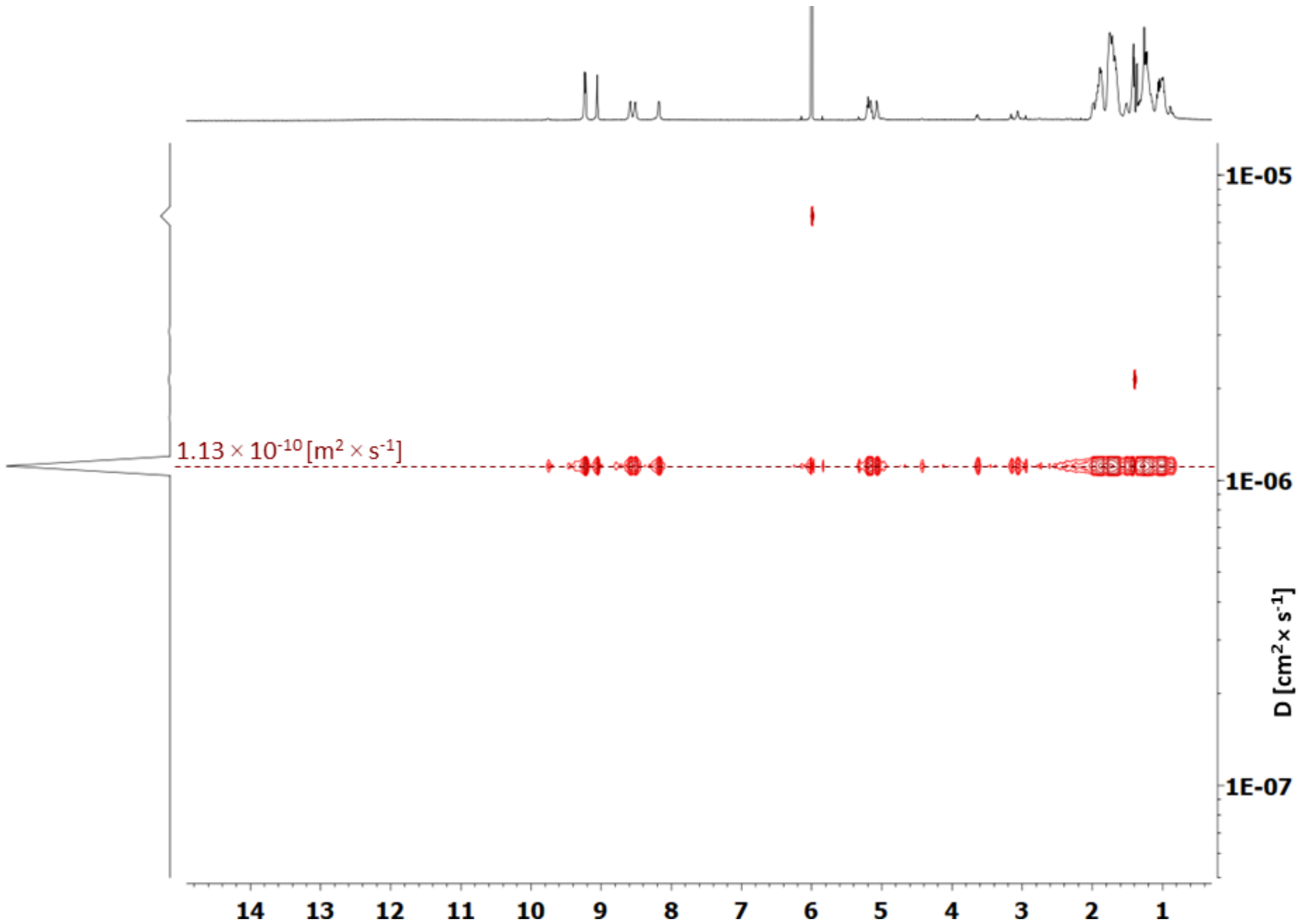

Figure S68. DOSY NMR (600 MHz TCE-d_2) spectrum of **8**.

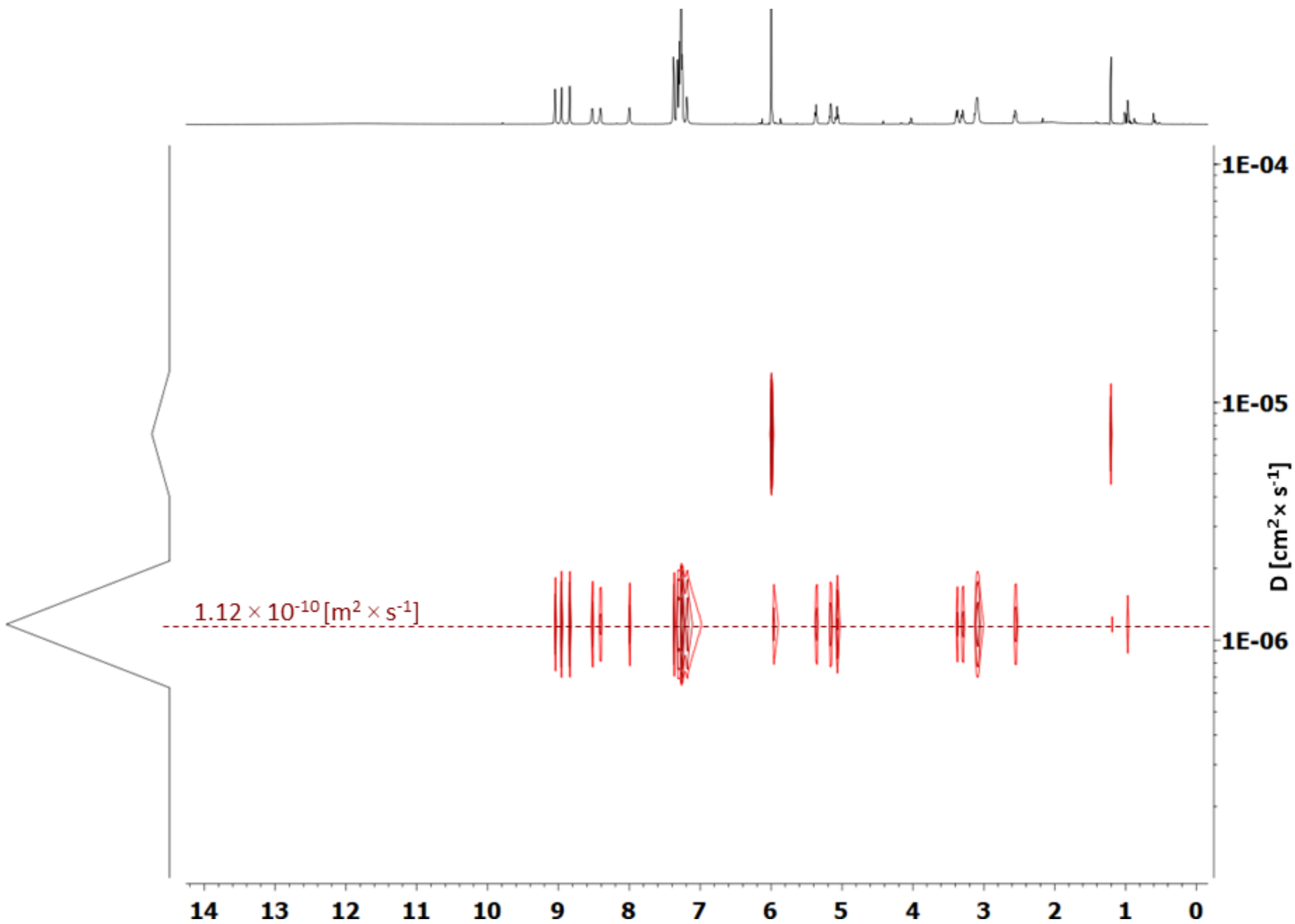

Figure S69. DOSY NMR (600 MHz TCE-d_2) spectrum of **9**.

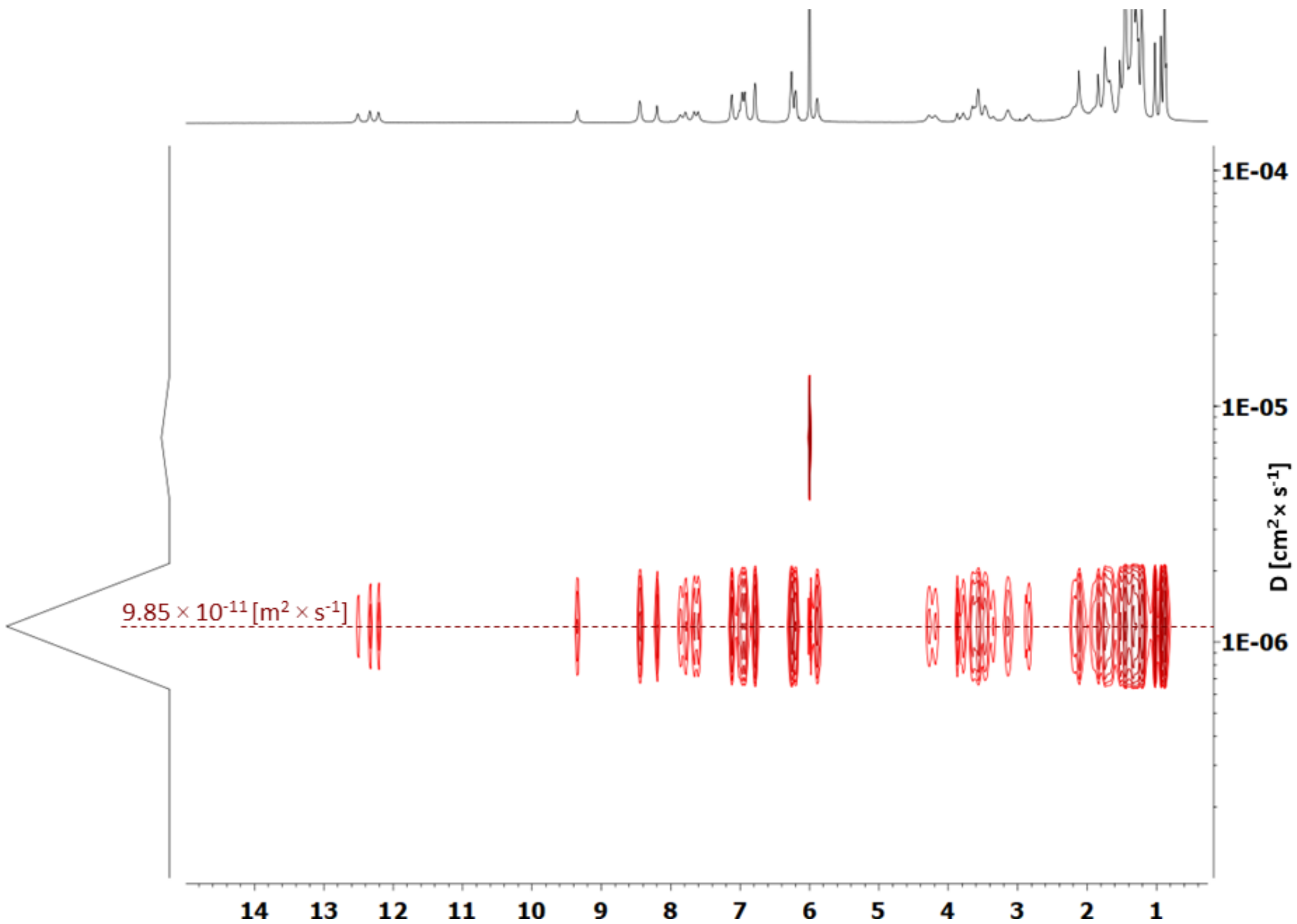

Figure S70. *DOSY NMR (600 MHz TCE-d$_2$) spectrum of **11**.*

Compound	TCE-*d*$_2$ *D* [m^2/s]	TCE-*d*$_2$ d_{sol} [Å]
7	1.34×10^{-10}	22.32
8	1.13×10^{-10}	26.47
9	1.12×10^{-10}	26.71
11	9.85×10^{-11}	30.37

Figure S71. *Table with solvodynamic diameter of assemblies of **7**, **8**, **9** and **11** in deuterated 1,1,2,2-tetrachloroethane (TCE-d$_2$), as calculated using the Stokes–Einstein equation for the DOSY NMR data recorded at 298 K, and $C = 1.0 \times 10^{-2}$ M.*

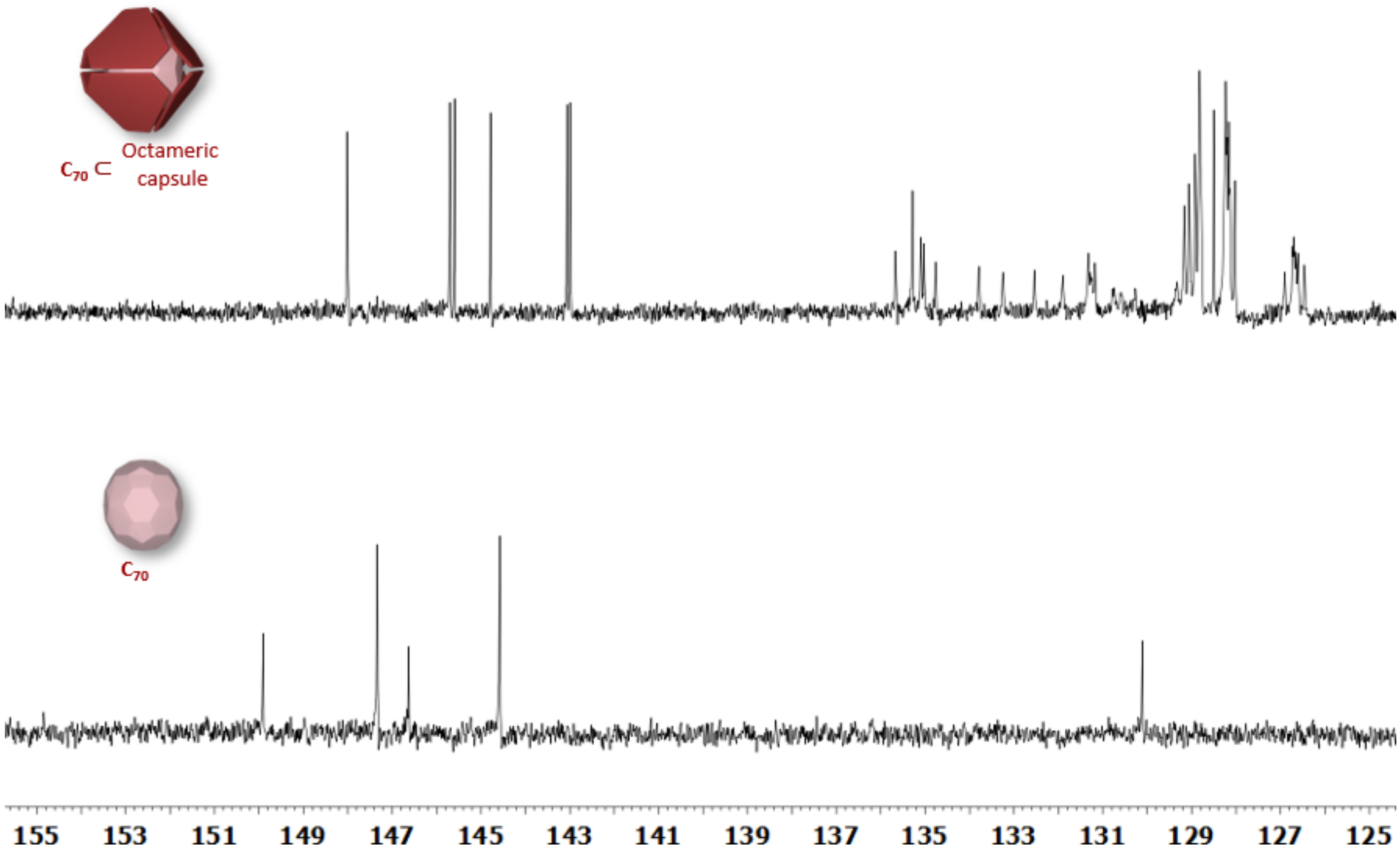

Figure S72. *Stacked* [13]*C NMR spectra (150 MHz, TCE-d₂) of mixture of compound **9** with C_{70} fullerene, and free C_{60} fullerene.*

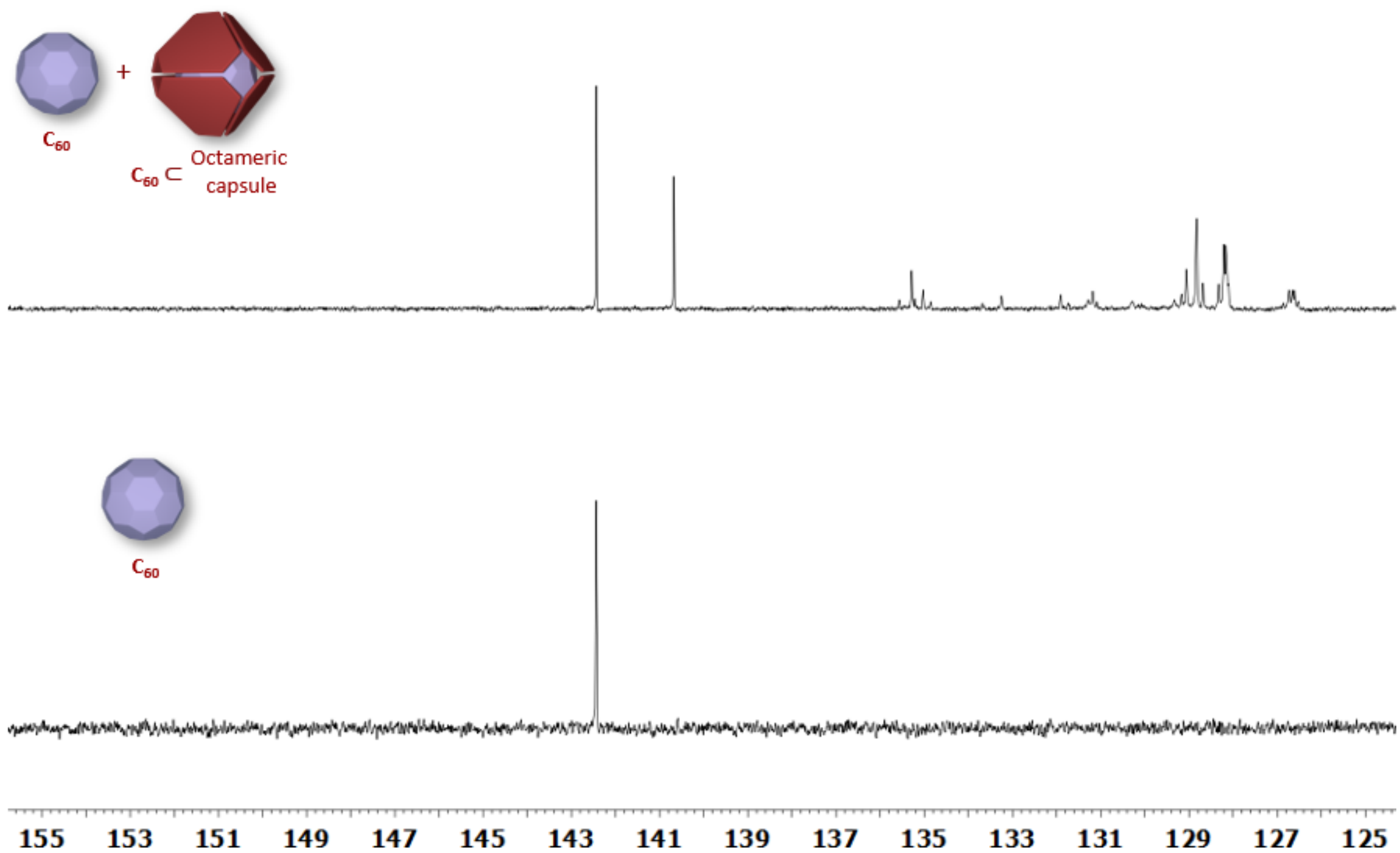

Figure S73. *Stacked* [13]*C NMR spectra (150 MHz, TCE-d₂) of mixture of compound **9** with C_{60} fullerene, and free C_{60} fullerene.*

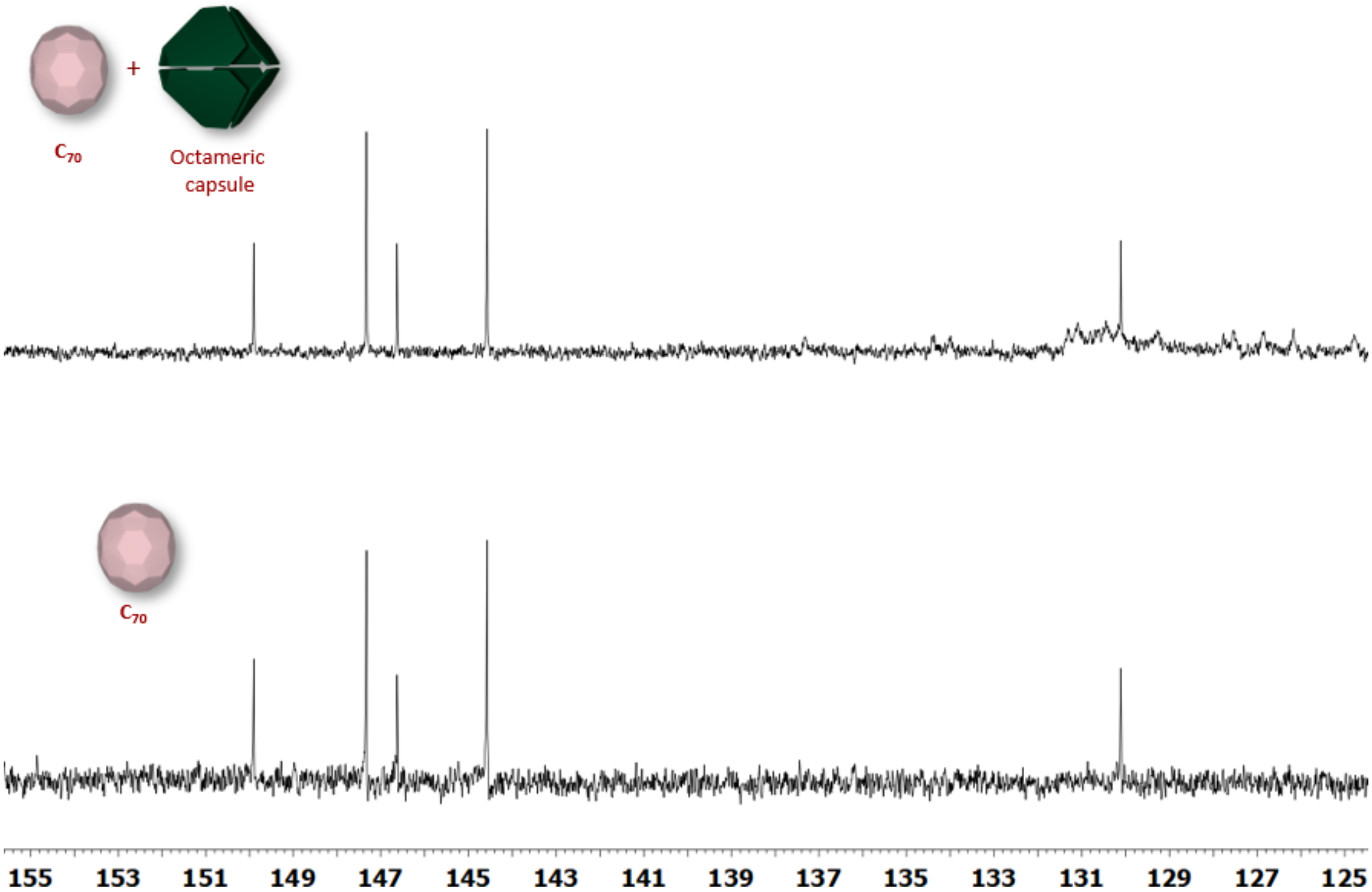

Figure S74. *Stacked* *^{13}C NMR spectra (150 MHz, TCE-d₂) of mixture of compound* ***11*** *with* $\boldsymbol{C_{70}}$ *fullerene, and free* $\boldsymbol{C_{70}}$ *fullerene.*

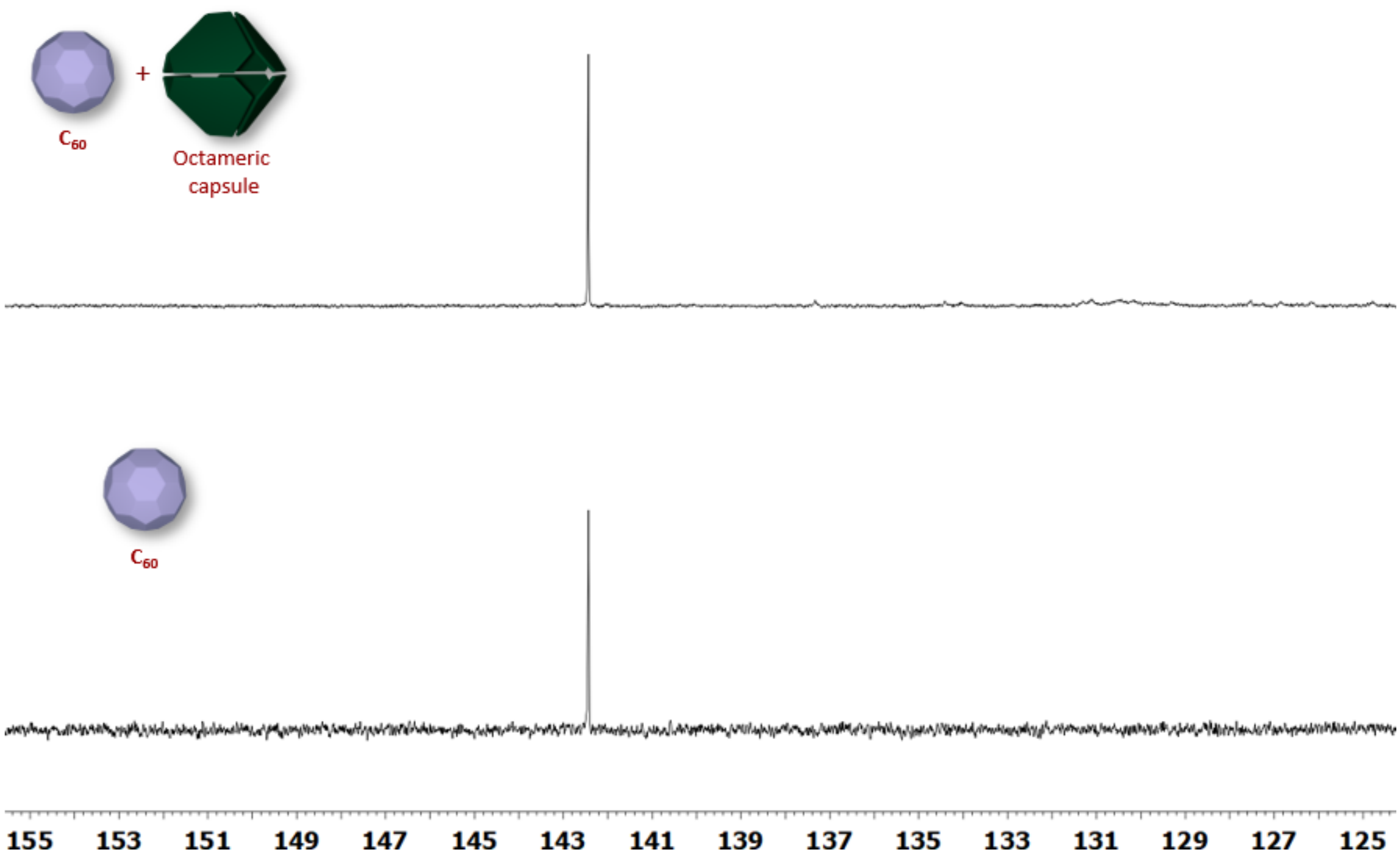

Figure S75. *Stacked* *^{13}C NMR spectra (150 MHz, TCE-d₂) of mixture of compound* ***11*** *with* $\boldsymbol{C_{60}}$ *fullerene, and free* $\boldsymbol{C_{60}}$ *fullerene.*

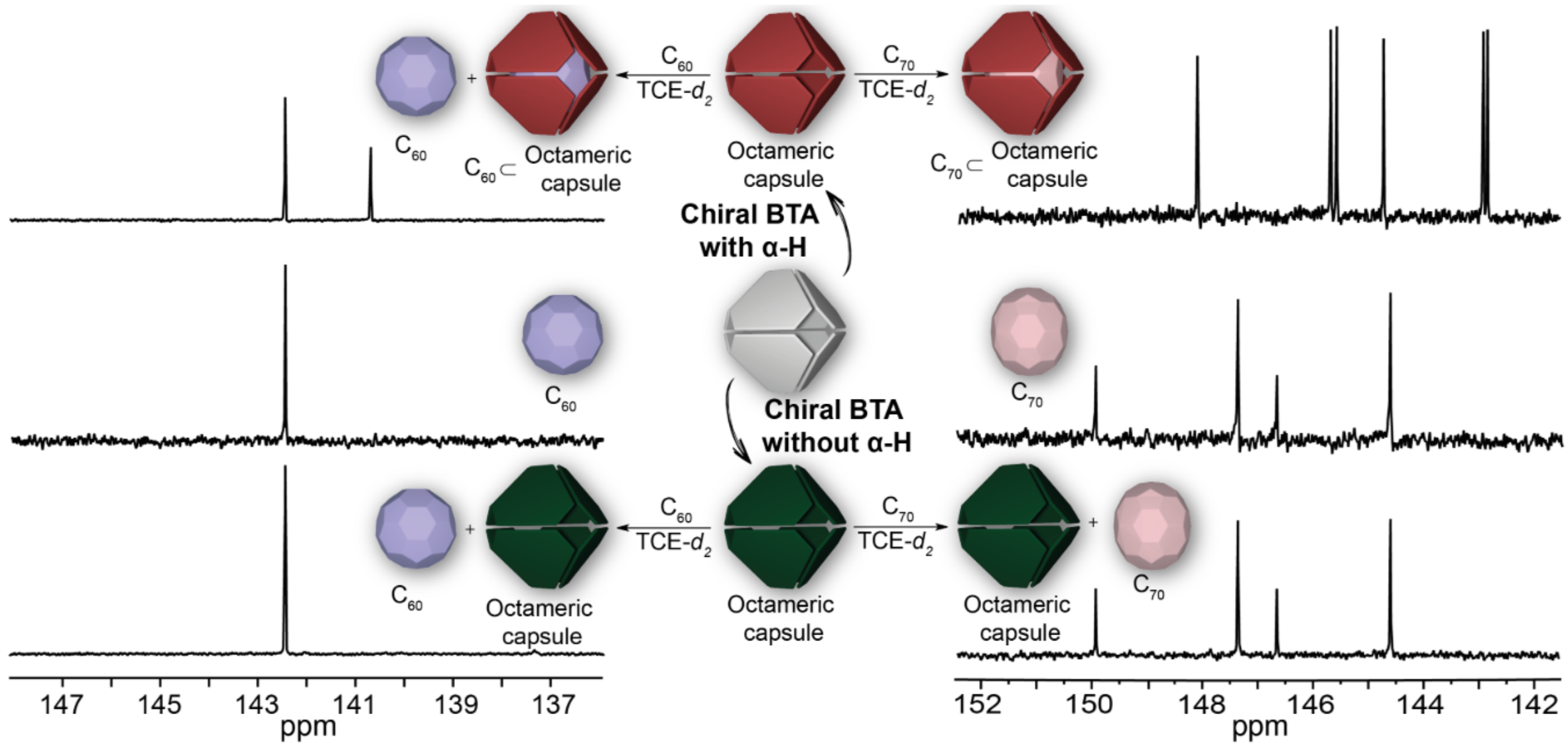

*Figure S76. Schematic illustration of the effect of the α-methyl group on fullerene encapsulation. Superimposed ^{13}C NMR spectra of a mixture of compound **9** with **C_{60}** fullerene (top left), **C_{60}** fullerene (middle left), and a mixture of compound **11** with **C_{60}** fullerene (bottom left), as well as a mixture of compound **9** with **C_{70}** fullerene (top right), **C_{70}** fullerene (middle right), and a mixture of compound **11** with **C_{70}** fullerene (bottom right) in TCE-d₂ (expansion of the fullerene signal region).*

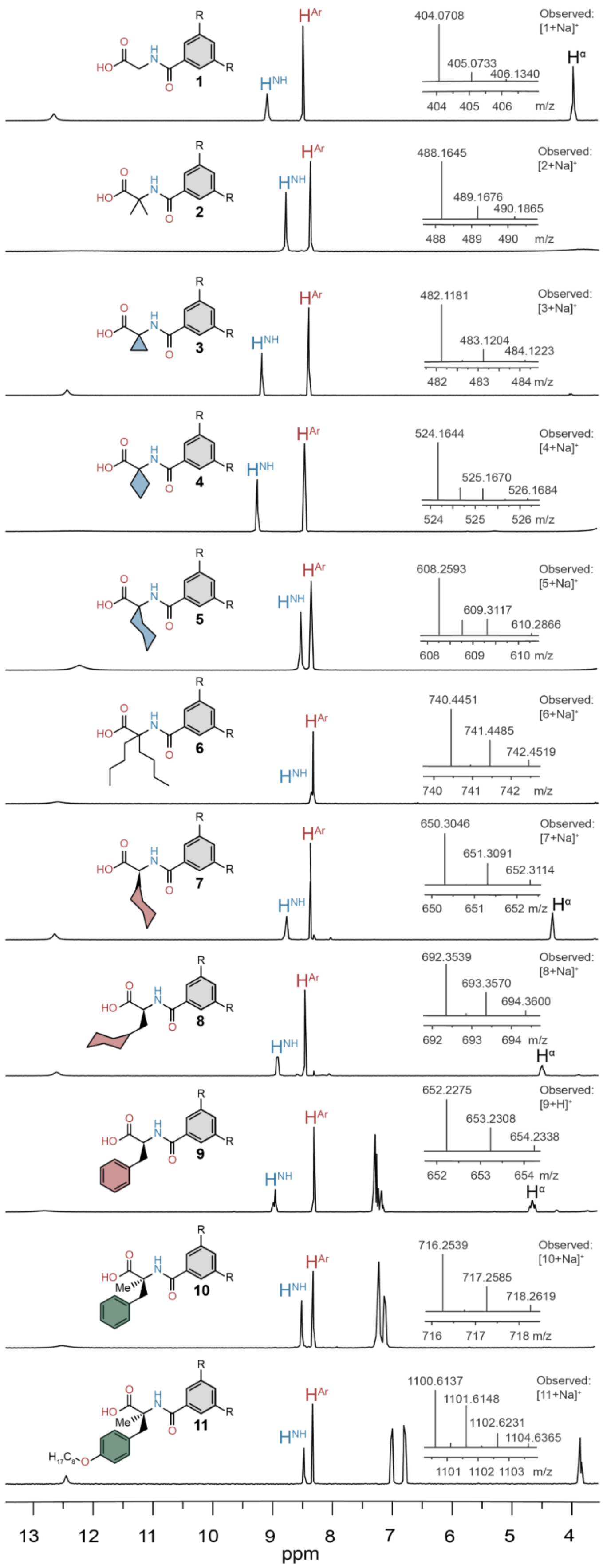

Figure S77. ^{1}H NMR (600 MHz, DMSO-d_6) spectra of **1**-**11**. Insets: HR-ESI-MS+ spectra of **1**-**11** as obtained from MeOH solutions.

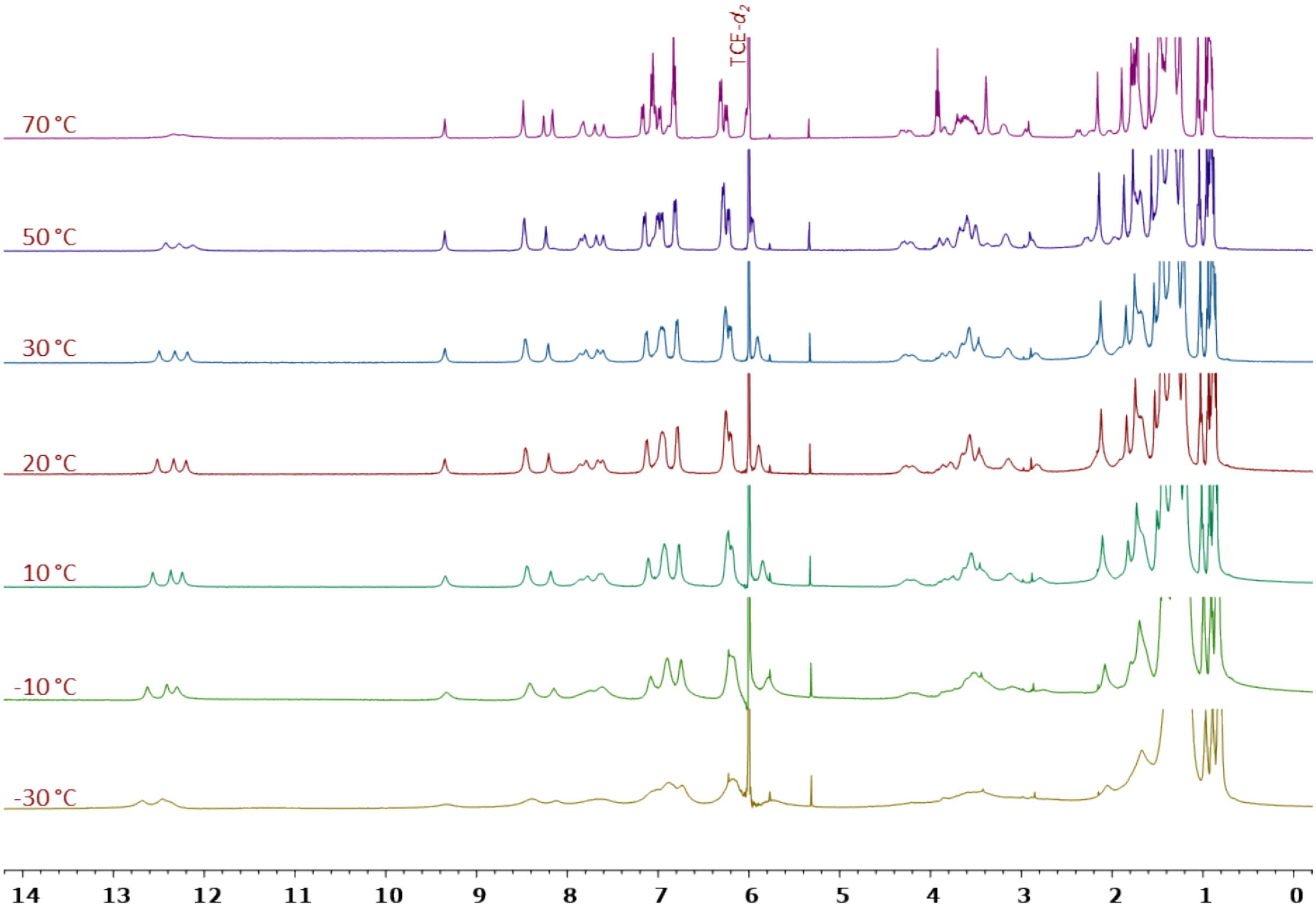

Figure S78. *Variable temperature NMR spectra (400 MHz TCE-d_2) of **11** in temperature range -30 °C - +70 °C.*

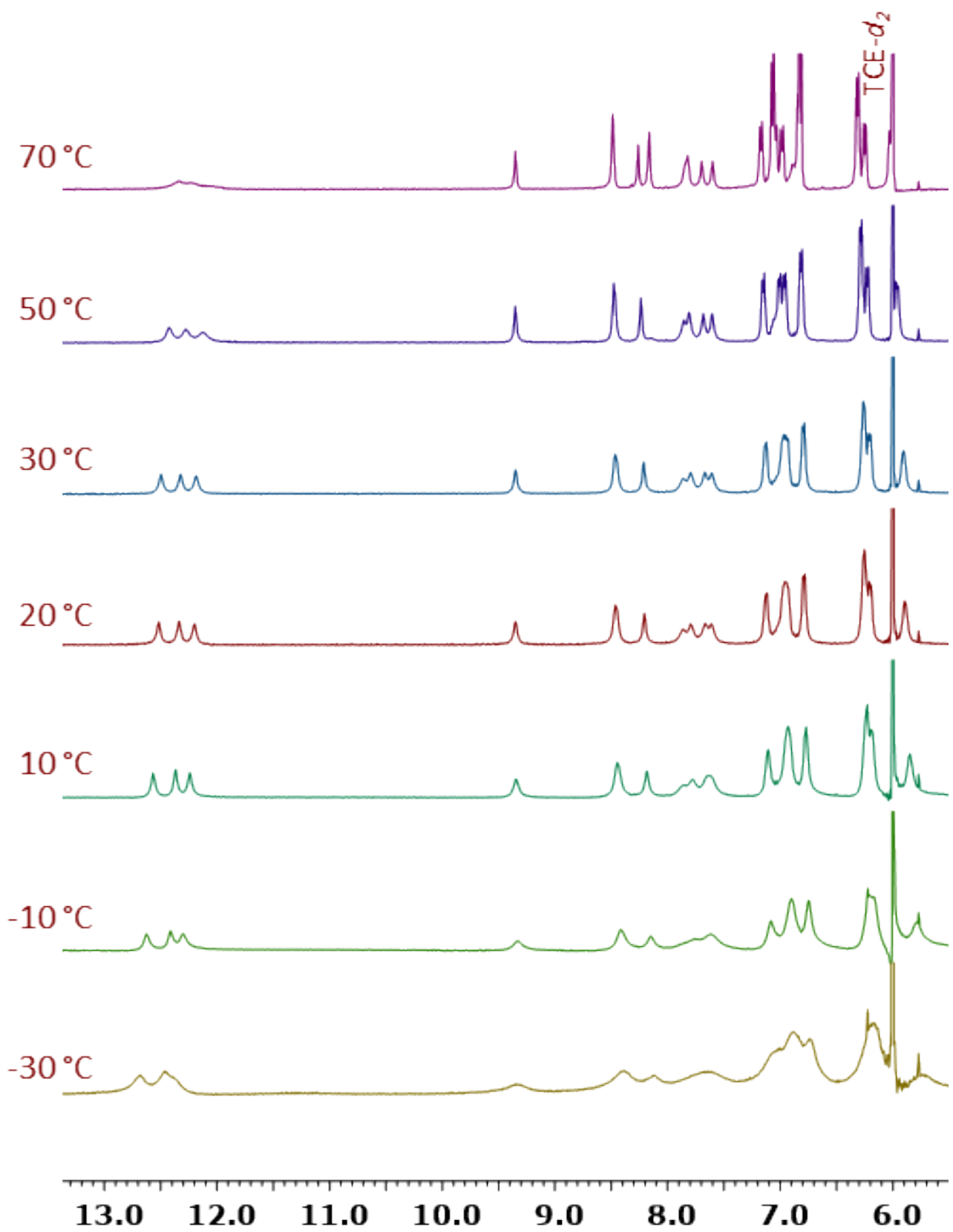

Figure S79. *Variable temperature NMR spectra (400 MHz TCE-d_2) of **11** (6-13 ppm range) in temperature range -30 °C - +70 °C.*

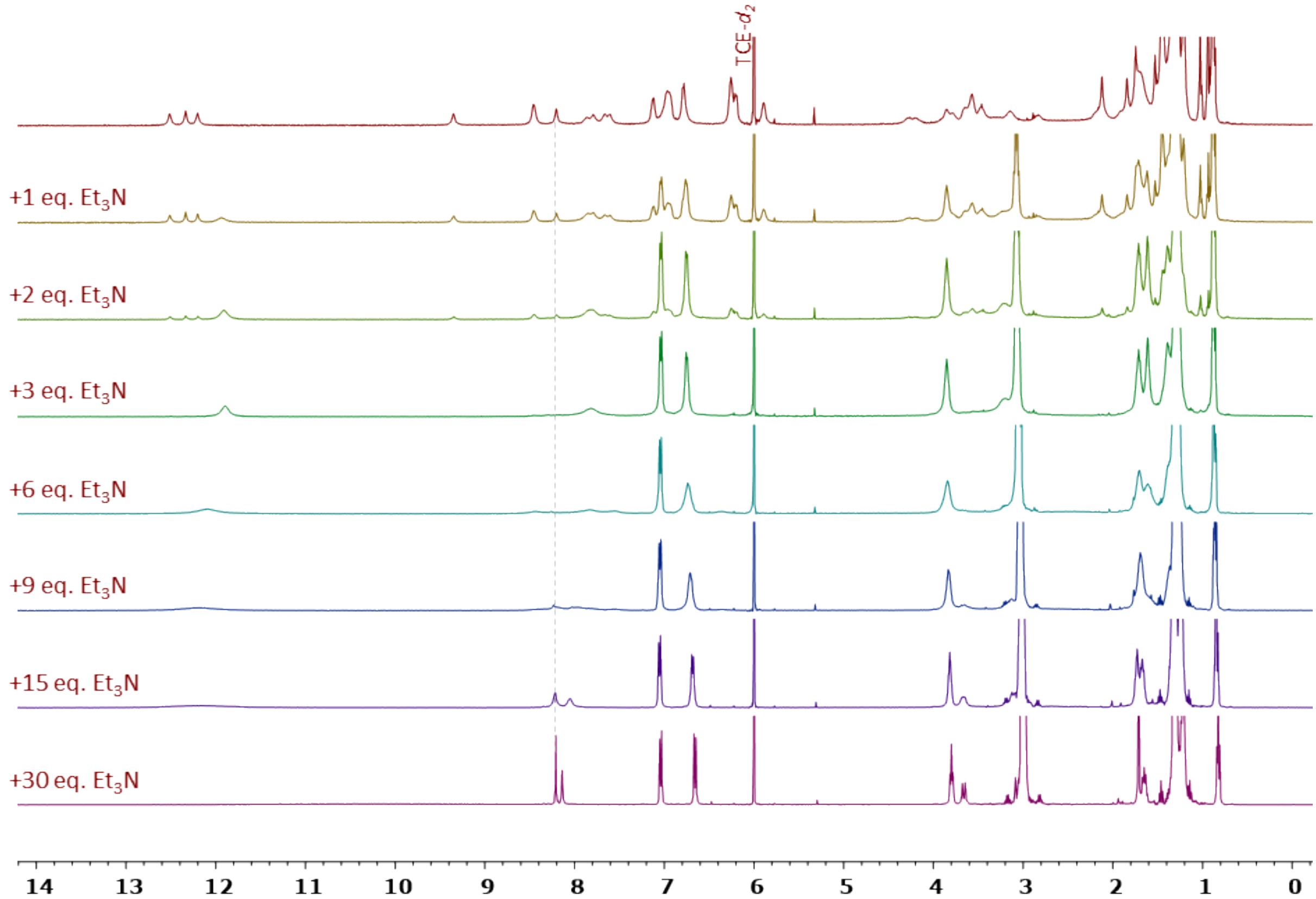

Figure S80. *Base titration spectra (400 MHz TCE-d_2) of **11** recorded at $C = 1.0 \times 10^{-3}$M. 1, 2, 3, 6, 9, 15, 30 equivalents per BTA molecule.*

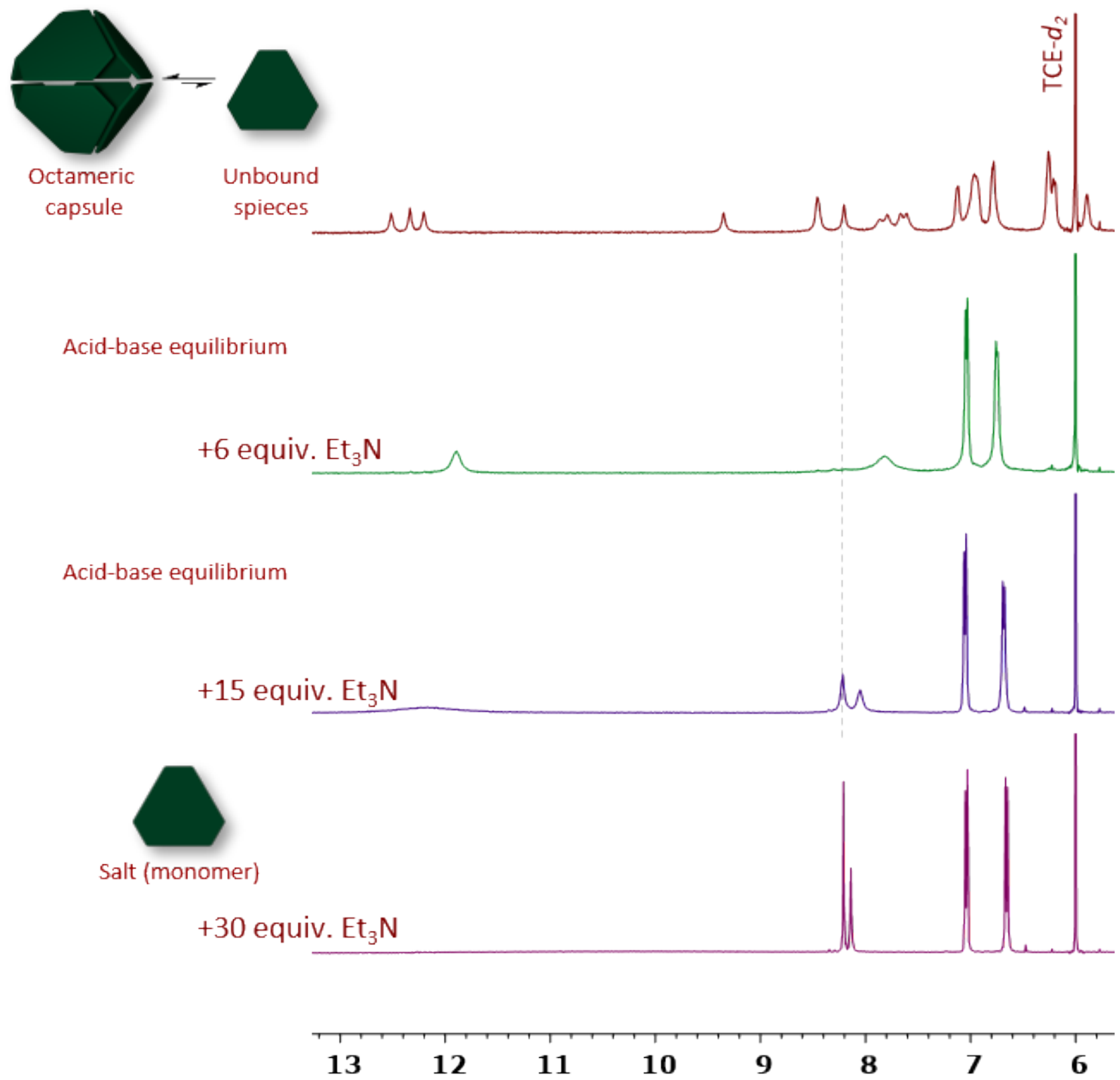

Figure S81. *Base titration spectra (400 MHz TCE-d_2) of* ***11*** *(6-13 ppm range) recorded at $C = 1.0 \times 10^{-3}$ M. 6, 15, 30 equivalents per BTA molecule.*

4 MS data

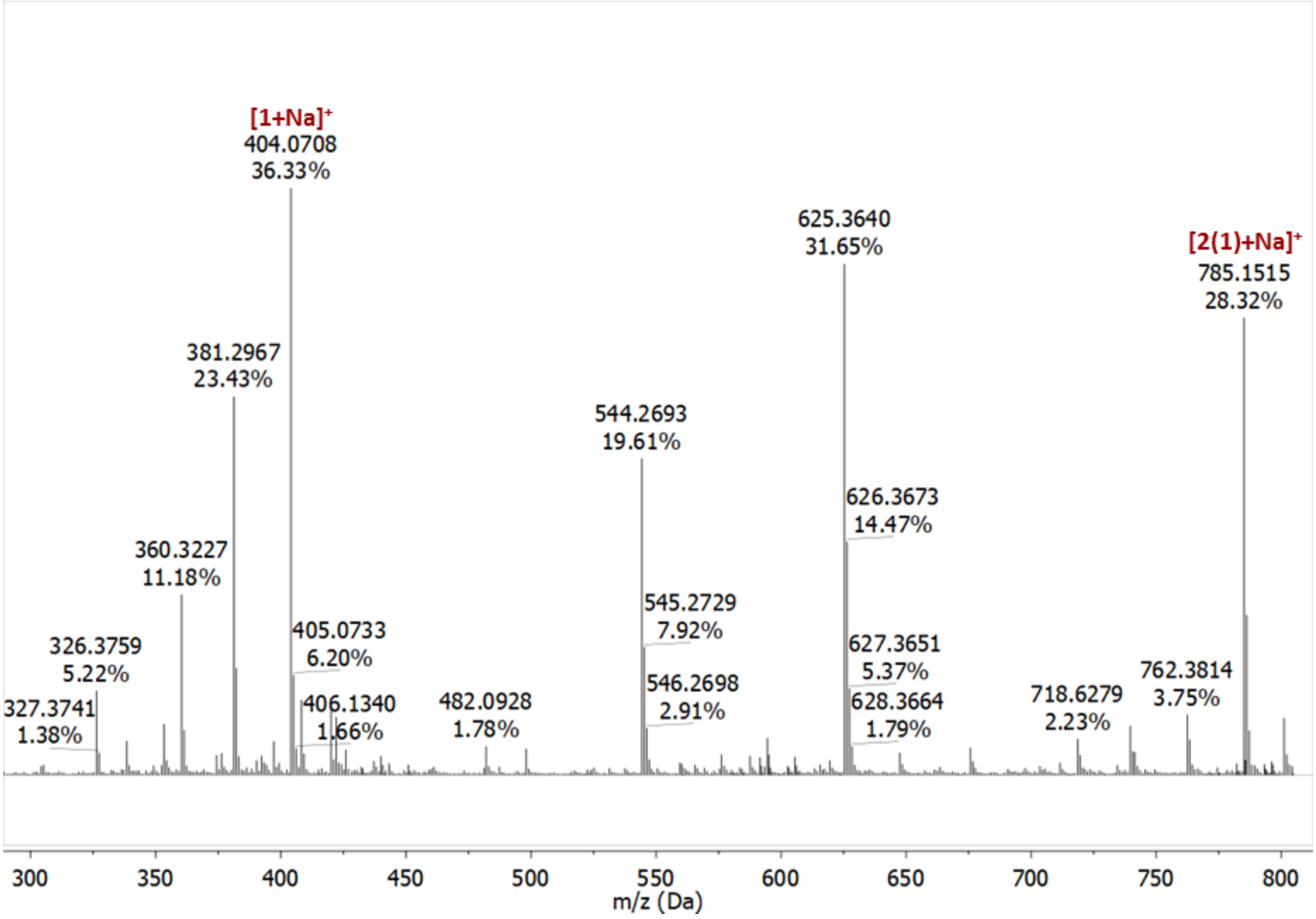

Figure S82. *ESI-MS spectrum of 1.*

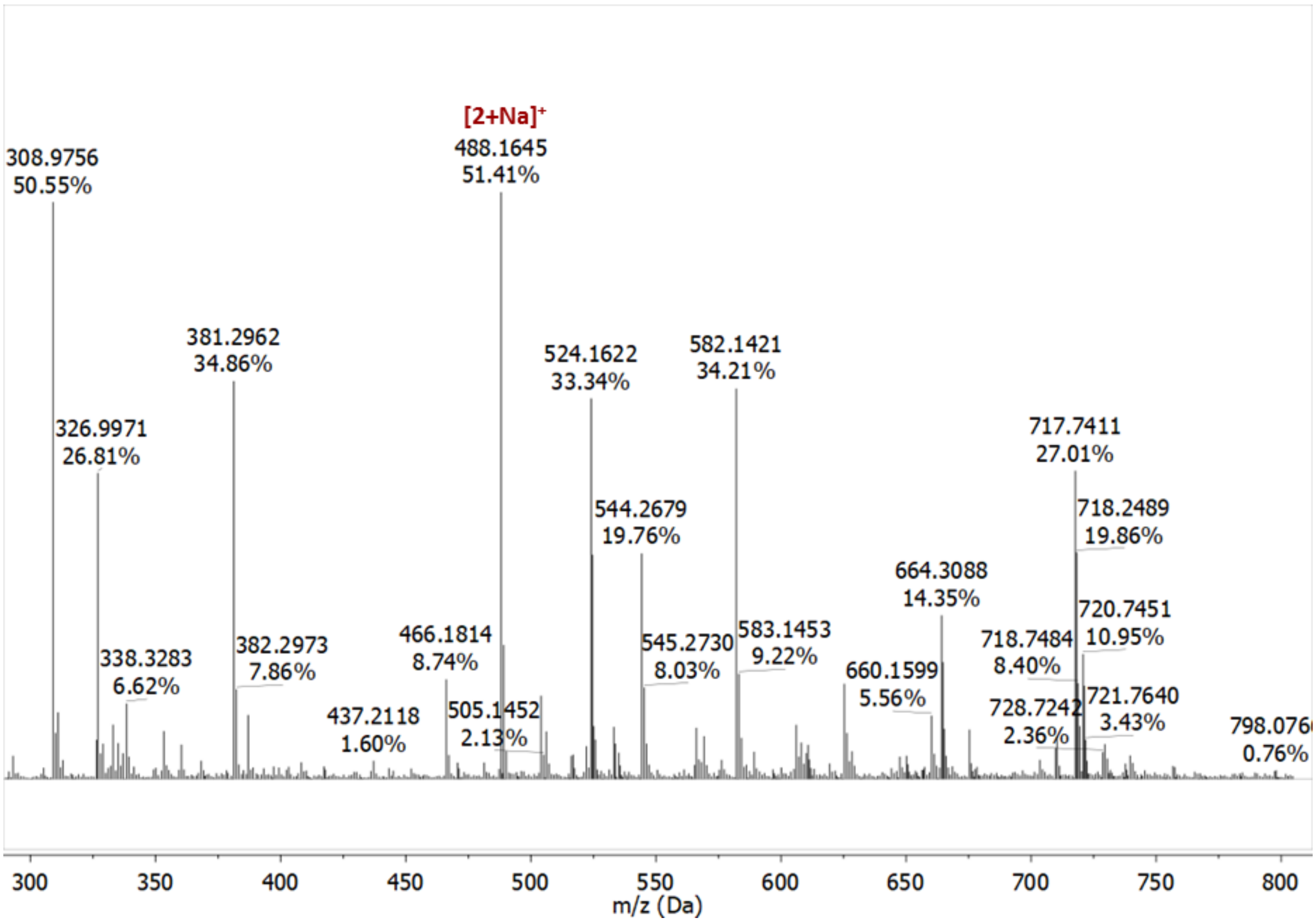

Figure S83. ESI-MS spectrum of 2.

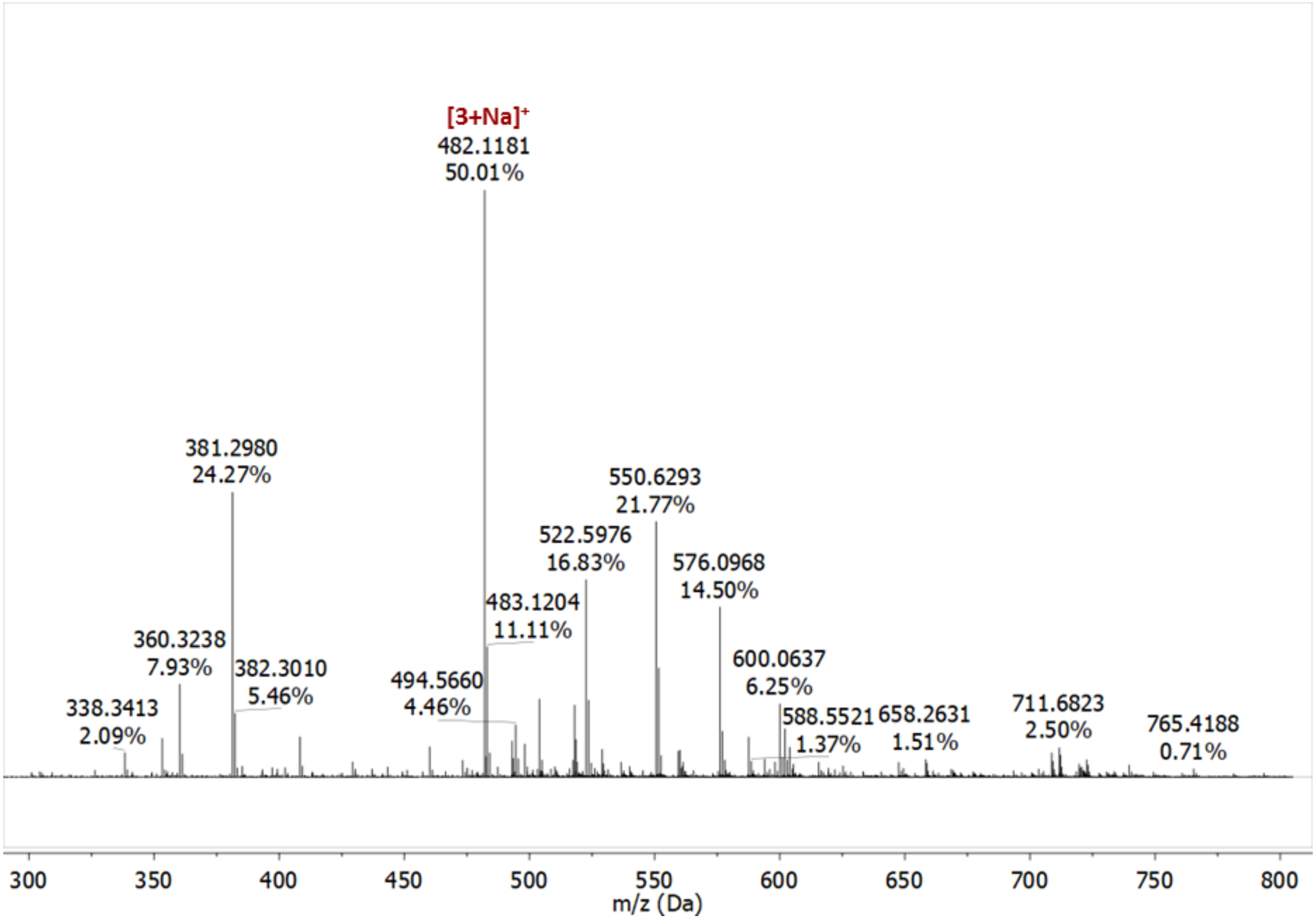

Figure S84. ESI-MS spectrum of 3.

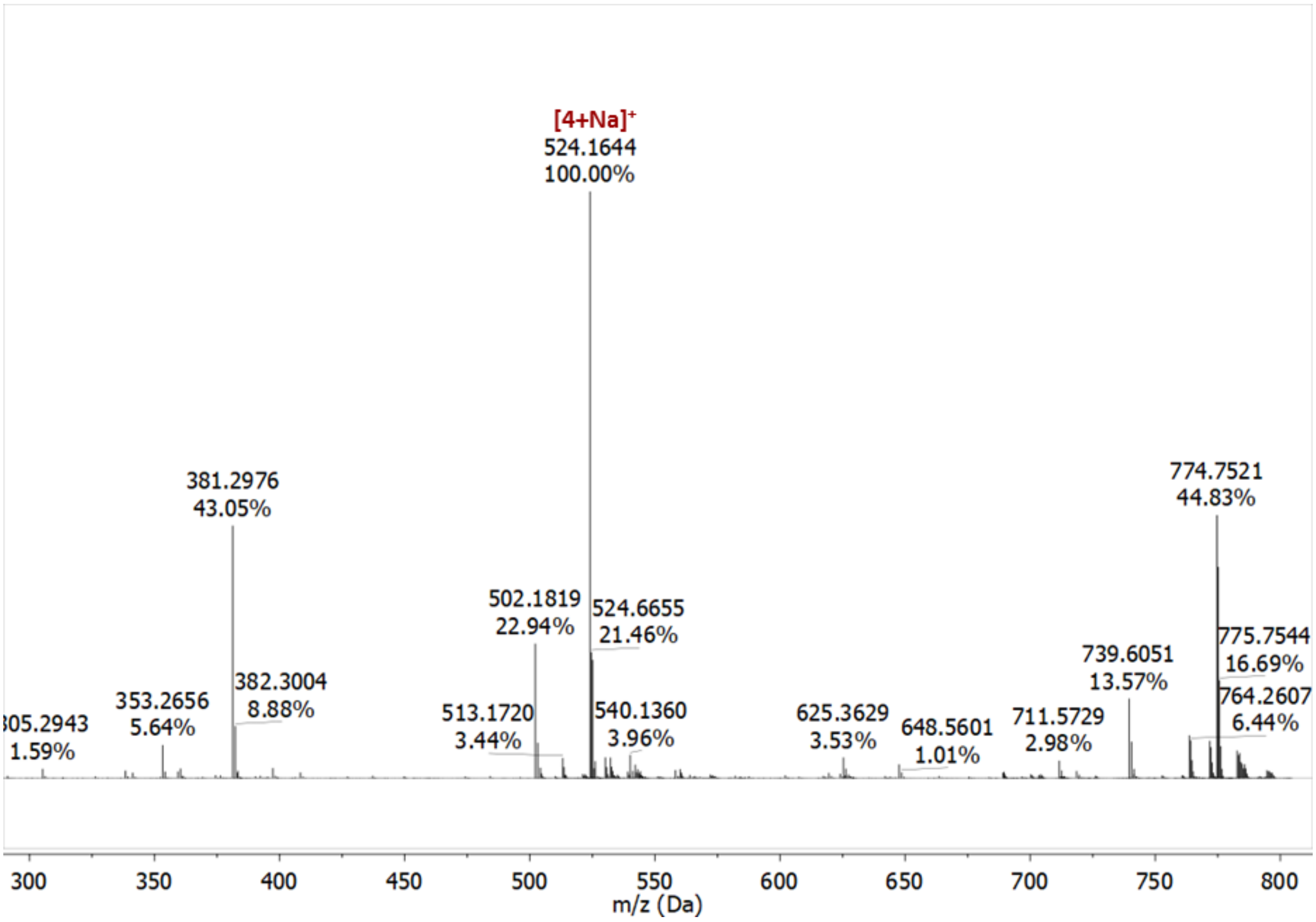

Figure S85. ESI-MS spectrum of 4.

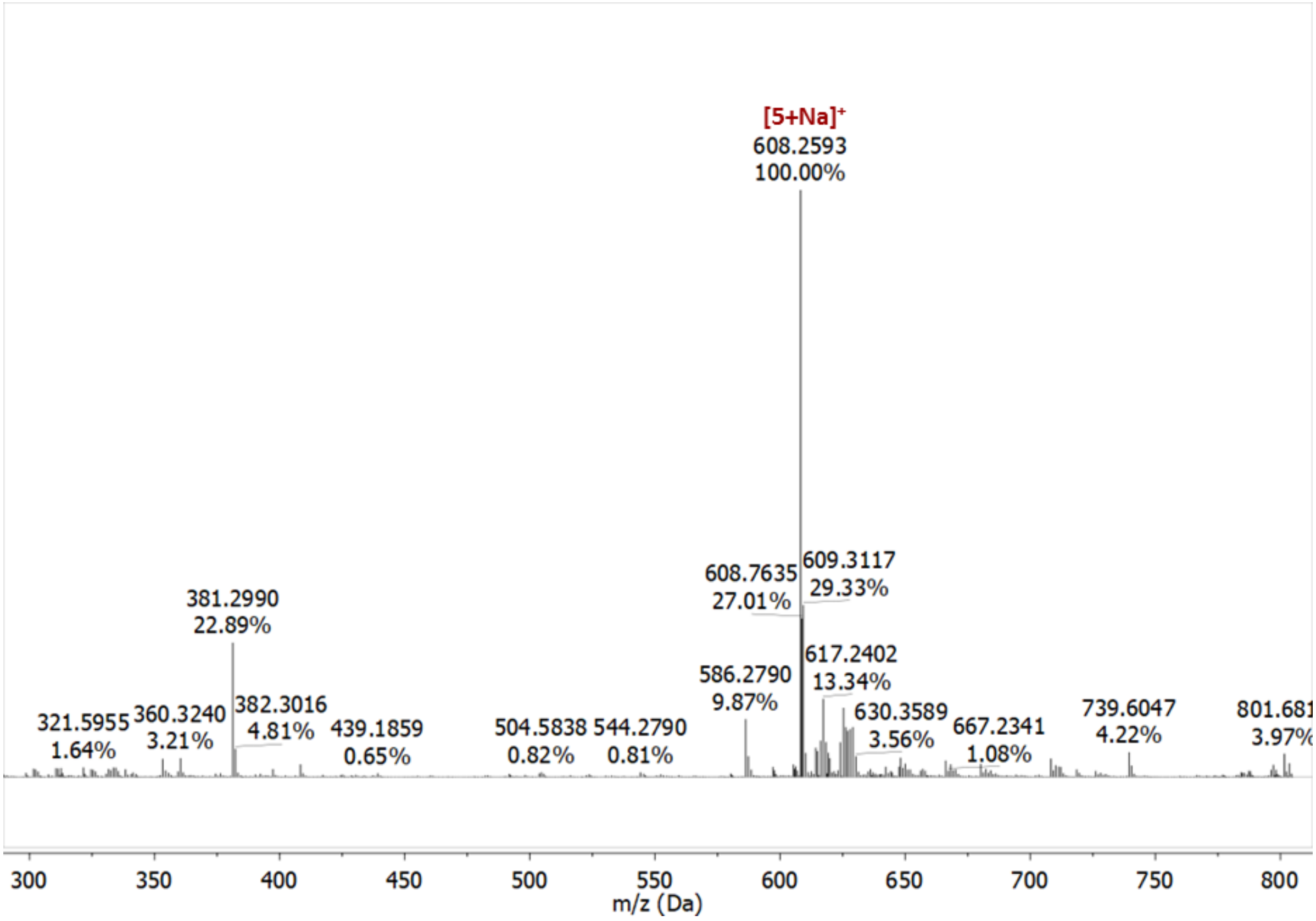

Figure S86. ESI-MS spectrum of 5.

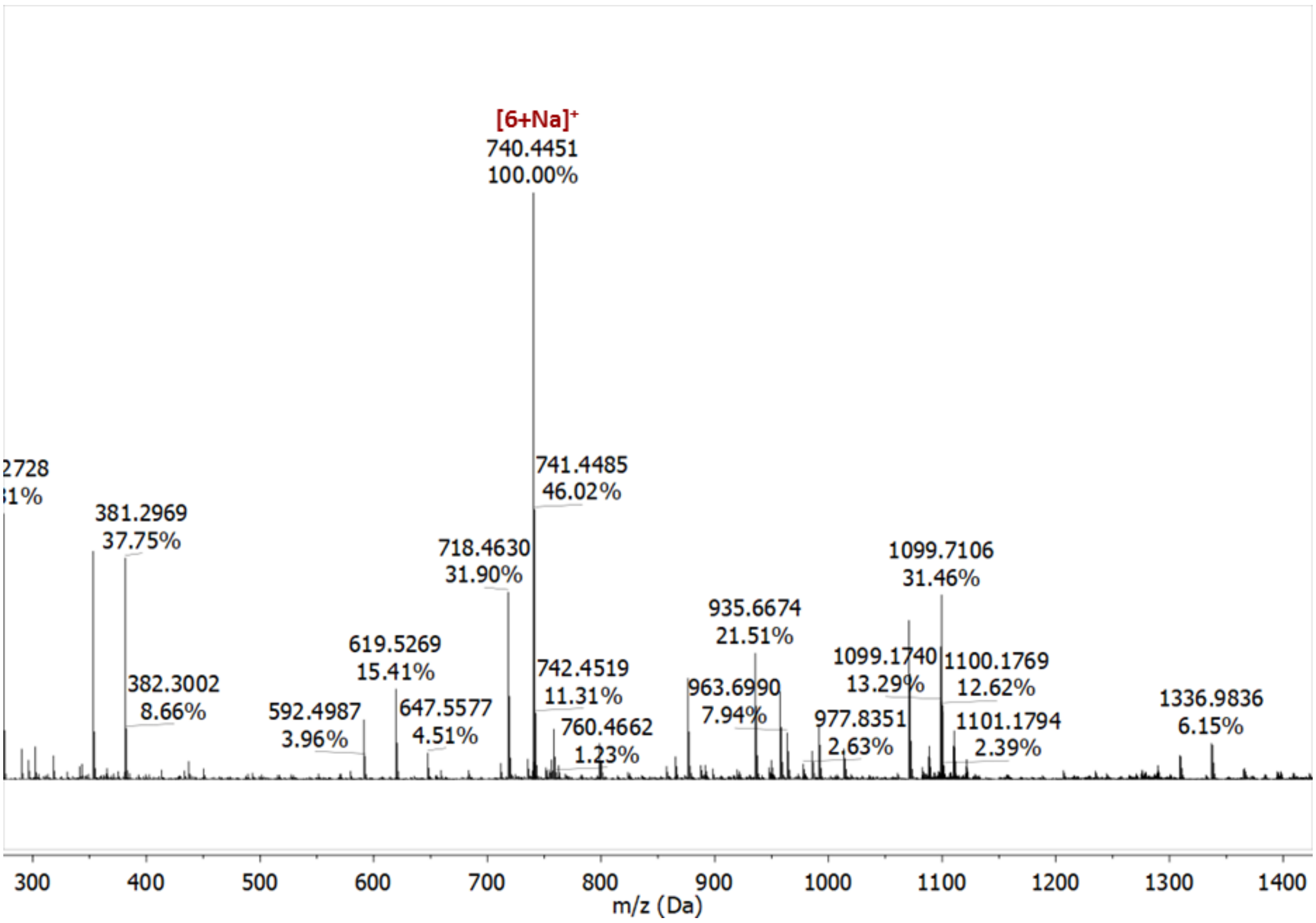

Figure S87. ESI-MS spectrum of 6.

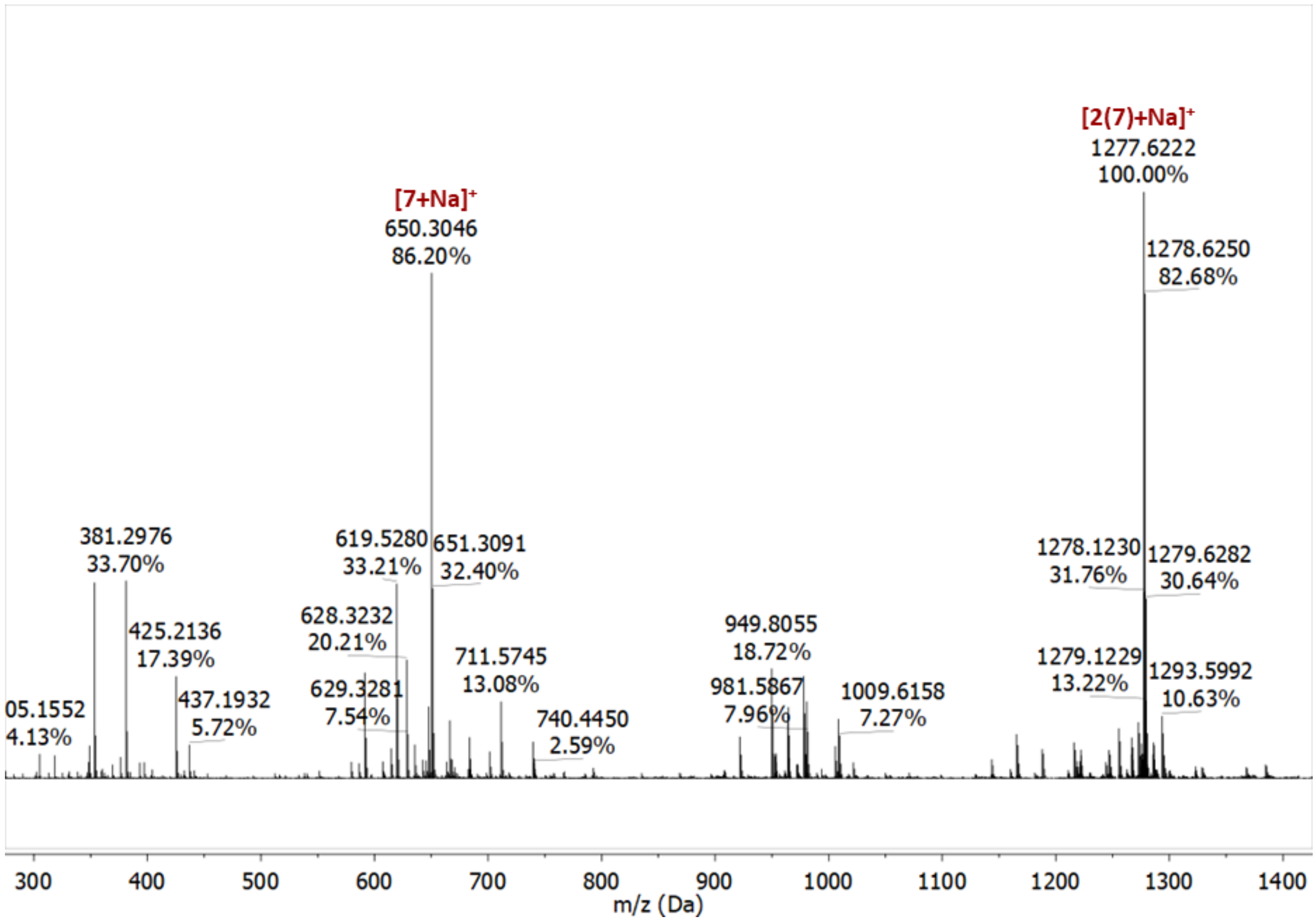

Figure S88. ESI-MS spectrum of 7.

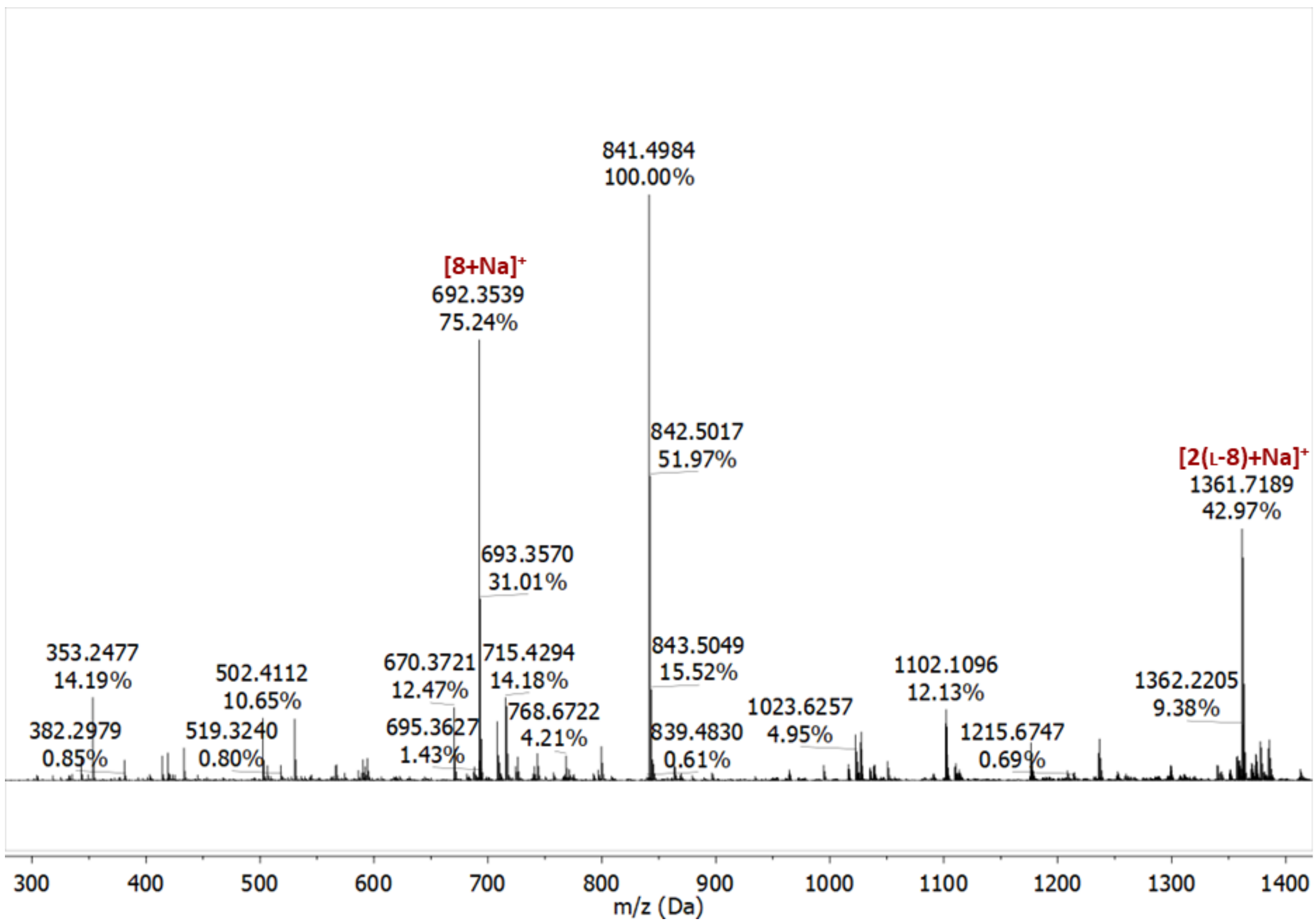

Figure S89. ESI-MS spectrum of 8.

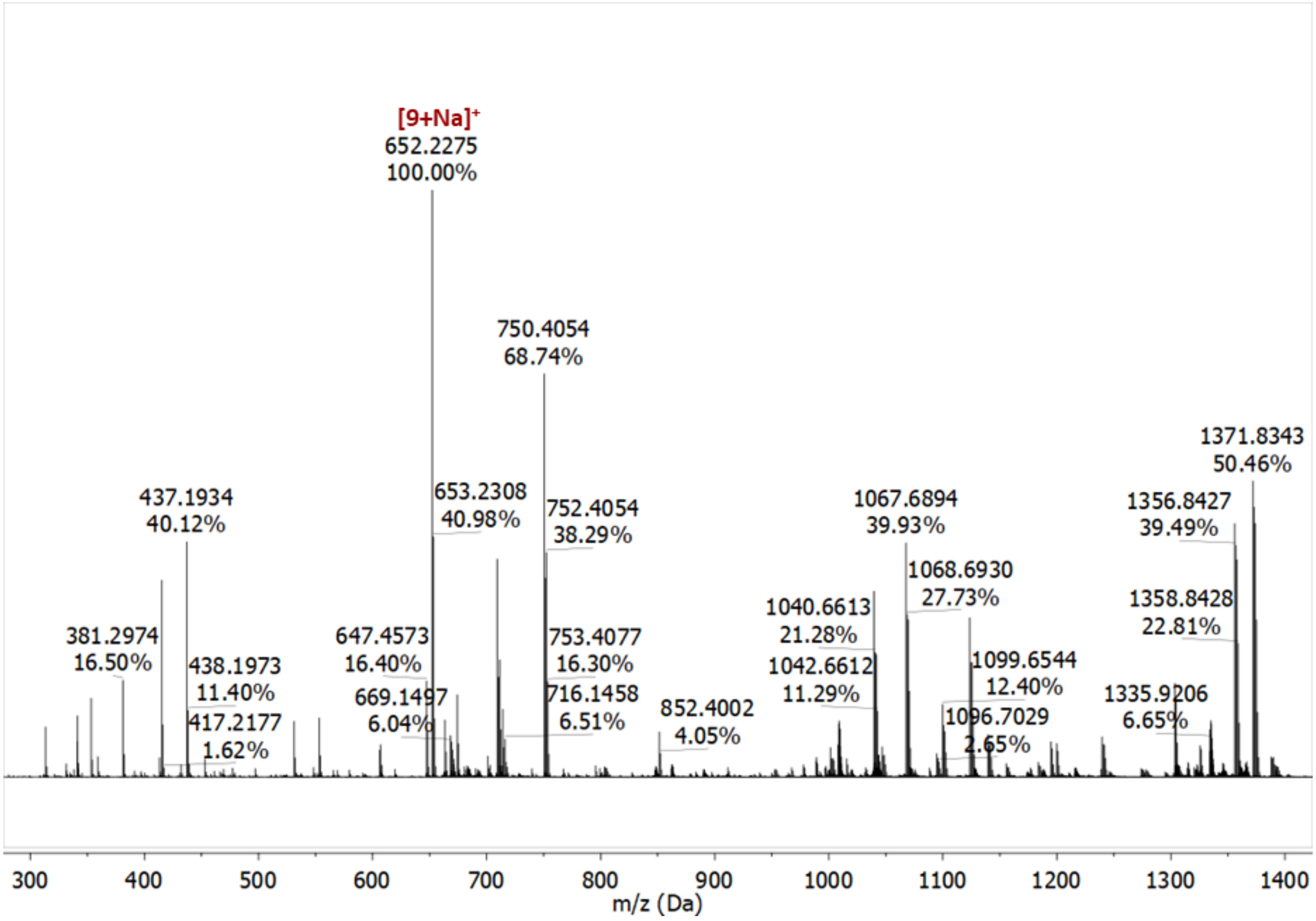

Figure S90. ESI-MS spectrum of 9.

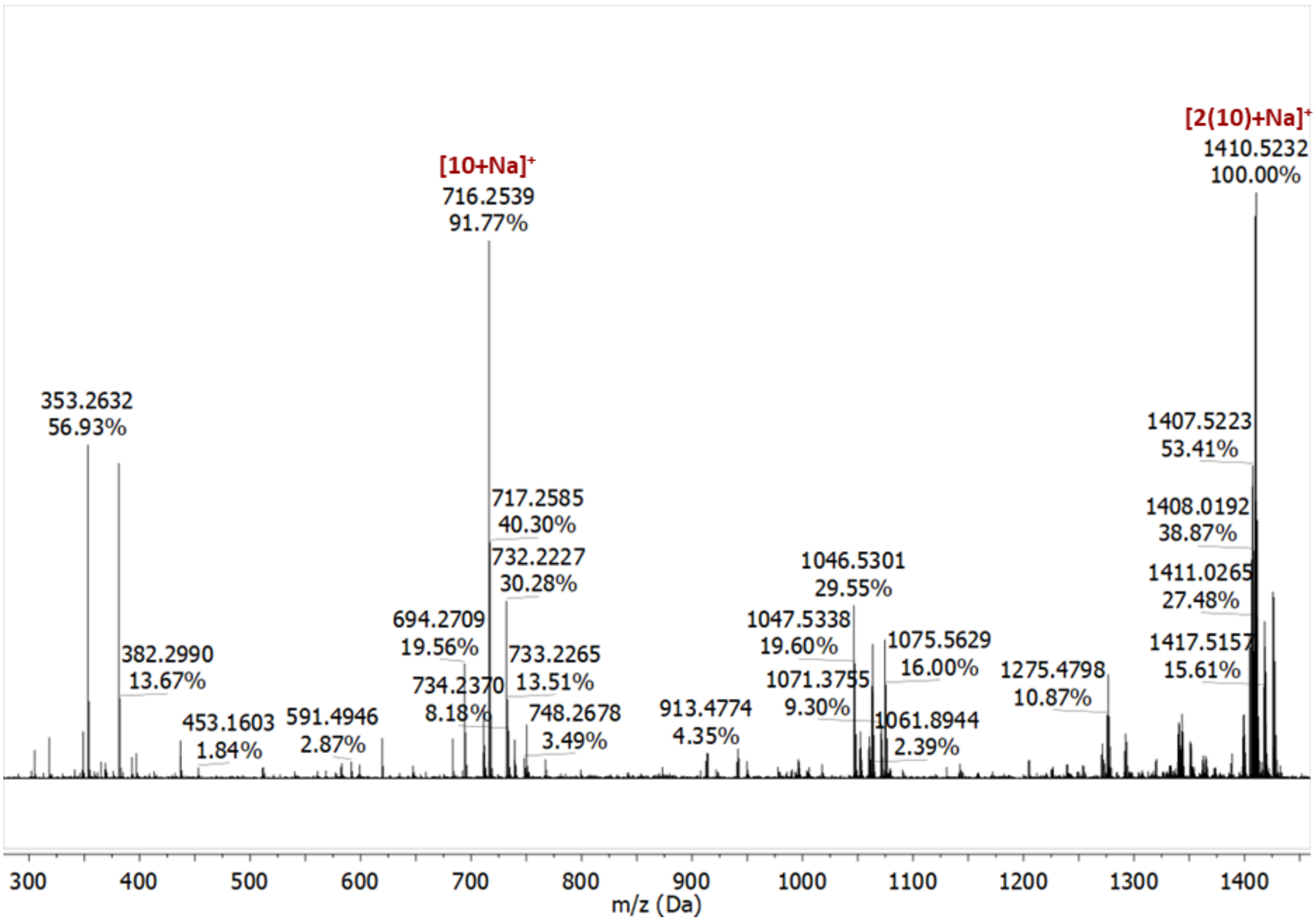

Figure S91. ESI-MS spectrum of 10.

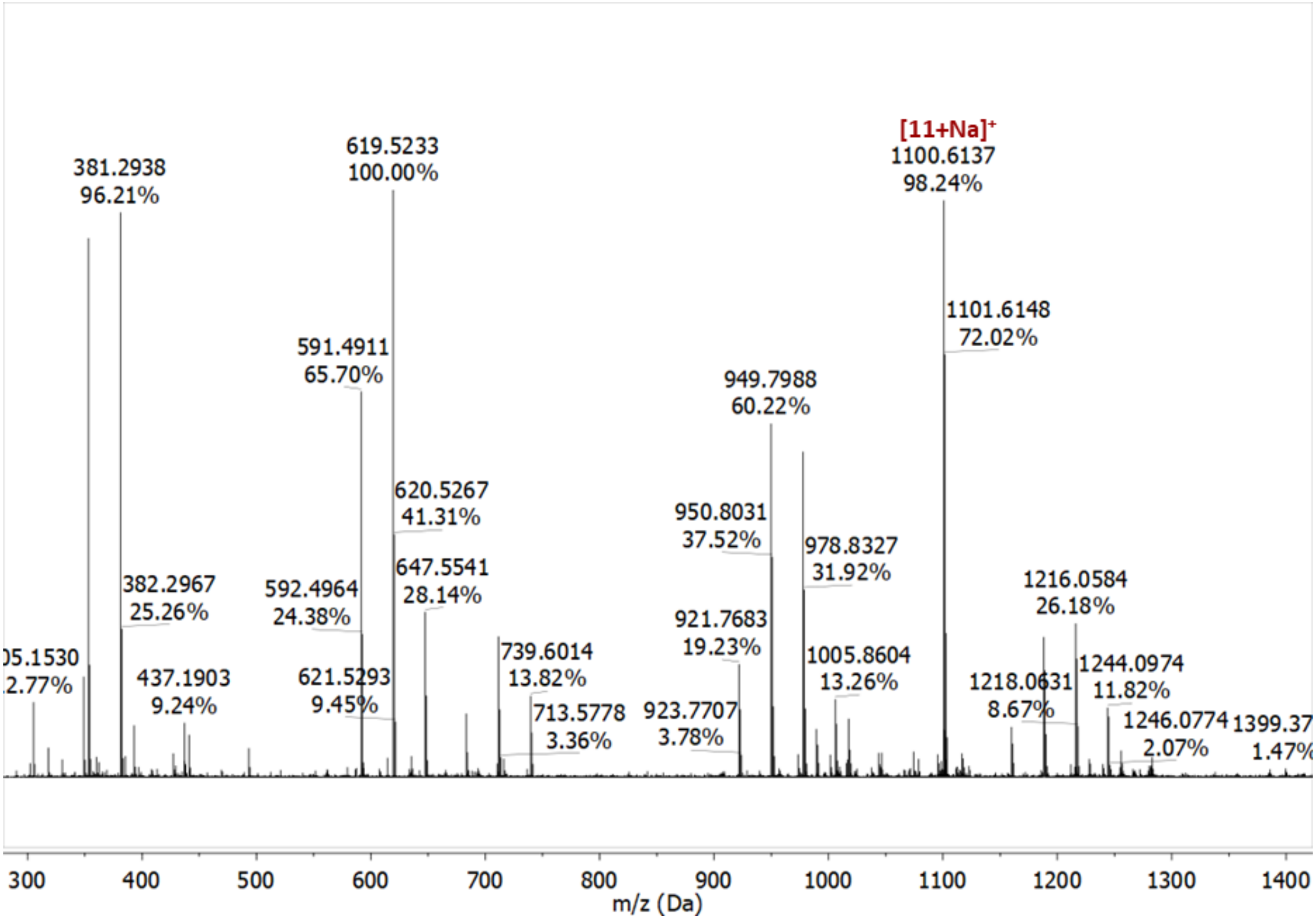

Figure S92. ESI-MS spectrum of 11.

5 FT-IR data

5.1 FT-IR analysis in solid state

The components **1**-**11** were also characterized in the solid state using FT-IR spectroscopy. The isolated solid products were finely ground with KBr and analysed as pressed pellets (Figure S107). The most diagnostically relevant absorption bands correspond to carbonyl stretching vibrations-namely, *ν(C=O)*^{acid} and *ν(C=O)*^{amide}, which are directly involved in intermolecular hydrogen bonding. For achiral synthons (**1**-**6**), characteristic absorption bands were observed in the ranges of 1720-1710 cm^{-1} and 1660-1640 cm^{-1} for the carboxylic and amide carbonyl groups, respectively. These features are consistent with participation in hydrogen bonding. However, the significant broadening and splitting of these bands suggest the formation of rather poorly organized aggregates in the solid state (Figure S93a-b).[7] In contrast, the chiral derivatives (**7**-**9**) displayed more well-defined absorption bands centered around 1725 and 1655 cm^{-1}, corresponding to hydrogen-bonded carboxylic and amide groups, respectively. Notably, these bands exhibit minimal splitting compared to their achiral counterparts, indicating higher order and directionality of the hydrogen bonding network (Figure S93c).[7] Comparable results were obtained for α-methylated derivatives (**10**, **11**), which exhibit bands at 1721 and 1655 cm^{-1} (Figure S93d). However, in these cases, the band shapes are less uniform, suggesting an increased degree of structural heterogeneity.

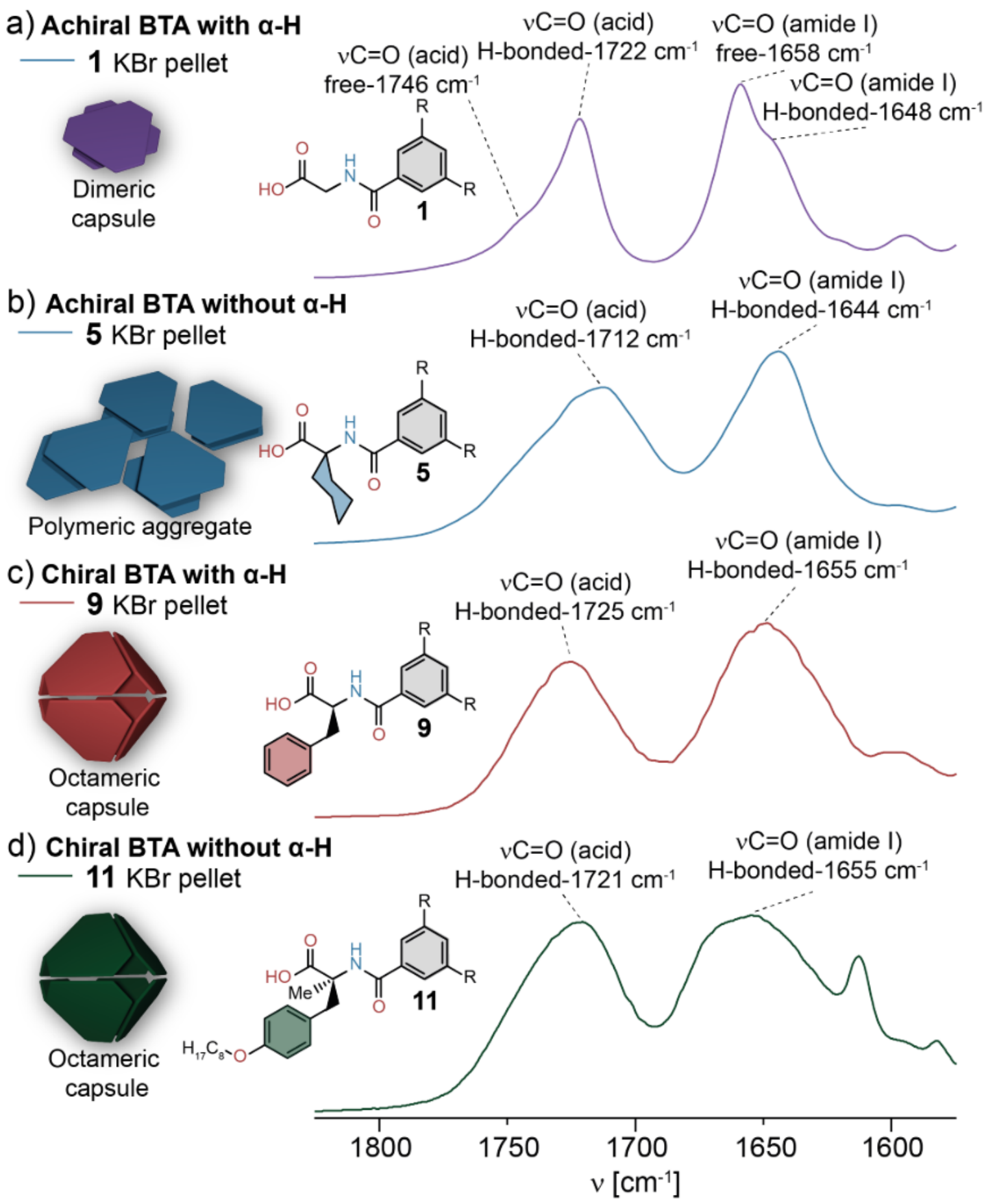

*Figure S93. Solid-state FT-IR spectra (KBr pellet) of selected compounds : a) **1**; b) **5**; c) **9**; and d) **11**. Characteristic absorption bands corresponding to hydrogen-bonded carboxylic and amide carbonyl groups are indicated. For spectra of all compounds see Figure S107.*

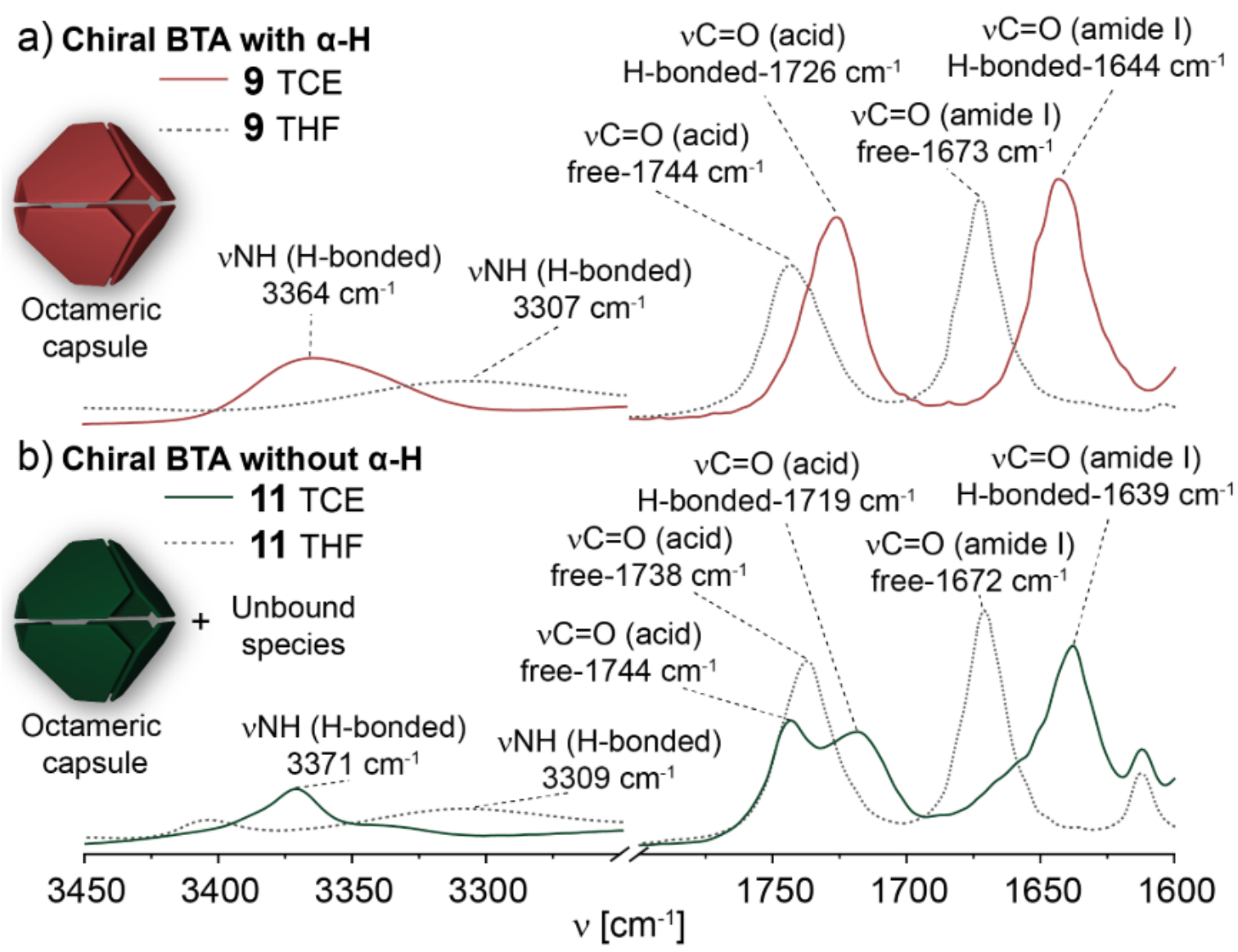

*Figure S94. a) Solution state FT-IR spectra (TCE and THF) of **9**. b) Solution state FT-IR spectra (TCE and THF) of **11**.*

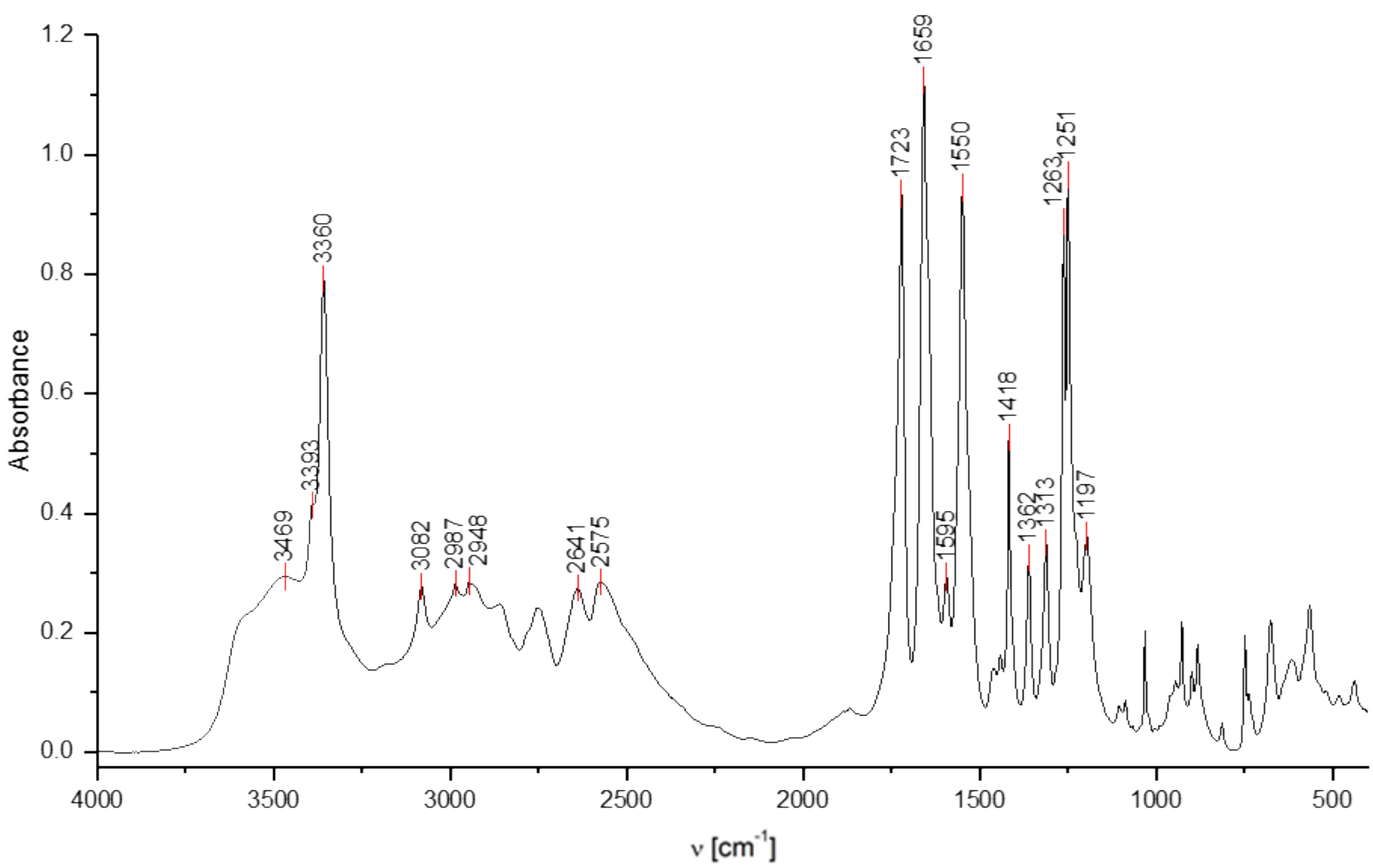

*Figure S95. FT-IR spectrum of **1** in KBr pellet recorded at 298 K.*

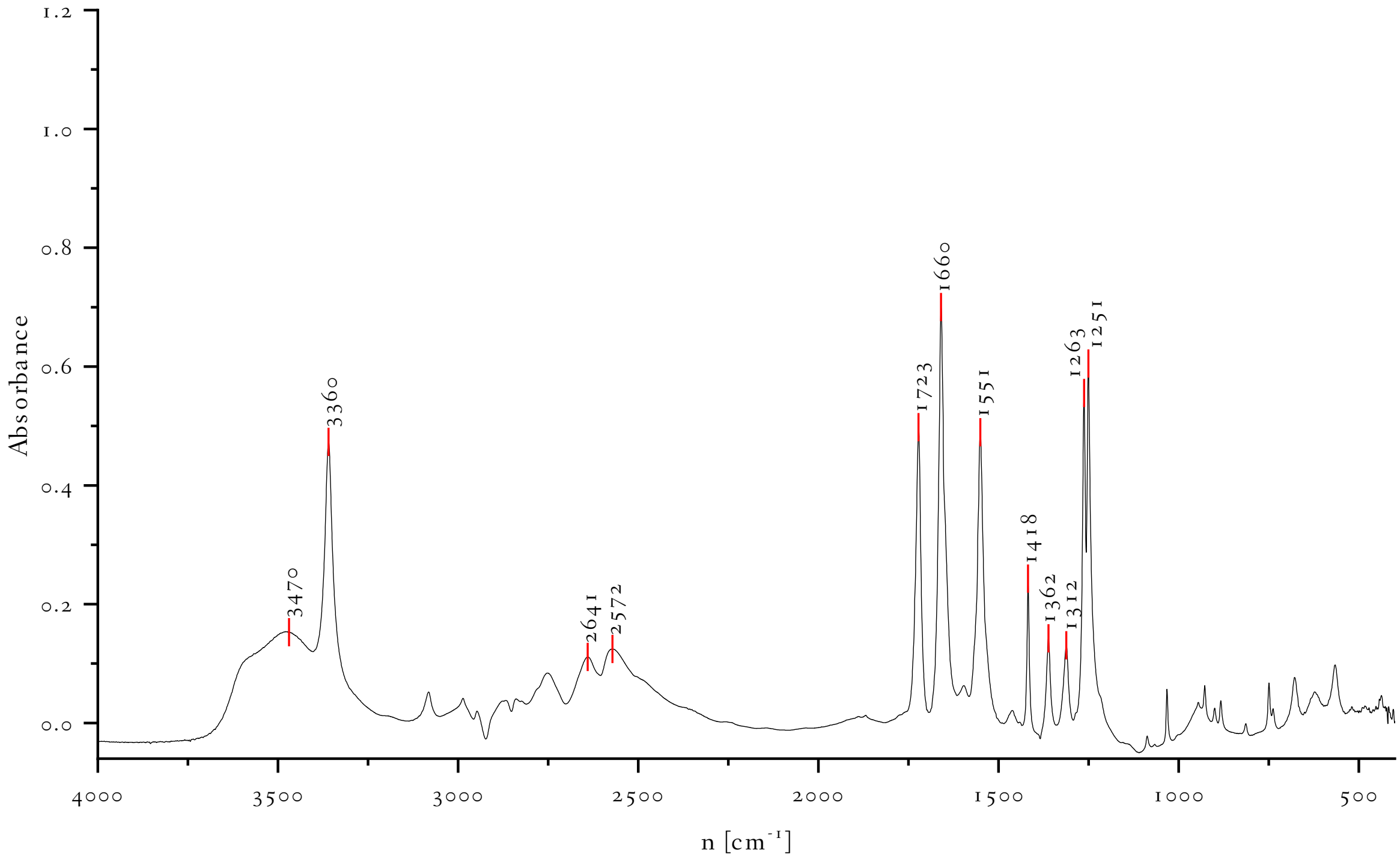

Figure S96. *FT-IR spectrum of **1·3H_2O** in KBr pellet recorded at 298 K.*

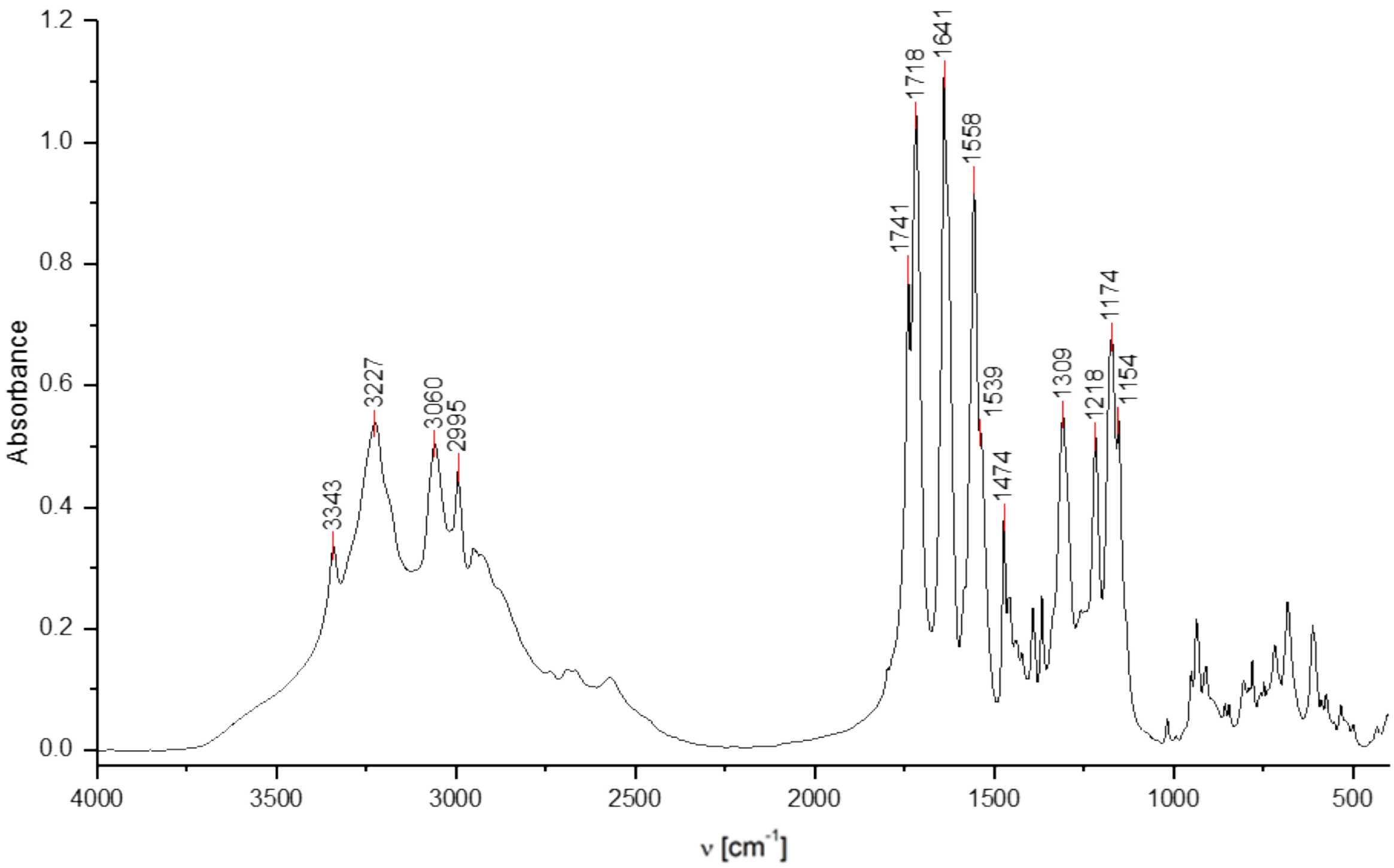

Figure S97. *FT-IR spectrum of **2** in KBr pellet recorded at 298 K.*

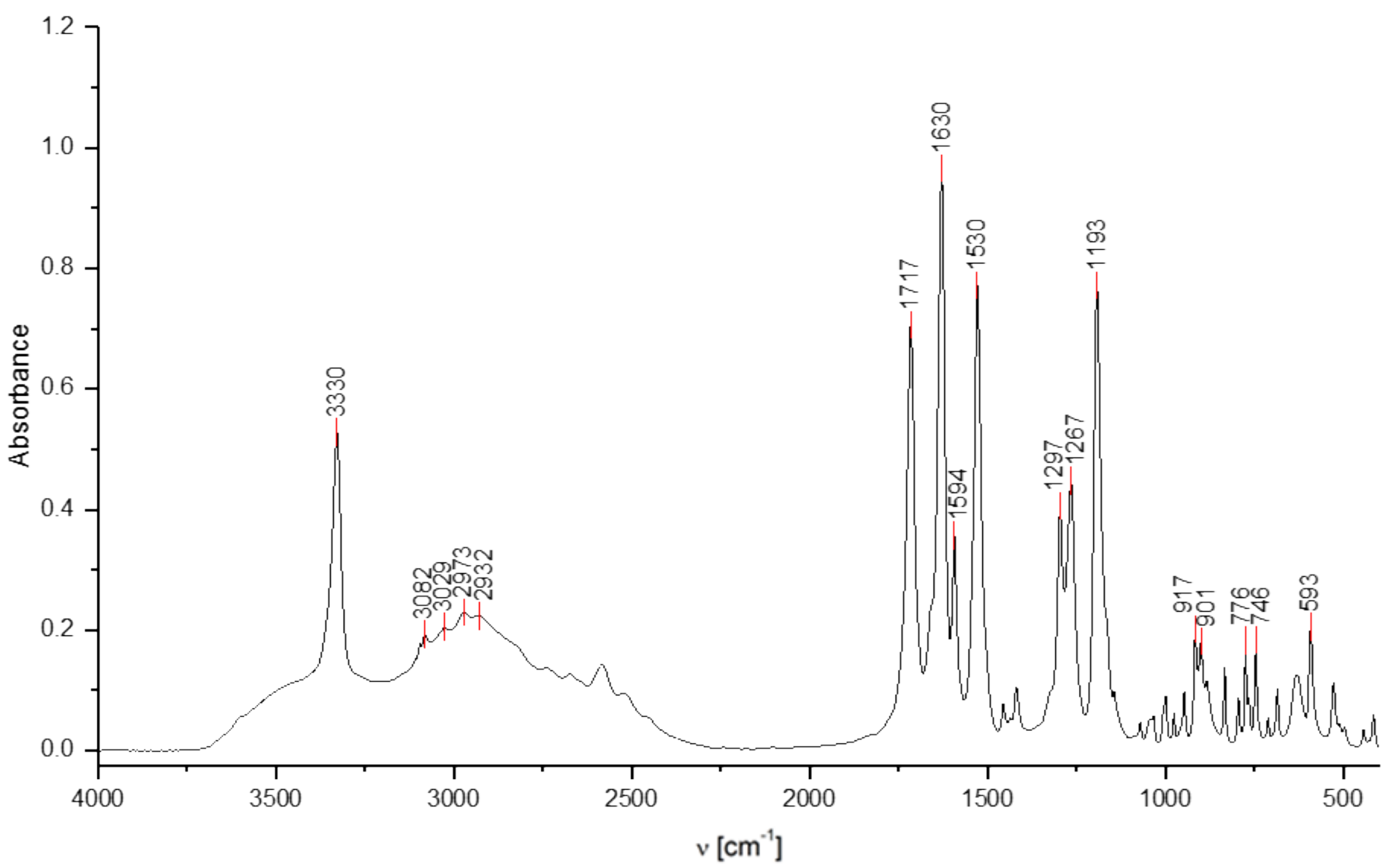

Figure S98. *FT-IR spectrum of **3** in KBr pellet recorded at 298 K.*

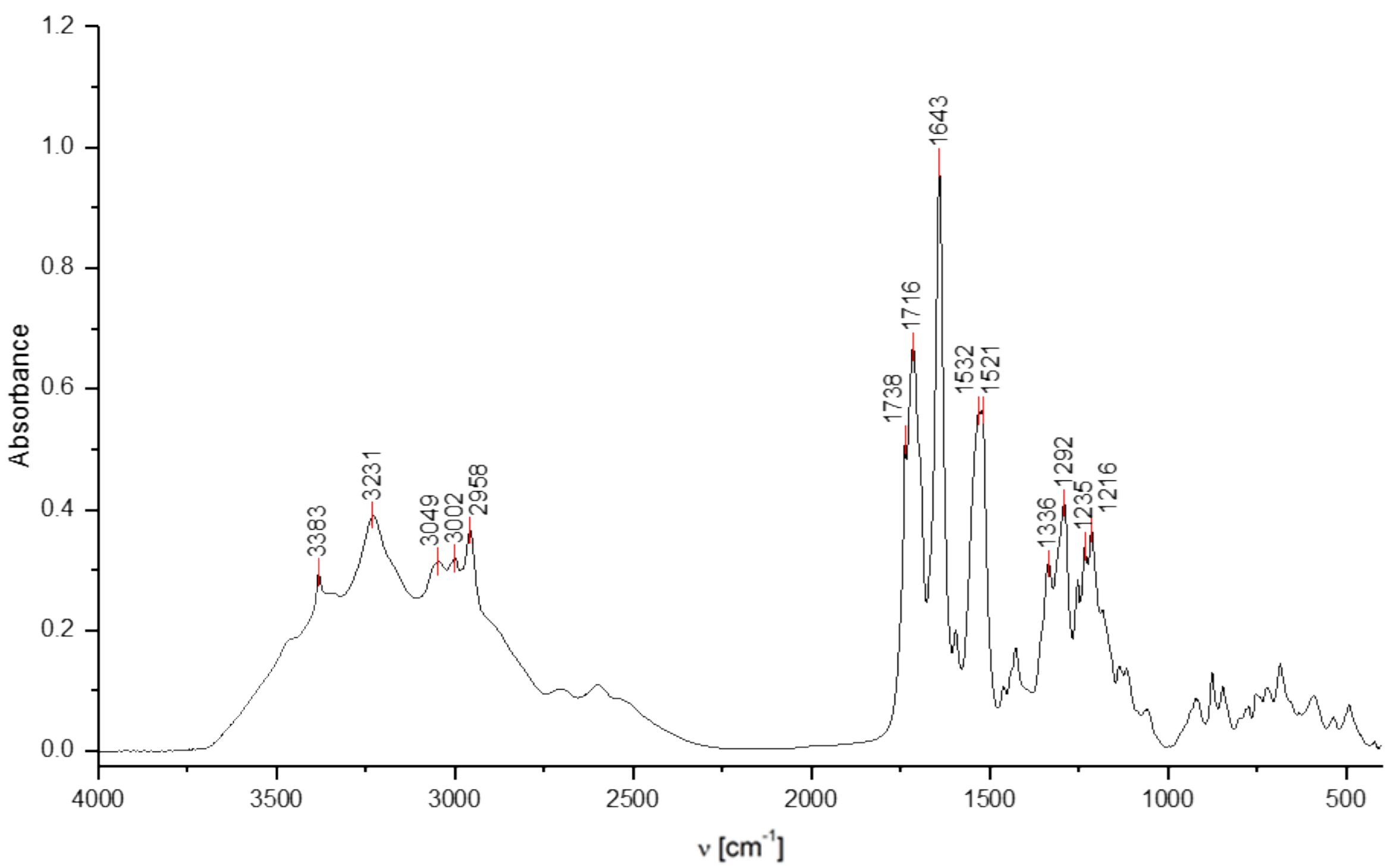

Figure S99. *FT-IR spectrum of **4** in KBr pellet recorded at 298 K.*

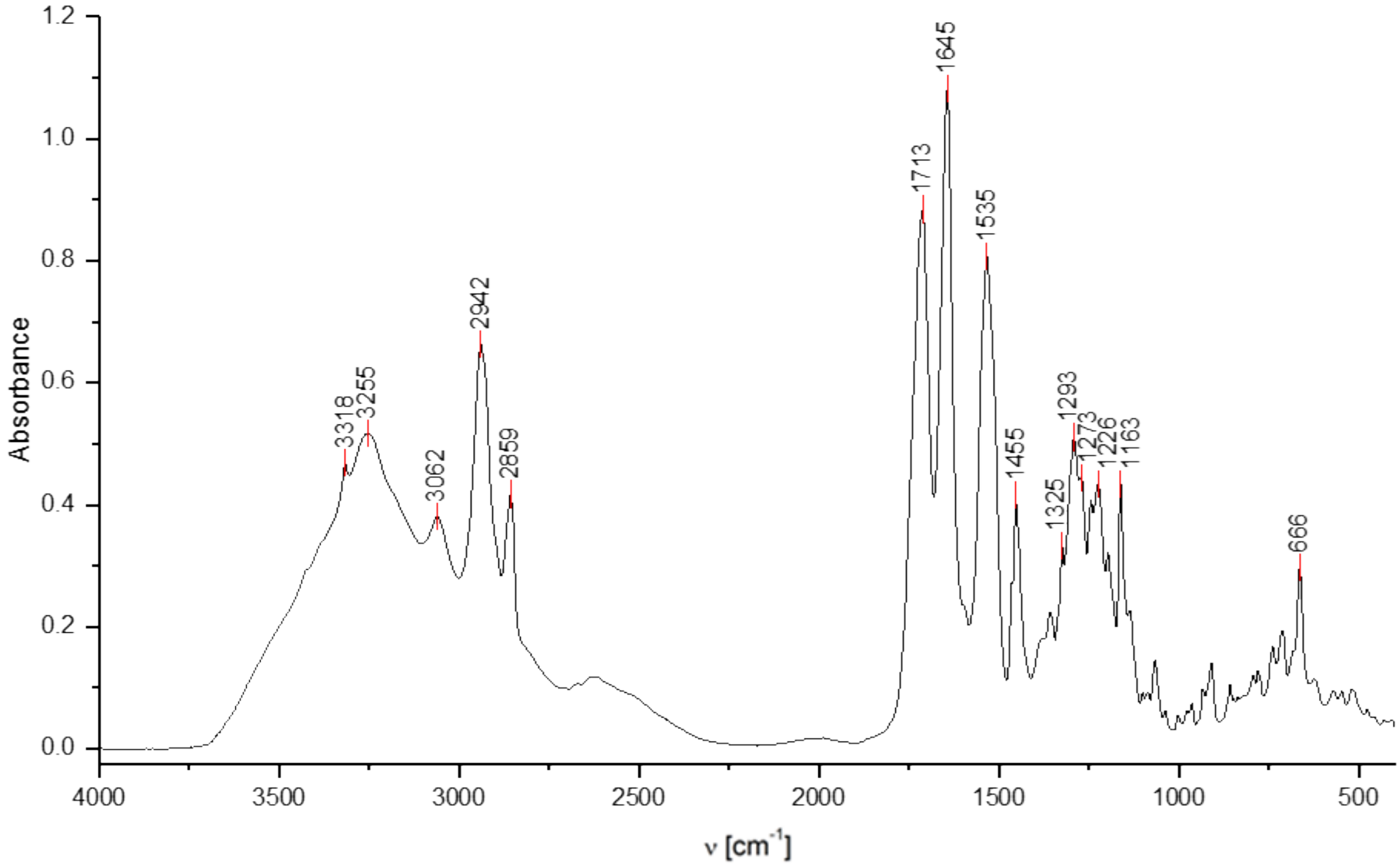

Figure S100. *FT-IR spectrum of* ***5*** *in KBr pellet recorded at 298 K.*

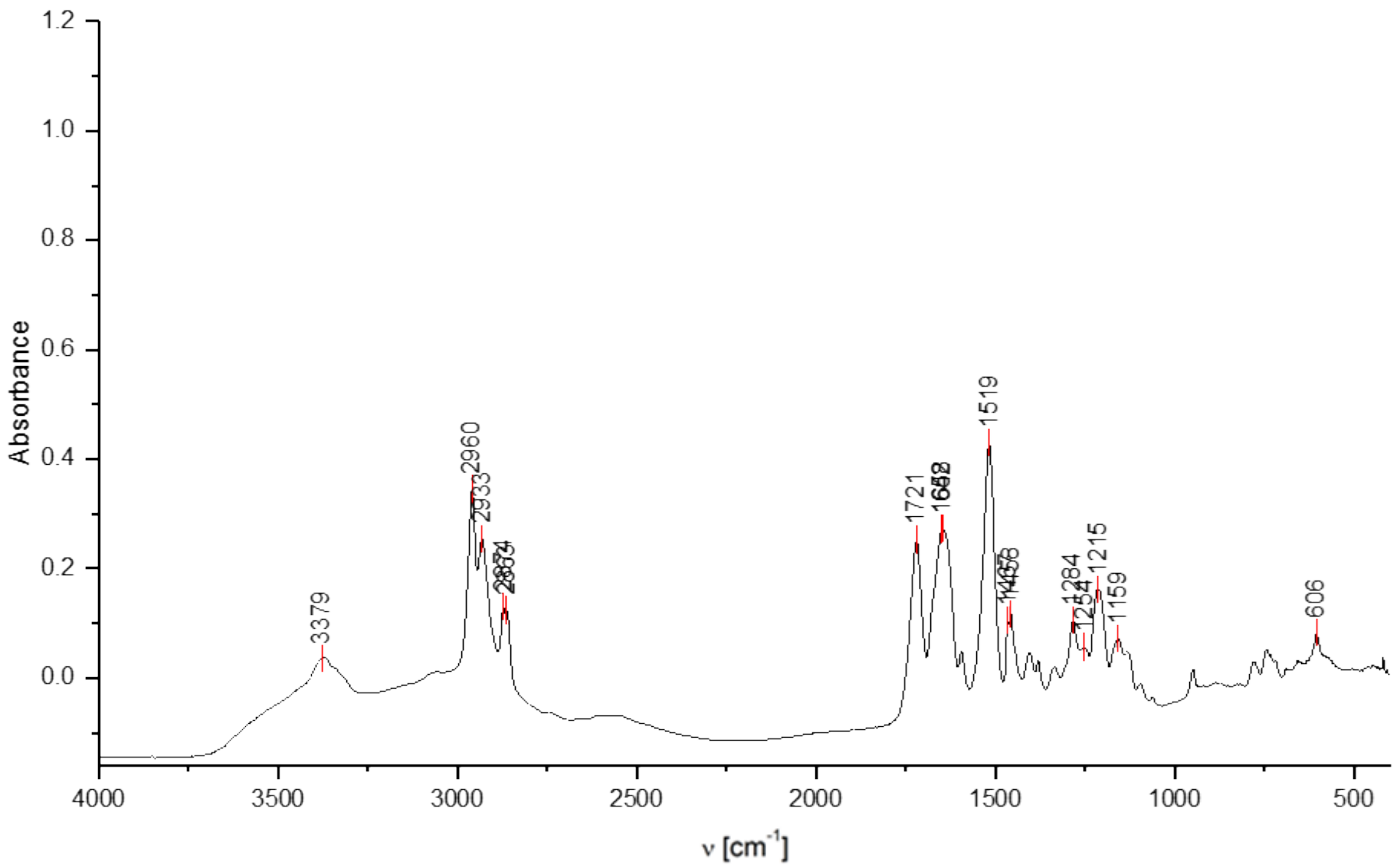

Figure S101. *FT-IR spectrum of* ***6*** *in KBr pellet recorded at 298 K.*

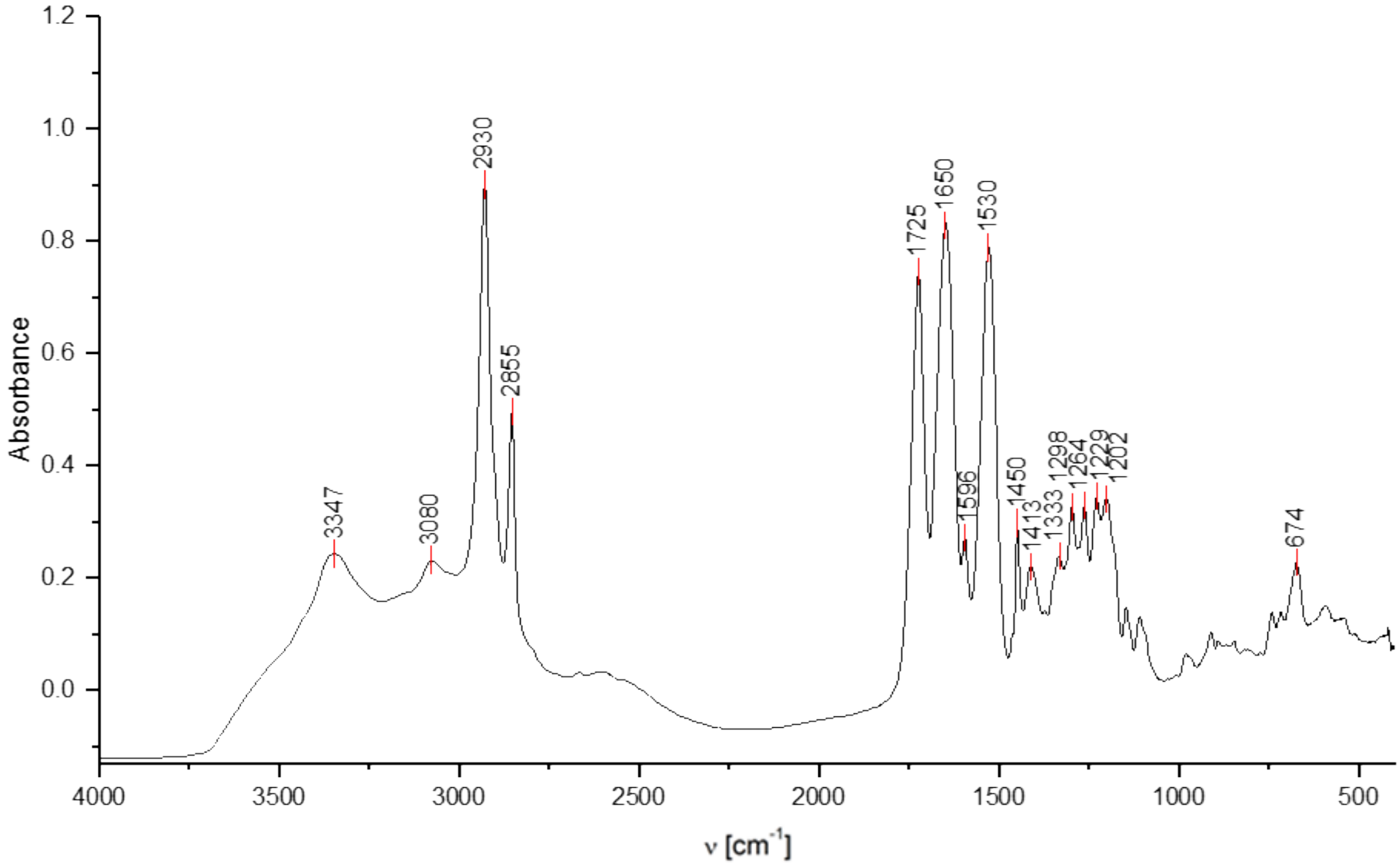

Figure S102. *FT-IR spectrum of **7** in KBr pellet recorded at 298 K.*

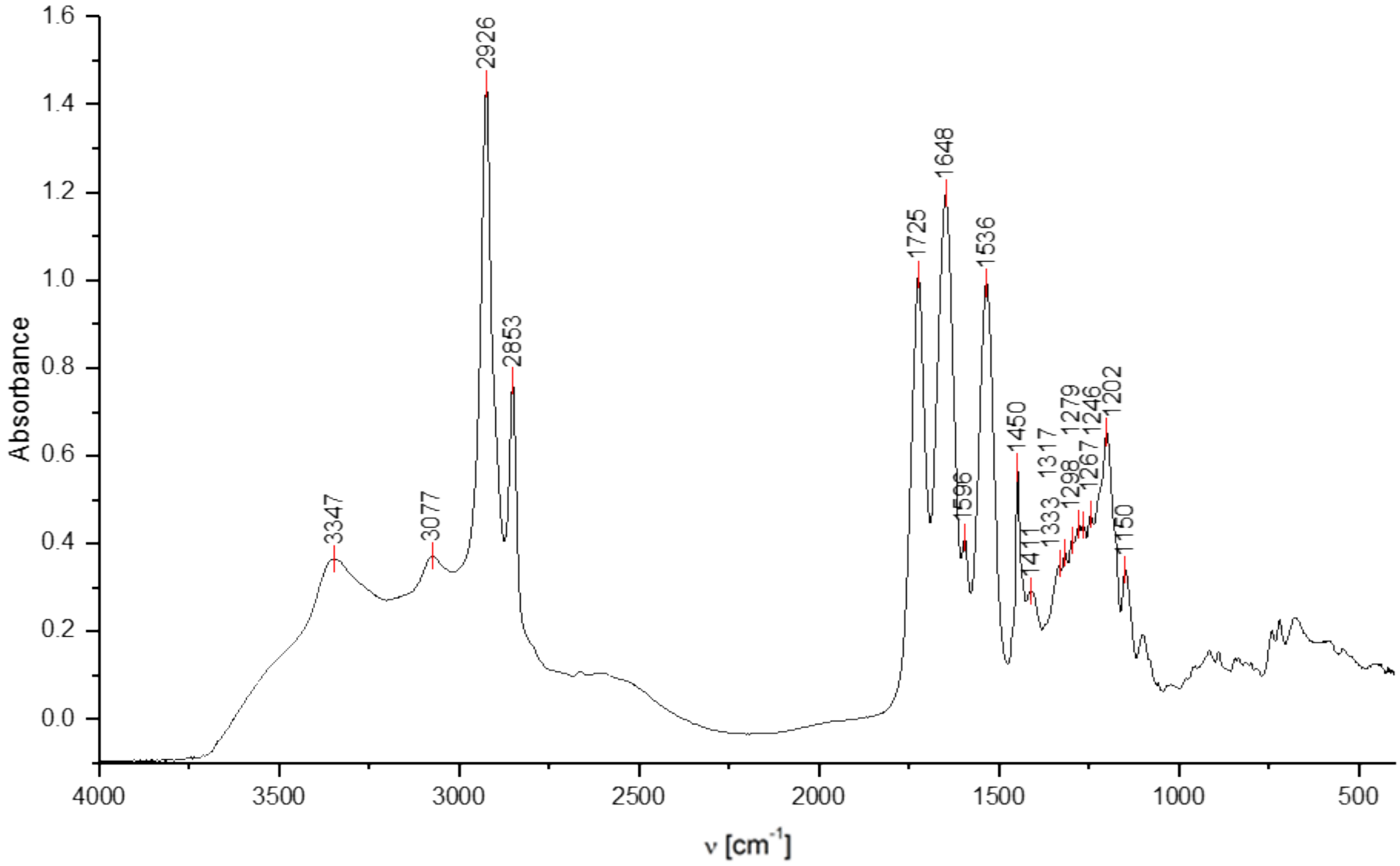

Figure S103. *FT-IR spectrum of **8** in KBr pellet recorded at 298 K.*

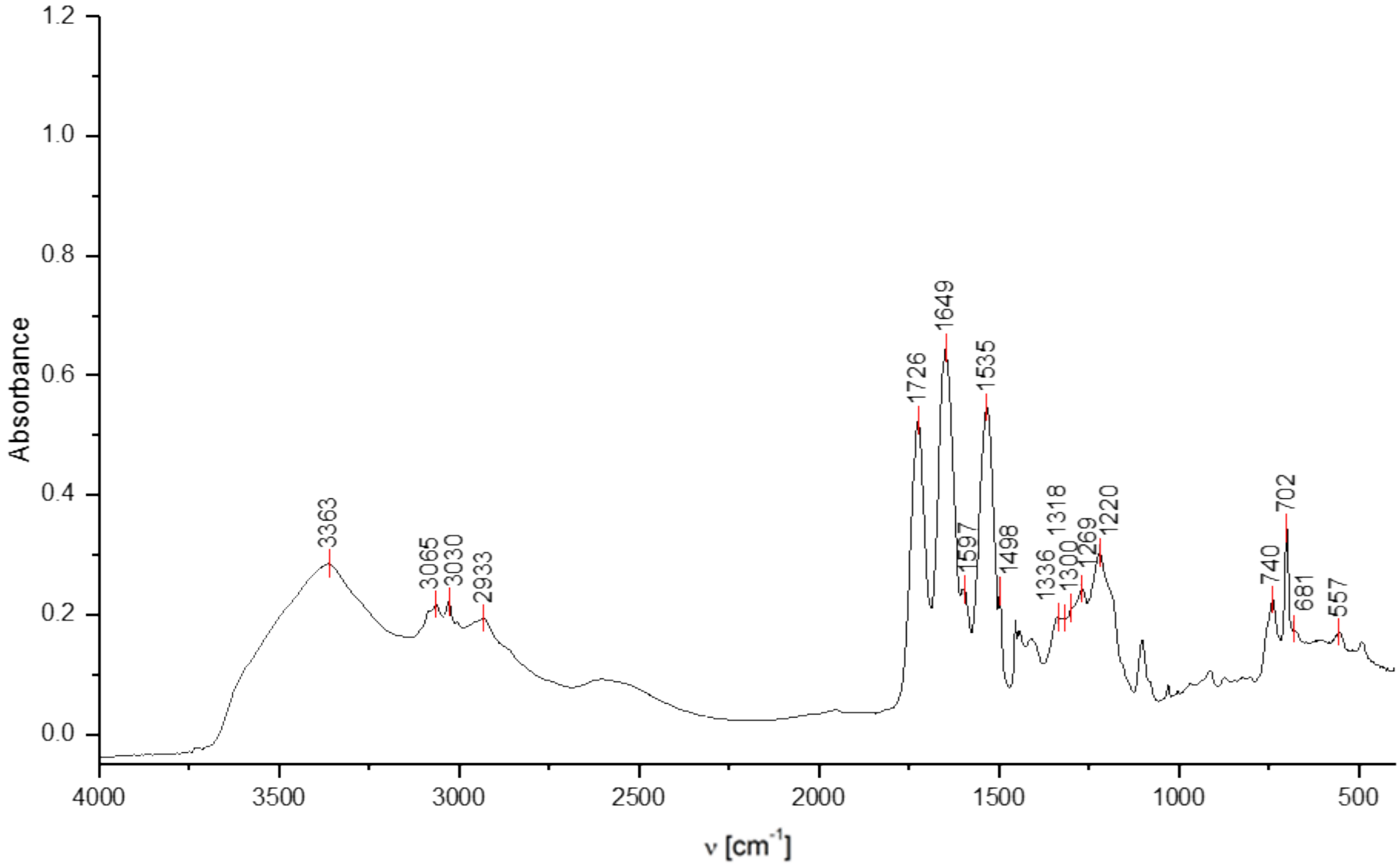

Figure S104. *FT-IR spectrum of **9** in KBr pellet recorded at 298 K.*

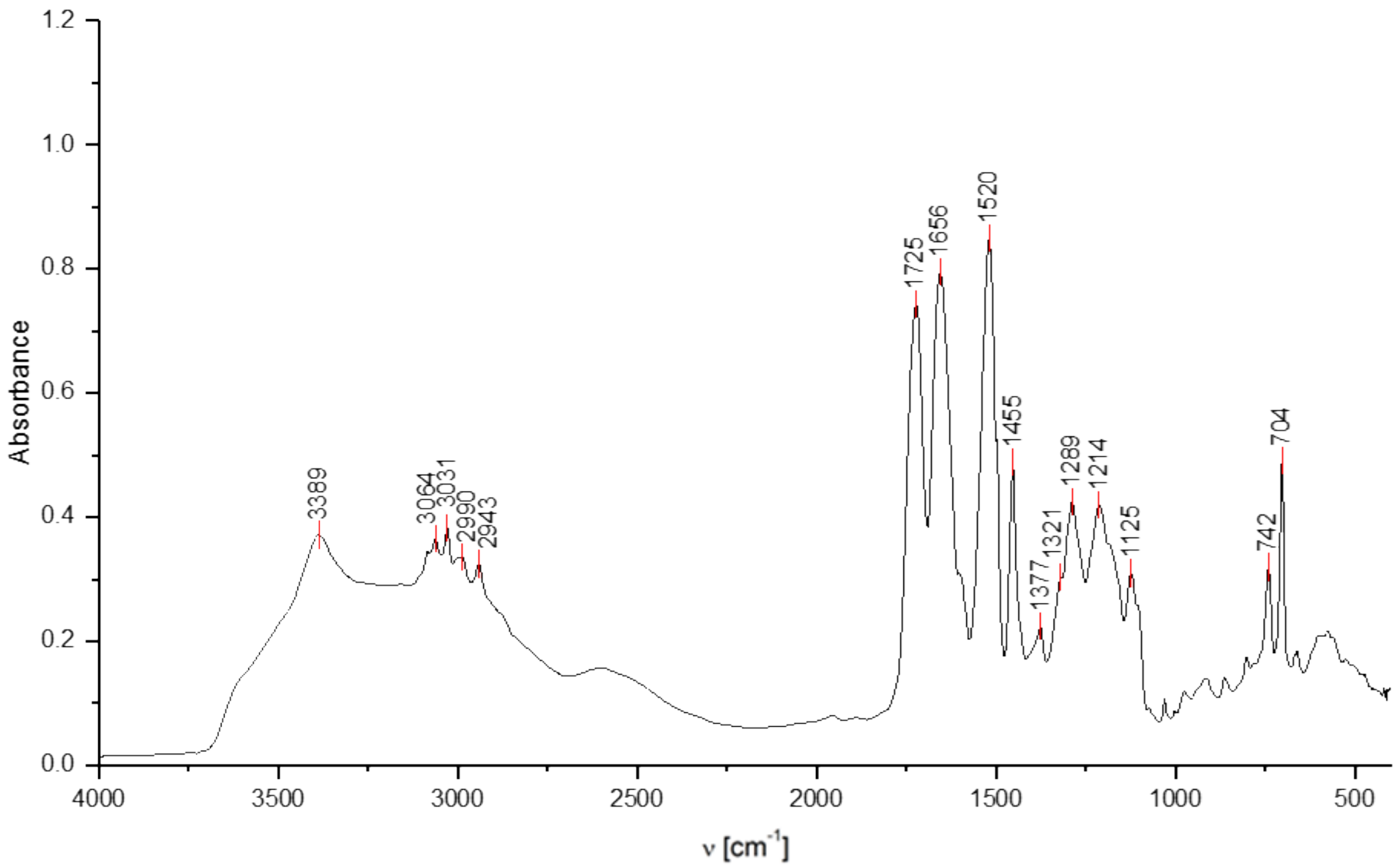

Figure S105. *FT-IR spectrum of **10** in KBr pellet recorded at 298 K.*

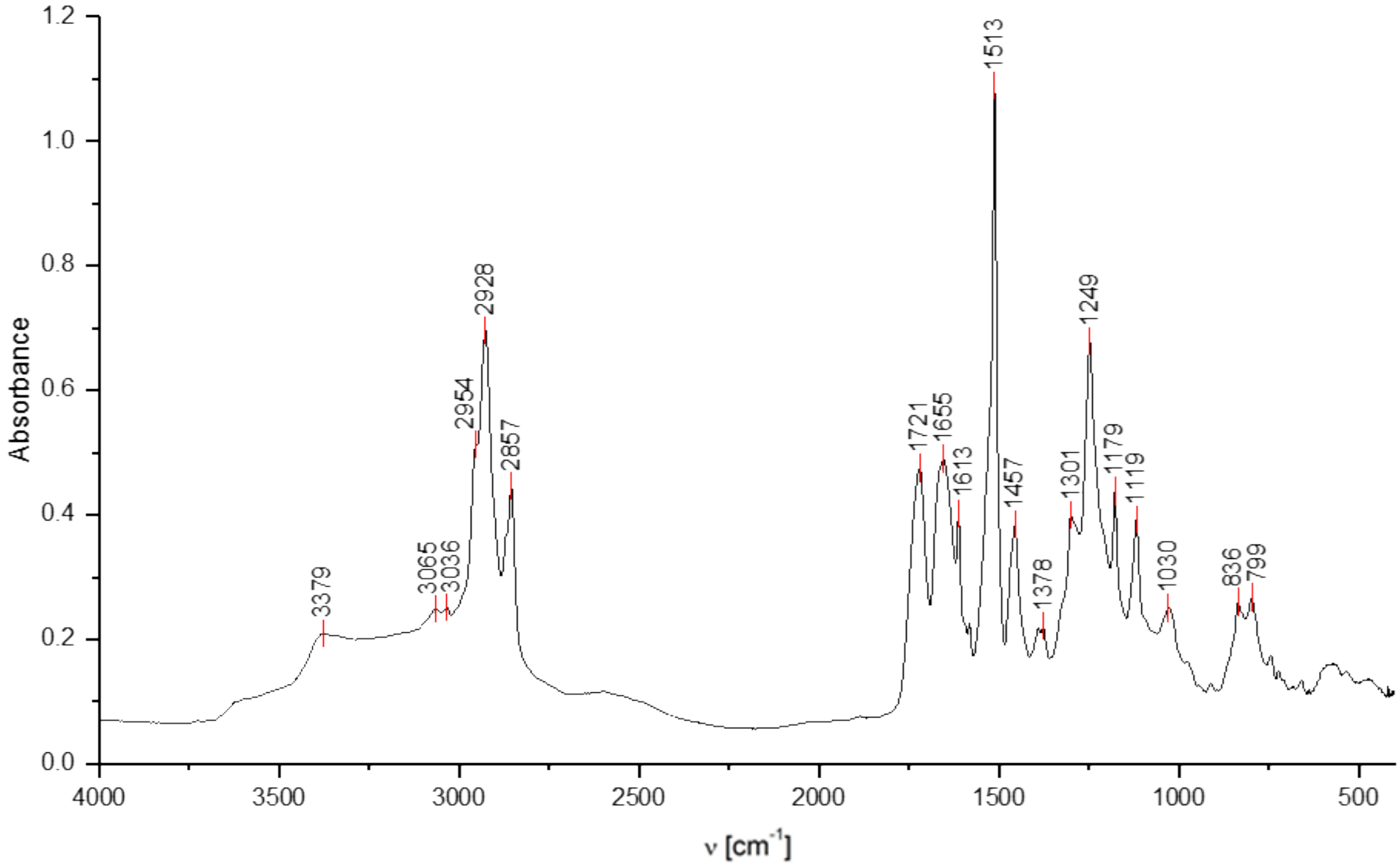

Figure S106. *FT-IR spectrum of **11** in KBr pellet recorded at 298 K.*

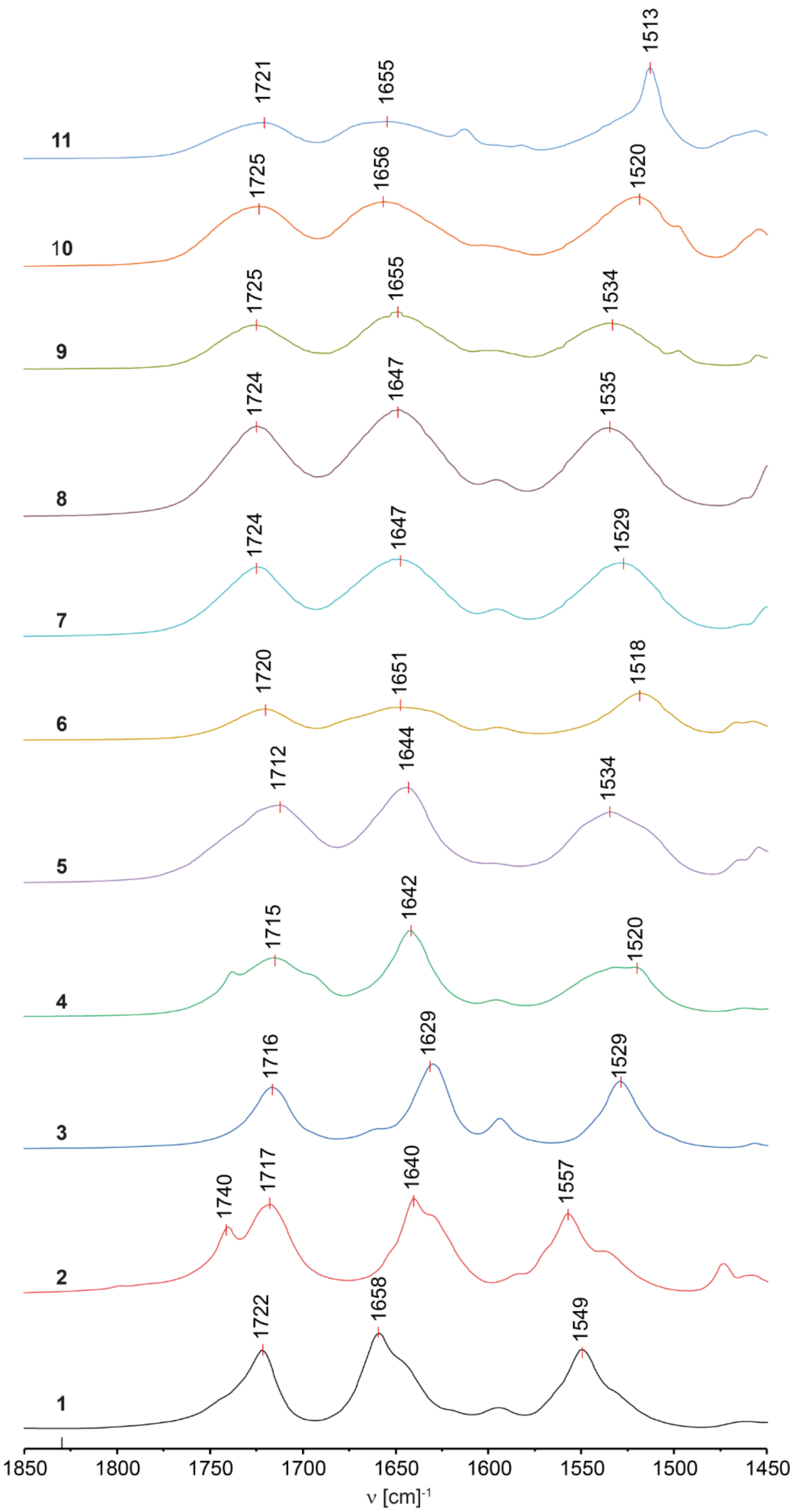

Figure S107. *FT-IR spectra **1-11** in KBr pellet recorded at 298 K.*

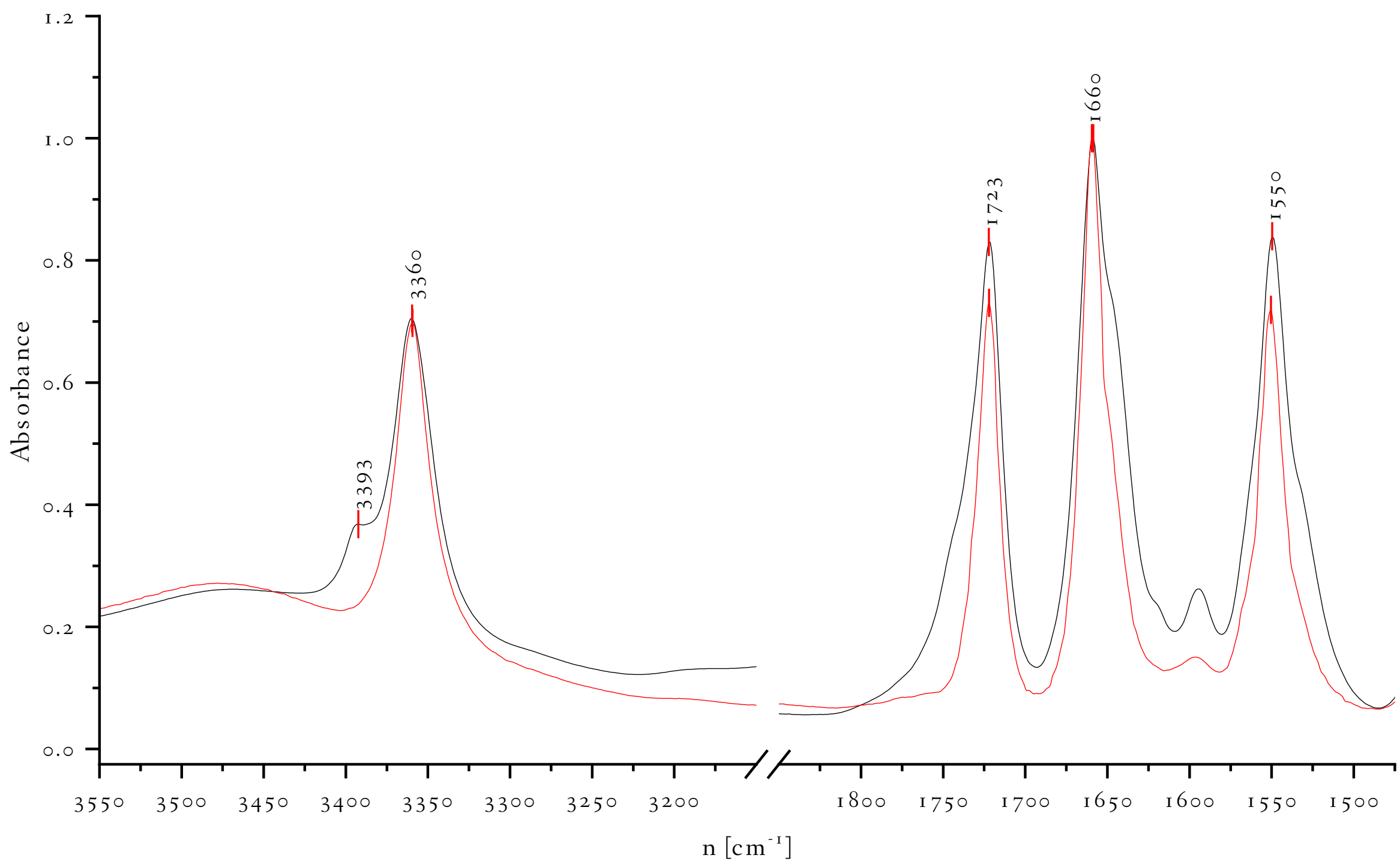

Figure S108. *FT-IR spectra of* ***1*** *(black) and* ***1·3H₂O*** *(red) in KBr pellet recorded at 298 K.*

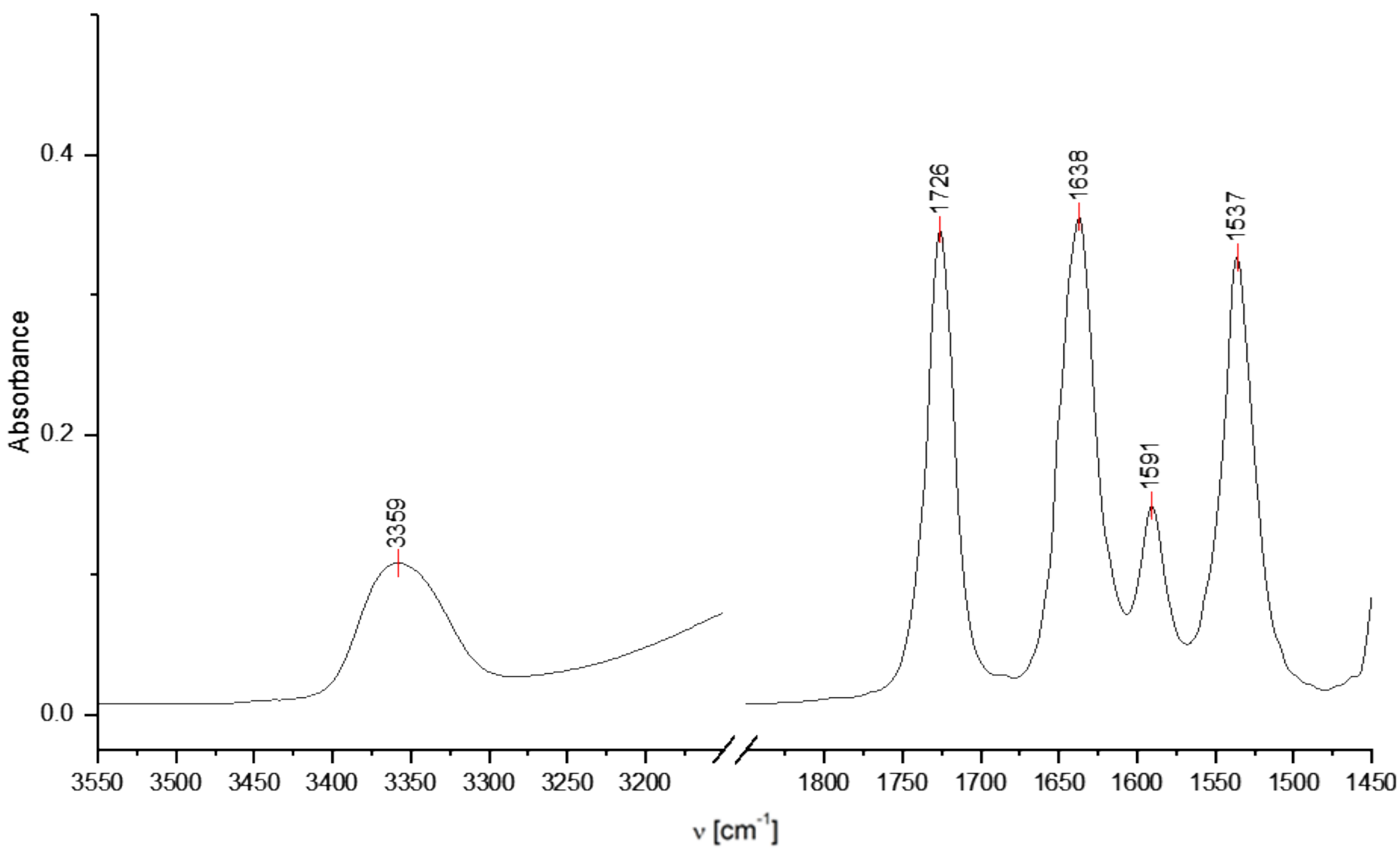

Figure S109. *FT-IR spectrum of* ***7*** *in 1,1,2,2-tetrachloroethane ($C = 1.0 \times 10^{-2}$ M).*

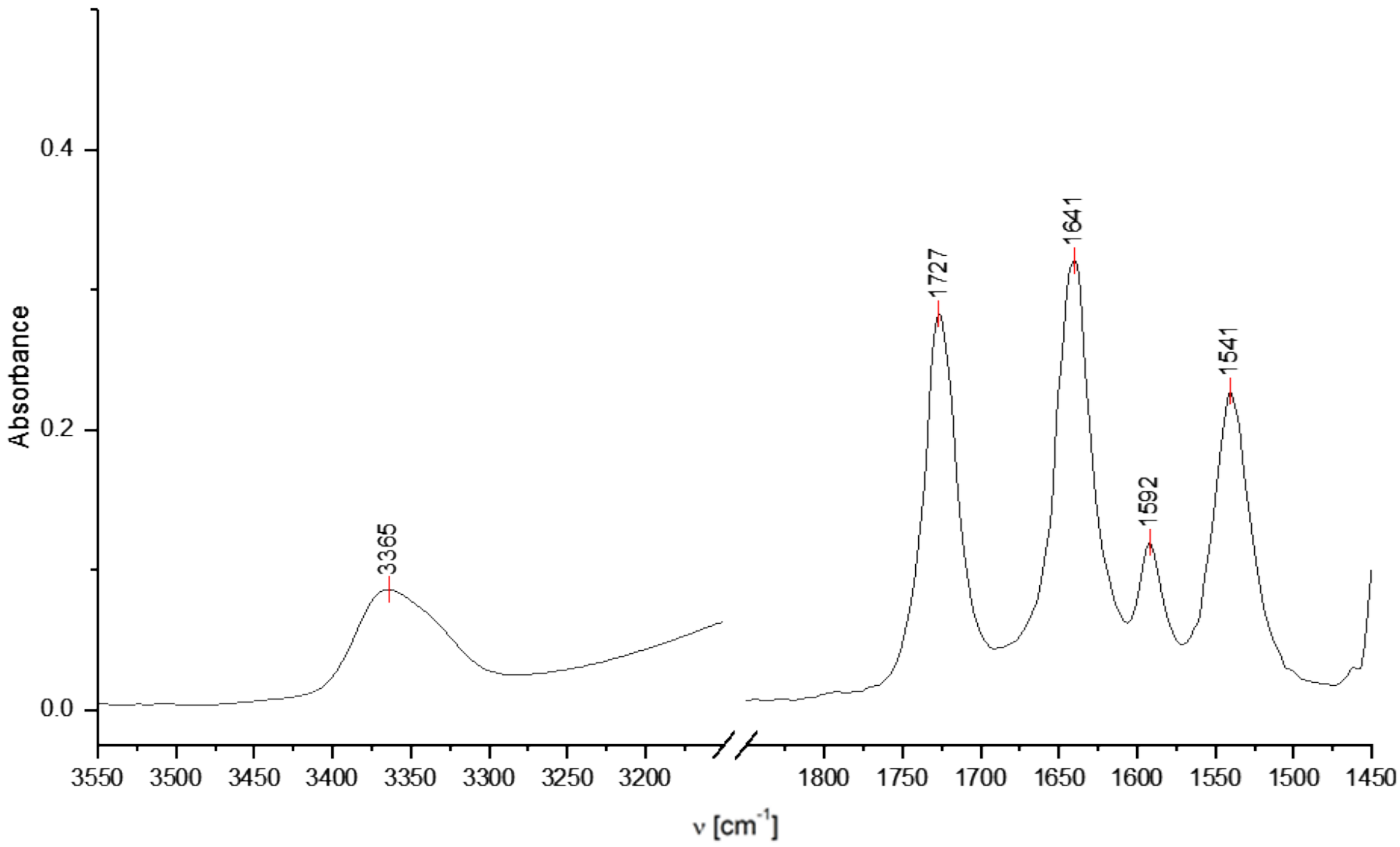

Figure S110. *FT-IR spectrum of* ***8*** *in 1,1,2,2-tetrachloroethane (C = 1.0 × 10*$^{-2}$ *M).*

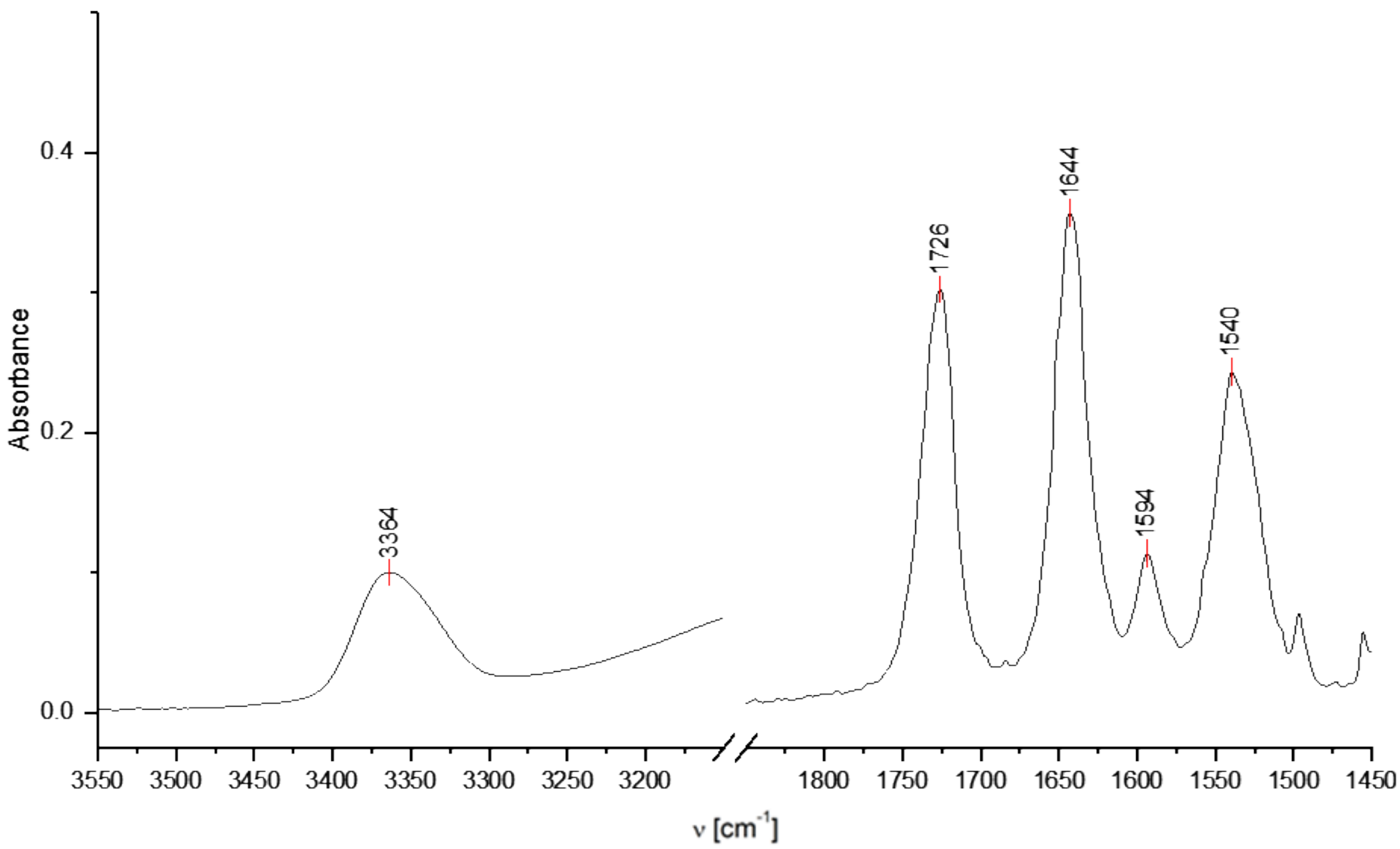

Figure S111. *FT-IR spectrum of* ***9*** *in 1,1,2,2-tetrachloroethane (C = 1.0 × 10*$^{-2}$ *M).*

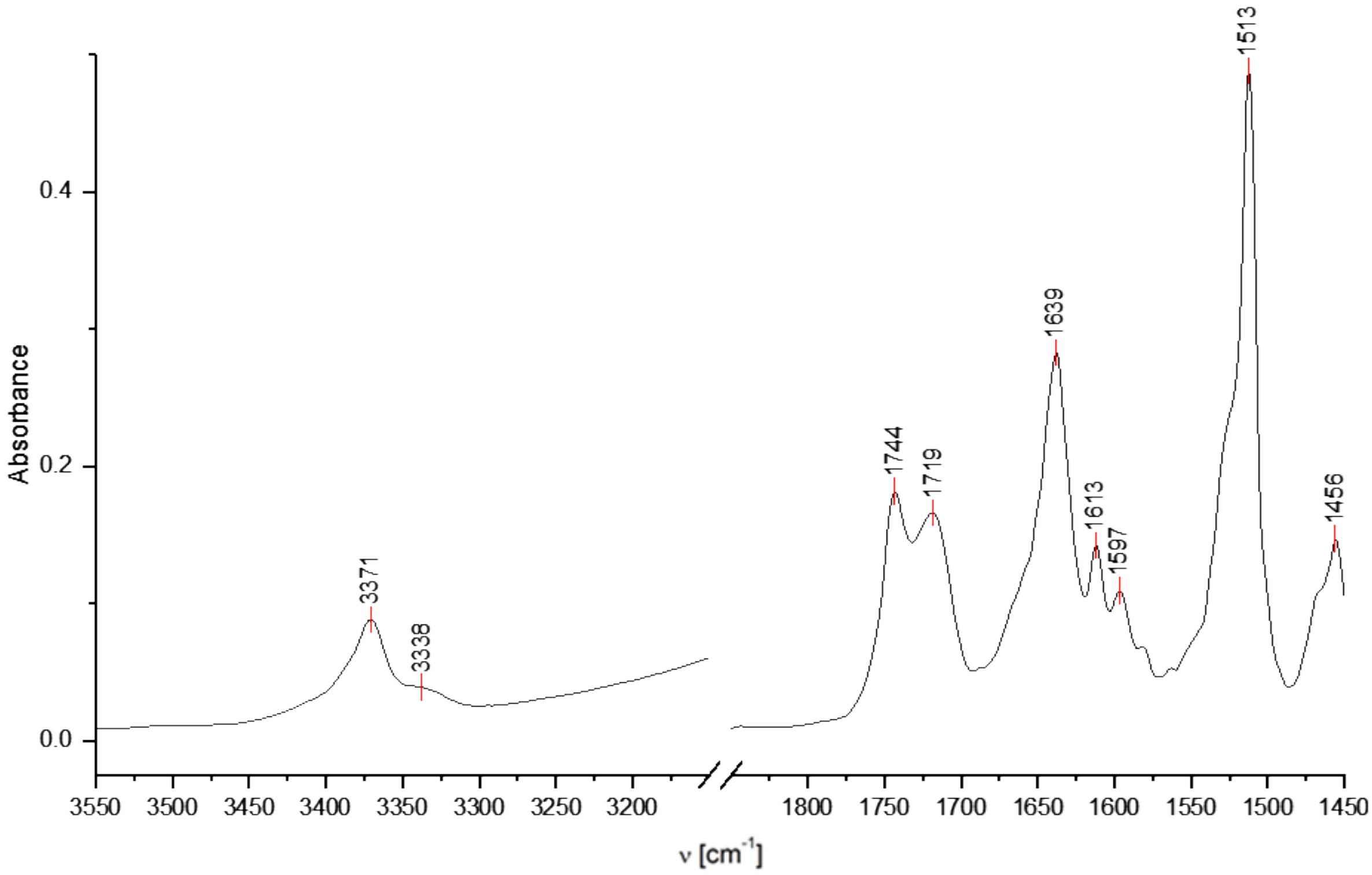

Figure S112. *FT-IR spectrum of* ***11*** *in 1,1,2,2-tetrachloroethane (C = 1.0 × 10⁻² M).*

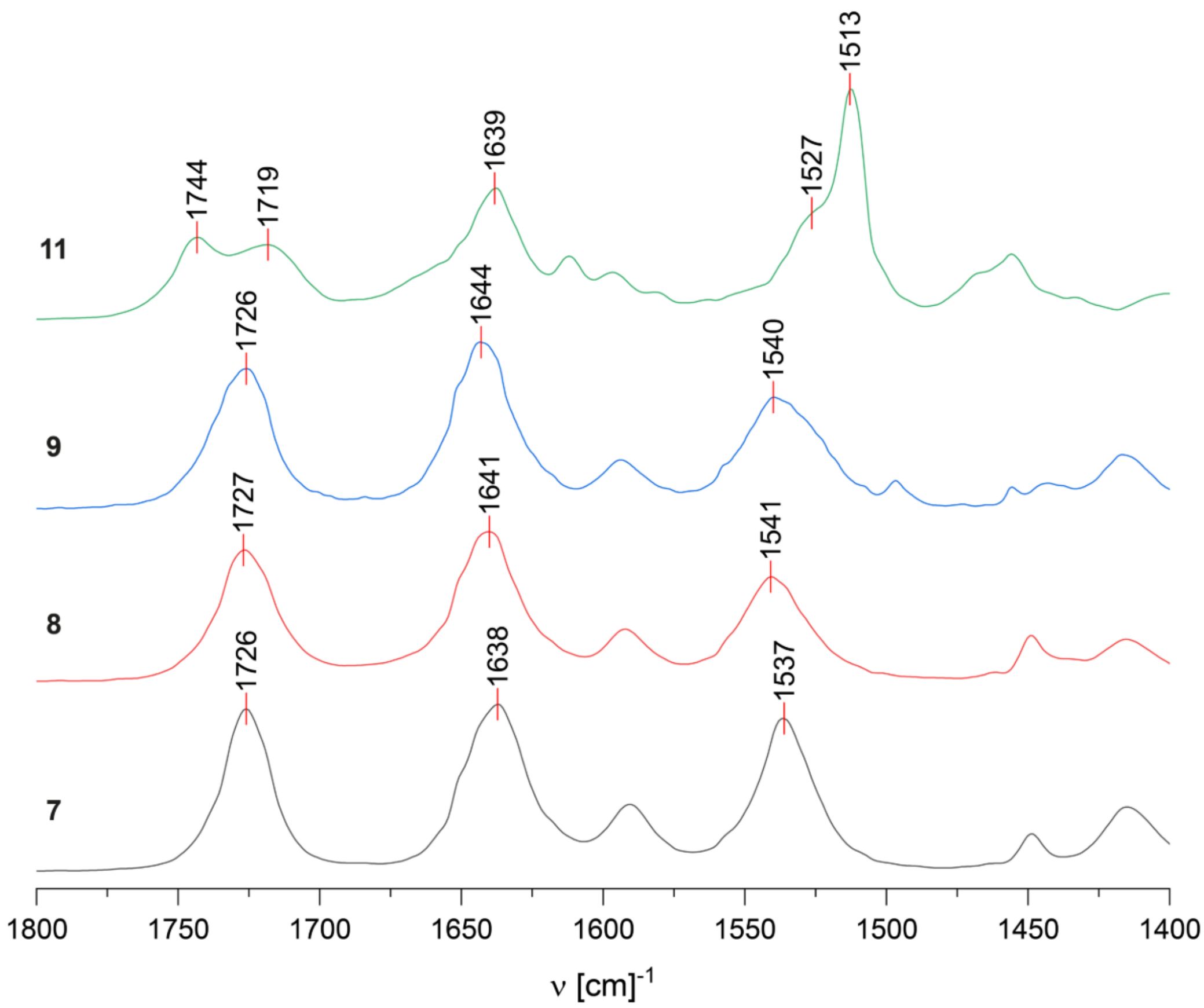

Figure S113. *FTFT-IR spectra of* ***7, 8, 9*** *and* ***11*** *in 1,1,2,2-tetrachloroethane (C = 1.0 × 10⁻² M).*

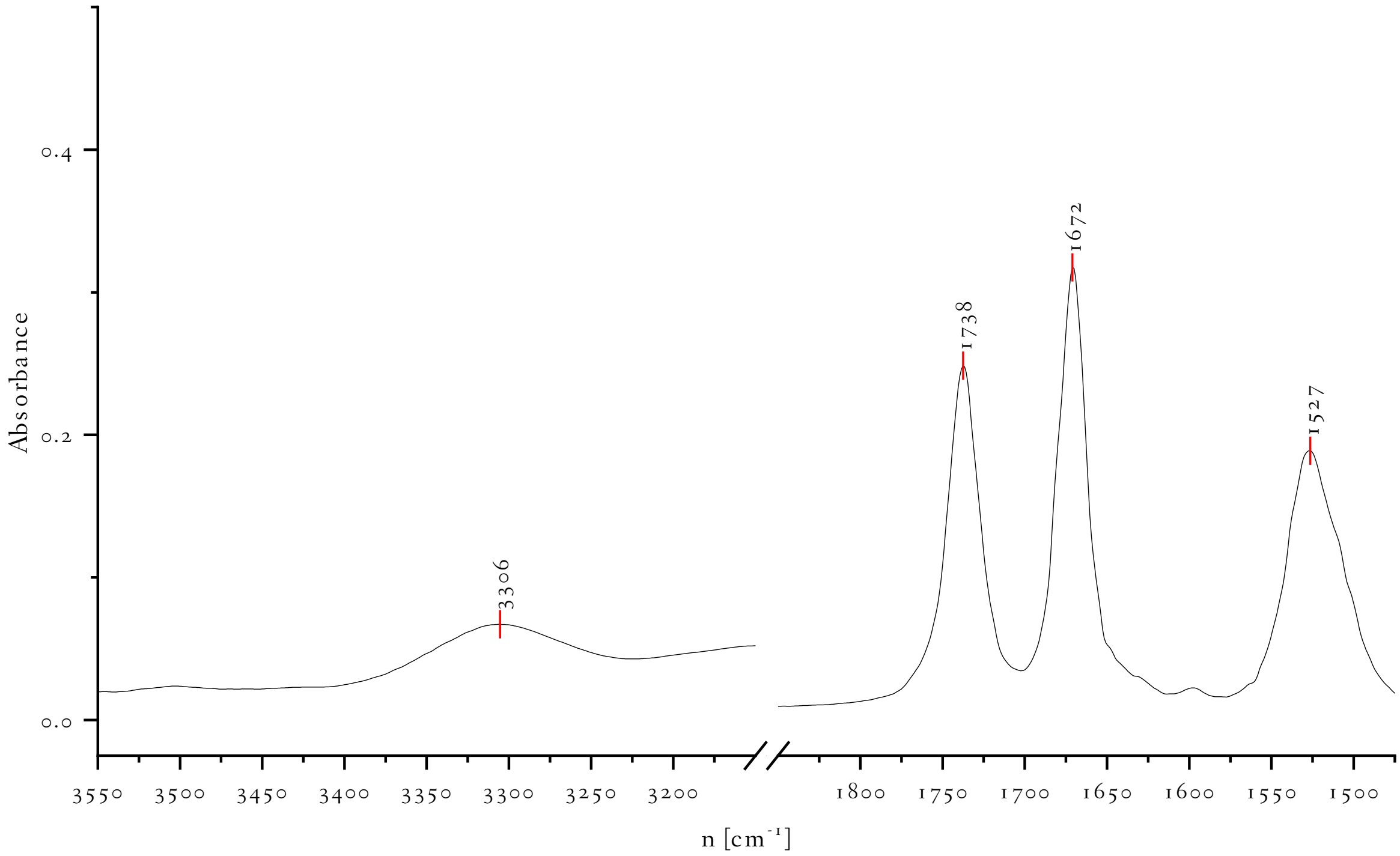

Figure S114. *FT-IR spectrum of **7** in tetrahydrofuran (C = 1.0 × 10^{-2} M).*

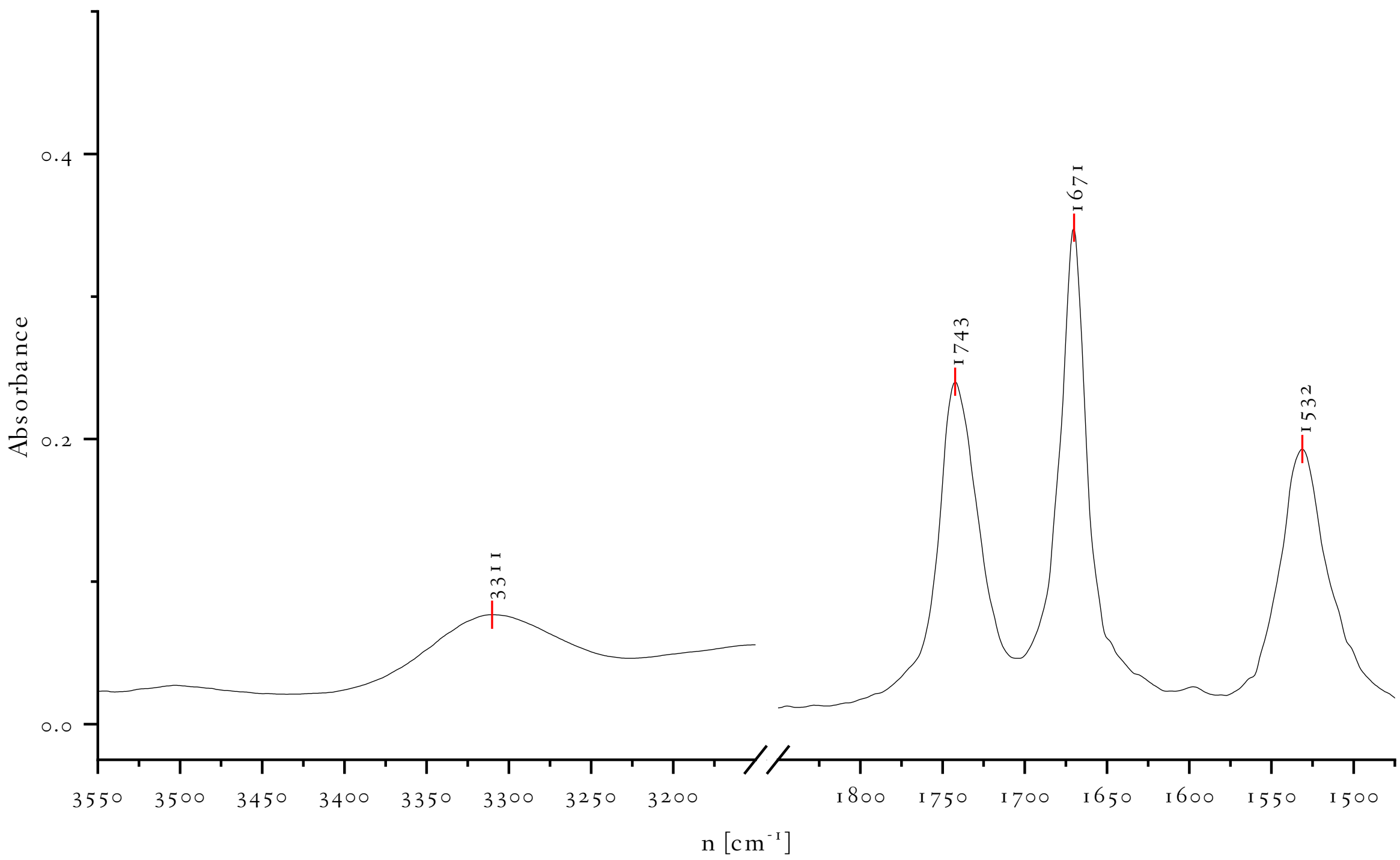

Figure S115. *FT-IR spectrum of **8** in tetrahydrofuran (C = 1.0 × 10^{-2} M).*

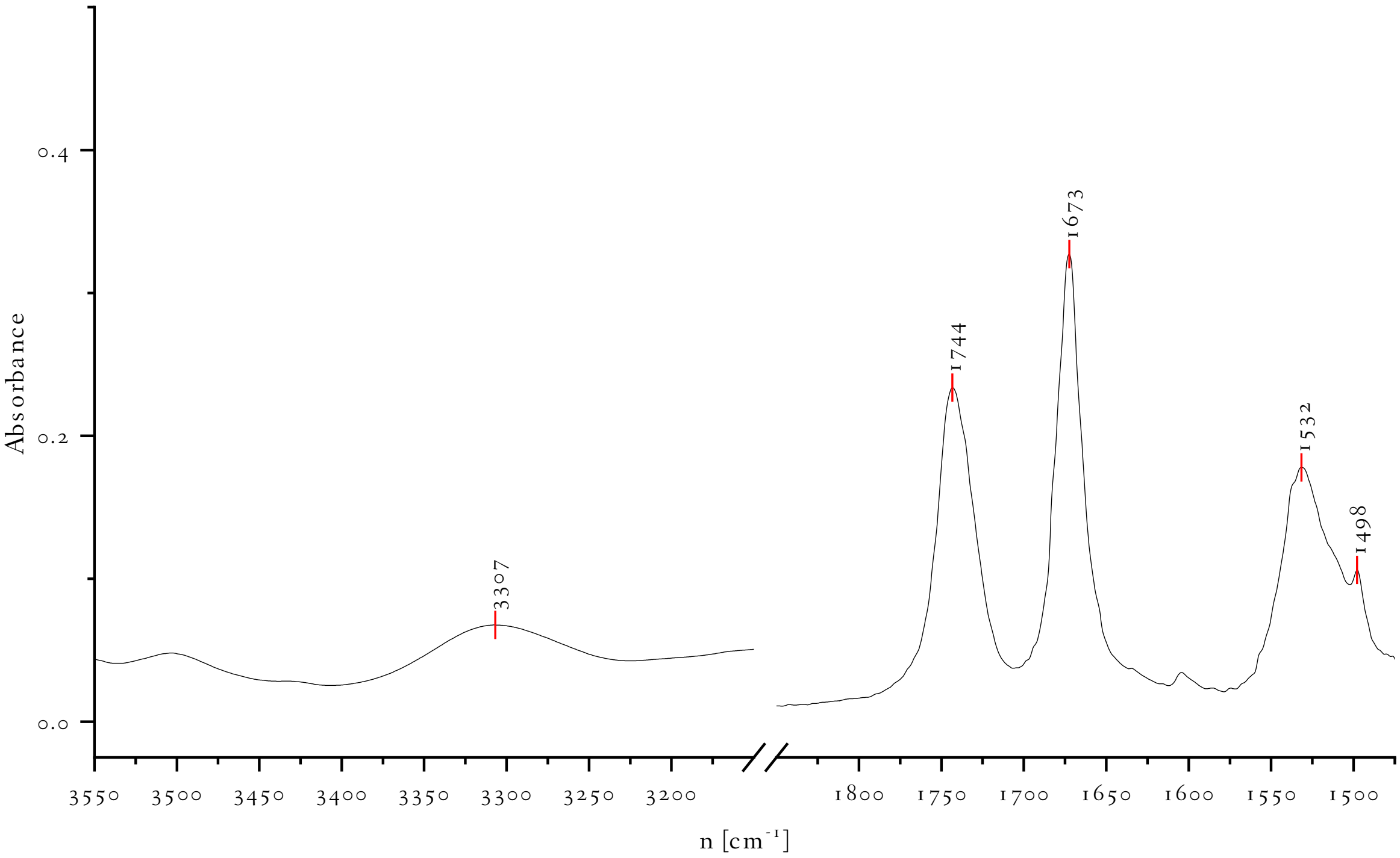

Figure S116. *FT-IR spectrum of **9** in tetrahydrofuran (C = 1.0 × 10⁻² M).*

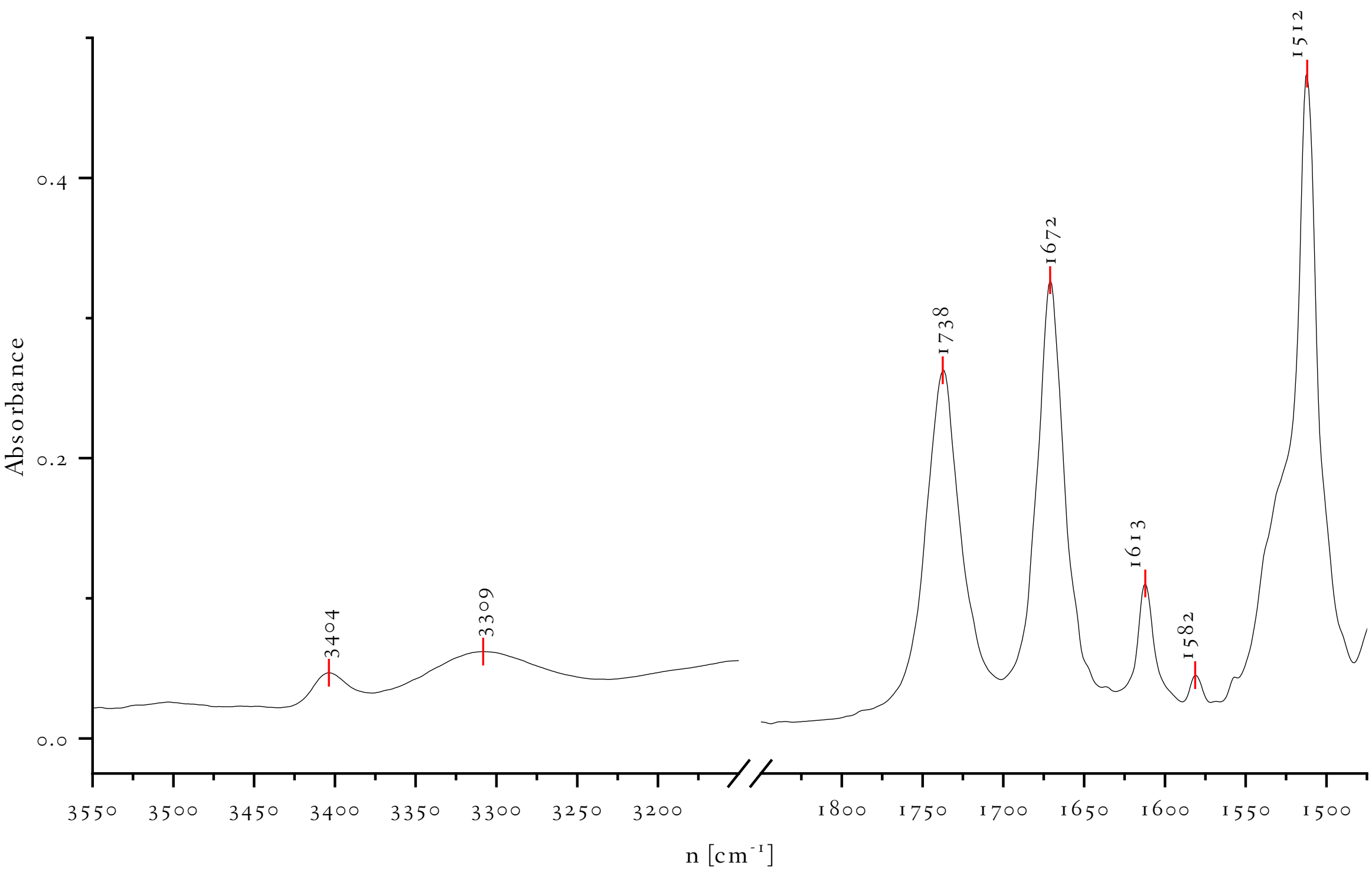

Figure S117. *FT-IR spectrum of **11** in tetrahydrofuran (C = 1.0 × 10^{-2} M).*

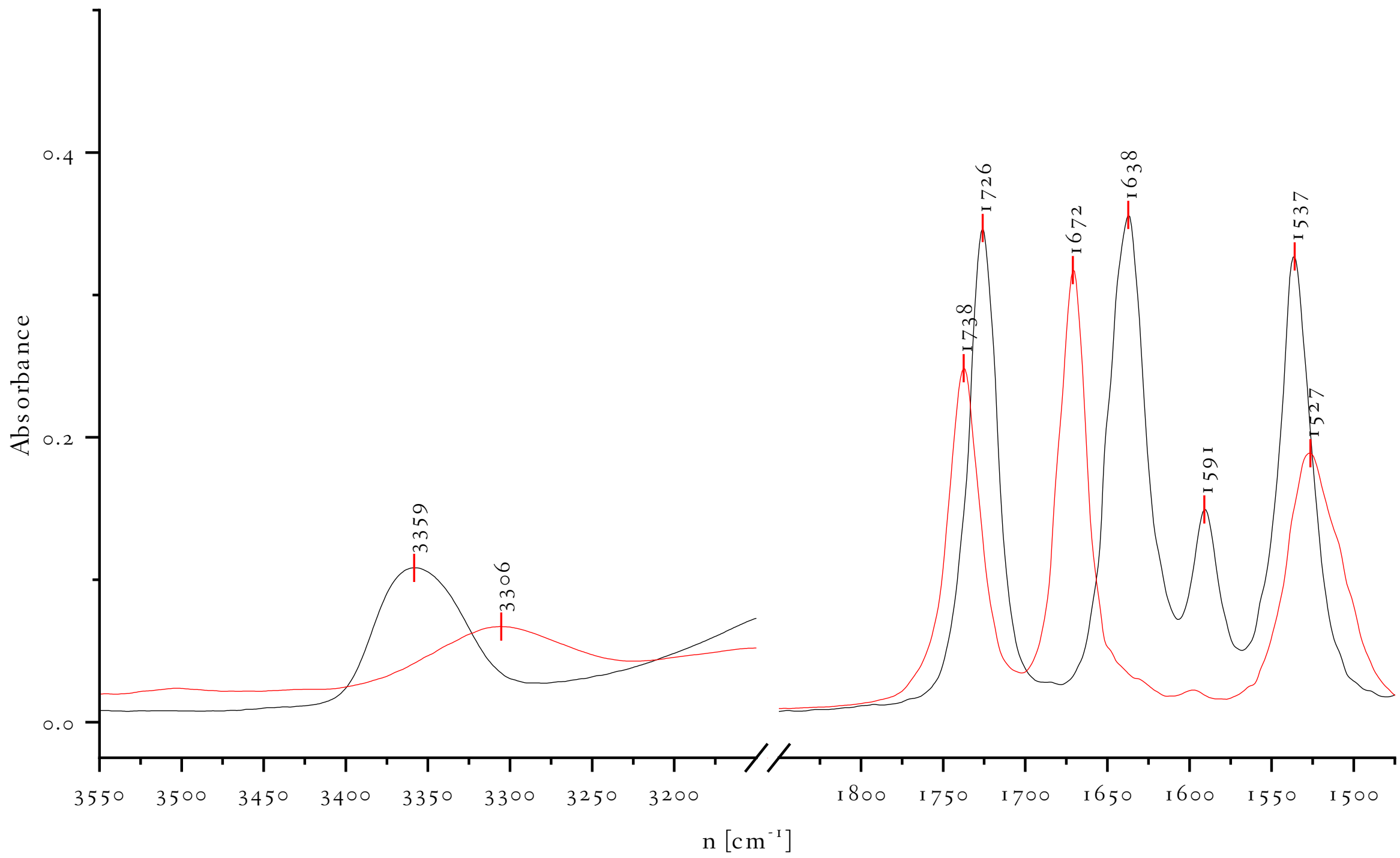

Figure S118. *FTFT-IR spectra of 7 in 1,1,2,2-tetrachloroethane (black) and tetrahydrofuran (red) ($C = 1.0 \times 10^{-2}$ M).*

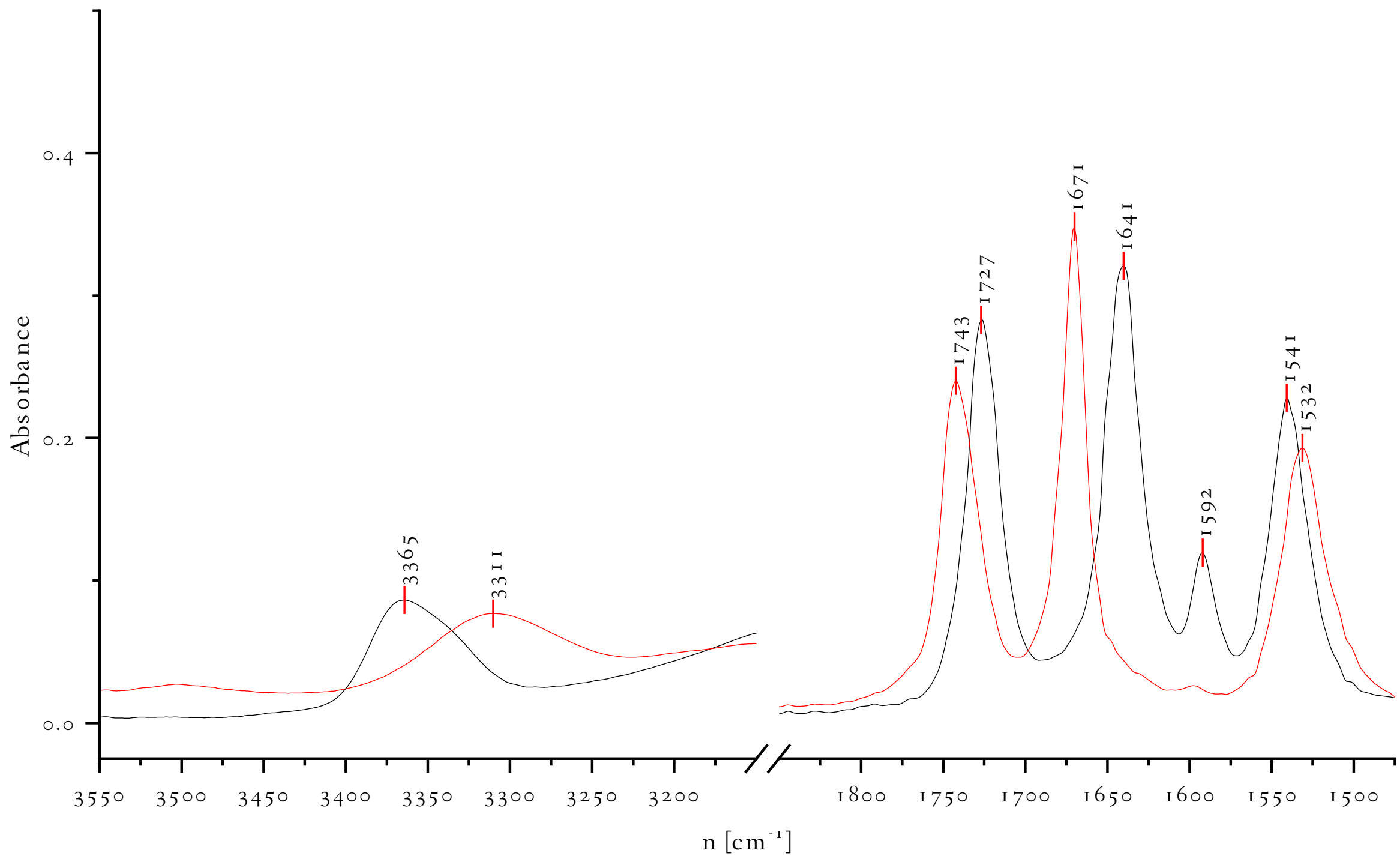

Figure S119. *FTFT-IR spectra of 8 in 1,1,2,2-tetrachloroethane (black) and tetrahydrofuran (red) ($C = 1.0 \times 10^{-2}$ M).*

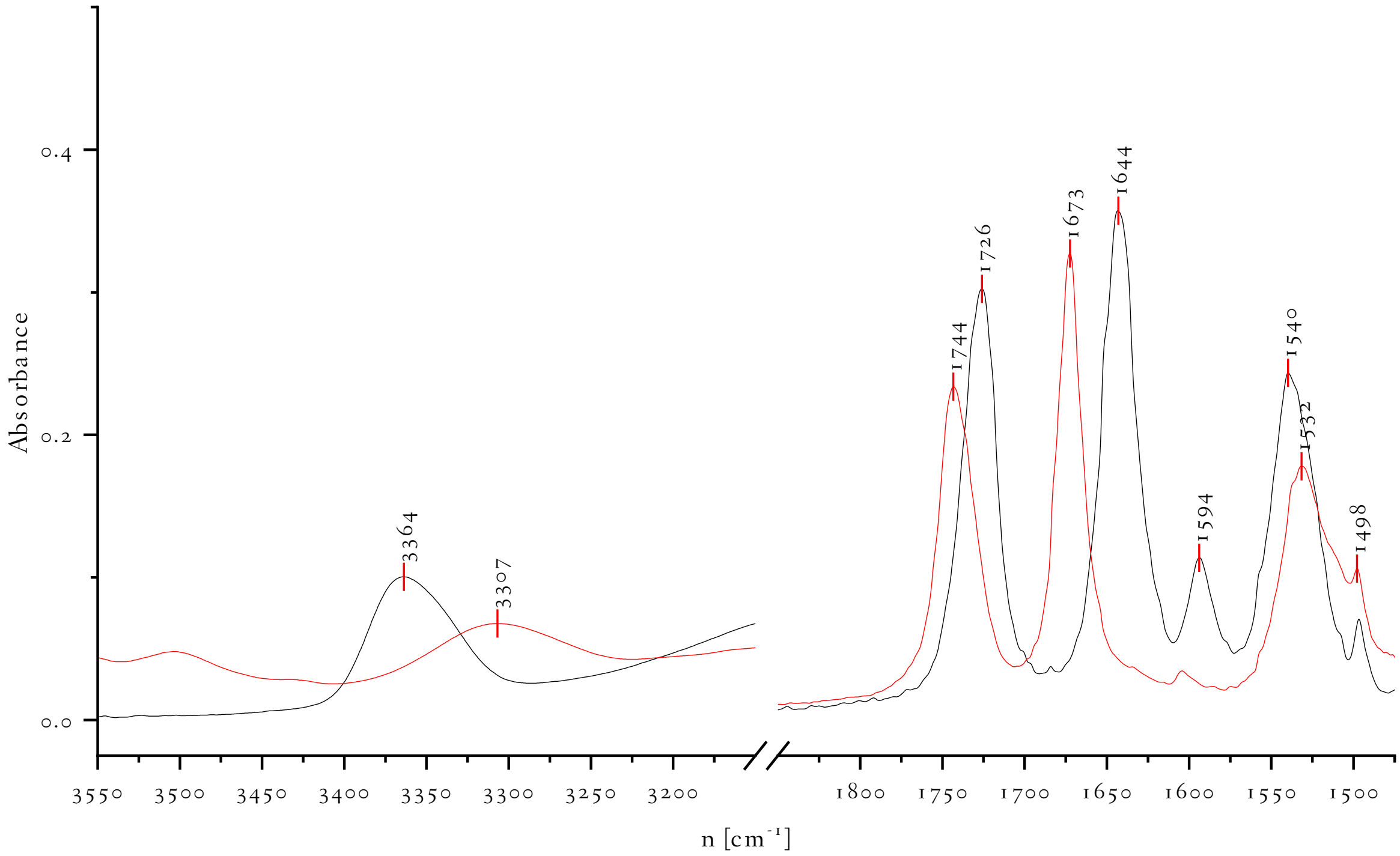

Figure S120. *FTFT-IR spectra of **9** in 1,1,2,2-tetrachloroethane (black) and tetrahydrofuran (red) ($C = 1.0 \times 10^{-2}$ M).*

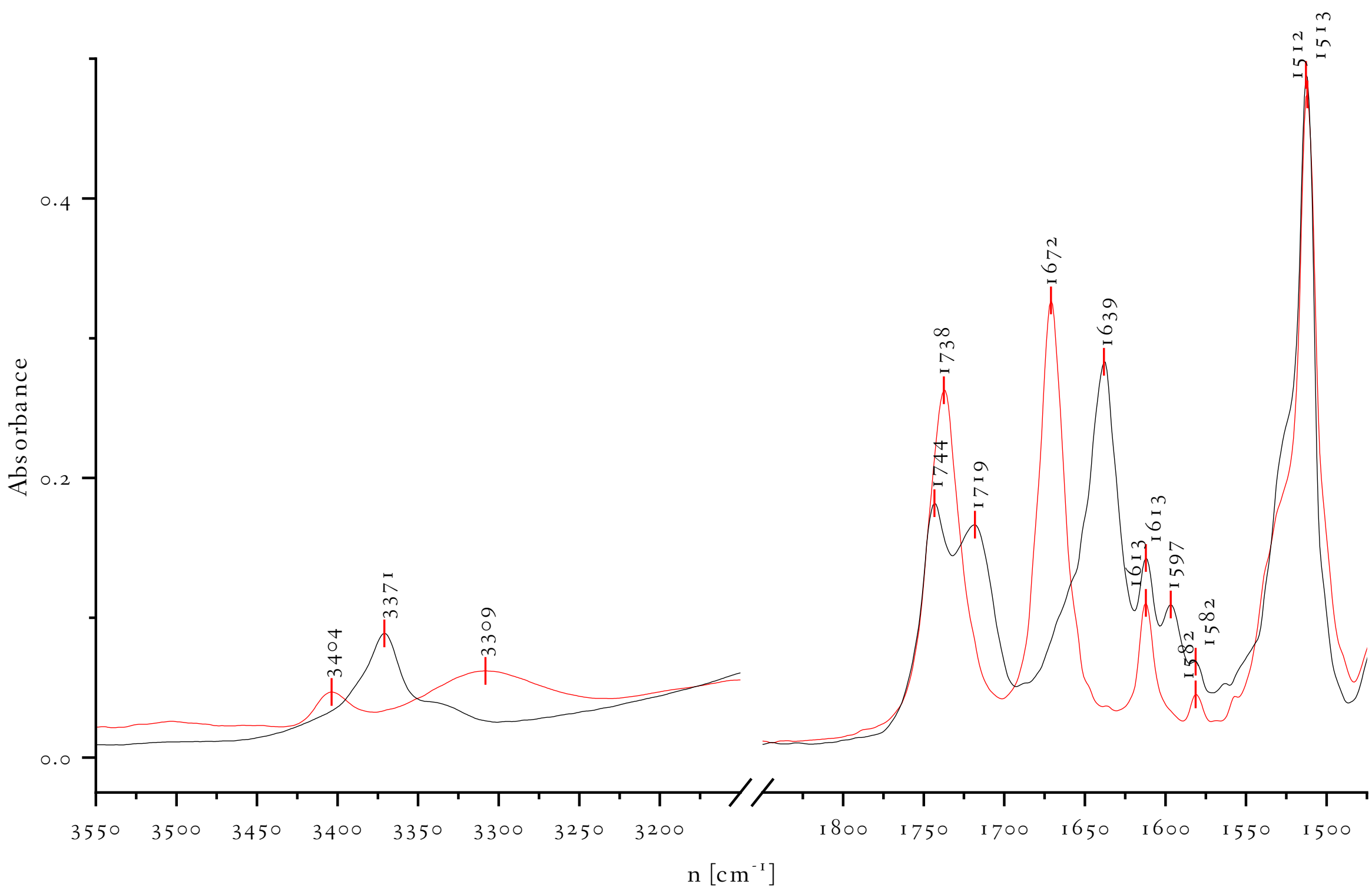

Figure S121. *FTFT-IR spectra of **11** in 1,1,2,2-tetrachloroethane (black) and tetrahydrofuran (red) ($C = 1.0 \times 10^{-2}$ M).*

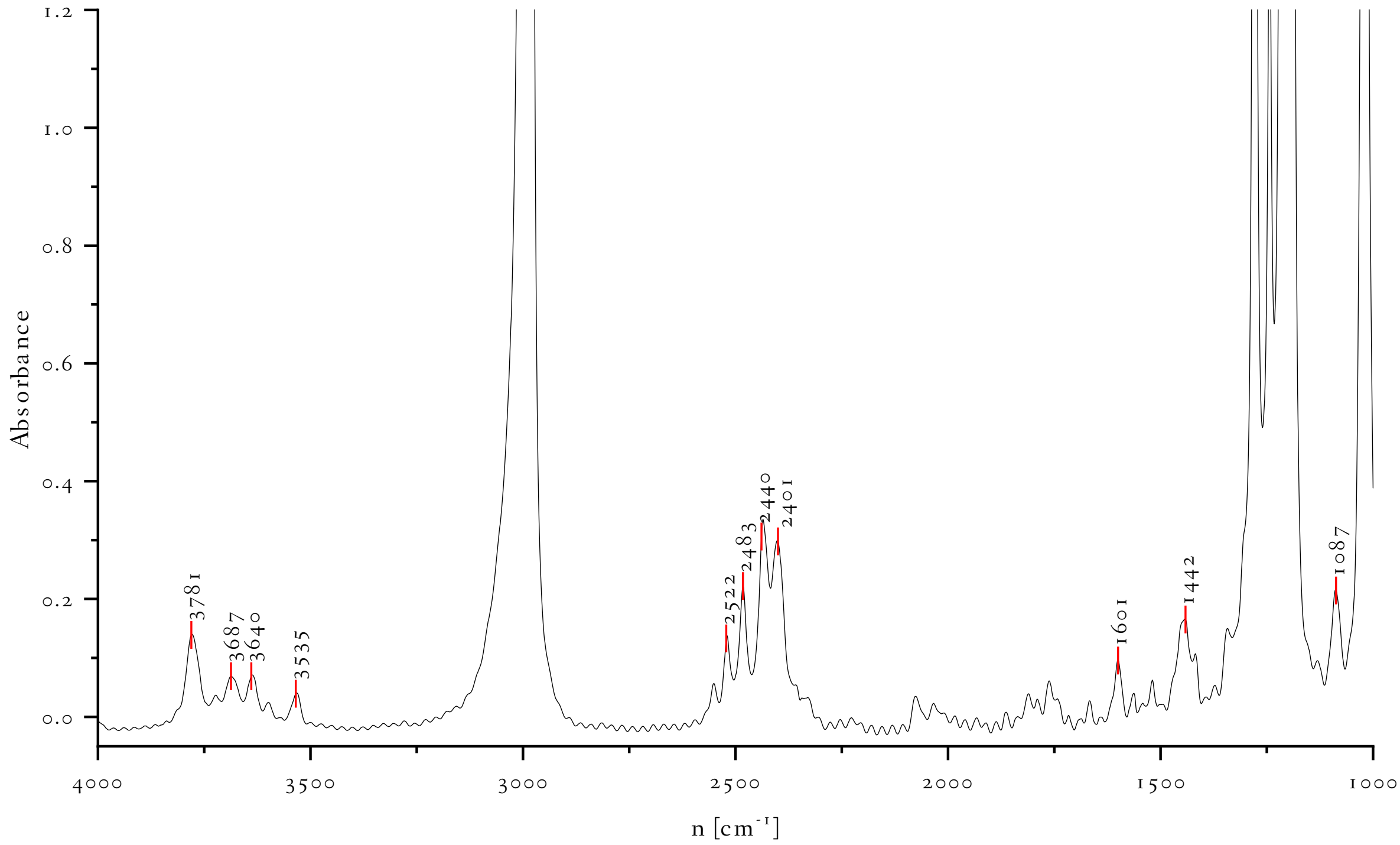

Figure S122. *FT-IR spectrum of **1,1,2,2-tetrachloroethane**.*

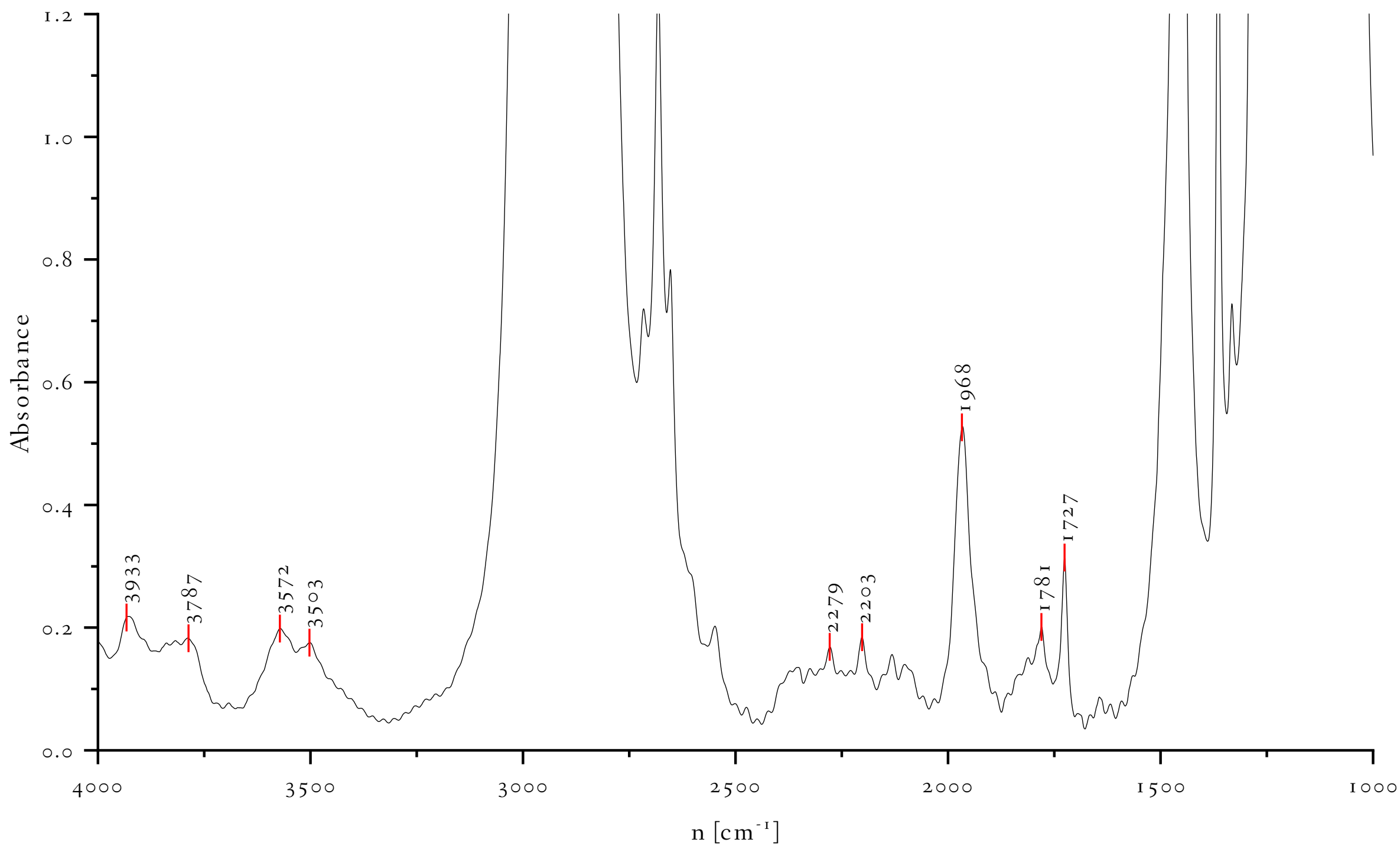

Figure S123. *FT-IR spectrum of **tetrahydrofuran**.*

6 Crystallographic data

6.1 Single crystal XRD analysis of 1 and 5

Single crystals of **1**×3H_2O, were obtained by dissolving **1** in hot water followed by slow cooling of the aqueous solution. X-ray analysis revealed that the compound crystallizes as a hydrate in the trigonal space group $R\bar{3}$, with six molecules per unit cell. Molecule **1** exhibits C_3 symmetry, featuring a 3-fold axis passing through the centre of the aromatic ring. All three amide arms are chemically equivalent and positioned on the same side of the benzene ring plane. The torsion angle C2(Ar)-C1(Ar)-C3-O3 is 21.0(2)° (Figure S129). The *ttg*+ conformation of the side chains and orientation of the amide groups are consistent with the formation of centrosymmetric, spiral-type [6 – 6/0] dimers with a S_6 symmetry, through N3-H3(amide)···O5(acid) hydrogen bonds (H3···O5^{i} 2.888(2) Å, i = $y - 1/3, -x + y + 1/3, -z + 1/3$) between adjacent arms (Figure S129, and Table S125). In addition to the N-H···O hydrogen bonds, each of the acid-carbonyl oxygen atoms is involved in the C2-H2···O5 interaction (C2···O5^{j} 2.373(16) Å, j = *2/3 – y + x, 1/3 + x, 1/3 - z*) with the residual aromatic protons, which influences the orientation of the carboxyl groups. The aromatic ring cores of the molecules forming the dimer, overlapping in the [001] projection, are face-to-face π-stacked with a centroid-to-centroid distance of 3.618(2) Å. No π···π interactions between the aromatic rings of adjacent dimers are observed. Hydrogen bonding interactions involving water molecules also play a crucial role in crystal packing. Cooperative O6-H6···O7-H7B···O3 and O6-H6···O7-H7A···O3 hydrogen bonds, involving the carboxyl groups, connect the dimers with solvent molecules, leading to the formation of supramolecular polymeric chains along the [100] direction (Table S125).

The lack of steric hindrance in the form of an alkyl substituent in the α-position of the glycine fragment also affects the self-assembly of molecules in the crystal lattice. Interestingly, despite the absence of side chains imposing a defined orientation and increased lability, all arms adopt the same directional alignment, leading to a fully symmetrical configuration. The α-H atoms are directed outward from the forming dimer, which promotes the formation of non-covalent interactions involving these hydrogen atoms with molecules of neighbouring dimers. The H4B atom of the methylene group of the arm of the **1** molecule is hydrogen-bonded with the acid-hydroxyl-O atom from the arm of the neighbouring molecule (C4-H4B···O6^{k} interaction with H4B···O6 of 2.62(2) Å, k = *1 – y, x – y, z*), which is not part of the dimer. In turn, the hydrogen atom H4A is involved in a C-H···π interaction (H4A···C2^{l} 2.93(2) Å, l = *1 – y + x, x, -z*) with the aromatic ring of yet another molecule.

In compound **5** addition of trifluoroacetic acid (TFA) to a 1,1,2,2-tetrachloroethane (TCE) solution of **5**, followed by slow evaporation of this mixture, yielded single-crystals. X-ray structural analysis showed the presence of these solvents in a crystal lattice and confirmed the formation of a **5**×2TFA×TCE solvate. The molecule **5** does not possess any internal symmetry: one amide arm (B-labelled) adopts the orientation opposite to the other two (A- and C-labelled) relative to the benzene ring plane. This asymmetry is reflected in the C(Ar)-C(Ar)-C-O torsion angles of 36.5(3)°, -19.7(2)° and -30.3(2)° (Figure S129).

The orientation of the amide groups in the side chain is strongly dependent on the TFA molecules embedded between arms A and B, as well as B and C, non-covalently bonding to the arms via N-H(amide)···O=C(TFA) and O-H(TFA)···O=C(amide) hydrogen bonds (Table S126). In addition, from the perspective of the [100] direction, it can be seen that the TFA molecules are located on the opposite sides of the benzene core of molecule **5**. The conformation of the side chains of **5** also largely depends on the presence of the cyclohexyl substituent in the α-position. The orientation of the aliphatic substituents practically on one side of the benzene ring caused the carboxyl groups to be directed in the opposite direction. Further analyses showed that such an orientation of the functional groups favours the formation of a supramolecular ribbon stretching along the [100] direction with a complex network of intermolecular interactions. Within these ribbons, the A-labelled arm forms a non-covalent connection with the A-arm of a neighbouring molecule *via* O-H(acid)···O=C(amide) hydrogen bonds. Simultaneously, the C-arm engages in N-H(amide)···O=C(acid) hydrogen bonding with the corresponding arm of another molecule (Table S126). This assembly is further stabilized by O-H(acid)···O=C(acid) hydrogen bonds between the carboxyl groups of the A and C-arms.

Interactions of the carboxyl groups from the B-arms, forming centrosymmetric carboxylic acid $R_2^2(8)$ dimers, organize the ribbons into a two-dimensional supramolecular polymer. In addition to the complex network of strong N-H···O and O-H···O hydrogen bonds, the self-assembly of molecules in crystal structure is also influenced by many weaker C-H···O interactions involving residual aromatic protons, methylene groups of the cyclohexyl fragment, or solvent molecules (Figure S130). Taken together, the solid-state structures of **1** and **5** highlight how substitution at the α-position profoundly alters hydrogen-bonding directionality and packing preferences, providing a structural basis for the contrasting self-assembly behaviours observed in solution.

	**1·3H$_2$O**	**5·2TFA·TCE**
**Empirical formula**	$C_{15}H_{15}N_3O_9{\cdot}3H_2O$	$C_{30}H_{39}N_3O_9{\cdot}2C_2H_1F_3O_2{\cdot}C_2H_2Cl_4$
**Formula weight**	435.35	981.53
**Temperature/K**	135.3(1)	138(2)
**Crystal system**	trigonal	triclinic
**Space group**	$R\bar{3}$	$P\bar{1}$
**a/Å**	13.6272(3)	12.5950(2)
**b/Å**	13.6272(3)	14.3099(4)
**c/Å**	18.4841(4)	14.9067(4)
**α/°**	90	63.601(3)
**β/°**	90	86.986(2)
**γ/°**	120	69.234(2)
**V/Å^3**	2972.65(15)	2232.40(11)
**Z, Z'**	6, 0.33333	2, 1
**ρ$_{calc}$/gcm^{-3}**	1.459	1.460
**μ/mm^{-1}**	1.116	3.197
**F(000)**	1368.0	1012.0
**Crystal size/mm^3**	0.23 × 0.15 × 0.15	0.21 × 0.13 × 0.08
**Radiation/Å**	CuKα (λ = 1.54184)	Cu Kα (λ = 1.54184)
**2θ range/°**	8.89 to 152.092	6.672 to 153.028
**Index ranges**	-11 ≤ h ≤ 16, -16 ≤ k ≤ 13, -22 ≤ l ≤ 20	-13 ≤ h ≤ 15, -18 ≤ k ≤ 17, -18 ≤ l ≤ 18
**Reflections collected**	2071	35540
**Independent reflections**	1278 [R_{int} = 0.0169, R_{sigma} = 0.0228]	9249 [R_{int} = 0.0371, R_{sigma} = 0.0273]
**Reflections with [I > 2σ (I)]**	1171	7781
**Data/restraints/parameters**	1278/0/120	9249/67/813
**Final R indexes [I ≥ 2σ (I)]**	R_1 = 0.0395, wR_2 = 0.1025	R_1 = 0.0489, wR_2 = 0.1329
**Final R indexes (all data)**	R_1 = 0.0429, wR_2 = 0.1057	R_1 = 0.0576, wR_2 = 0.1428
**Goodness-of-fit on F^2**	1.056	1.050
**Largest diff. peak/hole/eÅ^{-3}**	0.82/-0.32	0.63/-0.58
**CCDC deposit no.**	2422058	2421892

Figure S124. *Table with crystal data and structure refinement for **1** and **5**.*

D—H···*A*	*D*—H (Å)	H···*A* (Å)	*D*···*A* (Å)	*D*—H···*A* (°)
N3—H3···O5^{i}	0.85(2)	2.08(2)	2.8884(18)	157(2)
O7—H7A···O3^{ii}	0.89(3)	1.94(3)	2.8328(19)	178(3)
O7—H7B···O3^{iii}	0.85(4)	2.04(4)	2.8174(19)	152(3)
O6—H6···O7	1.05(4)	1.51(4)	2.5595(19)	176(3)
Symmetry code(s): (i) *y*−1/3, −*x*+*y*+1/3, −*z*+1/3; (ii) −*x*+*y*+2/3, −*x*+4/3, *z*+1/3; (iii) −*x*+5/3, −*y*+4/3, −*z*+1/3.				

Figure S125. *Table with hydrogen bond parameters for* ***1·3H$_2$O.***

D—H···*A*	*D*—H (Å)	H···*A* (Å)	*D*···*A* (Å)	*D*—H···*A* (°)
N7A—H7A···O20A	0.84(3)	2.19(3)	3.009(2)	167(2)
N7B—H7B···O20B	0.85(3)	2.21(3)	3.044(2)	168(3)
N7C—H7C···O14C^{i}	0.81(2)	2.28(3)	3.083(2)	168(2)
O15A—H15A···O7A^{iii}	0.90(4)	1.79(4)	2.686(2)	171(3)
O15B—H15B···O14B^{ii}	0.83(4)	1.80(4)	2.628(2)	178(3)
O15C—H15C···O14A^{i}	0.80(3)	1.98(3)	2.742(2)	159(3)
O21A—H21A···O7B	0.99(5)	1.57(5)	2.529(2)	160(4)
O21B—H21B···O7C	0.88(4)	1.67(4)	2.522(2)	162(4)
Symmetry code(s): (i) −*x*+1, −*y*+1, −*z*+1; (ii) −*x*+1, −*y*+2, −*z*; (iii) −*x*, −*y*+1, −*z*+1.				

Figure S126. *Table with N-H···O and O-H···O hydrogen bond parameters for* ***5·2TFA·TCE****.*

D—H···*A*	*D*—H (Å)	H···*A* (Å)	*D*···*A* (Å)	*D*—H···*A* (°)
C2—H2···O20A	0.94(2)	2.58(2)	3.228(2)	126.2(17)
C4—H4···O20B	0.95(2)	2.54(2)	3.200(3)	126.3(18)
C9C—H9CB···O7C	0.92(3)	2.46(3)	3.048(2)	122(2)
C11A—H11E···O14B^{i}	1.02(4)	2.62(4)	3.386(4)	132(3)
C12A—H12E···O20A	1.01(4)	2.74(4)	3.397(4)	123(3)
C13A—H13F···O20A	0.99(3)	2.73(3)	3.346(3)	121(2)
C13B—H13C···O20B	0.94(3)	2.79(3)	3.372(3)	120.7(18)
C30—H30···O14A^{ii}	1.00	2.51	3.367(4)	143.0
C30A—H30A···O14A^{ii}	1.00	2.29	3.29(3)	172.8
C31—H31···O7A^{iii}	1.00	2.26	3.212(5)	157.8
C31A—H31A···O7A^{iii}	1.00	2.32	3.080(18)	132.0
Symmetry code(s): (i) *x*−1, *y*, *z*; (ii) −*x*+1, −*y*+1, −*z*+1; (iii) *x*+1, *y*, *z*;				

Figure S127. *Table with C-H···O hydrogen bond parameters for* ***5·2TFA·TCE****.*

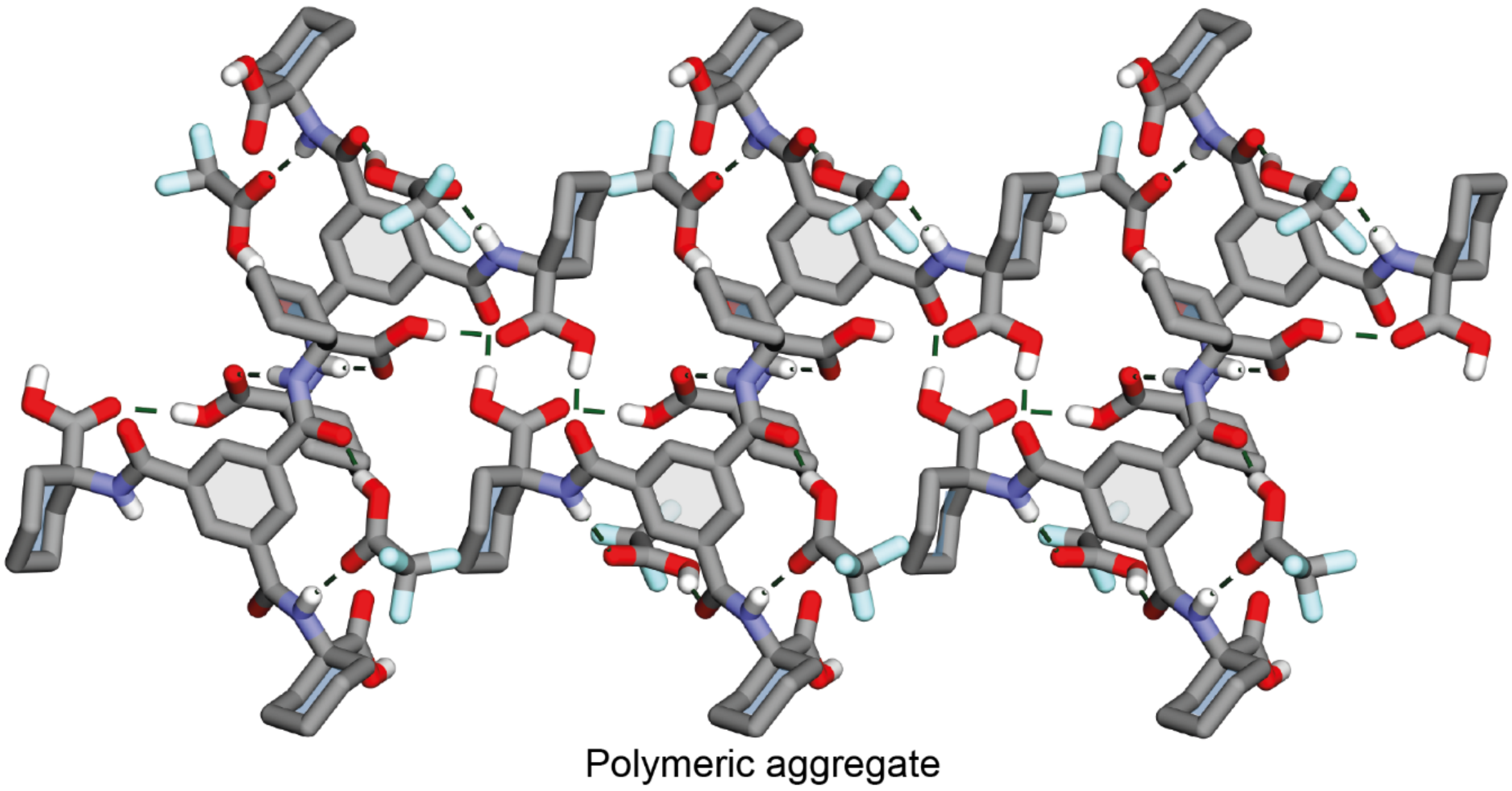

Figure S128. *Polymeric structure of **5** along the [100] direction, stabilized by intermolecular hydrogen bonding involving carboxylic acid and amide functional groups. TCE molecules, disorder in TFA molecules, as well as C-H aromatic and aliphatic interactions, were omitted for clarity.*

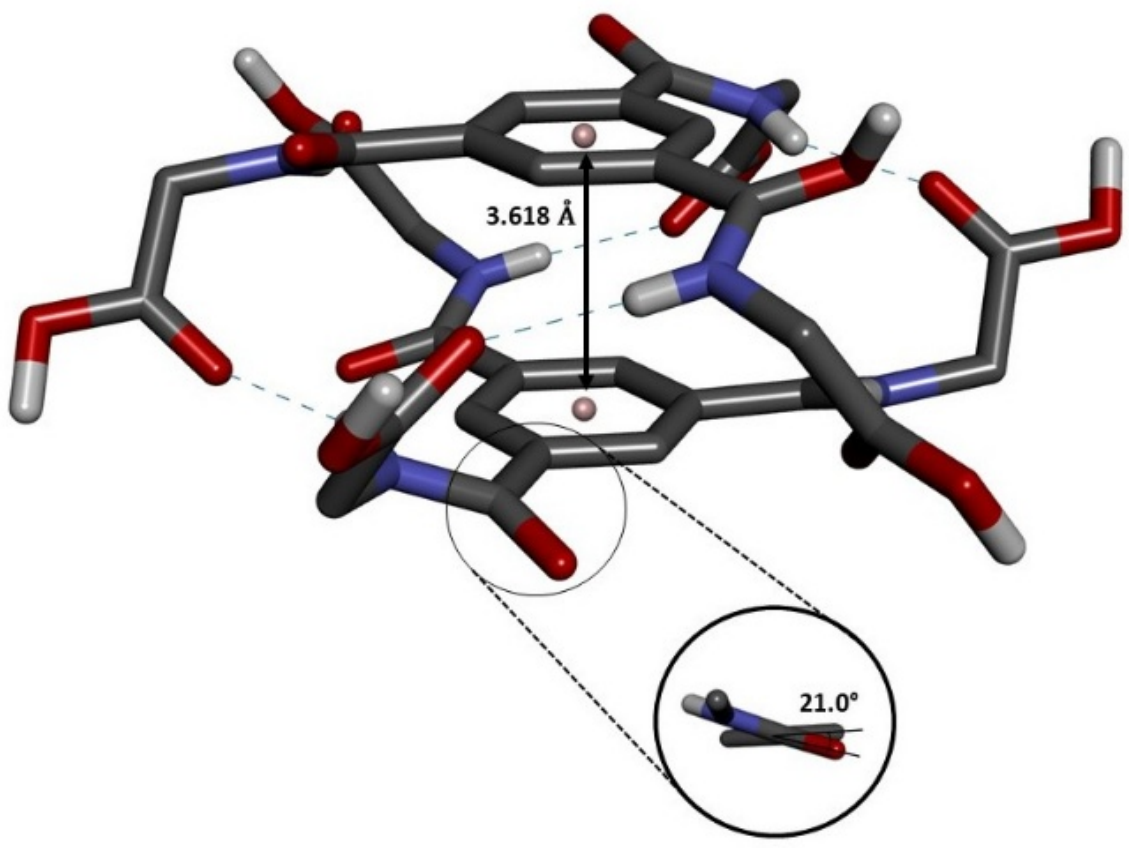

Figure S129. *Centrosymmetric spiral-type dimer of **1** formed through six N-H···O intermolecular hydrogen bonds. Side view showing the inter-centroid separation with distance 3.618(2) Å and the C2(Ar)-C1(Ar)-C3-O3 torsion angle of 21.0(2)°.*

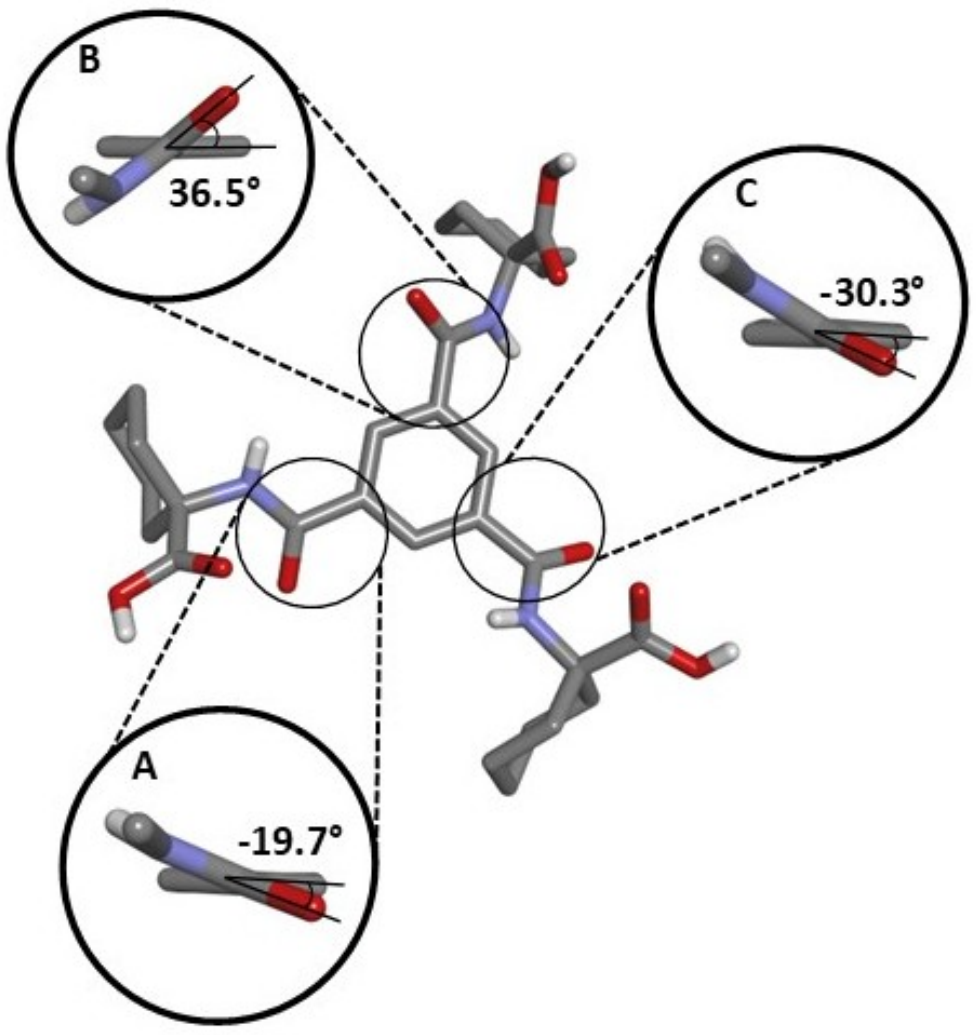

Figure S130. *Top view of **5** molecule representing side chains (labelled as a A, B and C) conformation with marked torsion angles showing amide groups orientation relative to the aromatic ring.*

7 Computational details

DFT Calculations

Individual models of the benzene-1,3,5-tricarboxamides (BTA) monomer core were extracted from the previously reported octameric amide structure. The R- groups of the monomers were functionalised with **1**, **2**, **3**, **4**, **5**, **6**, **7**, **8**, **9**, **10**, **11** monomer R groups using Maestro, where C_3 symmetry of the BTA arms was preserved.[8] (Figure S131)

Electrostatic potential (ESP) maps for monomers **1**-**12** and octamers of **1**, **9** and **11** were produced with CP2K (PBE/D3/TZVP) single point energies.(Figure S132, S133) The PBE functional,[9] def2-TZVP basis set,[10] a grid size of 400 Ry and a relative cut-off value of 100 Ry was used, with tight SCF convergence of 10^{-7} Hartree. Non-bonded interactions were accounted for with the Grimme-D3 dispersion correction.[11] The electron densities were cutoff beyond 0.03 a.u and -0.03 a.u, corresponding to an energy range of 160 kJ mol^{-1}, which is a reasonable range for probing polarity effects for organic materials.

Octamers were constructed by templating the centre of masses of BTA linkers from a previously reported study.[5] Due to the computational expense of octameric capsule construction, only octameric models for **1**, **9** and **11** were constructed, as illustrated in Figure S133. The window diameters for octameric models **9** and **11** were calculated within Maestro measuring atom centre-to-atom centre distances of the closest atoms within the window portals.[8]

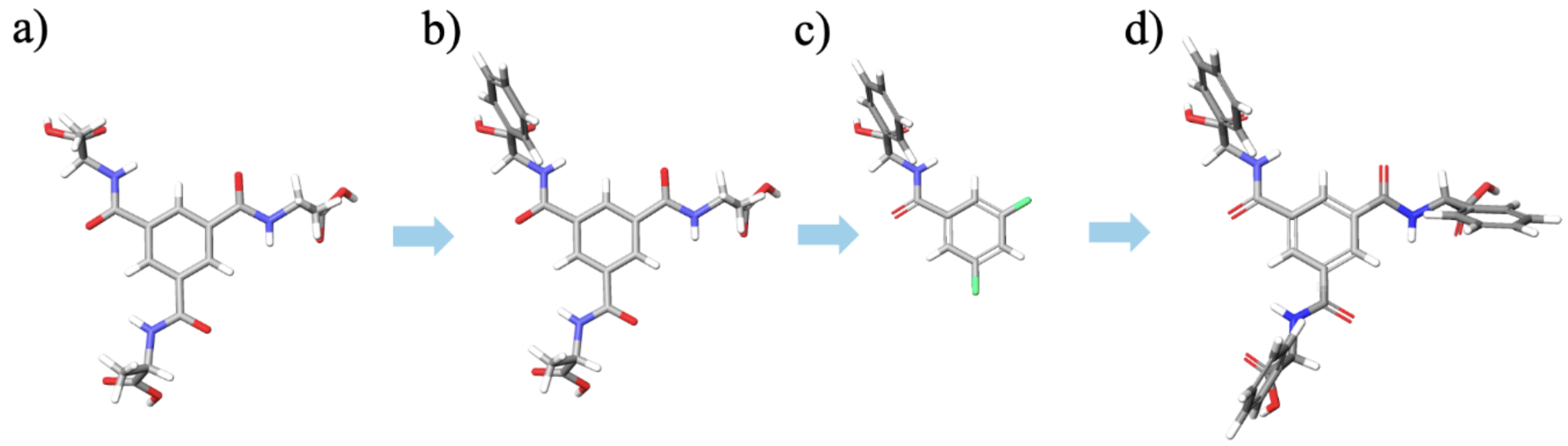

Figure S131. *Illustration of R group addition, benzene R- group substitution as an example for visual clarity: a) Placement of the BTA core, b) functionalisation of one arm, c) removal of pristine arms and replacement with dummy atoms, d) reintroducing the R groups onto the dummy atoms, using the operation in part b); therefore preserving C_3 symmetry as in the pristine BTA.*

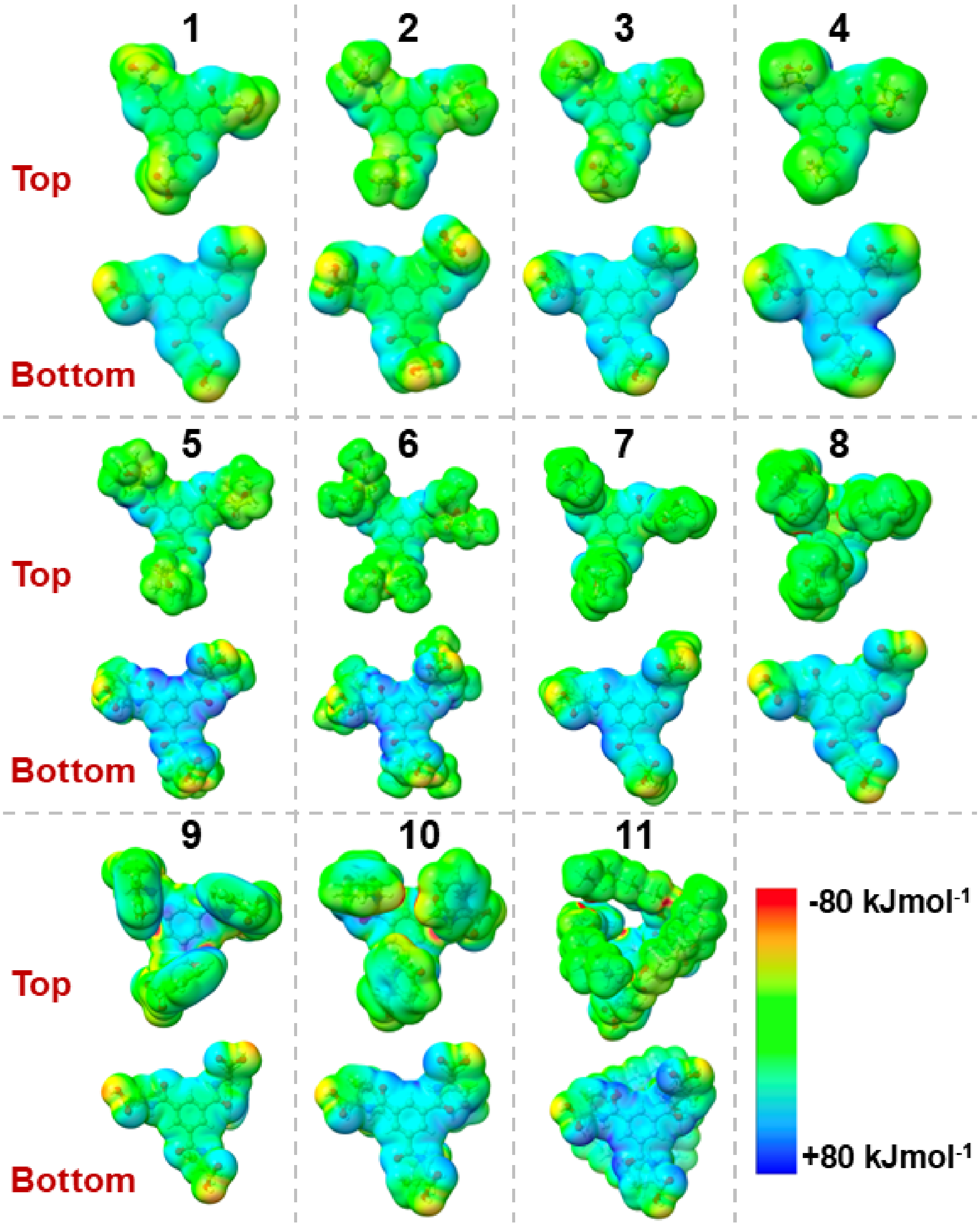

Figure S132. *Electrostatic potential maps for* ***1-12****, highlighting "Top" and "Bottom" parts of BTA cores (top and lower image for each structure, respectively). The electron energies have been cutoff beyond -0.03 and 0.03 a.u, with the electrostatic potential evaluated from a surface 1.4 Å outside the molecule surface for clarity. "Top view" corresponds to the R peripheral direction, whereas "Bottom" views correspond to carboxylate arms dentation direction.*

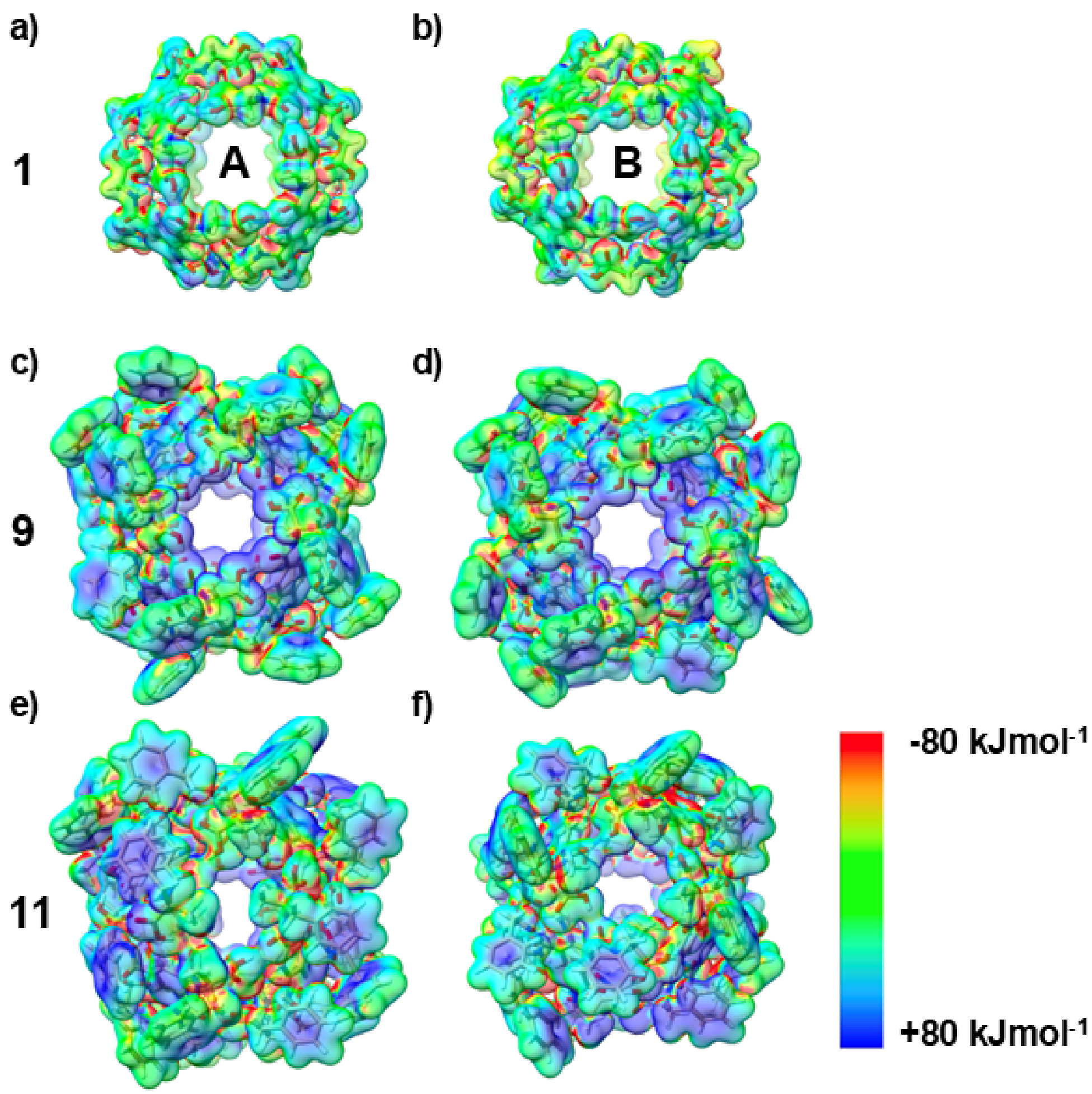

Figure S133. Electrostatic potential maps for octamers a-b) **1** c-d) **9** and e-f) **11**, where a-c-e) show pore A, and b-d-f) showing pore B. The electron densities have been cut off beyond -0.03 and 0.03 a. u. (-80 to +80 kJ mol^{-1}) with the electrostatic potential evaluated on the surface 1.4 Å outside the molecular surface.

8 References

1. C. P. Agilent and P. CrysAlis, Agilent Technologies Ltd, *Yarnton, Oxfordshire, England*, 2014, **2014**.
2. O. V. Dolomanov, L. J. Bourhis, R. J. Gildea, J. A. Howard and H. Puschmann, OLEX2: a complete structure solution, refinement and analysis program, *J. Appl. Crystallogr.*, 2009, **42**, 339-341.
3. G. M. Sheldrick, SHELXT - integrated space-group and crystal-structure determination, *Acta Crystallogr. A*, 2015, **71**, 3-8.
4. G. M. Sheldrick, Crystal structure refinement with SHELXL, *Acta Crystallogr. C*, 2015, **71**, 3-8.
5. G. Markiewicz, A. Jenczak, M. Kolodziejski, J. J. Holstein, J. K. M. Sanders and A. R. Stefankiewicz, Selective C70 encapsulation by a robust octameric nanospheroid held together by 48 cooperative hydrogen bonds, *Nat. Commun.*, 2017, **8**, 15109.
6. M. de Loos, J. H. van Esch, R. M. Kellogg and B. L. Feringa, C3-Symmetric, amino acid based organogelators and thickeners: a systematic study of structure–property relations, *Tetrahedron*, 2007, **63**, 7285-7301.
7. R. N. Lewis, R. N. McElhaney, W. Pohle and H. H. Mantsch, Components of the carbonyl stretching band in the infrared spectra of hydrated 1,2-diacylglycerolipid bilayers: a reevaluation, *Biophys. J.* , 1994, **67**, 2367-2375.
8. S. Wang, J. Witek, G. A. Landrum and S. Riniker, Improving Conformer Generation for Small Rings and Macrocycles Based on Distance Geometry and Experimental Torsional-Angle Preferences, *J. Chem. Inf. Model.* , 2020, **60**, 2044-2058.
9. J. P. Perdew, K. Burke and M. Ernzerhof, Generalized gradient approximation made simple, *Phys. Rev. Lett.*, 1996, **77**, 3865.
10. J. VandeVondele and J. Hutter, Gaussian basis sets for accurate calculations on molecular systems in gas and condensed phases, *J. Chem. Phys.*, 2007, **127**.
11. M. Bursch, J. M. Mewes, A. Hansen and S. Grimme, Best-practice DFT protocols for basic molecular computational chemistry, *Angew. Chem. Int. Ed.*, 2022, **134**, e202205735.
